# Pd(II) Complexes with Pyridine Ligands: Substituent Effects on the NMR Data, Crystal Structures, and Catalytic Activity

**DOI:** 10.1021/acs.inorgchem.2c01996

**Published:** 2022-08-19

**Authors:** Gracjan Kurpik, Anna Walczak, Mateusz Gołdyn, Jack Harrowfield, Artur R. Stefankiewicz

**Affiliations:** † Faculty of Chemistry, Adam Mickiewicz University in Poznań, Uniwersytetu Poznańskiego 8, Poznań 61-614, Poland; ‡ Center for Advanced Technology, Adam Mickiewicz University in Poznań, Uniwersytetu Poznańskiego 10, Poznań 61-614, Poland; § Institut de Science et d’Ingénierie Supramoléculaires, Université de Strasbourg, 8 allée Gaspard Monge, Strasbourg 67083, France

## Abstract

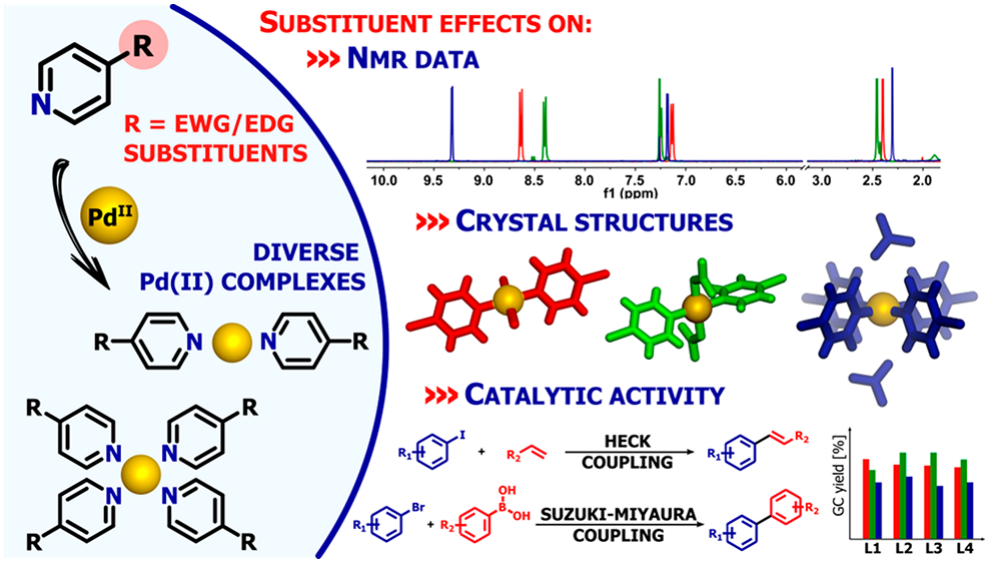

A wide range of functionalized pyridine ligands have been employed to synthesize a variety of Pd(II) complexes of the general formulas [Pd**L**_4_](NO_3_)_2_ and [Pd**L**_2_Y_2_], where **L** = 4-X-py and Y = Cl^–^ or NO_3_^–^. Their structures have been unambiguously established via analytical and spectroscopic methods in solution (NMR spectroscopy and mass spectrometry) as well as in the solid state (X-ray diffraction). This in-depth characterization has shown that the functionalization of ligand molecules with groups of either electron-withdrawing or -donating nature (EWG and EDG) results in significant changes in the physicochemical properties of the desired coordination compounds. Downfield shifts of signals in the ^1^H NMR spectra were observed upon coordination within and across the complex families, clearly indicating the relationship between NMR chemical shifts and the ligand basicity as estimated from p*K*_a_ values. A detailed crystallographic study has revealed the operation of a variety of weak interactions, which may be factors explaining aspects of the solution chemistry of the complexes. The Pd(II) complexes have been found to be efficient and versatile precatalysts in Suzuki–Miyaura and Heck cross-coupling reactions within a scope of structurally distinct substrates, and factors have been identified that have contributed to efficiency improvement in both processes.

## Abstract 2

A wide range of pyridine derivatives have been employed to synthesize a variety of di- and tetrasubstituted Pd(II) complexes of square-planar geometry. This in-depth characterization has shown that the functionalization of ligand molecules with groups of either electron-withdrawing or -donating nature results in significant changes in the physicochemical properties of the coordination compounds. Moreover, the complexes have been found to be of practical utility as efficient precatalysts for both Suzuki−Miyaura and Heck cross-coupling reactions.

## Introduction

Since its discovery by Thomas Anderson in 1849, pyridine has been one of the most popular heterocyclic compounds used in chemistry. Despite the many similarities between pyridine and benzene, the introduction of an electronegative N atom to the aromatic ring significantly differentiates their physicochemical properties.^1^ The presence of a pair of nonbonding electrons in the valence shell of the N atom enables pyridine derivatives to act as Lewis bases toward a wide variety of metal ions.^2^ Both pyridine and polypyridine ligands are good neutral donors of mono- or multidentate nature, and their coordination properties can be relatively easily altered by substitution with electron-donating or -withdrawing groups.^3^ The structural and electronic modifications achieved by the functionalization of heterocyclic rings enable modulation of the metal coordination sphere, which can lead to improvement of the desired properties and potential applicability.^4^ Furthermore, pyridine and its derivatives can be utilized as model units for research on important biomolecules such as nicotine, pyridoxine, or nicotinamide adenine dinucleotide phosphate (NADP).^4a,5^ A wide variety of transition-metal complexes with pyridine-based ligands having both academic and industrial importance have been successfully generated, as reflected in the rich literature in this field.^6^ Notably, coordination structures based on pyridyl units have shown real application potential as catalysts,^7^ compounds of cytotoxic activity,^8^ and molecule magnets.^9^

Where direct complexation equilibrium measurements of the Lewis basicity of a ligand are unavailable, the Brønsted basicity, measured as p*K*_a_ values, usually rather readily obtained for pyridine derivatives, has been widely applied as a measure of the effect of any substituent on pyridine donor behavior.^10^ In a recent study of Pt(II) complexes of 4-substituted pyridines having some parallels with the present study of Pd(II) species,^11^ it was found that the ^1^H NMR chemical shifts of the 2/6 protons showed a same linear dependence on the p*K*_a_ for the coordinated as well as free ligands, consistent with protonation being a useful guide to coordination behavior. Pd(II) complexes with pyridine derivatives have been used as efficient catalysts in reactions such as the carbonylation of nitro compounds,^12^ reduction of nitro compounds to amines,^13^ or carbonylation of aniline derivatives by CO/O_2_.^14^ A successful example of the correlation between the catalytic efficiency and ligand basicity is provided in the conversion of nitrobenzene to ethyl *N*-phenylcarbamate catalyzed by a series of [Pd**L**_2_Cl_2_] complexes, where **L** = various di- and monosubstituted pyridines.^12a^ An increase in the reaction yield was observed when Pd(II) complexes based on more basic ligands were used as catalysts, although steric effects were also apparent in species with 2/6 substituents, leading to the conclusion that 3 or 4 substitution provided the best correlation with basicity.

**Scheme 1:**
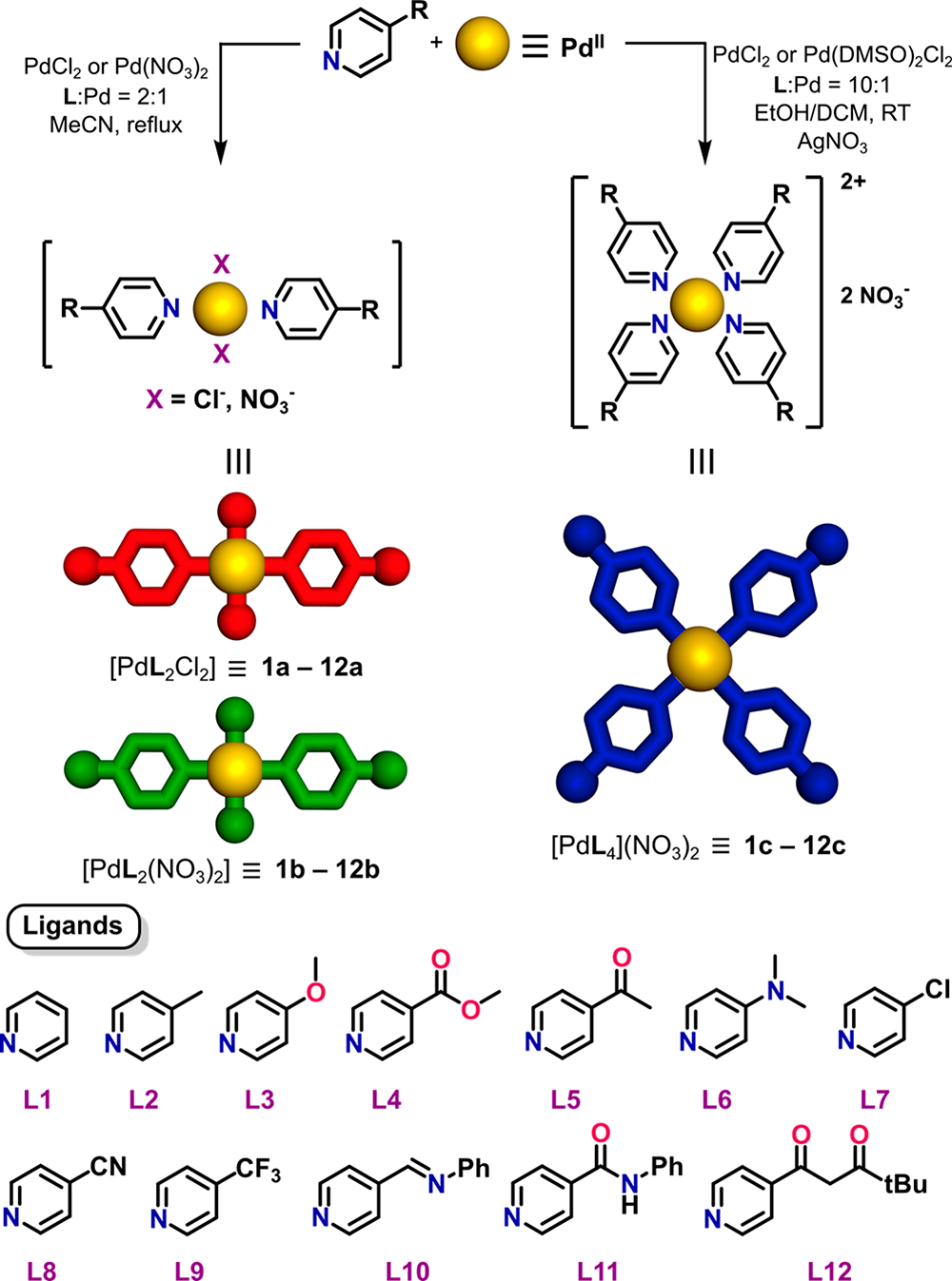
Synthetic Routes for the Pd(II) Complexes Based on the Pyridine Ligands **L1**–**L12**

In this work, we have employed a series of 4-substituted pyridine ligands, **L1**–**L12** (Scheme 1), to generate an array of coordination compounds with Pd(II) cations of a square-planar geometry. The particular reaction conditions employed provided di- and tetrasubstituted complexes diversified in terms of their charge and composition. We anticipated that the properties of such complexes could be tuned by modifying the nature of the ligand substituents, and our efforts to prove this are presented below. Detailed analyses have been made of the structures and ^1^H NMR spectra of the complexes in relation to substituent effects, and we have extended this investigation to that of functionality by employing the complexes as catalyst precursors in Suzuki–Miyaura and Heck cross-coupling reactions involving a range of organic reagents.

## Results and Discussion

### Synthesis of Complexes

**Figure 1:**
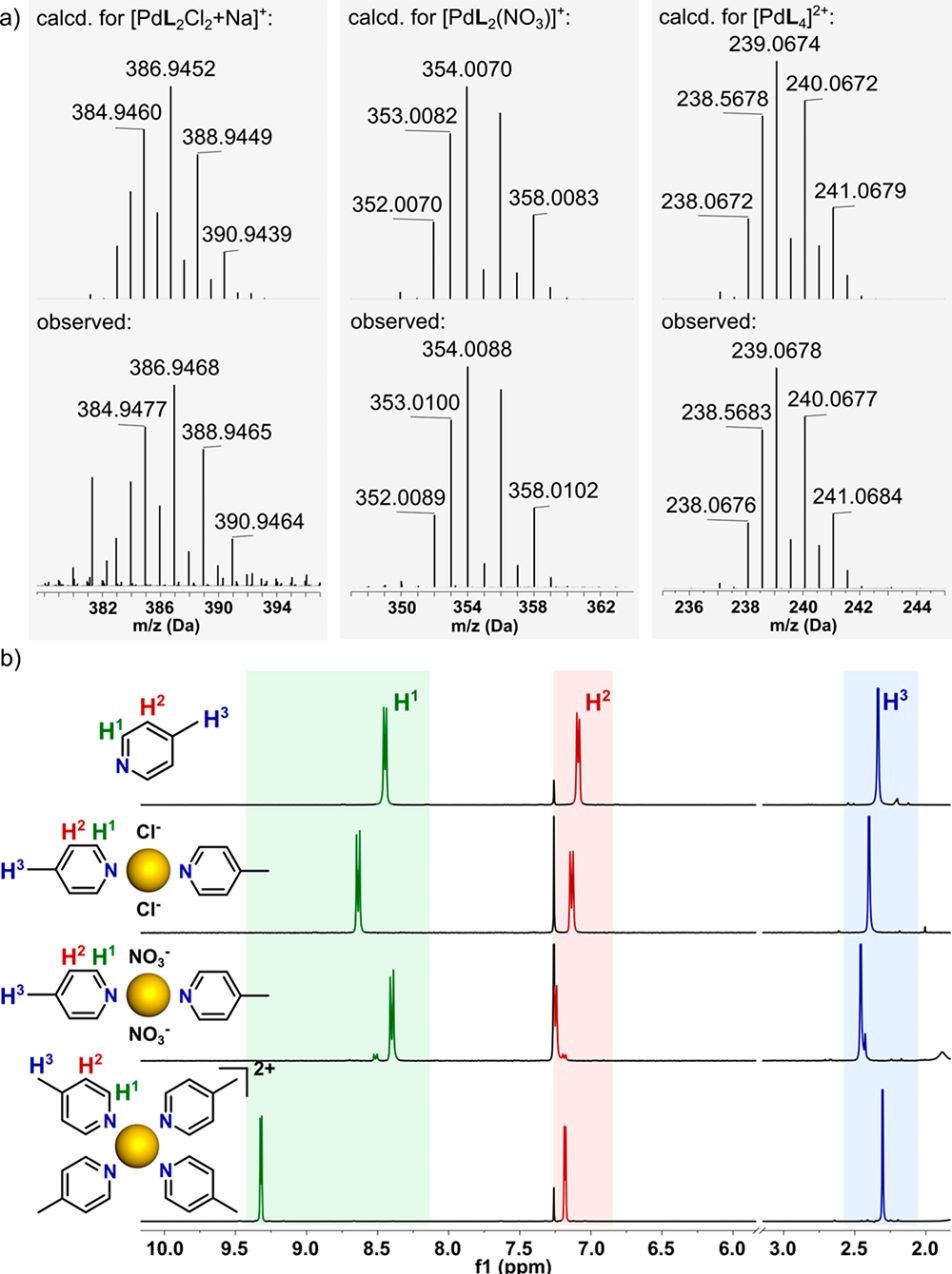
(a) ESI-MS spectra of Pd(II) complexes **2a**–**2c**, showing the calculated isotope model (top) and observed data (bottom). (b) ^1^H NMR spectra (300 MHz, CDCl_3_) of compounds **2a**–**2c**.

The substituents on the 4 position of ligands **L1**–**L12** span a range of both electron-withdrawing and -donating groups, but pyridine-N-bound complexes of all of the ligands can be isolated under the appropriate reaction conditions. While, in principle, mono, bis, tris, and tetrakis species are possible, the use of an exact 2:1 **L**/Pd(II) reaction stoichiometry enabled the isolation of neutral [Pd**L**_2_Y_2_], where Y = Cl^–^ or NO_3_^–^, whereas the use of a large excess of the ligand [10:1 **L**/Pd(II)] was required to shift the reaction equilibrium toward the exclusive generation of tetra-substituted compounds, allowing for the ready isolation of cationic complexes [Pd**L**_4_]^2+^ as their nitrate salts. The synthetic procedures are outlined in Scheme 1 and described in detail in the SI. On the basis of their ^1^H NMR spectra [see the Supporting Information (SI) and Figure 1b] allied to the X-ray structural results (see below), all of the [Pd**L**_2_Cl_2_] products (**1a**–**12a**) appeared to contain only the trans isomer, whereas the [Pd**L**_2_(NO_3_)_2_] products (**1b**–**12b**) contained minor but detectable amounts of the cis isomer. This preference for the trans configuration mimics that known for various Pt(II) analogues.^11^ The use of PdCl_2_ or [Pd(DMSO)_2_Cl_2_] (DMSO = dimethyl sulfoxide) as reactants for the formation of [Pd**L**_4_]^2+^ cations (**1c**–**12c**) was efficient but required the elimination of chloride from the reaction mixtures by the addition of AgNO_3_. The ease of formation of the [Pd**L**_4_]^2+^ cations appeared to increase with the basicity of the ligand, and this may explain why a pure species **6b** could not be isolated with the most strongly basic unit **L6**. The substituents of ligands **L10**–**L12** involve good coordinating sites that appeared, probably through competition with nitrate, to divert the formation of products **10b**–**12b** into that of inseparable mixtures.

### Mass Spectrometry (MS) Analysis

The successful generation of the desired mononuclear Pd(II) compounds was confirmed via electrospray ionization mass spectrometry (ESI-MS). As shown in Figure 1a, with the example of complexes with **L2**, isotopically resolved peaks were generally found for [M + Na^+^]^+^, [M – NO_3_^–^]^+^, and [M – 2NO_3_^–^]^2+^, where M represents the intact assembly for units of the general formulas [Pd**L**_2_Cl_2_] (**2a**), [Pd**L**_2_(NO_3_)_2_] (**2b**), and [Pd**L**_4_](NO_3_)_2_ (**2c**), respectively. All of the peaks were in good agreement with their calculated distribution, allowing the molecularity to be unambiguously established and to distinguish the specific types of complexes. The MS data for all of the units are available in the SI.

### NMR Spectroscopy

**Figure 2:**
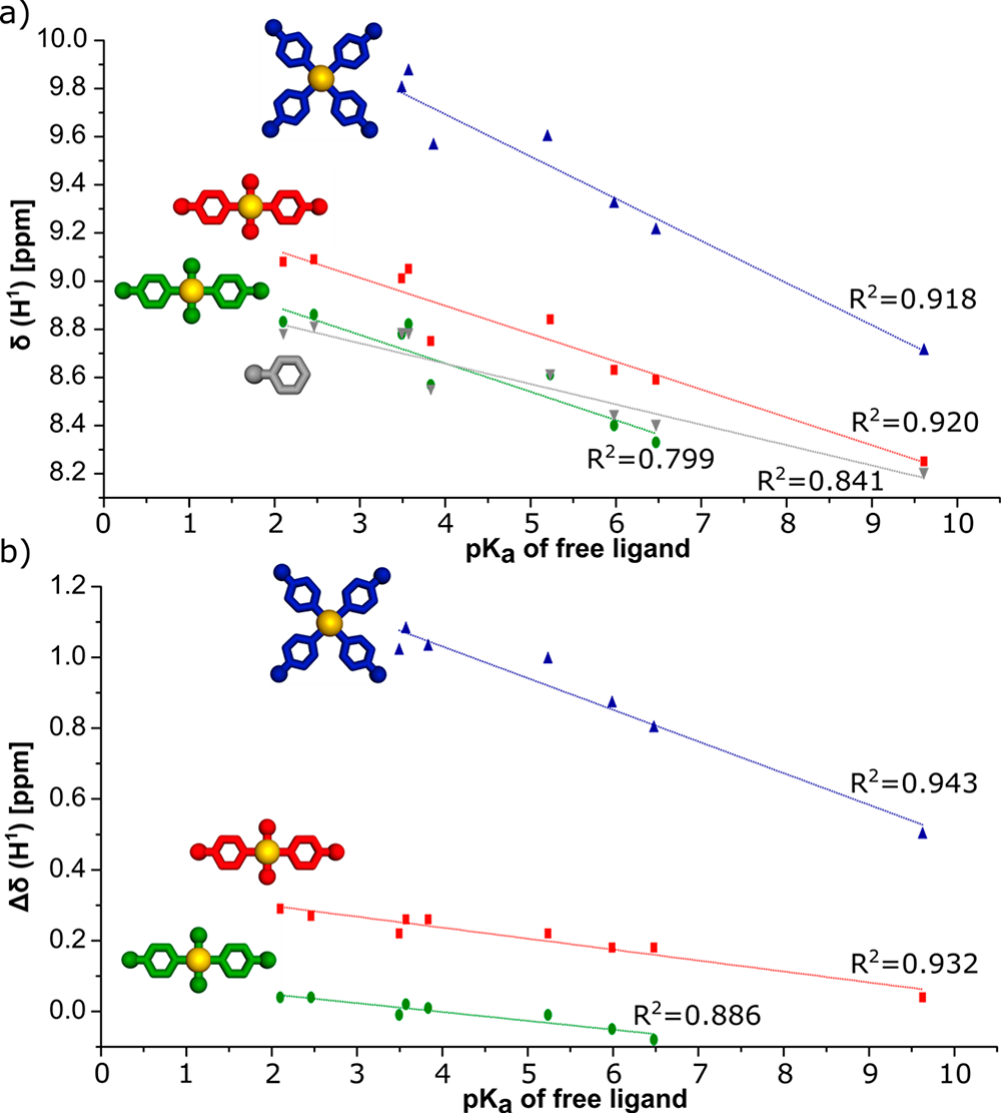
(a) Relationships between the chemical shifts (δ, ppm) of the signal H^1^ in the ^1^H NMR spectra (CDCl_3_, 25 °C) and p*K*_a_ values of free ligands for the Pd(II) complexes. (b) Relationships between the chemical shift changes (Δδ, ppm) of the signal H^1^ in the ^1^H NMR spectra (CDCl_3_, 25 °C) and p*K*_a_ values of free ligands for the Pd(II) complexes. Only ligands of known p*K*_a_ values are included in the graphs.

Apart from signals due to different substituents, the ^1^H NMR spectra were all very similar, with the 2-fold symmetry of the free ligands retained in all of the complexes and all ligand units in any particular complex being equivalent. In general, a greater sensitivity to the composition and structure of the complexes was seen in the chemical shifts of the H^1^ protons (on C adjacent to N) rather than in those of the H^2^ protons, and the presence of small amounts of cis isomer in the products **1b**–**12b**, <10% in all cases, was readily discerned on this basis. Spectra typical of the whole group are shown for the complexes of **L2** in Figure 1b, with the results for all other species being included in the SI. A comparison of the various *trans*-[Pd**L**_2_(NO_3_)_2_] (**1b**–**12b**) and *trans*-[Pd**L**_2_Cl_2_] (**1a**–**12a**) pairs shows that the H^1^ chemical shifts are sensitive to the nature of the adjacent donor atom, and this is presumably a contributor to the very large downfield shifts (∼0.5–1 ppm relative to those of the free ligands) for the H^1^ proton signals of complexes **1c**–**12c**, although the dominant effect here may be that of ion pairing involving C–H^1^···ONO_2_ bonding, as proposed to explain similar observations on Pt(II) analogues.^11^ Again, as observed for Pt(II), the H^1^ chemical shifts (Table 1) show a close-to-linear dependence on the p*K*_a_ values of the protonated ligands (Figure 2), indicating that the substituent effects remain operative along with any effects of Pd(II) coordination.

**Table 1:** ^1^H NMR Chemical Shifts (δ, ppm) in CDCl_3_ of H^1^ Protons for Pd(II) Complexes Based on Ligands **L1**–**L12**

		δ(H^1^) [ppm]			
	p*K*_a_^b^	**L**	Pd**L**_2_Cl_2_ (**1a**–**12a**)	Pd**L**_2_(NO_3_)_2_ (**1b**–**12b**)	Pd**L**_4_(NO_3_)_2_ (**1c**–**12c**)
**L1**	5.23	8.62	8.84	8.61	9.63
**L2**	5.98	8.45	8.63	8.40	9.32
**L3**	6.47	8.41	8.59	8.33	9.21
**L4**	3.49	8.79	9.01	8.78	9.80
**L5**	3.57	8.79	9.05	8.82	9.87
**L6**	9.61	8.21	8.25		8.71
**L7**	3.83	8.49	8.75	8.50	9.52
**L8**	2.10	8.79	9.08	8.83	
**L9**	2.46	8.82	9.09	8.86	
**L10**	3.07	8.77	8.97		9.79
**L11**^a^	3.12	8.79	8.99		9.44
**L12**	2.86	8.75	8.95		9.75

a The spectra of **L11** and its complexes were recorded in DMSO-*d*_6_.

b For ligands **L1**–**L9**, the experimental p*K*_a_ values are provided in the literature.^11,15^ For ligands **L10**–**L12**, the predicted p*K*_a_ values are provided by SciFinder.^16^

### Solution Complexation Equilibria

**Figure 3:**
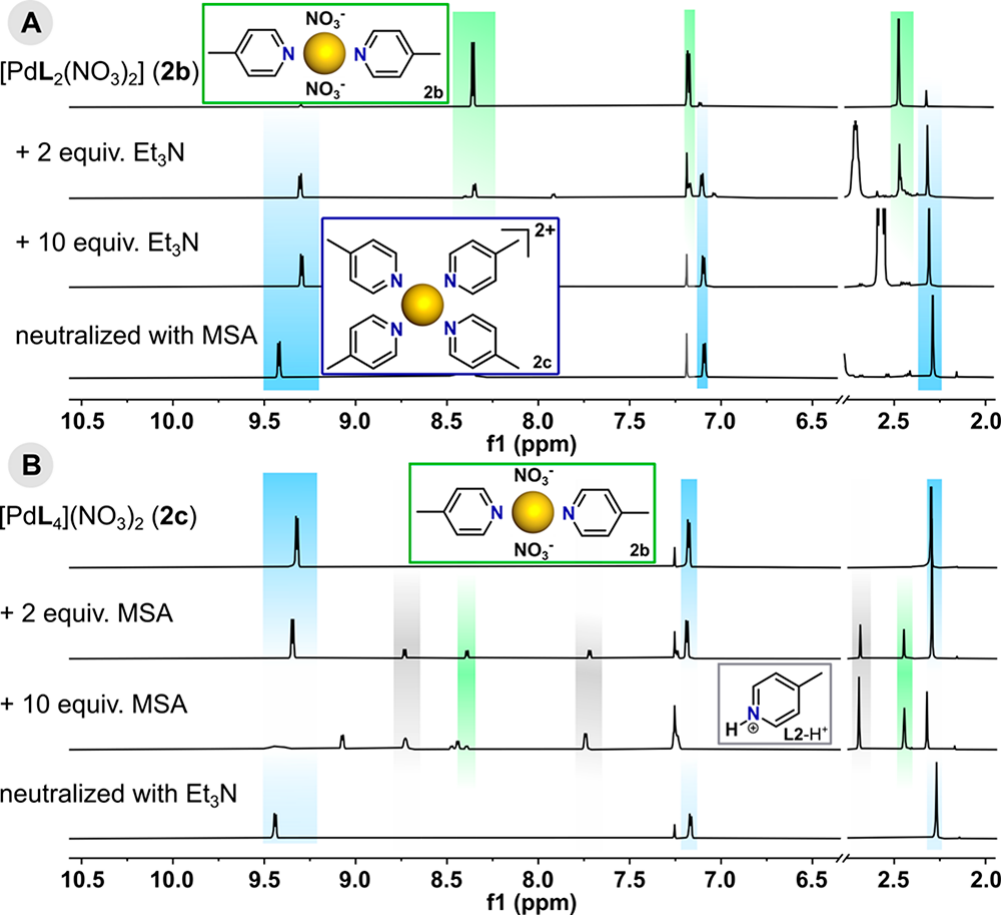
Parts of the ^1^H NMR spectra (600 MHz, CDCl_3_) showing the transformations of (a) **2b** upon the addition of Et_3_N and (b) **2c** upon the addition of MSA.

In regard to ligand substitution processes, Pd(II) is classified as a labile metal ion and its substitution reaction rates are typically orders of magnitude faster than those of Pt(II).^17^ This lability was readily observed for complexes **2a**–**2c** by using ^1^H NMR spectroscopy to follow titrations with acid (methanesulfonic acid, MSA) and base (triethylamine, Et_3_N). Results typical of what was observed generally are shown in Figure 3. Thus, the equilibrium mixture of *cis*- and *trans*-**2b** reacted with Et_3_N to give ultimately some **2c**, while no intermediates such as [Pd**L**_3_Y]^+^ were detected via NMR. Because of the ligand deficiency after the altered **L**/Pd(II) complex stoichiometry, species **2c** were necessarily accompanied by unidentifiable Pd(II) species to which **L2** was not coordinated. Neutralization of the reaction mixture with MSA did not return the original complex, retaining the structure of tetrakis(pyridine) units (Figures 3a and S33). In another experiment, the ^1^H NMR titration of complex **2c** with sequential portions of MSA led to changes indicative of the dissociation and protonation of **L2** and probably some substitution of nitrate by methanesulfonate, which resulted in the complete disappearance of signals from **2c**. Although the bis(ligand) units were identified as one of the decomposition products, their content decreased with increasing acid concentration. Neutralization of the mixture allowed regeneration of the tetrakis ions **2c** (Figures 3b and S36).

Conversely, no significant changes were observed during the ^1^H NMR titrations of **2b** and **2c** with MSA and Et_3_N, respectively (Figures S34 and S35). Moreover, **2a** turned out to be completely insensitive in both the basic and acidic environments (Figures S31 and S32).

**Figure 4:**
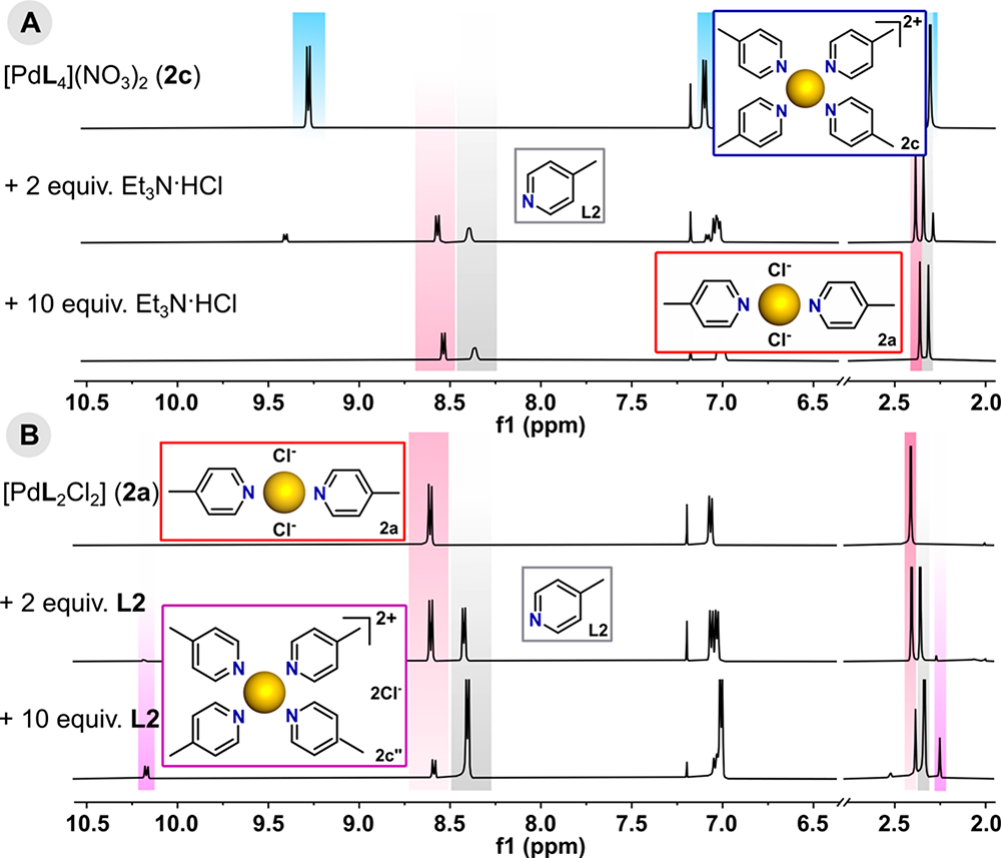
Parts of the ^1^H NMR spectra (600 MHz, CDCl_3_) showing the transformations of (a) **2c** into **2a** with Et_3_N·HCl and (b) **2a** upon the addition of **L2**.

In contrast to the series of [Pt**L**_4_]Cl_2_ units obtained by Marzilli et al.,^11^ the Pd(II) analogues have not been isolated despite many synthetic attempts. All experiments led to the formation of disubstituted species **1a**–**12a** even if a significant excess of ligand was used. To explain the distinct behavior of Pd(II) and Pt(II) complexes, we investigated the influence of Cl^–^ anions on the stability of the tetrakis(ligand) unit **2c**. During ^1^H NMR titration with triethylamine hydrochloride (Et_3_N·HCl) as the chloride source, complete disappearance of the signals from **2c** was noticed just after the addition of 2 equiv of the organic salt (Figures 4a and S29). Thus, in the presence of chloride, complete decomposition of **2c** and finally conversion to **2a** was observed along with the release of noncoordinated ligand molecules, as evidenced by the full consistency of NMR chemical shifts. Additionally, **2a** was titrated with sequential portions of ligand in an attempt to form the tetrakis species. Its structure remained initially intact, but unusually high downfield signals [Δδ(H^1^) = ∼1.8 ppm compared to that of free ligand] were found to appear with increasing ligand concentration (Figures 4b and S30). These signals could signify the replacement of chloride anions by **L2** and generation of the target [Pd**L**_4_]Cl_2_ (**2c″**), but the bis(pyridine) complex **2a** was still the dominant form even with a large excess of ligand (10 equiv). All attempts to isolate tetrakis units were unsuccessful.

### X-ray Crystallography

Slow diffusion of *n*-hexane vapor into saturated solutions of the complexes in chloroform afforded a number of crystals of Pd(II) units from three different families. Single-crystal X-ray structure determinations have been performed on 13 complexes: **2b**, **2c**, **3a**, **3b**, **4a**–**4c**, **5b**, **6a**, **6c**, and **7a**–**7c**. Separation of the trans isomers of **2b**, **3b**, **4b**, **5b**, and **7b** was achieved by selective crystallization from the mixture of geometrical isomers. All of the ORTEP representations with atom-labeling schemes are presented in Figures S40–S52. Selected geometric parameters are summarized in Table S18. These structure determinations establish the trans configuration of all of the [Pd**L**_2_Y_2_] (Y = Cl^–^ or NO_3_^–^) species and the unidentate N coordination of the pyridine ligands in all cases, basic features that, along with the bond lengths and bond angles, are important but in no way exceptional (Tables S14–S17). What a single-crystal X-ray structure determination can add to this information is a definition of the weak interactions that occur within the crystal, and one convenient method to achieve this is to consider the Hirshfeld surfaces of components involving primary bonding interactions, as defined through the use of the program *CrystalExplorer*.^18^

**Figure 5:**
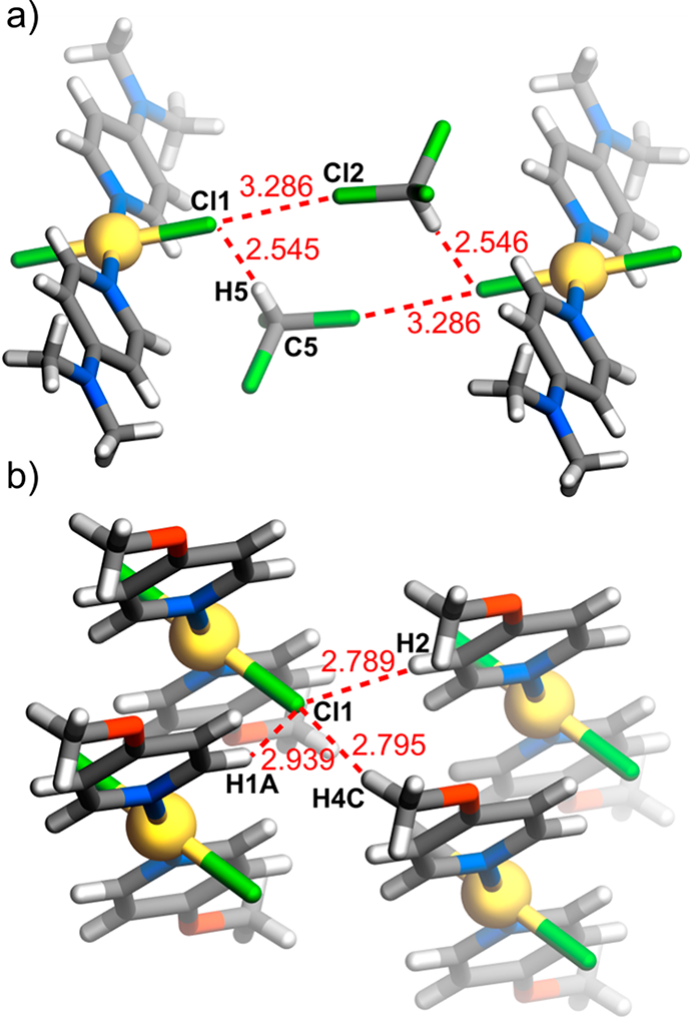
Weak interactions within the crystal structures of (a) **6a**·2CHCl_3_ and (b) **3a**.

The crystal structure of **6a**·2CHCl_3_ contains by far the most strongly basic ligand **L6** in the present series, and thus the complex provides a reference point of one extreme of the bis(ligand) species. In the crystals of metal-ion complexes of aza-aromatic ligands, it is common to find that the aza-aromatic units lie in parallel planes, forming arrays described as involving “π–π stacking”,^19^ although this may be a misleading or at least inadequate term as a description of the actual interactions occurring^20^ and they may well be only part of a panoply of weak associative effects.^21^ Indeed, the **L6** units in the crystal of **6a** do form stacks, but the Hirshfeld surface shows that the interactions involved are purely dispersive and that the only interactions that exceed dispersion are those involving the solvent molecules. These interactions involve both C–H···Cl and Cl···Cl (halogen bonding^22^) contacts and provide a model for solvation of the complex by chloroform as well as possibly explaining why the pyridine units are tilted with respect to the PdN_2_Cl_2_ plane (Figure 5a).

Passage to a complex of a much less basic ligand **L2** and the replacement of chloride by nitrate in **2b** lead to a much more complicated array of interactions exceeding dispersion. They derive exclusively, however, from the nitrate ligands and involve both O···H–C and O···C(aromatic) bonding, here perhaps indicating how an association between molecules might occur in solution, with the absence of solvent in the crystal indicating that solvation involves weaker interactions. Note that the **L2** units do lie in parallel planes but with a centroid···centroid separation of 4.78 Å and no overlap in the projection perpendicular to the planes, so that they do not constitute a “stacked” array (Figure S53).

A more direct comparison of the consequences of replacing chloride by nitrate is possible through examination of the structures of **3a** and **3b**. Ligand **L3** is again much less basic than **L6**, although slightly more basic than **L2**, and the methoxyl O is now an important point of interaction in both structures. In both, it is possible to find stacked arrays of the pyridine units, but again any interaction does not exceed dispersion in either case. In complex **3a**, where the molecular unit has 2-fold symmetry, the chloride ligands are involved in interactions exceeding dispersion with both aromatic and aliphatic H, and these are complemented by O(methoxyl)···H–C(methoxyl), O(methoxyl)···H–C(pyridine) and C(methoxyl)–H···Cl interactions (Figures 5b and S54). In the crystal of complex **3b**, the two pyridine ligands of each molecule are not equivalent and only one methoxyl group is involved in interactions exceeding dispersion. The polyatomic nature of the nitrate ligands means that they have multiple sites for interaction, but just like the chloride ligands of **3a**, they serve to link molecules through interactions with both aromatic and aliphatic HC (Figure S55).

Pyridine (**L1**) itself is, of course, the parent ligand of all of the derivatives considered here, so that the nature of its complexes provides another reference point for the present series. Its consideration at this stage is appropriate in that it is less basic than **L2**, **L3**, or **L6** but more so than any of the other ligands presently employed. Complex **1a** has particular significance in that it has been structurally characterized in three different polymorphs, space groups *C*2/*c*,^23^*P*1̅,^24^ and *P*2_1_/*n*.^25^ This polymorphism can be understood in that the Hirshfeld surfaces show dispersion interactions to be dominant, completely for the *P*1̅ polymorph and in association with limited reciprocal C–H···Cl interactions for the other two. Only in the *P*2_1_/*n* polymorph can it be said that there is an approach to a stacked array of pyridine units, but the centroid···centroid separation is 3.9159(2) Å and no indication of ring atom interactions beyond dispersion are apparent. Given the nondirectional nature of dispersion interactions, it is understandable that subtle differences in the conditions of crystallization might well give rise to the occupation of different local energy minima.

**Figure 6:**
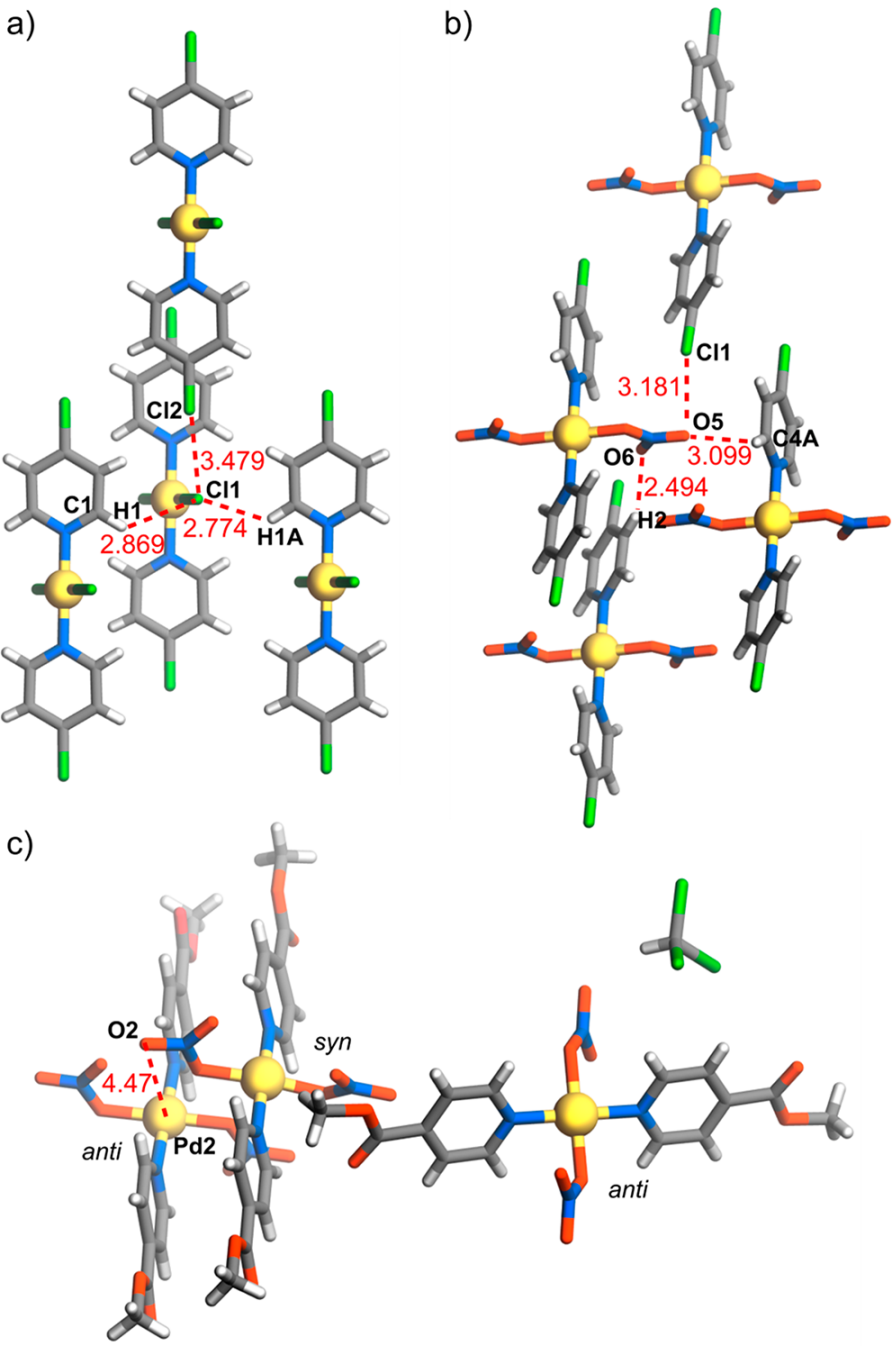
Weak interactions within the crystal structures of (a) **7a** and (b) **7b**. (c) Syn and anti orientations of nitrate ligands in the structure of **4b**.

Another direct comparison of the consequences of replacing chloride by nitrate is provided in the structures of **7a** and **7b**. In complex **7a**, each coordinated chloride has two interactions with pyridine-CH units and one barely discernible interaction with a pyridine-4-Cl unit (Figure 6a) While in complex **7b** each bound nitrate is involved in O···H–C(pyridine) interactions analogous to the Cl···H–C interactions in **7a**, one is also bound (through separate O atoms) to aromatic C and, as is clearly evident, to pyridine-4-Cl, and the other has just an additional O···Cl(pyridine) interaction (Figure 6b; in complex **7a**, the two chloride ligands are equivalent). In both complexes, these local interactions are associated with limited stacking of the pyridine units, but these involve centroid···centroid separations near 4.8 Å, with no evidence of any interaction outside dispersion. As a different polymorph (but also *P*1̅), complex **7a** has been structurally characterized previously as part of an investigation of halogen bonding within crystals of complexes of the [M(X-py)_2_(halogen)_2_] type.^26^ The Hirshfeld surface for this polymorph is very similar to that of complex **7a**, although the Cl···Cl interactions are somewhat more prominent. The centroid···centroid separation of the closest parallel pyridine ring pairs is also shorter at 3.9039(8) Å, although still with no indication of interactions exceeding dispersion.

Ligand **L4** is appreciably less basic than pyridine, implying a significant electron-withdrawing effect of the methoxycarbonyl group on the pyridine ring, but in the structure of both complexes **4a** and **4b**, there is no indication on the Hirshfeld surfaces of a change in face-to-face pyridine ring interactions from that of dispersion. Instead, as observed in the structures already described, it is the coordinated anions and pyridine substituent that are involved in all interactions that exceed dispersion. The chloride ligands of the centrosymmetric complex **4a** are involved in interactions with both pyridine and ester methyl CH atoms of adjacent molecules of the ester substituent interacting with pyridine C, again a reciprocated case (Figure S56). Complex **4b** was in fact crystallized as a hemisolvate, **4b**_2_·CHCl_3_, and the presence of three inequivalent Pd sites as well as the presence of the solvent makes a description of the weak interactions in the crystal rather complicated. What is particularly interesting here, though, is the fact that while two of the three inequivalent Pd centers can be considered to have a square-planar coordination sphere, the third (Pd1) is square-pyramidal because of axial binding to nitrate O. Axial binding of a reaction substrate can, of course, be one of the initial steps in a catalytic mechanism, and while five coordination of Pd(II) is a well-understood occurrence,^27^ it does not appear to be particularly favored in the present systems. The Pd1 environment in complex **4b** is unique in the present series in that, perhaps in order to accommodate the axial interaction, the two nitrate ligands have a syn orientation relative to the PdN_2_O_2_ plane, while in all other cases, it is anti (Figure 6c).

Ligand **L5** has Brønsted basicity very similar to that of **L4**, but the Hirshfeld surface for the centrosymmetric complex **5b** indicates that the acetyl substituent produces more significant charge relocalization in the pyridine ring than does the methyl ester group. Thus, nitrate O is involved in interactions not only with both aromatic and aliphatic H, as in complexes **2b**, **3b**, **4b**, and **7b**, but also with the carbonyl C of the substituent and the pyridine C adjacent to it (Figure S57). Unlike complex **4b**, complex **5b** shows no evidence of an axial interaction with Pd exceeding dispersion, but as for Pd2 and Pd3 in complex **4b**, two O atoms are located, here, 3.776(6) Å above and below the PdN_2_O_2_ plane in a line with the Pd, indicating again that an axial approach could be a minimum energy pathway to binding an extra ligand. (In complex **4b**, the O atoms are 3.141(3) Å from Pd2 and 3.252(3) Å from Pd3.)

**Figure 7:**
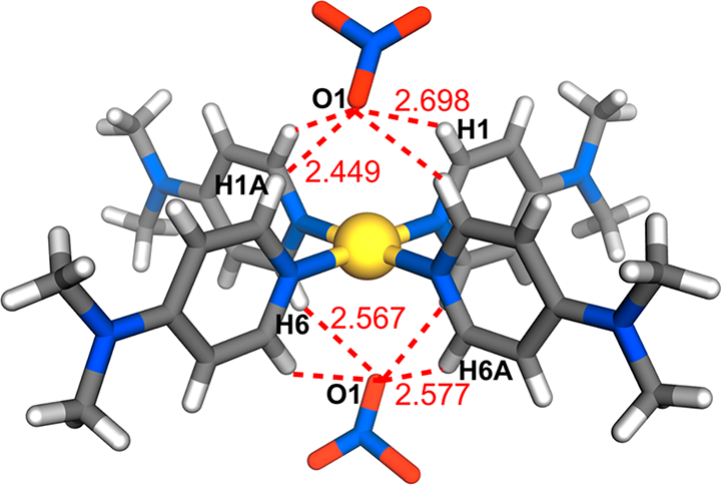
Nonbonding C–H···O interactions between [Pd**L**_4_]^2+^ and nitrate counterions in the structure of complex **6c**.

The application of Hirshfeld surface analysis to complexes **2c**, **4c**, **6c**, and **7c** is limited by the disorder present in the structures of **4c** and **7c**, so that a detailed analysis has been applied to the structures of complexes **2c** and **6c** only. All four complexes do, however, have a structure in which all four pyridine units lie close to perpendicular to the PdN_4_ plane, a feature well-known in various tetrakis(pyridine) complexes and commonly ascribed to its enabling of the minimization of repulsion between the ligands,^28^ with such a repulsion also being considered the reason for the difficulty in obtaining hexakis(pyridine) complexes of octahedral metal ions.^29^ For [Pt**L**_4_]^2+^ cations, where the same conformation is observed, an alternative explanation based on the observation of specific interactions of cations with counteranions has, however, been offered.^11^ In the structure of complex **6c**, it is possible to discern a degree of interlocking of the cations with a resemblance to what is found in instances of the “terpyridine embrace”,^19b^ but as is seen in the bis(ligand) species **6a**, the Hirshfeld surface provides no evidence for interactions exceeding dispersion between **L6** units. What is evident on the Hirshfeld surface is the versatility of nitrate in forming O···H–C bonds involving both aromatic and aliphatic H atoms. One result of this is that nitrate anions do form “caps” to each cation, as seen with the Pt(II) analogues,^11^ by the interaction of O1 with three aromatic CH atoms (H1A, H6, and H6A) of adjacent ligands (Figure 7). The additional interactions of O2 with methyl CH (H9B and H9AB) and aromatic CH (H2) atoms serve to link cations into sheets parallel to (010) from which **L6** units project so that the sheets are linked through dispersion interactions. In the structure of complex **2c**, there is disorder of the anions, which complicates the interpretation of their interactions, but the Hirshfeld surface of the cations shows that the chains of cations running along [001] are, in fact, linked by C(aromatic)–H···C(aromatic) interactions, providing an example of where changing the substituent on pyridine results in the generation of pyridine···pyridine interactions exceeding dispersion, a feature not apparent in any of the other comparisons of the present work. Regardless of their disorder, the nitrate anions do appear to occupy capping regions of the cations, as seen in complex **6c**, and this is true also for the nondisordered anions associated with the disordered cations in complexes **4c** and **7c**.

What is observed in the solid state through crystal structure determinations does not necessarily apply to solutions, but the rarity of solvent incorporation in the structures presently described indicates that solvation interactions can be in competition with a variety of other forces determined by the particular nature of the solute. What has not been overtly considered in the discussion above of the tetrakis(pyridine) complexes is the fact that they are considered to be ionic species and thus that there should be an electrostatic factor to be allowed for in the cation···anion interactions. The calculation of Hirshfeld surfaces with neutral-atom wave functions may therefore be misleading in regard to the intensity of interactions but not their directionality, so that the cation capping by nitrate seen in the structures of complexes **2c**, **4c**, **6c**, and **7c** can still be seen as a consequence of O···H–C interactions. As argued in the case of Pt(II) analogues,^11^ the preservation of such interactions in solution could explain why strong downfield shifts are also observed in the ^1^H NMR spectra of complexes **2c**, **4c**, **6c**, and **7c**, although it is also important to note that the environment of the pyridine protons in the bis(pyridine) complexes is quite varied and quite different from that in the tetrakis species. In regard to catalysis by [Pd**L**_*n*_Y_*m*_], the interactions of different substituents and counteranions indicate possible structural features of a substrate that might enhance its binding to the catalyst, but this, of course, is one step in what must be a more complicated process.

### Catalytic Studies

Because of the structural differences between Pd(II) complexes with pyridine ligands, catalytic studies were undertaken in order to investigate their activity in Pd-catalyzed cross-coupling reactions and explore their diversity in functionality as well. Thus, their catalytic properties were tested and compared in both the Suzuki–Miyaura and Heck reactions.

### Suzuki–Miyaura Coupling

Complex **2c** was selected as a model catalyst precursor for which the reaction conditions were optimized in the coupling between 4′-bromoacetophenone and phenylboronic acid (Table S22). Among the tested bases (K_2_CO_3_, K_3_PO_4_, NaOH, and Et_3_N) and solvents (chloroform, toluene, 1,4-dioxane, and *N*,*N*-dimethylformamide), the combination of K_3_PO_4_ and toluene allowed formation of the expected 4-acetylbiphenyl in the highest gas chromatography (GC) yield. The catalytic reactions were performed at 80 °C and, importantly, without the need to exclude air or water. Taking economic and environmental considerations into account, the optimal catalyst concentration was 0.1 mol %, which resulted in an almost quantitative conversion just after 2 h.

Subsequently, the catalytic activity of the full range of structurally diversified Pd(II) complexes with pyridine ligands was tested under the same optimized reaction conditions. The majority of the catalyst precursors provided the cross-coupling product in excellent yields of >90% (Table 2). Only minor differences were observed between bis and tetrakis complexes of a given ligand, so it appears that the nature of the complex and the different counterions does not directly influence the effectiveness of the catalyzed reaction. Nevertheless, some differences could be noted depending on the ring substituent. The lowest GC yields were observed for the complexes based on **L4** (64–78%). Better GC yields (>70%) were achieved for the complexes based on **L5**, **L7**–**L9**, and **L11**, while those of **L1**–**L3**, **L6**, and **L10** showed the highest activity in Suzuki–Miyaura coupling. Although no simple correlation was observed between GC yields and p*K*_a_ values of the ligands (Figure S93), Pd(II) complexes with more basic pyridine ligands generally showed slightly greater catalytic effectiveness.

**Table 2:** GC Yields [%]^a^ in Suzuki–Miyaura and Heck Cross-Coupling Reactions Catalyzed by Pd(II) Complexes Based on Ligands **L1**–**L12**

		GC yield [%] in Suzuki–Miyaura coupling^b^			GC yield [%] in Heck coupling^c^		
	p*K*_a_ of **L**	Pd**L**_2_Cl_2_ (**1a**–**12a**)	Pd**L**_2_(NO_3_)_2_ (**1b**–**12b**)	Pd**L**_4_(NO_3_)_2_ (**1c**–**12c**)	Pd**L**_2_Cl_2_ (**1a**–**12a**)	Pd**L**_2_(NO_3_)_2_ (**1b**–**12b**)	Pd**L**_4_(NO_3_)_2_ (**1c**–**12c**)
**L1**	5.23	97	93	95	85	88	90
**L2**	5.98	93	92	98	90	91	94
**L3**	6.47	93	91	91	86	82	76
**L4**	3.49	78	72	64	89	92	79
**L5**	3.57	86	87	88	80	92	75
**L6**	9.61	93		90	86		83
**L7**	3.83	82	74	75	90	92	80
**L8**	2.10	88	66		91	93	
**L9**	2.46	87	70		81	91	
**L10**	3.07	98		90	93		88
**L11**	3.12	86		79	88		90
**L12**	2.86	83		92	92		77

a Reaction yields were determined by GC–MS measurement of 4′-bromoacetophenone or iodobenzene decay as the average of three results.

b Reaction conditions: 4′-bromoacetophenone (0.2 mmol, 1 equiv), phenylboronic acid (0.24 mmol, 1.2 equiv), K_3_PO_4_ (0.4 mmol, 2 equiv), and Pd(II) complex (0.1 mol %) were stirred in toluene (2 mL) at 80 °C for 2 h.

c Reaction conditions: iodobenzene (0.2 mmol, 1 equiv), styrene (0.24 mmol, 1.2 equiv), Et_3_N (1.0 mmol, 5 equiv), and Pd(II) complex (0.1 mol %) were stirred in DMSO (2 mL) at 120 °C for 2 h.

**Scheme 2:**
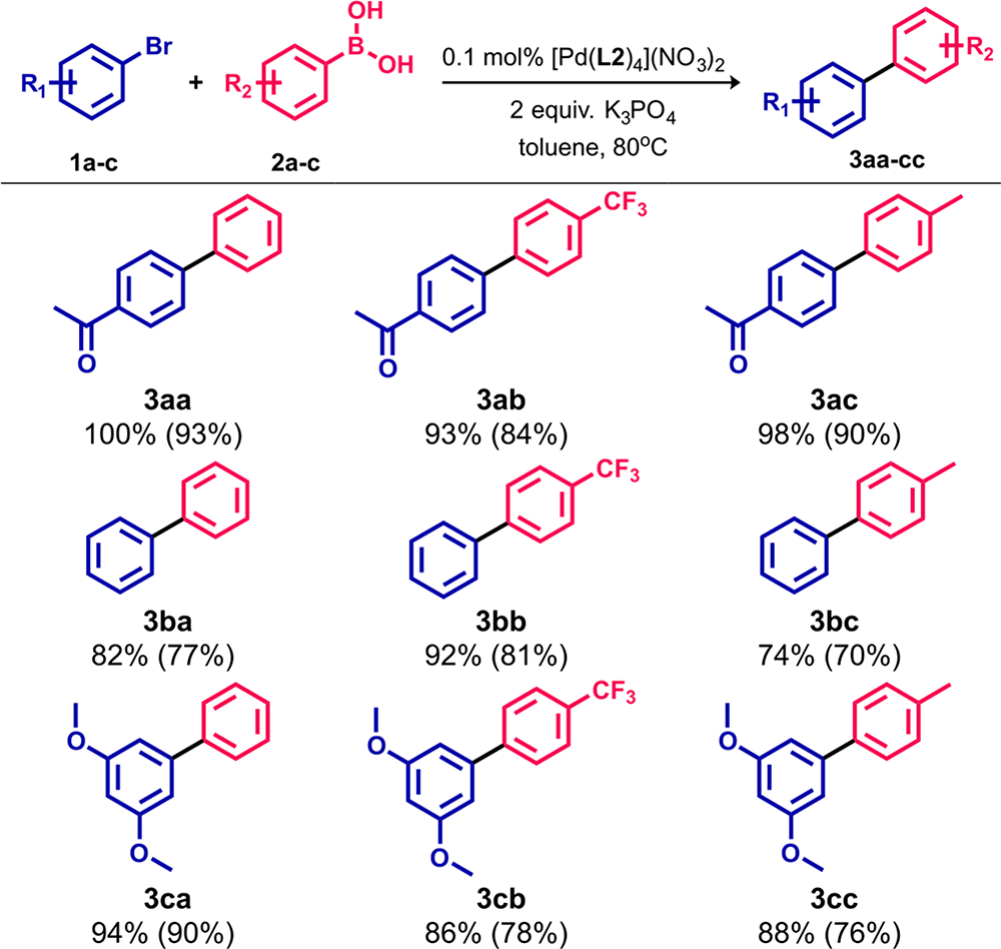
Scope of the Suzuki–Miyaura Cross-Coupling Reaction between Aryl Bromides and Arylboronic Acids The GC yields were determined by GC–MS measurement of aryl bromide decay. The yields in parentheses are for the isolated compounds.

Complex **2c**, as one of the most effective systems, was selected to explore the capabilities in the Suzuki–Miyaura cross-coupling in terms of functional-group tolerance. Under the optimized reaction conditions, a set of functionalized aryl bromides and arylboronic acids were reacted together. The **2c** unit enabled the synthesis of scope of structurally distinct biphenyl derivatives **3aa**–**3cc** in high-to-excellent yields (74–100%; Scheme 2). The high efficiency was observed regardless of the presence of electron-donating (−Me and −OMe) or electron-withdrawing (−CF_3_ and −COMe) substituents in the substrate molecules, highlighting the catalyst precursor versatility.

### Heck Coupling

For an initial assessment of the efficacy of the complexes as catalyst precursors for the Heck reaction, the cross-coupling of iodobenzene with styrene catalyzed by **2c** was chosen as a model reaction to develop the reaction conditions (Table S24). Under the conditions optimized for the Suzuki–Miyaura reaction, only traces of the Heck coupling product were observed. For this reason, different variations in terms of solvents and bases were tested using a 1 mol % Pd(II) complex. The reaction did not proceed successfully in the presence of inorganic bases (K_3_PO_4_ and K_2_CO_3_) or nonpolar solvent (toluene). The pair of Et_3_N and DMSO represented the best combination to reach high yields because almost quantitative conversion was achieved at 120 °C just after 2 h. Additional experiments showed that the catalyst loading could be reduced to 0.1 mol %. This concentration was sufficient to guarantee good conversion at the same time, whereas using 0.01 mol % significantly extended the reaction time. With these results in hand, subsequent catalytic reactions were performed in DMSO at 120 °C using Et_3_N as a base and 0.1 mol % Pd(II) complex. Note that the Pd(II) complexes essentially retain their structure under the reaction conditions, as indicated by the ^1^H NMR spectra recorded after heating in DMSO at 120 °C (Figures S37–S39).

A comparison of the catalytic activities for a number of the other Pd(II) complexes was performed for the Heck reaction as well. As with the Suzuki–Miyaura cross-coupling, potential catalyst precursors were examined to evaluate the substituent effect on the efficiency in catalyzed reactions. Under the same conditions, very high GC yields (>90%) were obtained in most of the reactions, and yields of <80% were observed in only a few cases (Table 2). Overall, the tetrakis(pyridine) complexes, especially with ligands **L3**–**L5** and **L12**, provided lower GC yields (75–79%) in comparison to neutral bis(ligand) species. Any ring substituent effect was negligible, and no clear relationship between the ligand basicity and catalytic activity of the Pd(II) complexes was apparent (Figure S95). In all cases, the selectivity in the (*E*)-stilbene formation was very high, ranging from 89% to 99%, and was completely independent of the catalyst precursor structure.

**Scheme 3:**
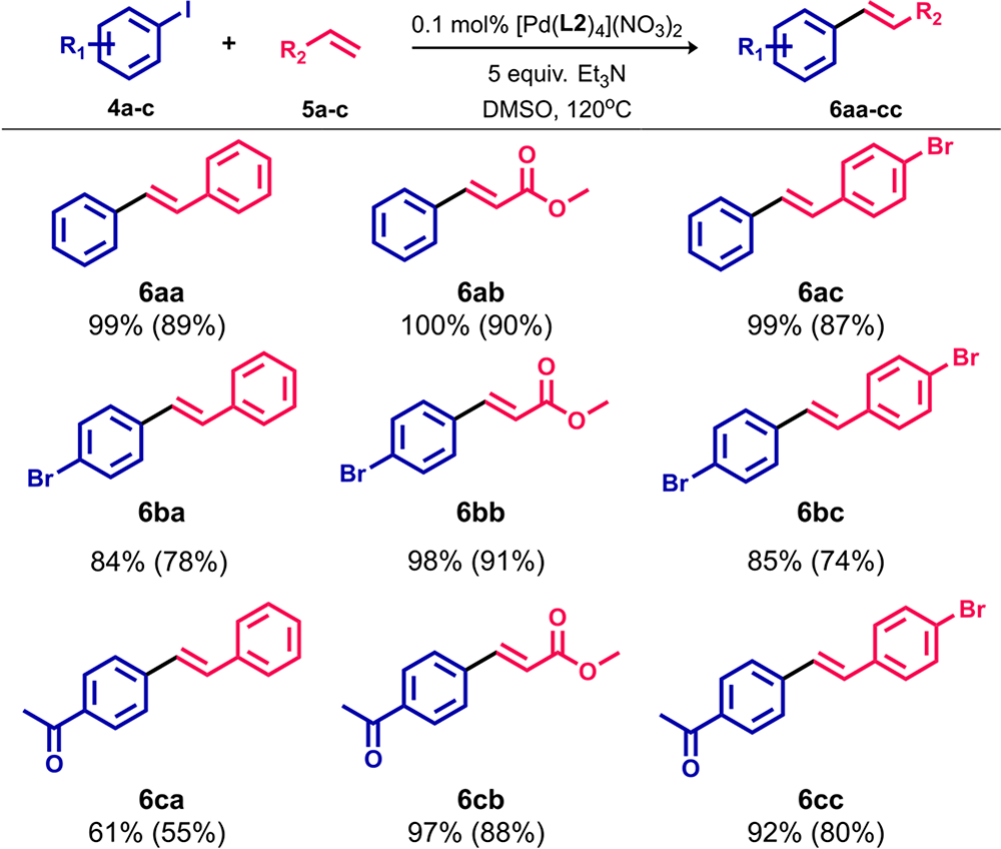
Scope of the Heck Cross-Coupling Reaction between Aryl Iodides and Olefins The GC yields were determined by GC–MS measurement of aryl iodide decay. The yields in parentheses are for the isolated compounds.

To investigate the scope of the Heck cross-coupling reaction, the catalytic properties of complex **2c** were further studied by using a set of functionalized substrates under the conditions described above. As shown in Scheme 3, **2c** showed good catalytic activity and selectivity in the reactions between aryl iodides and olefins, giving GC yields in the range of 61–100%. It is noteworthy that an excellent conversion was accomplished for acrylate derivatives (97–100%). The reaction system exhibited also great chemoselectivity toward iodoarenes because no cross-coupling involving bromoarene moieties, as either olefin or haloarene coupling partners, was observed.

Complex **2c** as a representative of the multiple family of Pd(II) complexes with pyridyl ligands has been extensively investigated with respect to catalytic properties that demonstrated high catalytic activity in the Suzuki–Miyaura and Heck cross-coupling reactions. On the basis of the experiments carried out, it can be concluded that all of the units presented herein constitute a group of versatile precatalysts that can be successfully applied in Pd-catalyzed reactions.

Because of the multitude of literature reports on the mechanism of both Suzuki–Miyaura and Heck cross-coupling, profound studies have not been conducted in this area. We assume that the bis- and tetrakis(pyridine) complexes considered in this paper play the precatalyst role. According to the generally accepted mechanism, the reduction of Pd(II) to Pd(0) occurs at the beginning of the catalytic cycle, leading to the generation of active species. The process then proceeds in a typical manner for Pd-catalyzed transformations, through the sequence of three consecutive stages involving oxidative addition, transmetalation or carbometalation, and reductive elimination, as described in numerous works.^30^ The precatalyst was degraded during the cycle that was observed as precipitation of metallic Pd; therefore, it could not be regenerated and then reused.

## Conclusions

In summary, a series of Pd(II) complexes based on a wide range of functionalized pyridine derivatives have been successfully generated and analyzed in solution via NMR spectroscopy and MS as well as in the solid state via X-ray diffraction. This work has been based on two sets of complexes of the general formulas [Pd**L**_4_](NO_3_)_2_ and [Pd**L**_2_Y_2_], where Y = Cl^–^ or NO_3_^–^. Their properties have been examined in light of the ligand basicity as a factor of influence, although the results obtained have shown that this is just one of several factors that may be important. The complexes have been found to be of practical utility as simple and efficient catalyst precursors for both the Suzuki–Miyaura and Heck cross-coupling reactions for a scope of substrates under relatively mild conditions.

## Experimental Section

### General Procedures

All reagents were purchased from commercial suppliers (mainly Merck or Fluorochem) and used without further purification. High-purity solvents were purchased from VWR. NMR solvents were purchased from Deutero GmbH (Germany) and used as received. NMR spectra were acquired on Bruker Fourier 300 MHz, Bruker Avance IIIHD 400 MHz, and Bruker Avance IIIHD 600 MHz spectrometers at 25 °C and referenced to a tetramethylsilane signal or solvent residual peaks. All NMR data were processed with Mestrelab Research *MNova* software. ESI-MS spectra were recorded on Bruker HD Impact and ABSciex QTOF 5600 spectrometers in positive-ion mode. Theoretical MS spectra were predicted using Mestrelab Research *MNova* software. GC–MS analyses were performed on a Bruker 450-GC spectrometer with a 30 m Varian DB-5 0.25 mm capillary column and a Scion SQ-MS detector.

### X-ray Crystallography

X-ray measurements were performed using an Oxford Diffraction SuperNova diffractometer with monochromatic Cu Kα radiation for **2c**, **3a**, **5b**, and **6a**. The diffraction data were collected on a Rigaku XtaLAB Synergy diffractometer equipped with a rotating anode as a Cu Kα radiation source for **4a**. The remaining compounds were subjected to X-ray measurements on an Oxford Diffraction Xcalibur diffractometer with Mo Kα radiation. Data collection and data reduction for all Pd(II) complexes were carried out using the *CrysAlisPRO* software.^31^ Using *OLEX2*, the intrinsic phasing method (*ShelXT*) was used for crystal structure solution.^32^ The exception is the **4a** structure, which was solved with direct methods (*ShelXS*).^33^ The refinement process was performed with anisotropic displacement parameters for non-H atoms with the full-matrix least-squares method based on *F*^2^ (*ShelXL*).^32b^ In all structures, except for **7a** and **7b**, the H atoms were placed in calculated positions and refined using a riding model. The high quality of the obtained single crystals of complexes **7a** and **7b** made it possible to carry out high-resolution X-ray measurements. Therefore, the H atoms have been derived from the difference Fourier map and refined without constraints. Crystallographic data, details on the refinement, twin structures, and disordered fragments in the crystal structures are included in the SI.

### Synthesis of Ligands

Ligands **L1**–**L9** were purchased from commercial suppliers and used as received. Ligands **L10**–**L12** were prepared according to the previously described procedures.^7e,34^

### Synthesis of [Pd**L**_2_Cl_2_] Complexes (**1a**–**12a**)

One of the ligands **L1**–**L12** (∼0.2 mmol, 2 equiv) was added to an acetonitrile (MeCN) solution of PdCl_2_ (∼0.1 mmol, 1 equiv in 5 mL of MeCN). Then the resulting mixture was heated under reflux for 12 h. The precipitate that formed was centrifuged off, washed with MeCN (10 mL) and diethyl ether (Et_2_O; 2 × 10 mL), and dried under vacuum. Specific details on the synthetic procedures and analytical data (quantities used, yields, NMR and MS data, etc.) can be found in the SI.

### Synthesis of [Pd**L**_2_(NO_3_)_2_] Complexes (**1b**–**12b**)

One of the ligands **L1**–**L12** (0.2 mmol, 2 equiv) was added to an MeCN solution of Pd(NO_3_)_2_·2H_2_O (0.1 mmol, 1 equiv in 5 mL of MeCN). Then, the resulting mixture was heated under reflux for 12 h. The solvent was then evaporated under reduced pressure. The crude product was redissolved in MeCN (1 mL) and reprecipitated by the addition of Et_2_O (10 mL). The precipitate was centrifuged off, washed with Et_2_O (2 × 10 mL), and dried under a vacuum. Specific details on the synthetic procedures and analytical data (quantities used, yields, NMR and MS data, etc.) can be found in the SI.

### Synthesis of [PdL_4_](NO_3_)_2_ Complexes (**1c**–**12c**)

To a suspension of PdCl_2_ or Pd(DMSO)_2_Cl_2_ (∼0.1 mmol, 1 equiv) in ethanol (5 mL) was added a solution of one of the ligands **L1**–**L12** (∼1.0 mmol, 10 equiv) in dichloromethane (DCM; 5 mL), and the resulting mixture was stirred at room temperature for 1 h. Then, AgNO_3_ (∼0.2 mmol, 2 equiv) in 0.5 mL of H_2_O was added, and the resulting suspension was stirred for an additional 12 h excluding light. The reaction mixture was filtered to remove AgCl, and then the filtrate was evaporated under reduced pressure. The crude product was redissolved in DCM (1 mL) and reprecipitated by the addition of *n*-hexane (10 mL). The precipitate was centrifuged off, washed with *n*-hexane (2 × 10 mL), and dried under a vacuum. Specific details on the synthetic procedures and analytical data (quantities used, yields, NMR and MS data, etc.) can be found in the SI.

### Suzuki–Miyaura Coupling

A reaction vessel equipped with a stirring bar was charged with aryl bromide (1.0 mmol, 1.0 equiv) and arylboronic acid (1.2 mmol, 1.2 equiv) dissolved in toluene (10 mL). Then, the Pd(II) precatalyst (0.001 mmol, 0.001 equiv) as a solution in chloroform (0.05 mL) and solid K_3_PO_4_ (2.0 mmol, 2.0 equiv) was added. The vial was sealed, and the reaction mixture was heated for 2 h at 80 °C. Then, the resulting solution was cooled to room temperature, diluted with DCM (50 mL), and washed with distilled water (40 mL). The collected aqueous phase was extracted with DCM (2 × 50 mL). The organic layers were gathered, dried over Na_2_SO_4_, and filtered, and the solvent was removed under reduced pressure. The residue was purified by column chromatography on silica gel to obtain the desired products **3aa**–**3cc**. The full characterization of the coupling products is available in the SI.

### Heck Reaction

A reaction vessel equipped with a stirring bar was charged with aryl iodide (1.0 mmol, 1.0 equiv) and olefin (1.2 mmol, 1.2 equiv) dissolved in DMSO (10 mL). Then, the Pd(II) precatalyst (0.001 mmol, 0.001 equiv) as a solution in DMSO (0.05 mL) and Et_3_N (5.0 mmol, 5.0 equiv) was added. The vial was sealed, and the reaction mixture was heated for 2 h at 120 °C. Then, the resulting solution was cooled to room temperature, diluted with ethyl acetate (50 mL), and washed with icy distilled water (40 mL). The collected aqueous phase was extracted with ethyl acetate (2 × 50 mL). The organic layers were gathered, dried over Na_2_SO_4_, and filtered, and the solvent was removed under reduced pressure. The residue was purified by column chromatography on silica gel to obtain the desired products **6aa**–**6cc**. The full characterization of the coupling products is available in the SI.

## Supporting Information Available

Additional experimental details, materials and methods, NMR and ESI-MS spectra for all compounds, NMR titration experiments, crystal data and structure refinement for Pd(II) complexes, reaction development for catalytic tests, and characterization of cross-coupling products (PDF)
