# Supplementary material for: Pd(II) Complexes with Pyridine Ligands: Substituent Effects on the NMR Data, Crystal Structures, and Catalytic Activity
Source: Inorg Chem. 2022 Aug 19;61(35):14019–29. doi: 10.1021/acs.inorgchem.2c01996 (PMC9455277; doi:10.1021/acs.inorgchem.2c01996)
Supplement: Supplementary file 1 — ic2c01996_si_001.pdf [file ic2c01996_si_001.pdf]

Supporting Information for

Pd(II) complexes with pyridine ligands: substituent effects on NMR data, crystal structures and catalytic activity

Gracjan Kurpik,[a, b] Anna Walczak,[a, b] Mateusz Gołdyn,[a] Jack Harrowfield,[c] and Artur R. Stefankiewicz[a, b]*

[a] *Faculty of Chemistry, Adam Mickiewicz University, Uniwersytetu Poznańskiego 8, 61-614 Poznań, Poland*

[b] *Center for Advanced Technology, Uniwersytetu Poznańskiego 10, 61-614 Poznań, Poland*

[c] *ISIS, Université de Strasbourg, 8 allée Gaspard Monge, 67083 Strasbourg, France*

Table of contents

1. Synthesis of ligands S3
2. Synthetic methods for Pd(II) complexes S5
3. Characterization of Pd(II) complexes S6
3.1. Pd(II) complexes based on pyridine (L1) S6
3.2. Pd(II) complexes based on 4-methylpyridine (L2) S8
3.3. Pd(II) complexes based on 4-methoxypyridine (L3) S10
3.4. Pd(II) complexes based on methyl isonicotinate (L4) S12
3.5. Pd(II) complexes based on methyl 4-acetylpyridine (L5) S13
3.6. Pd(II) complexes based on 4-(dimethylaminio)pyridine (L6) S15
3.7. Pd(II) complexes based on 4-chloropyridine (L7) S16
3.8. Pd(II) complexes based on 4-pyridinecarbonitrile (L8) S18
3.9. Pd(II) complexes based on 4-(trifluoromethyl)pyridine (L9) S20
3.10. Pd(II) complexes based on N-phenyl-1-(pyridin-4-yl)methanimine (L10) S21
3.11. Pd(II) complexes based on *N*-phenylisonicotinamide (L11) S22
3.12. Pd(II) complexes based on 4,4-dimethyl-1-(pyridin-4-yl)pentane-1,3-dione (L12). S24
4. ^{1}H NMR analysis of Pd(II) complexes based on pyridine ligands S26
4.1. The relationship between chemical shifts in the ^{1}H NMR spectra and basicity of free ligands S27
4.2. The relationship between chemical shift changes in the ^{1}H NMR spectra and basicity of free ligands S27
5. ^{1}H NMR titration of $[Pd(L2)_4](NO_3)_2$ with $Et_3N \cdot HCl$ S28
6. ^{1}H NMR titration of $[Pd(L2)_2Cl_2]$ with L2 .... S28
7. Acid – base titrations of the Pd(II) complexes based on the ligand L2 .... S29

7.1. ^{1}H NMR titration of $[Pd(L2)_2Cl_2]$ with Et_3N S29
7.2. ^{1}H NMR titration of $[Pd(L2)_2Cl_2]$ with MSA S29
7.3. ^{1}H NMR titration of $[Pd(L2)_2(NO_3)_2]$ with Et_3N S30
7.4. ^{1}H NMR titration of $[Pd(L2)_2(NO_3)_2]$ with MSA S30
7.5. ^{1}H NMR titration of $[Pd(L2)_4](NO_3)_2$ with Et_3N S31
7.6. ^{1}H NMR titration of $[Pd(L2)_4](NO_3)_2$ with MSA S31
8. Stability investigation of Pd(II) complexes at high temperature in DMSO S32
9. X-ray crystal structure analysis S33
9.1. Additional details for crystal structure solution and refinement S33
9.2. Description of the X-ray structure of Pd(II) complexes S34
9.3. ORTEP representations of Pd(II) complexes S37
10. Comparison of selected parameters for crystals of Pd(II) complexes S47
11. Investigation of catalytic activity in the Suzuki-Miyaura cross-coupling S52
11.1. Reaction development for the Suzuki-Miyaura cross-coupling S52
11.2. Catalytic activity of Pd(II) complexes based on pyridine ligands in the Suzuki-Miyaura reaction S53
11.3. General synthetic procedure for the Suzuki-Miyaura cross-coupling S53
11.4. Characterization of the cross-coupling products S53
11.5. NMR spectra of the cross-coupling products S56
12. Investigation of catalytic activity in the Suzuki-Miyaura cross-coupling S65
12.1. Reaction development for the Heck reaction S65
12.2. Catalytic activity of Pd(II) complexes based on pyridine ligands in the Heck reaction S65
12.3. General synthetic procedure for the Heck cross-coupling S66
12.4. Characterization of the cross-coupling products S66
12.5. NMR spectra of the cross-coupling products S69
13. Influence of the type of pyridine ligands and the nature of Pd(II) complexes on GC yields in catalyzed reactions S77
13.1. The relationship between GC yield and the character of Pd(II) complex in the Suzuki-Miyaura cross-coupling S77
13.2. The relationship between GC yield and the ligand basicity in the Suzuki-Miyaura cross-coupling S77
13.3. The relationship between GC yield and the character of Pd(II) complex in the Heck cross-coupling S78
13.4. The relationship between GC yield and the ligand basicity in the Heck cross-coupling S78
14. References S78

1. Synthesis of ligands

1.1. N-phenyl-1-(pyridin-4-yl)methanimine (L10)

The ligand **L10** was prepared according to a literature procedure.[1] The reaction of 4-pyridinecarboxaldehyde (1.95 mL, 20.73 mmol) with aniline (1.89 mL, 20.73 mmol) gave **L10** as a white solid. Yield: 85%, 3.21 g.

^{1}H NMR (300 MHz, $CDCl_3$) δ = 8.77 (d, *J* = 6.0 Hz, 2H, H^1), 8.46 (s, 1H, H^3), 7.76 (d, *J* = 6.0 Hz, 2H, H^2), 7.43 (t, *J* = 7.6 Hz, 2H, H^5), 7.33 – 7.22 (m, 3H, $H^{4,6}$).

^{1}H NMR results are in a good accordance with data in the literature.[2]

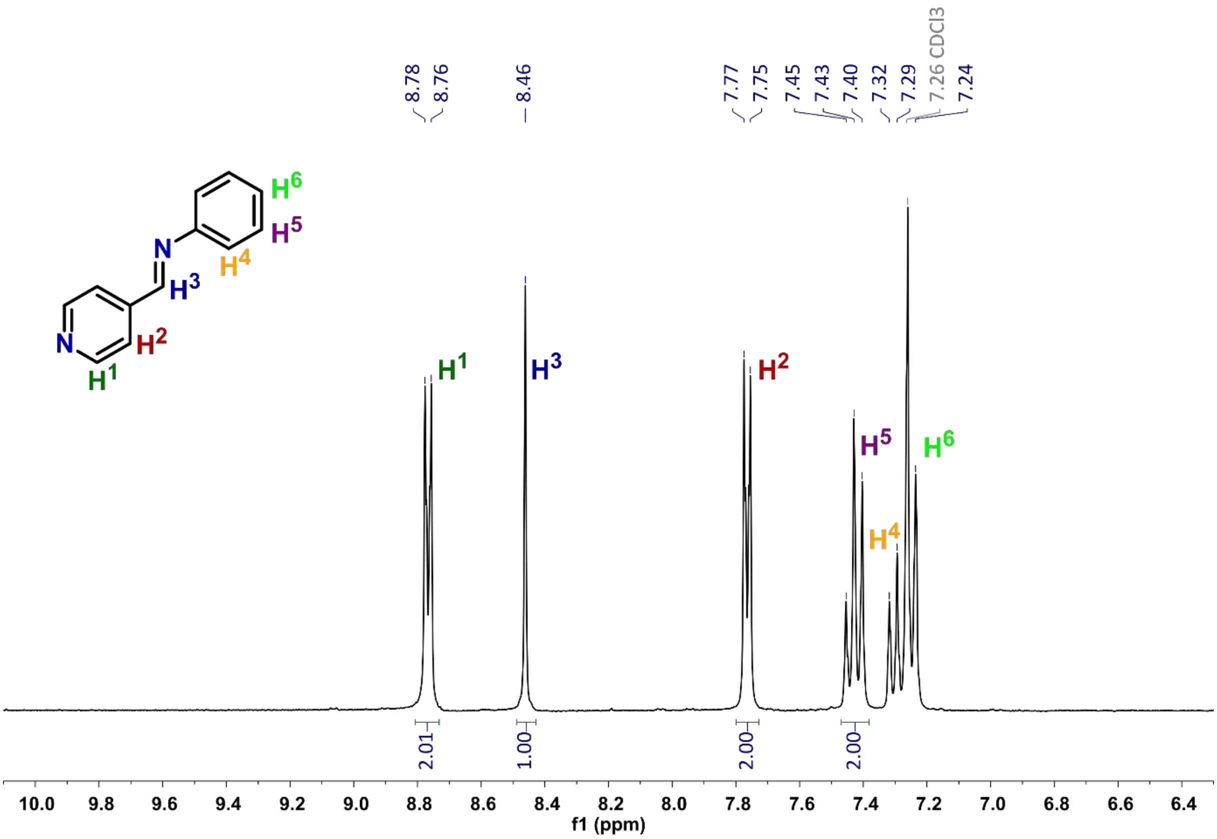

Figure S1. ^{1}H NMR spectrum (300 MHz, $CDCl_3$) of N-phenyl-1-(pyridin-4-yl)methanimine (**L10**).

1.2. *N*-phenylisonicotinamide (L11)

The ligand **L11** was prepared according to a literature procedure.[3] The reaction of isonicotinic acid (2.00 g, 16.24 mmol) with thionyl chloride (15.00 mL), and next aniline (1.48 mL, 16.24 mmol) gave **L11** as a light yellow solid. Yield: 97%, 3.11 g.

^{1}H NMR (300 MHz, $CDCl_3$) δ = 8.78 (d, *J* = 6.1 Hz, 2H, H^1), 8.01 (s, 1H, H^6), 7.70 (d, *J* = 6.1 Hz, 2H, H^2), 7.64 (d, *J* = 7.6 Hz, 2H, H^3), 7.39 (t, *J* = 7.7 Hz, 2H, H^4), 7.20 (t, J = 7.4 Hz, 1H, H^5).

^{1}H NMR results are in a good accordance with data in the literature.[4]

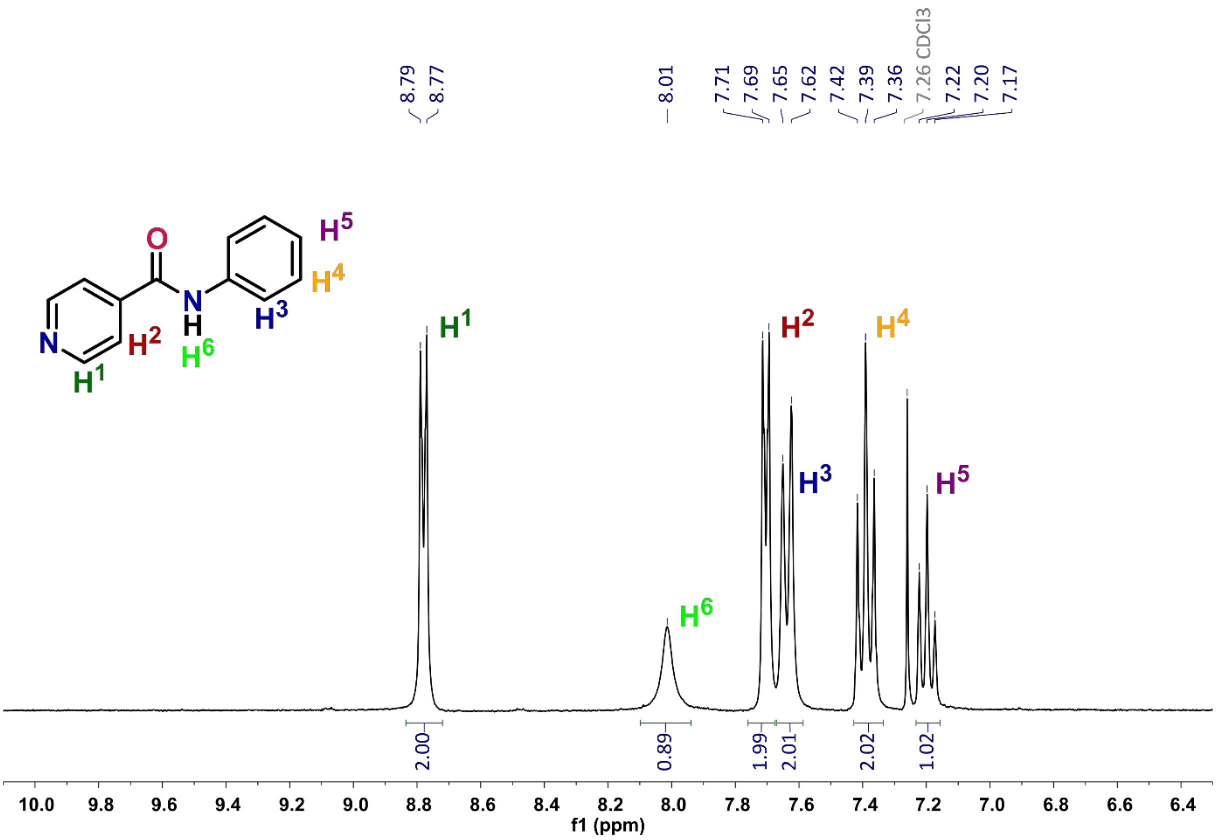

Figure S2. ^{1}H NMR spectrum (300 MHz, $CDCl_3$) of *N*-phenylisonicotinamide (**L11**).

1.3. 4,4-dimethyl-1-(pyridin-4-yl)pentane-1,3-dione (L12)

The ligand **L12** was prepared according to a literature procedure.[5] The reaction of methyl isonicotinate (5.00 g, 36.5 mmol) with pinacolone (6.41 mL, 51.1 mmol) gave **L12** as a brown oil. Yield: 78%, 5.82 g.

^{1}H NMR (300 MHz, $CDCl_3$) δ = 16.09 (s, 1H, H^5), 8.75 (dd, *J* = 6.5, 2.6 Hz, 2H, H^1), 7.69 (dd, *J* = 6.5, 2.8 Hz, 2H, H^2), 6.34 (s, 1H, H^3), 1.26 (s, 9H, H^4).

^{1}H NMR results are in a good accordance with data in the literature.[5]

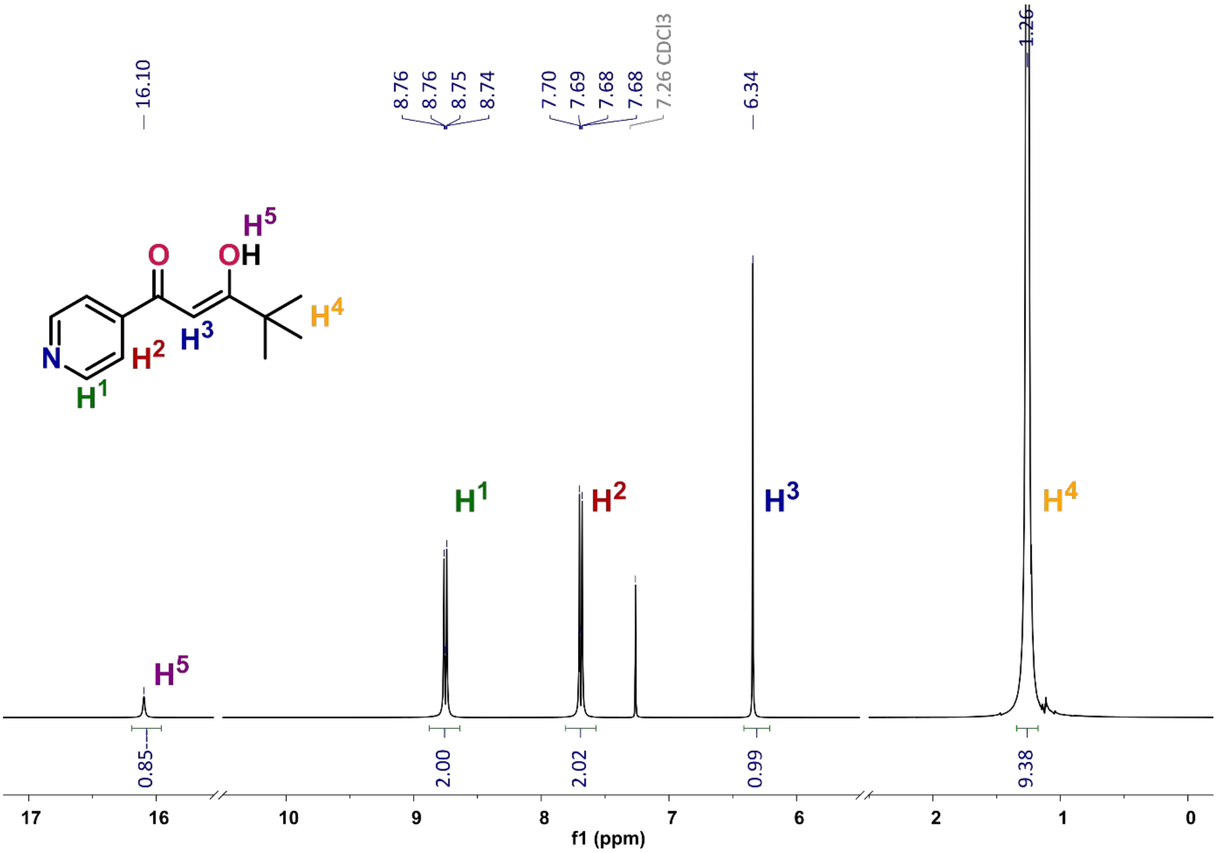

Figure S3. ^{1}H NMR spectrum (300 MHz, $CDCl_3$) of 4,4-dimethyl-1-(pyridin-4-yl)pentane-1,3-dione (**L12**).

2. Synthetic methods for Pd(II) complexes

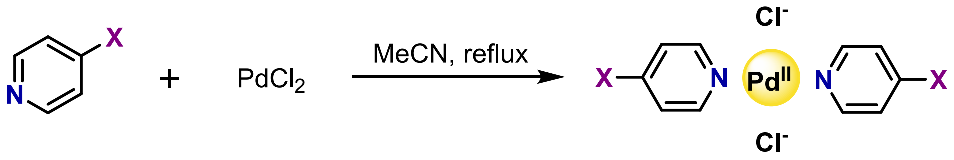

Scheme S1. Reaction scheme for the preparation of [Pd**L**$_2$Cl$_2$].

Method I. Ligand **L1 – L12** (~0.2 mmol, 2 equiv.) was added to an acetonitrile solution of $PdCl_2$ (~0.1 mmol, 1 equiv. in 5 mL MeCN). After, the resulting mixture was heated under reflux for 12 h. The precipitate that formed was centrifuged off, washed with MeCN (10 mL) and Et_2O (2 x 10 mL), and dried under vacuum.

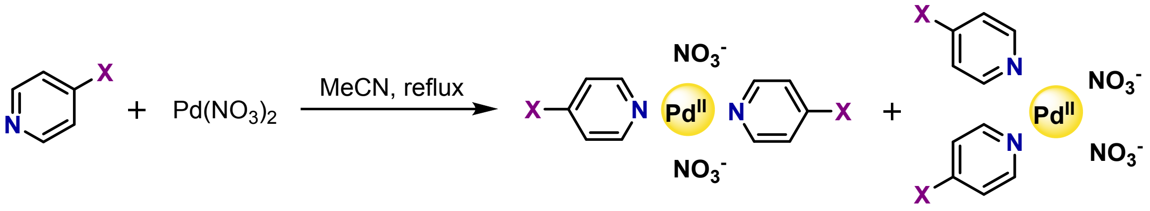

Scheme S2. Reaction scheme for the preparation of [Pd**L**$_2$(NO$_3$)$_2$].

Method II. Ligand **L1 – L12** (~0.2 mmol, 2 equiv.) was added to an acetonitrile solution of $Pd(NO_3)_2{\cdot}2H_2O$ (~0.1 mmol, 1 equiv. in 5 mL MeCN). After, the resulting mixture was heated under reflux for 12 h. The solvent was then evaporated under reduced pressure. The crude

product was redissolved in MeCN (1 mL) and reprecipitated by the addition of Et_2O (10 mL). This precipitate was centrifuged off, washed with Et_2O (2 x 10 mL), and dried under vacuum.

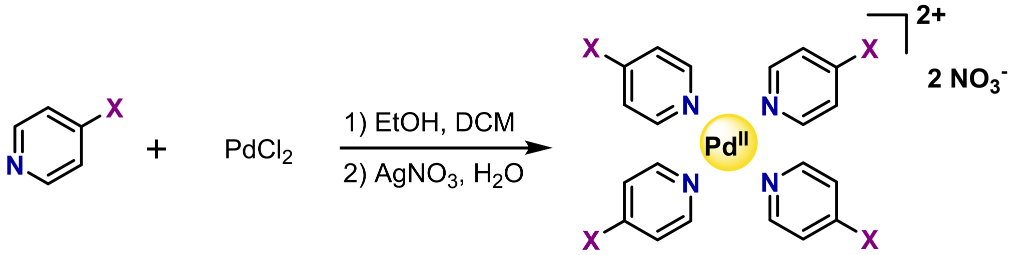

Scheme S3. Reaction scheme for the preparation of [Pd**L**$_4$]$(NO_3)_2$.

Method III. To a suspension of $PdCl_2$ (~0.1 mmol, 1 equiv.) in EtOH (5 mL), a solution of ligand **L1 – L12** (~1.0 mmol, 10 equiv.) in DCM (5 mL) was added, and the resulting mixture was stirred at room temperature for 1 h. After, $AgNO_3$ (~0.2 mmol, 2 equiv.) in 0.5 mL H_2O was added and the resulting suspension was stirred for an additional 12 h excluding light. The reaction mixture was filtered to remove AgCl, then the filtrate was evaporated under reduced pressure. The crude product was redissolved in DCM (1 mL) and reprecipitated by the addition of *n*-hexane (10 mL). This precipitate was centrifuged off, washed with *n*-hexane (2 x 10 mL), and dried under vacuum.

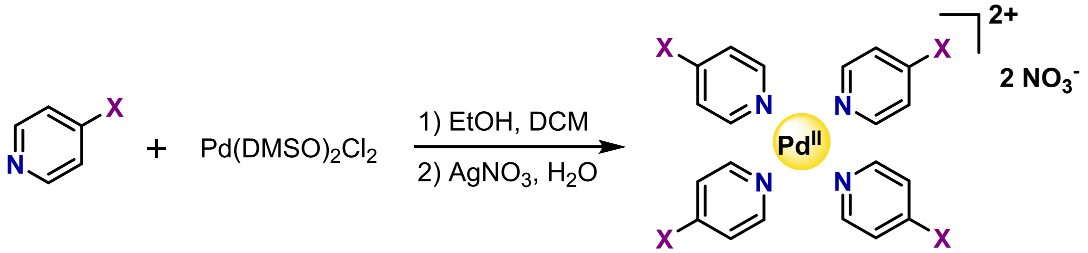

Scheme S4. Reaction scheme for the preparation of [Pd**L**$_4$]$(NO_3)_2$.

Method IV. To a suspension of $Pd(DMSO)_2Cl_2$ (~0.1 mmol, 1 equiv.) in EtOH (5 mL), a solution of ligand **L1 – L12** (~1.0 mmol, 10 equiv.) in DCM (5 mL) was added, and the resulting mixture was stirred at room temperature for 1 h. After, $AgNO_3$ (~0.2 mmol, 2 equiv.) in 0.5 mL H_2O was added and the resulting suspension was stirred for an additional 12 h excluding light. The reaction mixture was filtered to remove AgCl, then the filtrate was evaporated under reduced pressure. The crude product was redissolved in DCM (1 mL) and reprecipitated by the addition of *n*-hexane (10 mL). This precipitate was centrifuged off, washed with *n*-hexane (2 x 10 mL), and dried under vacuum.

3. Characterization of Pd(II) complexes

3.1. Pd(II) complexes based on pyridine (L1)

[Pd(**L1**)$_2$Cl$_2$]: The complex was prepared according to the method I, using $PdCl_2$ (19.5 mg, 0.11 mmol) and **L1** (17.7 µL, 0.22 mmol). Yield: 74%, 27.3 mg.

^{1}H NMR (300 MHz, $CDCl_3$) δ = 8.84 (d, *J* = 5.3 Hz, 2H), 7.79 (t, *J* = 7.8 Hz, 1H), 7.35 (t, *J* = 7.3 Hz, 2H).

ESI-MS calcd. for [Pd(**L1**)$_2$Cl$_2$+Na]$^+$ [M+Na]$^+$: *m/z* = 358.9139, observed: *m/z* = 358.9141.

[Pd(**L1**)$_2$(NO$_3$)$_2$]: The mixture of complexes was prepared according to the method II, using Pd(NO$_3$)$_2$·2H$_2$O (25.2 mg, 0.09 mmol) and **L1** (15.2 µL, 0.19 mmol). Yield: 69%, 25.2 mg.

trans-[Pd(**L1**)$_2$(NO$_3$)$_2$] (90%): ^{1}H NMR (300 MHz, CDCl$_3$) δ = 8.61 (d, *J* = 5.0 Hz, 2H), 7.92 (t, *J* = 7.9 Hz, 1H), 7.48 (t, *J* = 7.6 Hz, 2H).

cis-[Pd(**L1**)$_2$(NO$_3$)$_2$] (10%): ^{1}H NMR (300 MHz, CDCl$_3$) δ = 8.72 (d, *J* = 5.3 Hz, 2H), 7.84 (t, *J* = 7.8 Hz, 1H), 7.41 (t, *J* = 7.5 Hz, 2H).

ESI-MS calcd. for [Pd(**L1**)$_2$(NO$_3$)]$^+$ [M-NO$_3$]$^+$: *m/z* = 325.9756, observed: *m/z* = 325.9762.

[Pd(**L1**)$_4$](NO$_3$)$_2$: The complex was prepared according to the following procedure. Pd(NO$_3$)$_2$·2H$_2$O (30.1 mg, 0.11 mmol) was suspended in pyridine (5 mL) and heated under reflux for 12 h. The solvent was then evaporated under reduced pressure. The product was washed with chloroform (10 mL) and diethyl ether (2 x 10 mL), and dried under vacuum. Yield: 71%, 43.9 mg.

^{1}H NMR (300 MHz, CDCl$_3$) δ = 9.63 (d, *J* = 5.3 Hz, 2H), 7.76 (t, *J* = 7.6 Hz, 1H), 7.44 (t, *J* = 7.4 Hz, 2H).

ESI-MS calcd. for [Pd(**L1**)$_4$]$^{2+}$ [M-2NO$_3$]$^{2+}$: *m/z* = 211.0360, observed: *m/z* = 211.0357.

Table S1. ^{1}H NMR chemical shifts (δ, ppm) and shifts differences (Δδ, ppm) in CDCl$_3$ for ligand **L1** and Pd(II) complexes based on this ligand.

	**L1**	[Pd(**L1**)$_2$Cl$_2$]		*trans*-[Pd(**L1**)$_2$(NO$_3$)$_2$]		*cis*-[Pd(**L1**)$_2$(NO$_3$)$_2$]		[Pd(**L1**)$_4$](NO$_3$)$_2$	
	δ	δ	Δδ	δ	Δδ	δ	Δδ	δ	Δδ
**H^1**	8.62	8.84	0.22	8.61	-0.01	8.72	0.10	9.63	1.01
**H^2**	7.29	7.35	0.06	7.48	0.19	7.41	0.12	7.44	0.15
**H^3**	7.68	7.79	0.11	7.92	0.24	7.84	0.16	7.76	0.08

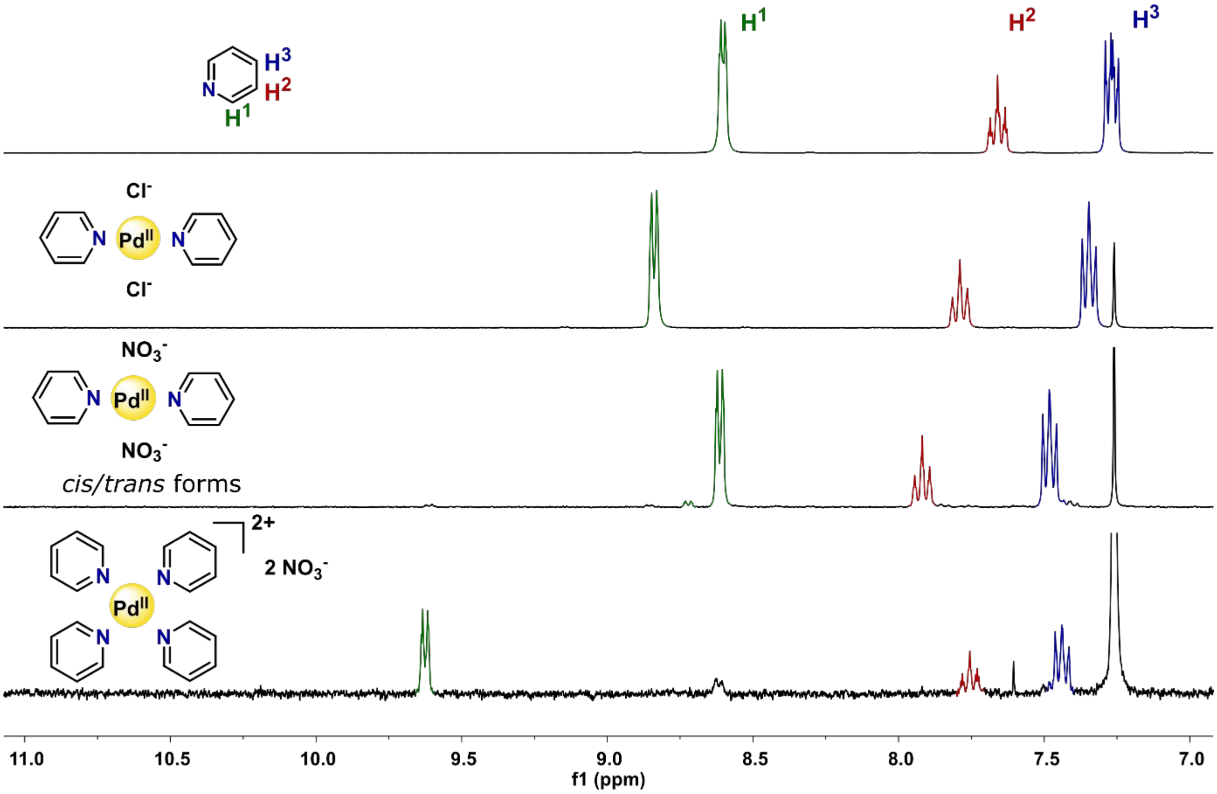

Figure S4. ^{1}H NMR spectra of Pd(II) complexes based on pyridine (**L1**).

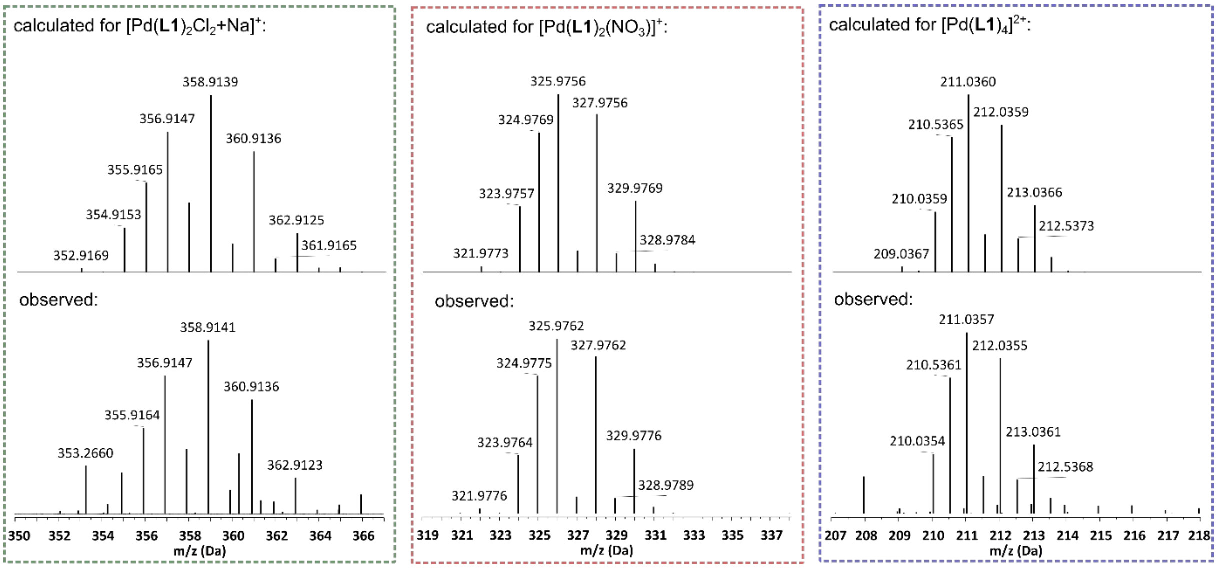

Figure S5. ESI-MS analysis of Pd(II) complexes based on pyridine (**L1**), showing the observed data (bottom) and the theoretical isotope model (top).

3.2. Pd(II) complexes based on 4-methylpyridine (L2)

[Pd(**L2**)$_2$Cl$_2$]: The complex was prepared according to the method I, using $PdCl_2$ (25.0 mg, 0.14 mmol) and **L2** (27.4 µL, 0.28 mmol). Yield: 73 %, 37.4 mg.

^{1}H NMR (300 MHz, $CDCl_3$) δ = 8.63 (d, *J* = 6.7 Hz, 2H), 7.13 (d, *J* = 6.4 Hz, 2H), 2.40 (s, 3H).

ESI-MS calcd. for [Pd(**L2**)$_2$Cl$_2$+Na]$^+$ [M+Na]$^+$: *m/z* = 386.9452, observed: *m/z* = 386.9468.

$[Pd(\mathbf{L2})_2(NO_3)_2]$: The mixture of complexes was prepared according to the method II, using $Pd(NO_3)_2{\cdot}2H_2O$ (26.2 mg, 0.10 mmol) and **L2** (19.1 µL, 0.20 mmol). Yield: 57%, 23.4 mg.

trans-$[Pd(\mathbf{L2})_2(NO_3)_2]$ (92%): ^1H NMR (300 MHz, $CDCl_3$) δ = 8.40 (d, *J* = 6.6 Hz, 2H), 7.25 (d, *J* = 6.1 Hz, 2H), 2.46 (s, 3H).

cis-$[Pd(\mathbf{L2})_2(NO_3)_2]$ (8%): ^1H NMR (300 MHz, $CDCl_3$) δ = 8.51 (d, *J* = 6.6 Hz, 2H), 7.19 (d, *J* = 6.0 Hz, 2H), 2.43 (s, 3H).

ESI-MS calcd. for $[Pd(\mathbf{L2})_2(NO_3)]^+$ $[M\text{-}NO_3]^+$: *m/z* = 354.0070, observed: *m/z* = 354.0088.

$[Pd(\mathbf{L2})_4](NO_3)_2$: The complex was prepared according to the method III, using $PdCl_2$ (21.6 mg, 0.12 mmol) and **L2** (118.5 µL, 1.22 mmol). Yield: 89%, 65.4 mg.

^1H NMR (300 MHz, $CDCl_3$) δ = 9.32 (d, *J* = 6.6 Hz, 2H), 7.18 (d, *J* = 6.6 Hz, 2H), 2.30 (s, 3H).

ESI-MS calcd. for $[Pd(\mathbf{L2})_4]^{2+}$ $[M\text{-}2NO_3]^{2+}$: *m/z* = 239.0674, observed: *m/z* = 239.0678.

Table S2. ^1H NMR chemical shifts (δ, ppm) and shifts differences (Δδ, ppm) in $CDCl_3$ for ligand **L2** and Pd(II) complexes based on this ligand.

	L2	$[Pd(\mathbf{L2})_2Cl_2]$		*trans*-$[Pd(\mathbf{L2})_2(NO_3)_2]$		*cis*-$[Pd(\mathbf{L2})_2(NO_3)_2]$		$[Pd(\mathbf{L2})_4](NO_3)_2$	
	δ	δ	Δδ	δ	Δδ	δ	Δδ	δ	Δδ
H^1	8.45	8.63	0.18	8.40	-0.05	8.51	0.06	9.32	0.87
H^2	7.09	7.13	0.04	7.25	0.16	7.19	0.10	7.18	0.09
H^3	2.34	2.40	0.06	2.46	0.12	2.43	0.09	2.30	-0.04

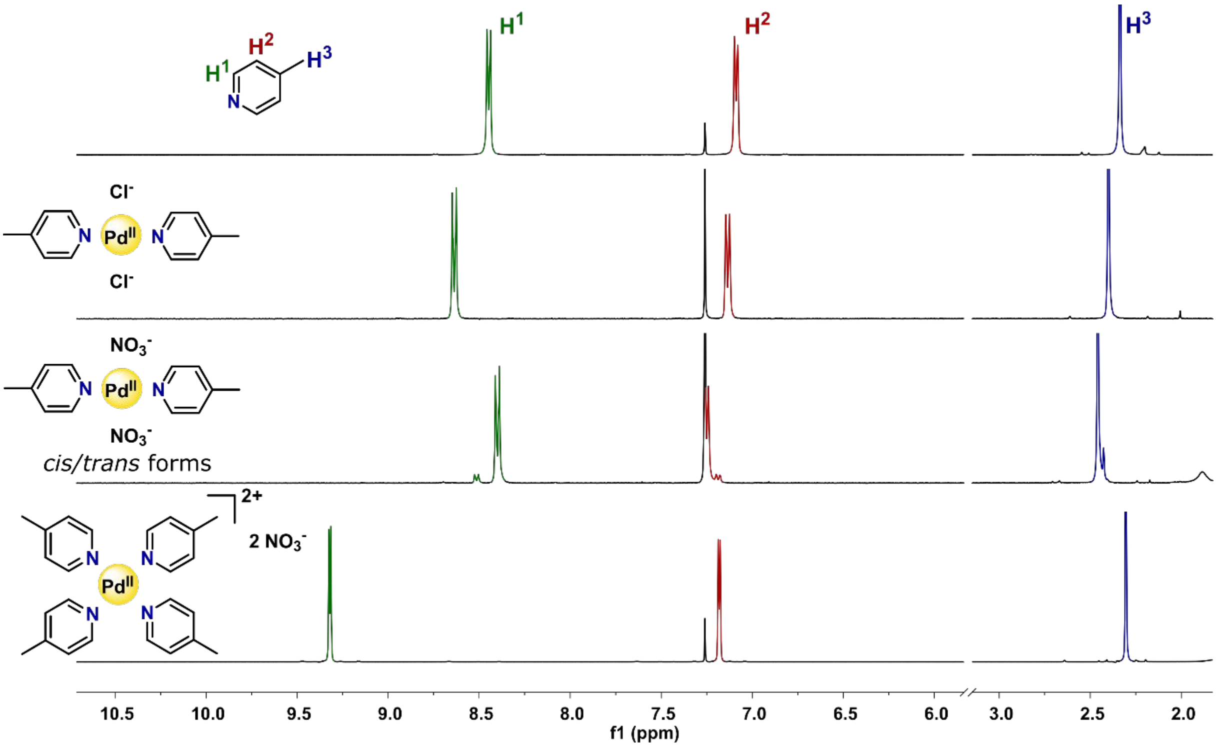

Figure S6. ^1H NMR spectra of Pd(II) complexes based on 4-methylpyridine (**L2**).

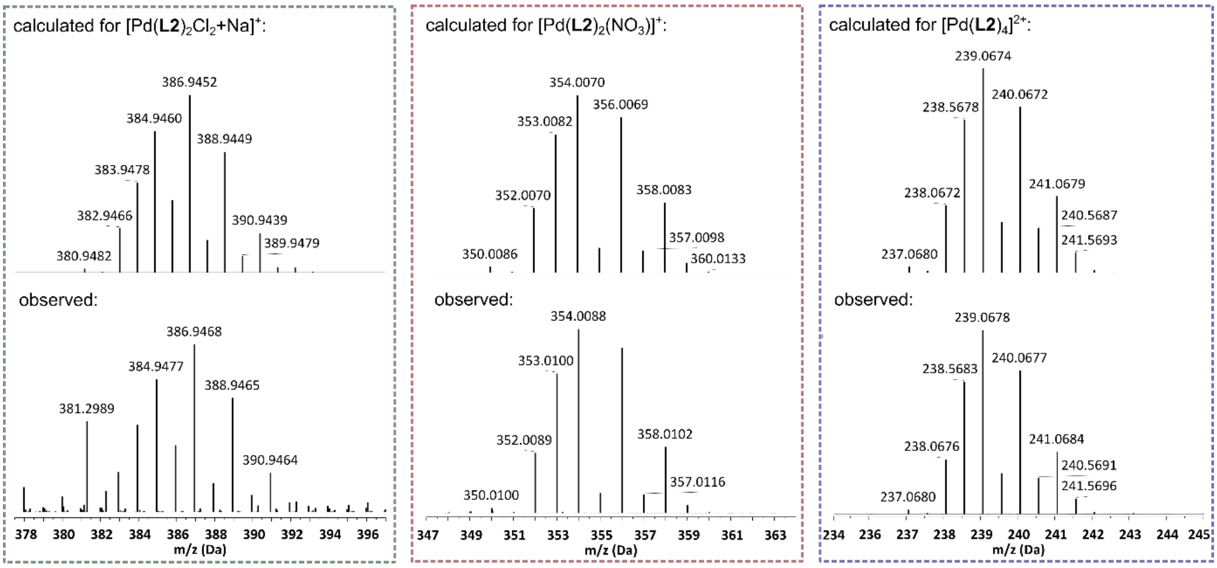

Figure S7. ESI-MS analysis of Pd(II) complexes based on 4-methylpyridine (**L2**), showing the observed data (bottom) and the theoretical isotope model (top).

3.3. Pd(II) complexes based on 4-methoxypyridine (L3)

[Pd(**L3**)$_2$Cl$_2$]: The complex was prepared according to the method I, using $PdCl_2$ (23.4 mg, 0.13 mmol) and **L3** (26.8 μL, 0.26 mmol). Yield: 68%, 35.3mg.

^{1}H NMR (300 MHz, $CDCl_3$) δ = 8.59 (d, *J* = 7.2 Hz, 2H), 6.81 (d, *J* = 7.2 Hz, 2H), 3.88 (s, 3H).

ESI-MS calcd. for [Pd(**L3**)$_2$Cl$_2$+Na]$^+$ [M+Na]$^+$: *m/z* = 418.9351, observed: *m/z* = 418.9360.

[Pd(**L3**)$_2$(NO$_3$)$_2$]: The mixture of complexes was prepared according to the method II, using $Pd(NO_3)_2 \cdot 2H_2O$ (26.5 mg, 0.10 mmol) and **L3** (20.2 μL, 0.20 mmol). Yield: 47%, 21.0 mg.

trans-[Pd(**L3**)$_2$(NO$_3$)$_2$] (93%): ^{1}H NMR (300 MHz, $CDCl_3$) δ = 8.33 (d, J = 7.2 Hz, 2H), 6.91 (d, J = 7.2 Hz, 2H), 3.91 (s, 3H).

cis-[Pd(**L3**)$_2$(NO$_3$)$_2$] (7%): ^{1}H NMR (300 MHz, $CDCl_3$) δ = 8.45 (d, *J* = 7.1 Hz, 2H), 6.85 (d, *J* = 7.1 Hz, 2H), 3.89 (s, 3H).

ESI-MS calcd. for [Pd(**L3**)$_2$(NO$_3$)]$^+$ [M-NO$_3$]$^+$: *m/z* = 385.9968, observed: *m/z* = 385.9977.

[Pd(**L3**)$_4$](NO$_3$)$_2$: The complex was prepared according to the method III, using $PdCl_2$ (23.0 mg, 0.13 mmol) and **L2** (131.7 μL, 1.30 mmol). Yield: 85%, 73.5 mg.

^{1}H NMR (300 MHz, $CDCl_3$) δ = 9.21 (d, *J* = 7.2 Hz, 2H), 6.87 (d, *J* = 7.2 Hz, 2H), 3.79 (s, 3H).

ESI-MS calcd. for [Pd(**L3**)$_4$]$^{2+}$ [M-2NO$_3$]$^{2+}$: *m/z* = 271.0572, observed: *m/z* = 271.0571.

Table S3. ^{1}H NMR chemical shifts (δ, ppm) and shifts differences (Δδ, ppm) in $CDCl_3$ for ligand **L3** and Pd(II) complexes based on this ligand.

	**L3**	$[Pd(\mathbf{L3})_2Cl_2]$		*trans*-$[Pd(\mathbf{L3})_2(NO_3)_2]$		*cis*-$[Pd(\mathbf{L3})_2(NO_3)_2]$		$[Pd(\mathbf{L3})_4](NO_3)_2$	
	δ	δ	Δδ	δ	Δδ	δ	Δδ	δ	Δδ
**H¹**	8.41	8.59	0.18	8.33	-0.08	8.45	0.04	9.21	0.80
**H²**	6.79	6.81	0.02	6.91	0.12	6.85	0.06	6.87	0.08
**H³**	3.83	3.88	0.05	3.91	0.08	3.89	0.06	3.79	-0.04

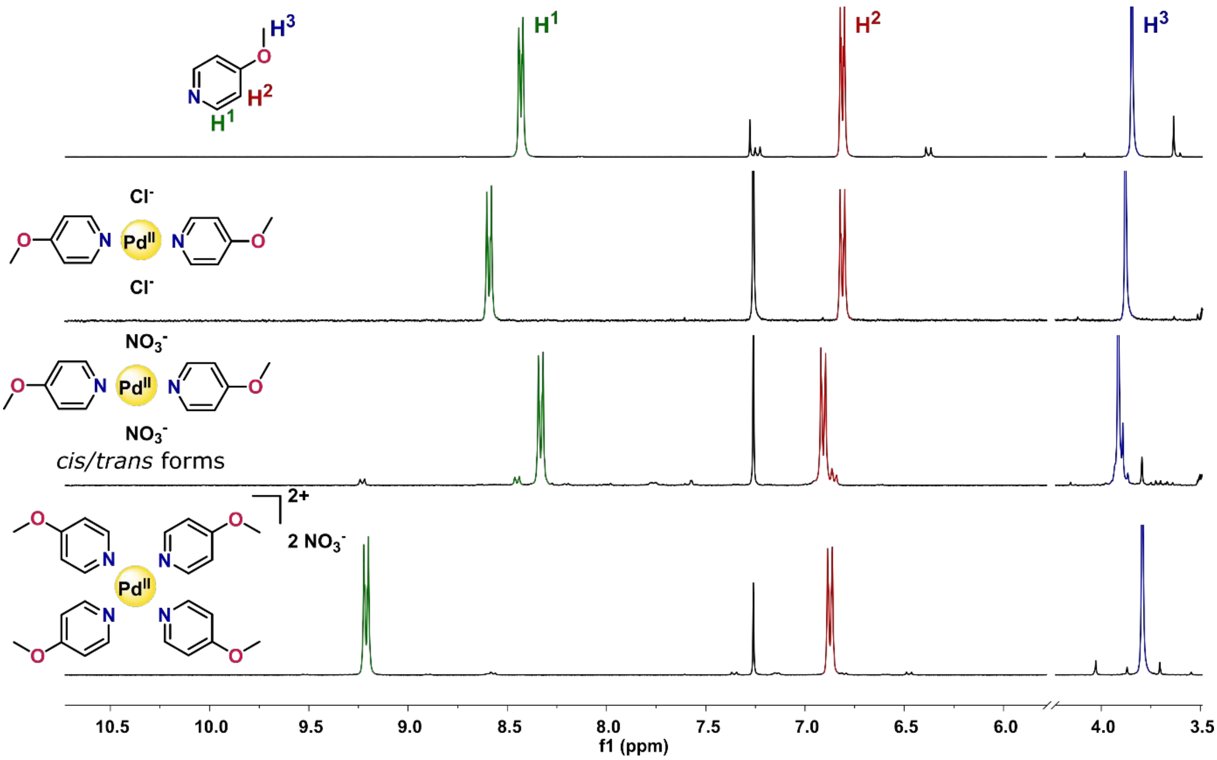

Figure S8. ^{1}H NMR spectra of Pd(II) complexes based on 4-methoxypyridine (**L3**).

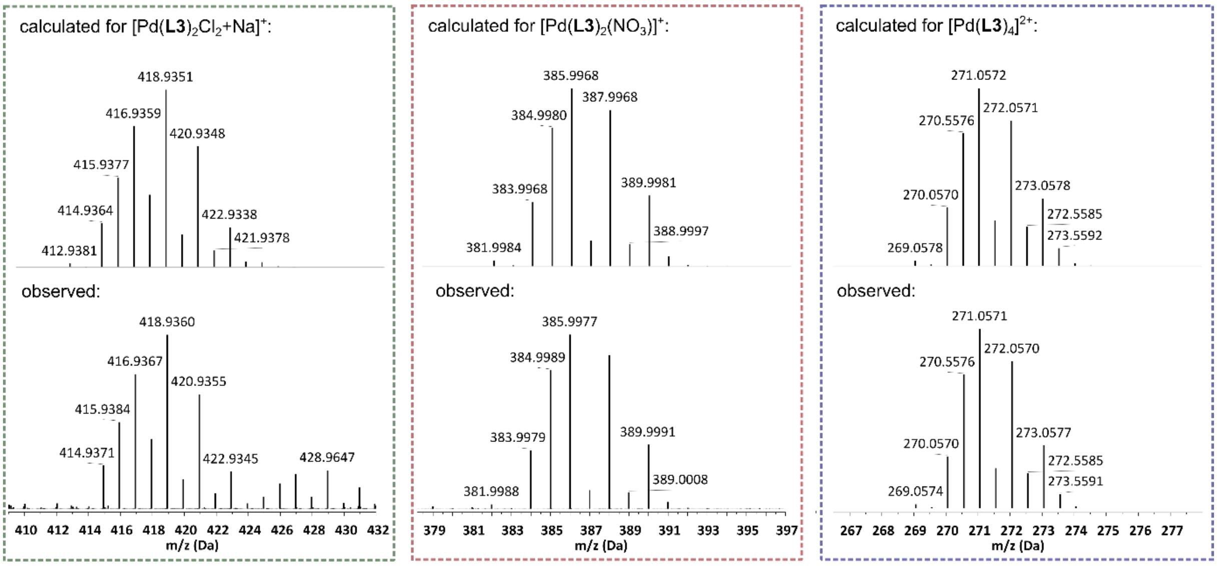

Figure S9. ESI-MS analysis of Pd(II) complexes based on 4-methoxypyridine (**L3**), showing the observed data (bottom) and the theoretical isotope model (top).

3.4. Pd(II) complexes based on methyl isonicotinate (L4)

[Pd(**L4**)$_2$Cl$_2$]: The complex was prepared according to the method I, using $PdCl_2$ (23.1 mg, 0.13 mmol) and **L4** (30.7 µL, 0.26 mmol). Yield: 80%, 47.1 mg.

^{1}H NMR (300 MHz, $CDCl_3$) δ = 9.01 (d, *J* = 6.7 Hz, 2H), 7.90 (d, *J* = 6.7 Hz, 2H), 3.99 (s, 3H).

ESI-MS calcd. for [Pd(**L4**)$_2$Cl$_2$+Na]$^+$ [M+Na]$^+$: *m/z* = 474.9250, observed: *m/z* = 474.9263.

[Pd(**L4**)$_2$(NO$_3$)$_2$]: The mixture of complexes was prepared according to the method II, using $Pd(NO_3)_2 \cdot 2H_2O$ (24.3 mg, 0.09 mmol) and **L4** (21.5 µL, 0.18 mmol). Yield: 64%, 29.5 mg.

trans-[Pd(**L4**)$_2$(NO$_3$)$_2$] (95%): ^{1}H NMR (300 MHz, $CDCl_3$) δ = 8.78 (d, *J* = 6.7 Hz, 2H), 8.03 (d, *J* = 6.7 Hz, 2H), 4.01 (s, 3H).

cis-[Pd(**L4**)$_2$(NO$_3$)$_2$] (5%): ^{1}H NMR (300 MHz, $CDCl_3$) δ = 8.89 (d, *J* = 6.7 Hz, 2H), 7.96 (d, *J* = 6.7 Hz, 2H), 4.00 (s, 3H).

ESI-MS calcd. for [Pd(**L4**)$_2$(NO$_3$)$_2$+MeCN+Li]$^+$ [M+MeCN+Li]$^+$: *m/z* = 552.0172, observed: *m/z* = 552.0177.

[Pd(**L4**)$_4$](NO$_3$)$_2$: The complex was prepared according to the method III, using $PdCl_2$ (21.8 mg, 0.12 mmol) and **L4** (145.2 µL, 1.23 mmol). Yield: 76%, 72.8 mg.

^{1}H NMR (300 MHz, $CDCl_3$) δ = 9.80 (d, *J* = 6.6 Hz, 2H), 7.98 (d, *J* = 6.6 Hz, 2H), 3.91 (s, 3H).

ESI-MS calcd. for [Pd(**L4**)$_4$]$^{2+}$ [M-2NO$_3$]$^{2+}$: *m/z* = 327.0471, observed: *m/z* = 327.0471.

Table S4. ^{1}H NMR chemical shifts (δ, ppm) and shifts differences (Δδ, ppm) in $CDCl_3$ for ligand **L4** and Pd(II) complexes based on this ligand.

	**L4**	[Pd(**L4**)$_2$Cl$_2$]		*trans*-[Pd(**L4**)$_2$(NO$_3$)$_2$]		*cis*-[Pd(**L4**)$_2$(NO$_3$)$_2$]		[Pd(**L4**)$_4$](NO$_3$)$_2$	
	δ	δ	Δδ	δ	Δδ	δ	Δδ	δ	Δδ
**H^1**	8.79	9.01	0.22	8.78	-0.01	8.89	0.10	9.80	1.01
**H^2**	7.84	7.90	0.06	8.03	0.19	7.96	0.12	7.98	0.14
**H^3**	3.96	3.99	0.03	4.01	0.05	4.00	0.04	3.91	-0.05

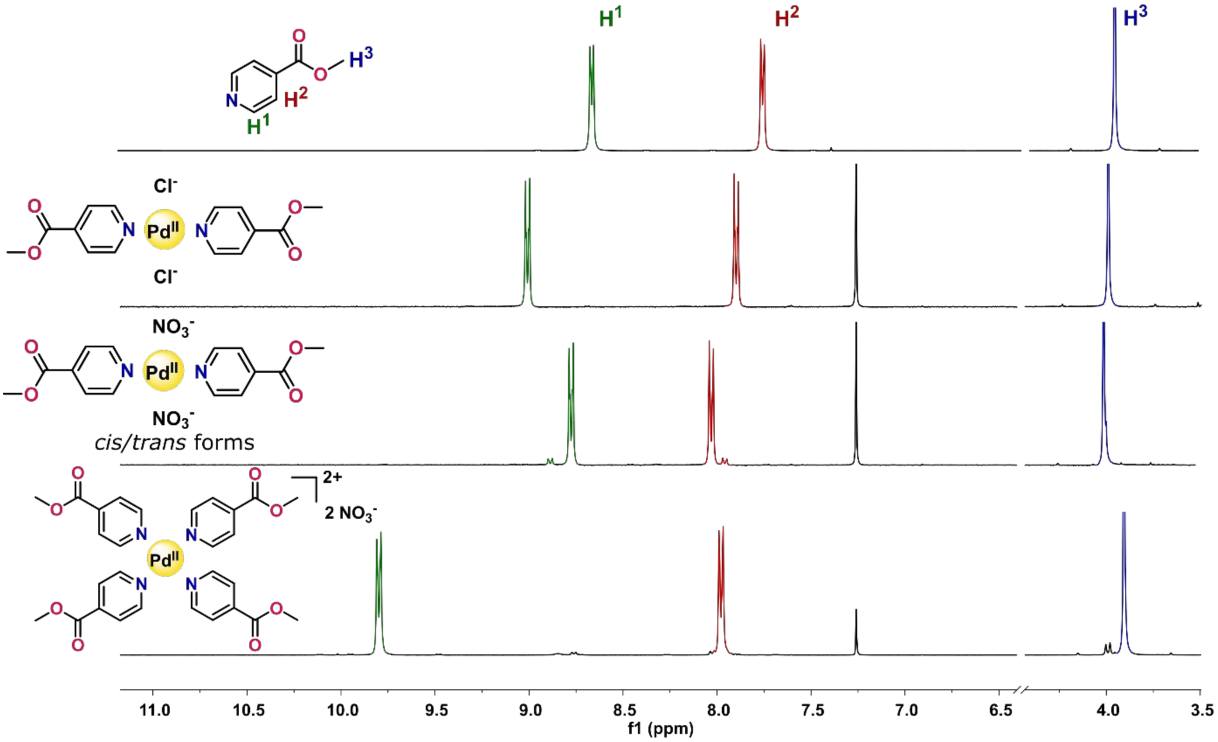

Figure S10. ^{1}H NMR spectra of Pd(II) complexes based on methyl isonicotinate (**L4**).

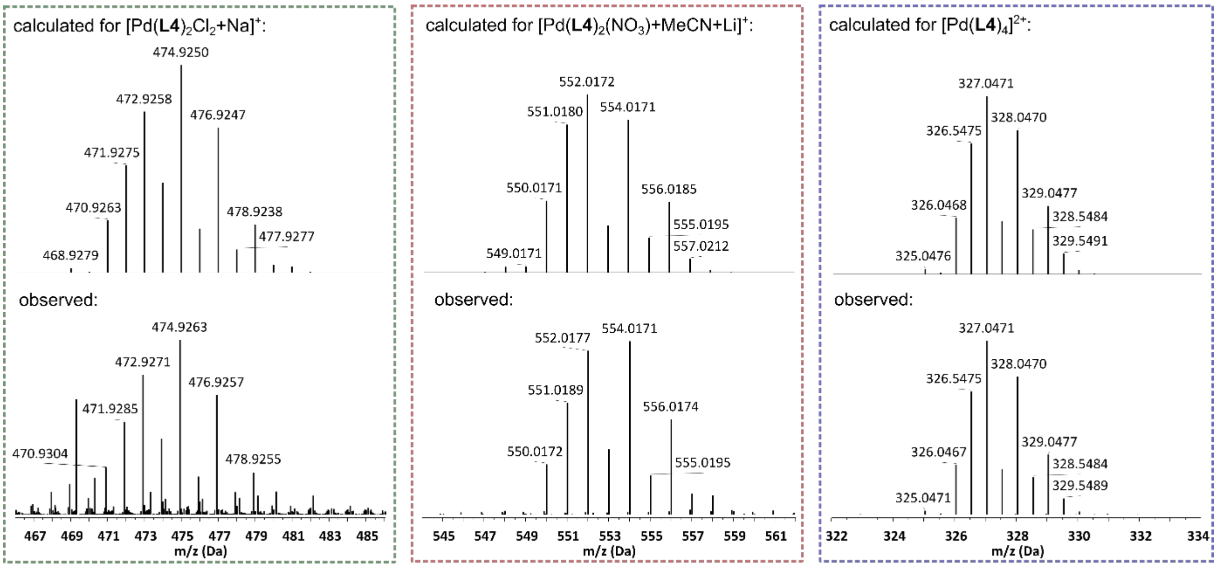

Figure S11. ESI-MS analysis of Pd(II) complexes based on methyl isonicotinate (**L4**), showing the observed data (bottom) and the theoretical isotope model (top).

3.5. Pd(II) complexes based on methyl 4-acetylpyridine (L5)

[Pd(**L5**)$_2$Cl$_2$]: The complex was prepared according to the method I, using $PdCl_2$ (20.9 mg, 0.12 mmol) and **L5** (26.1 µL, 0.24 mmol). Yield: 89%, 44.0 mg.

^{1}H NMR (600 MHz, $CDCl_3$) δ = 9.05 (d, J = 6.1 Hz, 2H), 7.78 (d, J = 6.1 Hz, 2H), 2.65 (s, 3H).

ESI-MS calcd. for [Pd(**L5**)$_2$Cl+DMSO]$^+$ [M-Cl+DMSO]$^+$: m/z = 462.9909, observed: m/z = 462.9903.

[Pd(**L5**)$_2$(NO$_3$)$_2$]: The mixture of complexes was prepared according to the method II, using Pd(NO$_3$)$_2$·2H$_2$O (27.0 mg, 0.10 mmol) and **L5** (22.4 μL, 0.20 mmol). Yield: 67%, 32.1 mg.

trans-[Pd(**L5**)$_2$(NO$_3$)$_2$] (91%): ^{1}H NMR (300 MHz, CDCl$_3$) δ = 8.82 (d, *J* = 6.3 Hz, 2H), 7.91 (d, *J* = 6.3 Hz, 2H), 2.68 (s, 3H).

cis-[Pd(**L5**)$_2$(NO$_3$)$_2$] (9%): ^{1}H NMR (300 MHz, CDCl$_3$) δ = 8.94 (d, *J* = 6.5 Hz, 2H), 7.85 (d, *J* = 6.5 Hz, 2H), 2.66 (s, 3H).

ESI-MS calcd. for [Pd(**L5**)$_2$(NO$_3$)]$^+$ [M-NO$_3$]$^+$: *m/z* = 409.9969, observed: *m/z* = 409.9971.

[Pd(**L5**)$_4$](NO$_3$)$_2$: The complex was prepared according to the method IV, using Pd(DMSO)$_2$Cl$_2$ (29.8 mg, 0.09 mmol) and **L5** (98.8 μL, 0.90 mmol). Yield: 65%, 41.5 mg.

^{1}H NMR (300 MHz, CDCl$_3$) δ = 9.87 (d, *J* = 6.7 Hz, 2H), 7.88 (d, *J* = 6.7 Hz, 2H), 2.55 (s, 3H).

ESI-MS calcd. for [Pd(**L5**)$_4$]$^{2+}$ [M-2NO$_3$]$^{2+}$: *m/z* = 295.0573, observed: *m/z* = 295.0571.

Table S5. ^{1}H NMR chemical shifts (δ, ppm) and shifts differences (Δδ, ppm) in CDCl$_3$ for ligand **L5** and Pd(II) complexes based on this ligand.

	**L5**	[Pd(**L5**)$_2$Cl$_2$]		*trans*-[Pd(**L5**)$_2$(NO$_3$)$_2$]		*cis*-[Pd(**L5**)$_2$(NO$_3$)$_2$]		[Pd(**L5**)$_4$](NO$_3$)$_2$	
	δ	δ	Δδ	δ	Δδ	δ	Δδ	δ	Δδ
**H^1**	8.79	9.05	0.26	8.82	0.03	8.94	0.15	9.87	1.08
**H^2**	7.70	7.78	0.08	7.91	0.21	7.85	0.15	7.88	0.18
**H^3**	2.61	2.65	0.04	2.68	0.07	2.66	0.05	2.55	-0.06

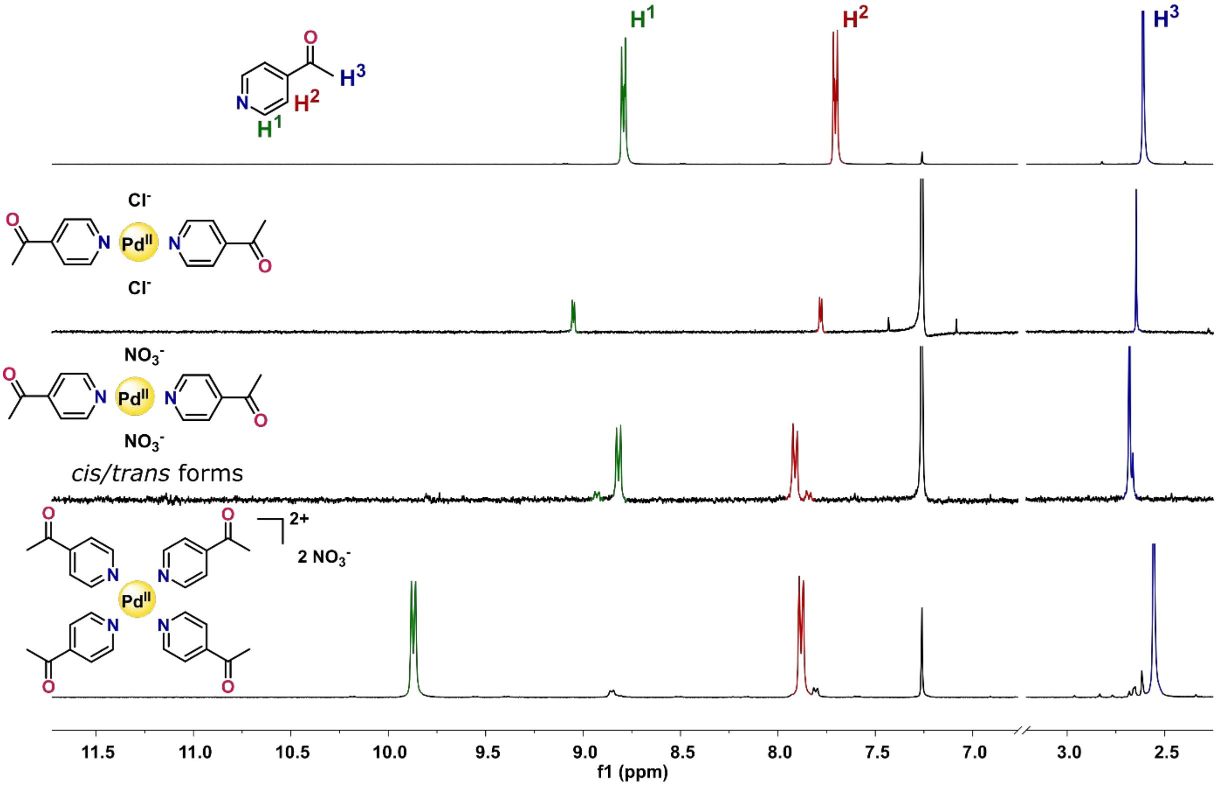

Figure S12. ^{1}H NMR spectra of Pd(II) complexes based on 4-acetylpyridine (**L5**).

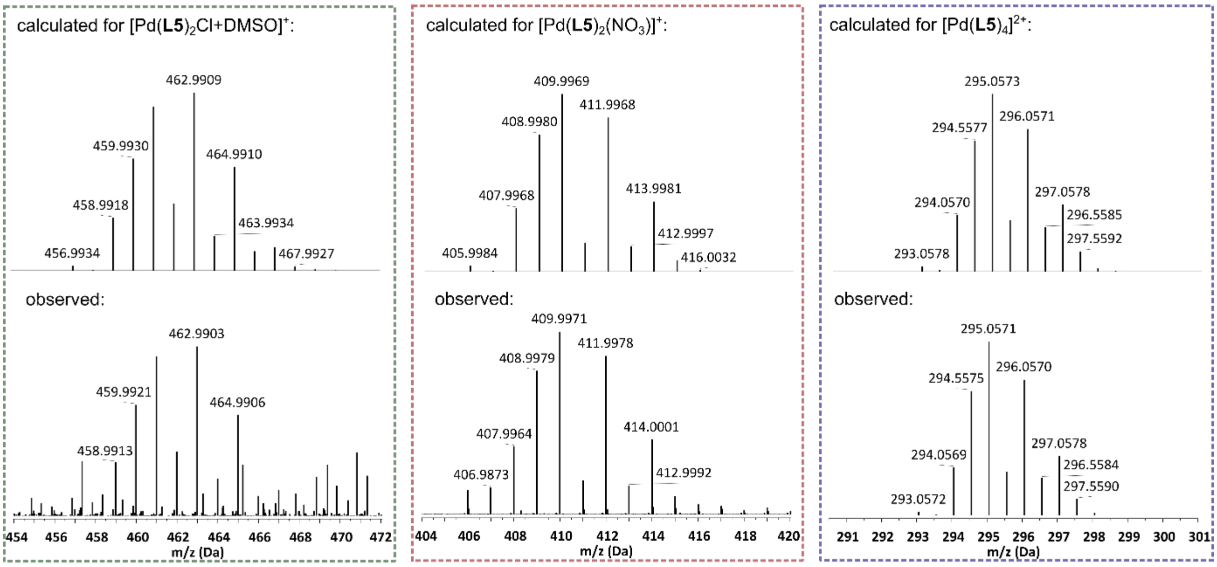

Figure S13. ESI-MS analysis of Pd(II) complexes based on 4-acetylpyridine (**L5**), showing the observed data (bottom) and the theoretical isotope model (top).

3.6. Pd(II) complexes based on 4-(dimethylaminio)pyridine (L6)

[Pd(**L6**)$_2$Cl$_2$]: The complex was prepared according to the method I, using $PdCl_2$ (20.0 mg, 0.11 mmol) and **L6** (27.6 mg, 0.22 mmol). Yield: 77%, 36.6 mg.

^{1}H NMR (600 MHz, $CDCl_3$) δ = 8.25 (d, J = 7.3 Hz, 2H), 6.40 (d, J = 7.3 Hz, 2H), 3.02 (s, 6H).

ESI-MS calcd. for [Pd(**L6**)$_2$Cl$_2$+Na]$^+$ [M+Na]$^+$: *m/z* = 444.9984, observed: *m/z* = 444.9981.

[Pd(**L6**)$_4$](NO$_3$)$_2$: The complex was prepared according to the method IV, using Pd(DMSO)$_2$Cl$_2$ (32.9 mg, 0.10 mmol) and **L6** (120.5 mg, 0.99 mmol). Yield: 81%, 57.4 mg.

^{1}H NMR (300 MHz, $CDCl_3$) δ = 8.71 (d, J = 7.3 Hz, 2H), 6.43 (d, J = 7.4 Hz, 2H), 2.93 (s, 6H).

ESI-MS calcd. for [Pd(**L6**)$_4$]$^{2+}$ [M-2NO$_3$]$^{2+}$: *m/z* = 297.1205, observed: *m/z* = 297.1206.

Table S6. ^{1}H NMR chemical shifts (δ, ppm) and shifts differences (Δδ, ppm) in $CDCl_3$ for ligand **L6** and Pd(II) complexes based on this ligand.

	**L6**	[Pd(**L6**)$_2$Cl$_2$]		[Pd(**L6**)$_4$](NO$_3$)$_2$	
	δ	δ	Δδ	δ	Δδ
**H^1**	8.21	8.25	0.04	8.71	0.50
**H^2**	6.47	6.40	-0.07	6.43	-0.04
**H^3**	2.99	3.02	0.03	2.93	-0.06

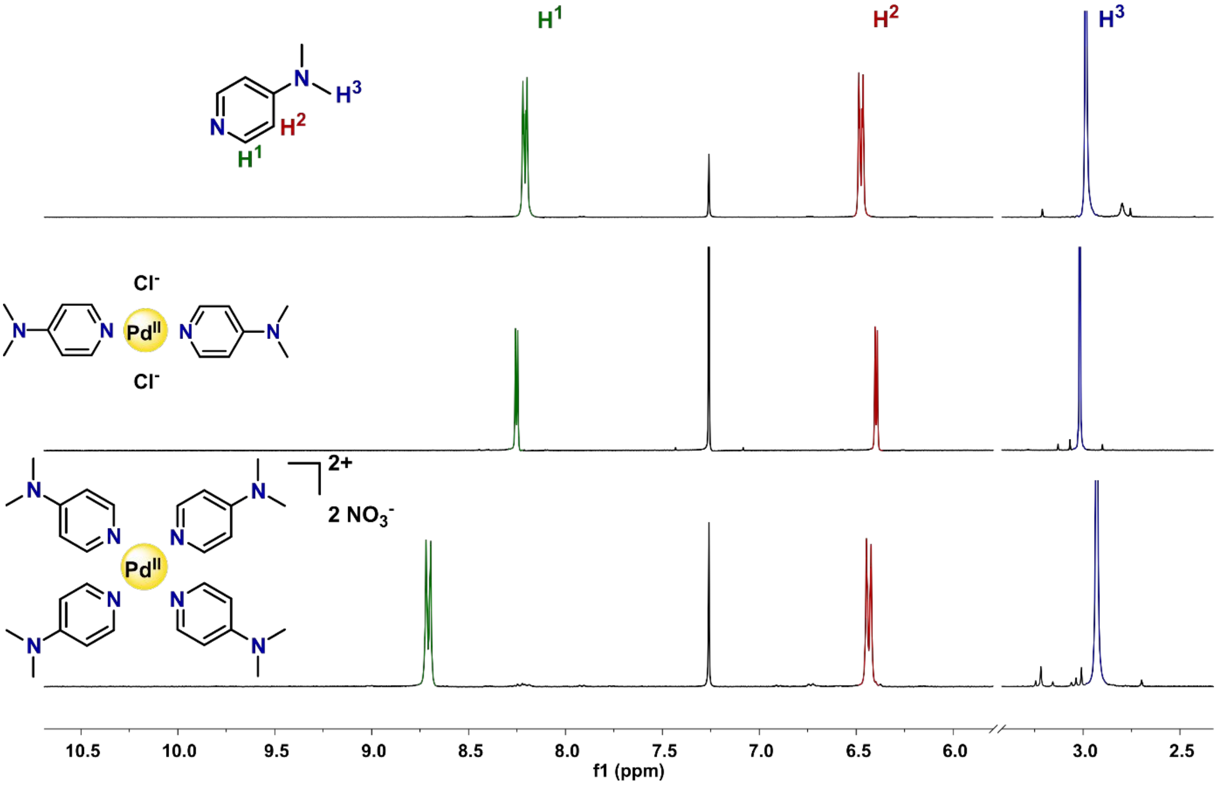

Figure S14. ^{1}H NMR spectra of Pd(II) complexes based on 4-(dimethylaminio)pyridine (**L6**).

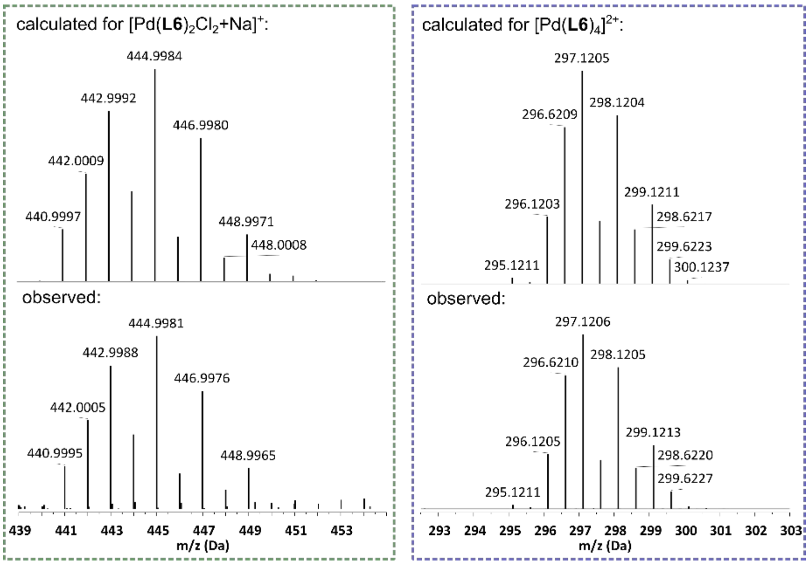

Figure S15. ESI-MS analysis of Pd(II) complexes based on 4-(dimethylaminio)pyridine (**L6**), showing the observed data (bottom) and the theoretical isotope model (top).

3.7. Pd(II) complexes based on 4-chloropyridine (L7)

[Pd(**L7**)$_2$Cl$_2$]: The complex was prepared according to the following procedure. K_2CO_3 (359.3 mg, 2.6 mmol) was added to the suspension of 4-chloropyridine hydrochloride (39.0 mg, 0.26 mmol) in acetonitrile (5 mL) and stirred in ice bath for 3 h. After, the solid residue was filtered off and ligand **L7** in the form of acetonitrile solution was added to a hot solution of $PdCl_2$ (23.0 mg, 0.13 mmol). The resulting mixture was heated under reflux for 12 h. The precipitate that formed was centrifuged off, washed with MeCN (10 mL) and Et_2O (2 x 10 mL), and dried under vacuum. Yield: 69%, 36.2 mg.

^{1}H NMR (300 MHz, $CDCl_3$) δ = 8.75 (d, *J* = 6.9 Hz, 2H), 7.37 (d, *J* = 6.9 Hz, 2H).

ESI-MS calcd. for $[Pd(\mathbf{L7})_2Cl]^+$ $[M-Cl]^+$: *m/z* = 368.8769, observed: *m/z* = 368.8772.

$[Pd(\mathbf{L7})_2(NO_3)_2]$: The mixture of complexes was prepared according to the following procedure. K_2CO_3 (304.1 mg, 2.20 mmol) was added to the suspension of 4-chloropyridine hydrochloride (33.0 mg, 0.22 mmol) in acetonitrile (5 mL) and stirred in ice bath for 3 h. After, the solid residue was filtered off and ligand **L7** in the form of acetonitrile solution was added to a hot solution of $Pd(NO_3)_2{\cdot}2H_2O$ (29.3 mg, 0.11 mmol). The resulting mixture was heated under reflux for 12 h. The solvent was then evaporated under reduced pressure. The crude product was redissolved in MeCN (1 mL) and reprecipitated by the addition of Et_2O (10 mL). This precipitate was centrifuged off, washed with Et_2O (3 x 10 mL), and dried under vacuum. Yield: 58%, 29.2 mg.

trans-$[Pd(\mathbf{L7})_2(NO_3)_2]$ (95%): ^{1}H NMR (300 MHz, $CDCl_3$) δ = 8.50 (d, *J* = 6.9 Hz, 2H), 7.50 (d, *J* = 6.9 Hz, 2H).

cis-$[Pd(\mathbf{L7})_2(NO_3)_2]$ (5%): ^{1}H NMR (300 MHz, $CDCl_3$) δ = 8.61 (d, *J* = 6.7 Hz, 2H), 7.43 (d, *J* = 6.7 Hz, 2H).

ESI-MS calcd. for $[Pd(\mathbf{L7})_2(NO_3)]^+$ $[M-NO_3]^+$: *m/z* = 395.8963, observed: *m/z* = 395.8973.

$[Pd(\mathbf{L7})_4](NO_3)_2$: The complex was prepared according to the following procedure.

K_2CO_3 (1.66 g, 12.0 mmol) was added to the suspension of 4-chloropyridine hydrochloride (180.0 mg, 1.20 mmol) in chloroform (10 mL) and stirred in ice bath for 3 h. After, the solid residue was filtered off. To a suspension of $Pd(DMSO)_2Cl_2$ (40.0 mg, 0.12 mmol) in EtOH (5 mL), a chloroform solution of ligand **L7** in DCM (5 mL) was added, and the resulting mixture was stirred at 0°C for 1 h. After, $AgNO_3$ (40.8 mg, 0.24 mmol) in 0.5 mL H_2O was added and the resulting suspension was stirred for an additional 12 h excluding light. The reaction mixture was filtered to remove AgCl, then the filtrate was evaporated under reduced pressure. The crude product was redissolved in chloroform (1 mL) and reprecipitated by the addition of *n*-hexane (10 mL). This precipitate was centrifuged off, washed with *n*-hexane (3 x 10 mL), and dried under vacuum. Yield: 65%, 53.4 mg.

^{1}H NMR (300 MHz, $CDCl_3$) δ = 9.52 (d, *J* = 7.0 Hz, 2H), 7.47 (d, *J* = 6.9 Hz, 2H).

ESI-MS calcd. for $[Pd(\mathbf{L7})_4]^{2+}$ $[M-2NO_3]^{2+}$: *m/z* = 279.9569, observed: *m/z* = 279.9578.

Table S7. ^{1}H NMR chemical shifts (δ, ppm) and shifts differences (Δδ, ppm) in $CDCl_3$ for ligand **L7** and Pd(II) complexes based on this ligand.

	**L7**	$[Pd(\mathbf{L7})_2Cl_2]$		*trans*-$[Pd(\mathbf{L7})_2(NO_3)_2]$		*cis*-$[Pd(\mathbf{L7})_2(NO_3)_2]$		$[Pd(\mathbf{L7})_4](NO_3)_2$	
	δ	δ	Δδ	δ	Δδ	δ	Δδ	δ	Δδ
**H^1**	8.49	8.75	0.26	8.50	0.01	8.61	0.12	9.52	1.03
**H^2**	7.30	7.37	0.07	7.50	0.20	7.43	0.13	7.47	0.17

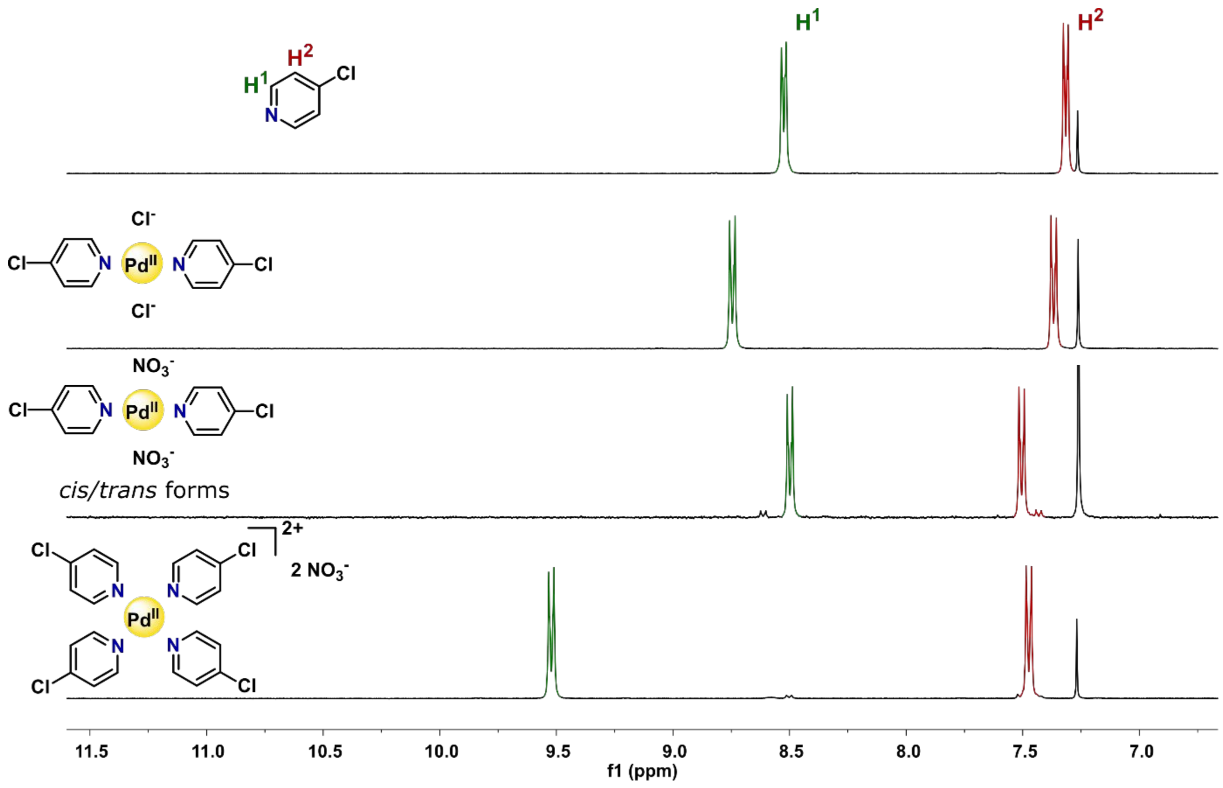

Figure S16. ^{1}H NMR spectra of Pd(II) complexes based on 4-chloropyridine (**L7**).

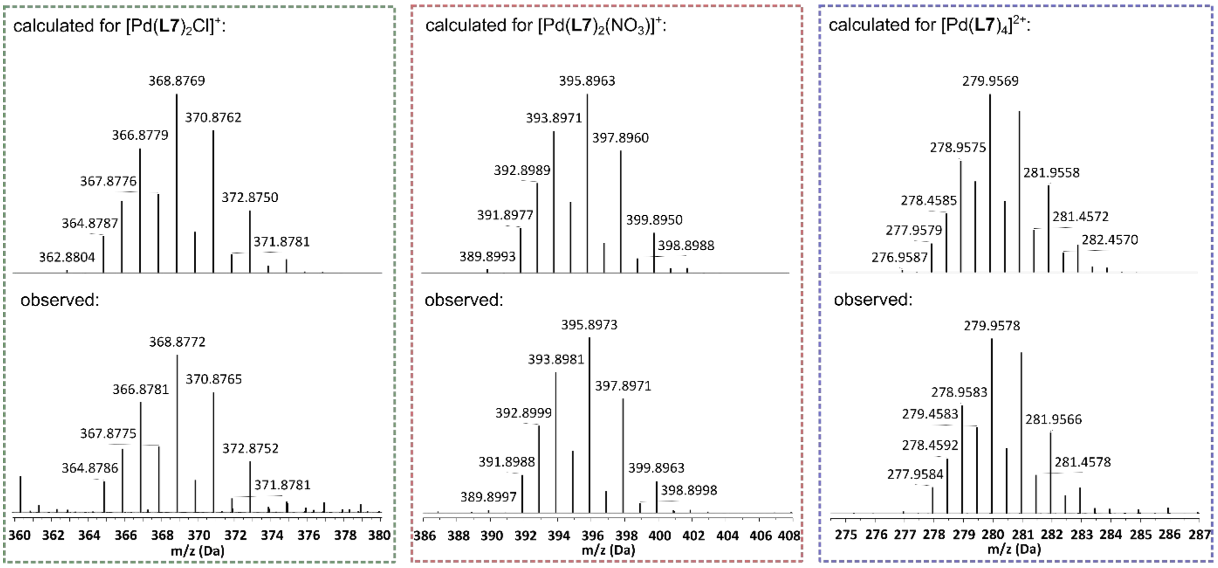

Figure S17. ESI-MS analysis of Pd(II) complexes based on 4-chloropyridine (**L7**), showing the observed data (bottom) and the theoretical isotope model (top).

3.8. Pd(II) complexes based on 4-pyridinecarbonitrile (L8)

[Pd(**L8**)$_2$Cl$_2$]: The complex was prepared according to the method I, using $PdCl_2$ (18.5 mg, 0.10 mmol) and **L8** (21.7 mg, 0.21 mmol). Yield: 85%, 34.2 mg.

^{1}H NMR (600 MHz, $CDCl_3$) δ = 9.08 (d, *J* = 6.8 Hz, 2H), 7.62 (d, *J* = 6.7 Hz, 2H).

$[Pd(\textbf{L8})_2(NO_3)_2]$: The complex was prepared according to the method II, using $Pd(NO_3)_2 \cdot 2H_2O$ (24.1 mg, 0.09 mmol) and **L8** (18.8 mg, 0.18 mmol). Yield: 87%, 34.5 mg.

trans-$[Pd(\textbf{L8})_2(NO_3)_2]$ (69%): ^{1}H NMR (300 MHz, $CDCl_3$) δ = 8.83 (d, *J* = 6.7 Hz, 2H), 7.78 (d, *J* = 6.7 Hz, 2H).

cis-$[Pd(\textbf{L8})_2(NO_3)_2]$ (31%): ^{1}H NMR (300 MHz, $CDCl_3$) δ = 8.94 (d, *J* = 6.9 Hz, 2H), 7.71 (d, *J* = 6.9 Hz, 2H).

ESI-MS calcd. for $[Pd(\textbf{L8})_2(NO_3)]^+$ $[M-NO_3]^+$: *m/z* = 375.9661, observed: *m/z* = 375.9688.

Table S8. ^{1}H NMR chemical shifts (δ, ppm) and shifts differences (Δδ, ppm) in $CDCl_3$ for ligand **L8** and Pd(II) complexes based on this ligand.

	**L8**	$[Pd(\textbf{L8})_2Cl_2]$		*trans*-$[Pd(\textbf{L8})_2(NO_3)_2]$		*cis*-$[Pd(\textbf{L8})_2(NO_3)_2]$	
	δ	δ	Δδ	δ	Δδ	δ	Δδ
**H¹**	8.79	9.08	0.29	8.83	0.04	8.94	0.15
**H²**	7.52	7.62	0.10	7.78	0.26	7.71	0.19

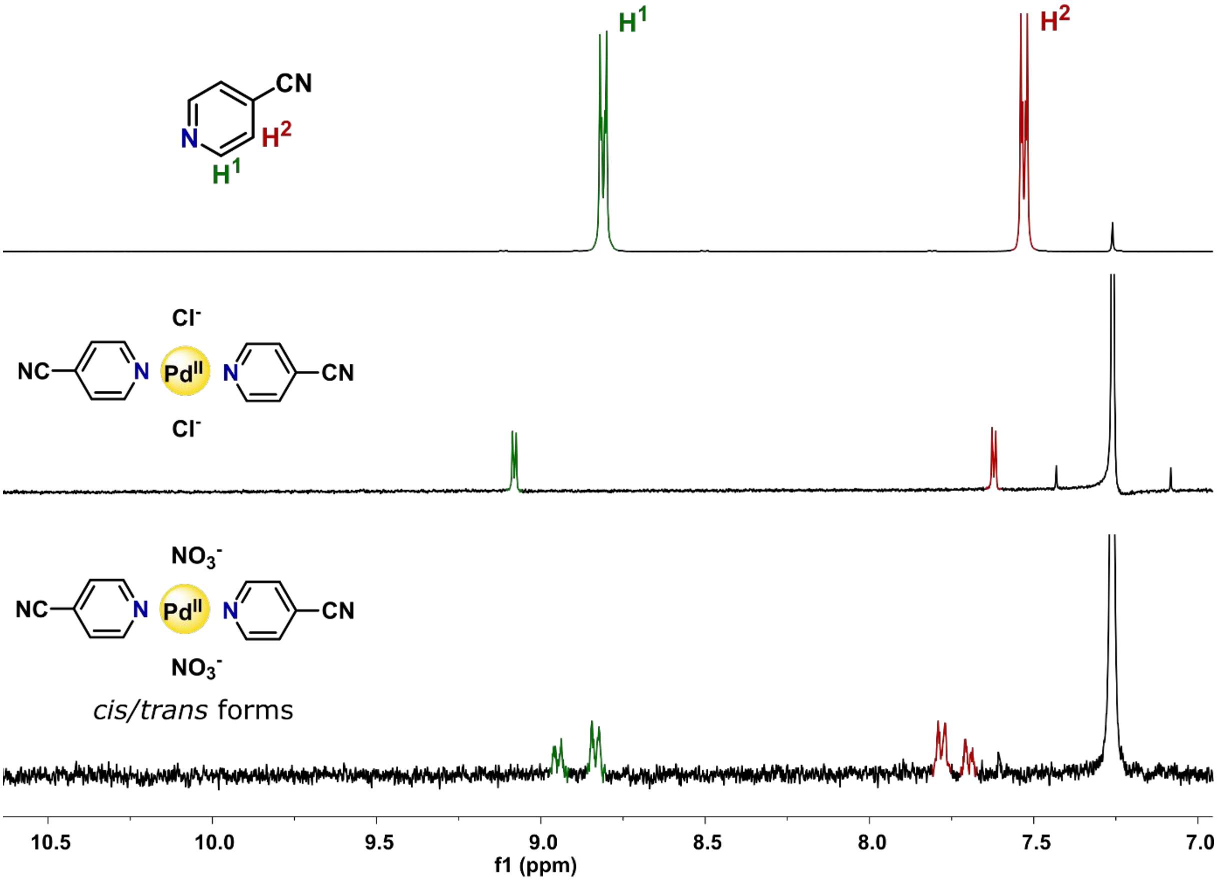

Figure S18. ^{1}H NMR spectra of Pd(II) complexes based on 4-pyridinecarbonitrile (**L8**).

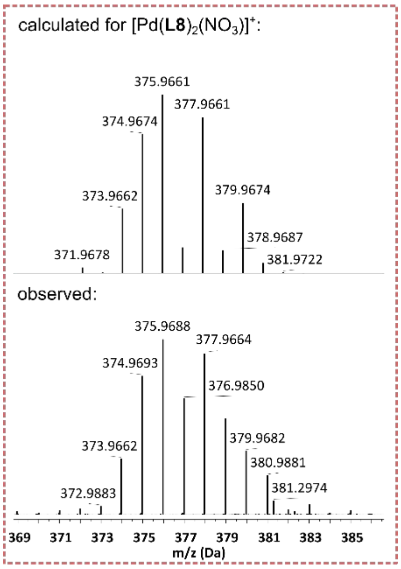

Figure S19. ESI-MS analysis of Pd(II) complex based on 4-pyridinecarbonitrile (**L8**), showing the observed data (bottom) and the theoretical isotope model (top).

3.9. Pd(II) complexes based on 4-(trifluoromethyl)pyridine (L9)

[Pd(**L9**)$_2$Cl$_2$]: The complex was prepared according to the method I, using $PdCl_2$ (21.2 mg, 0.12 mmol) and **L9** (27.8 μL, 0.24 mmol). Yield: 76%, 42.8 mg.

^{1}H NMR (600 MHz, $CDCl_3$) δ = 9.09 (d, *J* = 6.4 Hz, 2H), 7.62 (d, *J* = 6.5 Hz, 2H).

ESI-MS calcd. for [Pd(**L9**)$_2$Cl+DMSO]$^+$ [M-Cl+DMSO]$^+$: *m/z* = 514.9445, observed: *m/z* = 514.9462.

[Pd(**L9**)$_2$(NO$_3$)$_2$]: The complex was prepared according to the method II, using $Pd(NO_3)_2 \cdot 2H_2O$ (23.8 mg, 0.09 mmol) and **L9** (20.7 μL, 0.18 mmol). Yield: 61%, 28.6 mg.

^{1}H NMR (300 MHz, $CDCl_3$) δ = 8.86 (d, *J* = 6.4 Hz, 2H), 7.77 (d, *J* = 6.5 Hz, 2H).

ESI-MS calcd. for [Pd(**L9**)$_2$(NO$_3$)]$^+$ [M-NO$_3$]$^+$: *m/z* = 461.9504, observed: *m/z* = 461.9521.

Table S9. ^{1}H NMR chemical shifts (δ, ppm) and shifts differences (Δδ, ppm) in $CDCl_3$ for ligand **L9** and Pd(II) complexes based on this ligand.

	**L9**	[Pd(**L9**)$_2$Cl$_2$]		[Pd(**L9**)$_2$(NO$_3$)$_2$]	
	δ	δ	Δδ	δ	Δδ
**H^1**	8.82	9.09	0.27	8.86	0.04
**H^2**	7.52	7.62	0.10	7.77	0.25

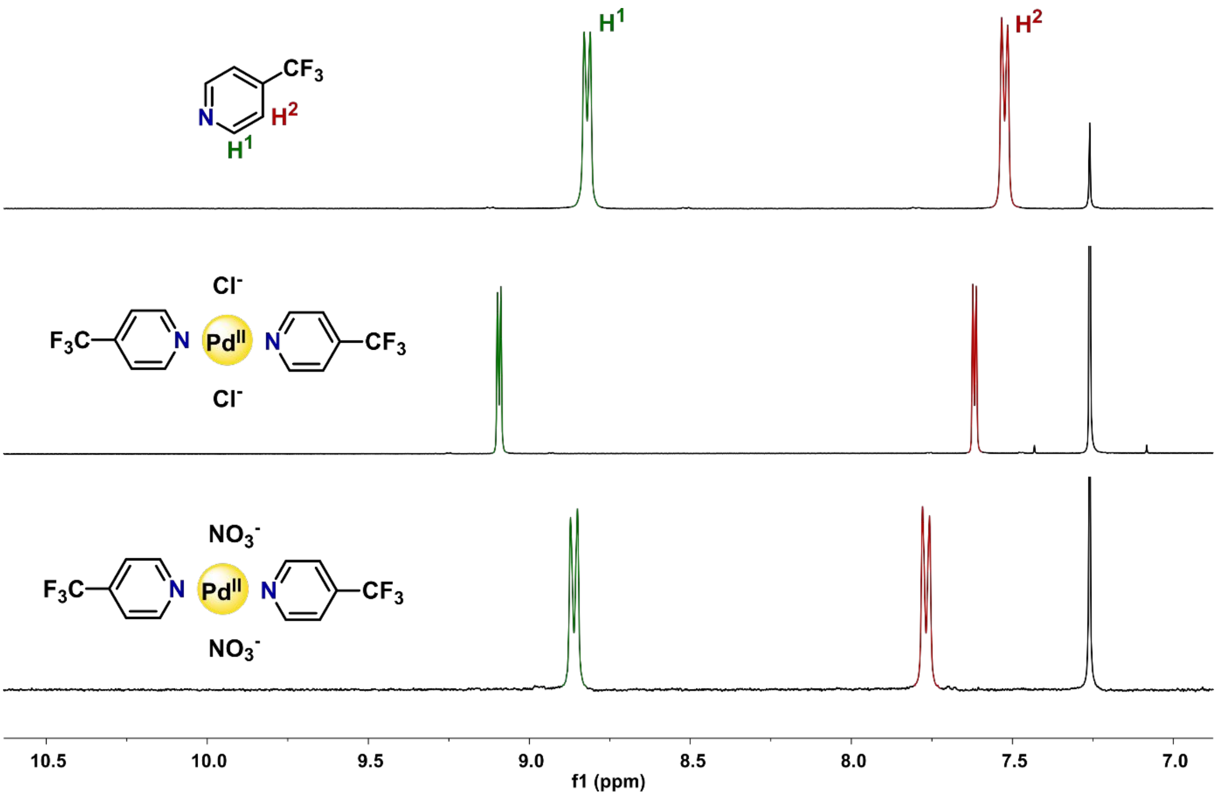

Figure S20. ^{1}H NMR spectra of Pd(II) complexes based on 4-(trifluoromethyl)pyridine (**L9**).

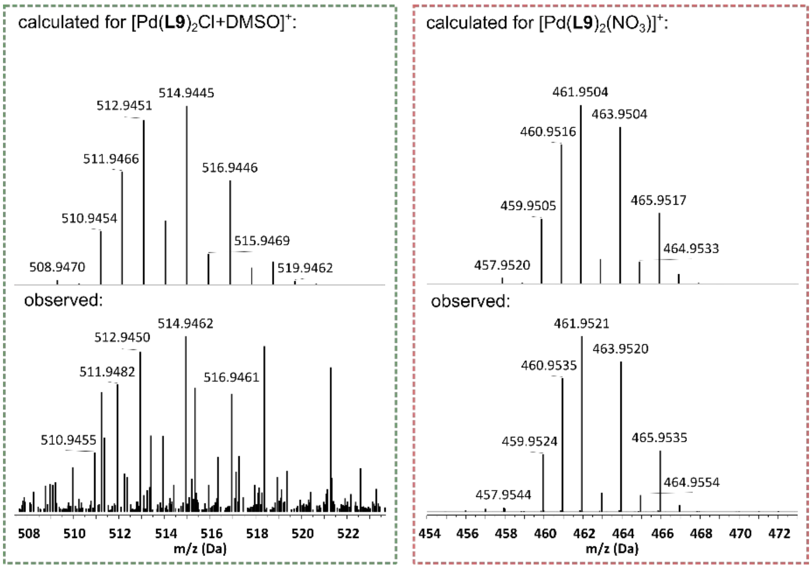

Figure S21. ESI-MS analysis of Pd(II) complexes based on 4-(trifluoromethyl)pyridine (**L9**), showing the observed data (bottom) and the theoretical isotope model (top).

3.10. Pd(II) complexes based on N-phenyl-1-(pyridin-4-yl)methanimine (L10)

[Pd(**L10**)$_2$Cl$_2$]: The complex was prepared according to the method I, using $PdCl_2$ (17.5 mg, 0.10 mmol) and **L10** (36.0 mg, 0.20 mmol). Yield: 71%, 38.0 mg.

^{1}H NMR (300 MHz, $CDCl_3$) δ = 8.97 (d, *J* = 6.6 Hz, 2H), 8.47 (s, 1H), 7.82 (d, *J* = 6.8 Hz, 2H), 7.45 (t, *J* = 7.7 Hz, 2H), 7.33 (t, *J* = 7.5 Hz, 1H), 7.28 (d, *J* = 7.3 Hz, 2H).

[Pd(**L10**)$_4$](NO$_3$)$_2$: The complex was prepared according to the method IV, using Pd(DMSO)$_2$Cl$_2$ (33.5 mg, 0.10 mmol) and **L10** (182.2 mg, 1.00 mmol). Yield: 84%, 89.9 mg.

^{1}H NMR (300 MHz, CDCl$_3$) δ = 9.79 (d, *J* = 6.6 Hz, 2H), 8.39 (s, 1H), 7.93 (d, *J* = 6.5 Hz, 2H), 7.41 (t, *J* = 7.4 Hz, 2H), 7.30 (t, *J* = 7.3 Hz, 1H), 7.21 (d, *J* = 7.3 Hz, 2H).

Table S10. ^{1}H NMR chemical shifts (δ, ppm) and shifts differences (Δδ, ppm) in CDCl$_3$ for ligand **L10** and Pd(II) complexes based on this ligand.

	**L10**	[Pd(**L10**)$_2$Cl$_2$]		[Pd(**L10**)$_4$](NO$_3$)$_2$	
	δ	δ	Δδ	δ	Δδ
**H^1**	8.77	8.97	0.20	9.79	1.02
**H^2**	7.76	7.82	0.06	7.93	0.17
**H^3**	8.46	8.47	0.01	8.39	-0.07
**H^4**	7.24	7.28	0.04	7.21	-0.03
**H^5**	7.43	7.45	0.02	7.41	-0.02
**H^6**	7.29	7.33	0.04	7.30	0.01

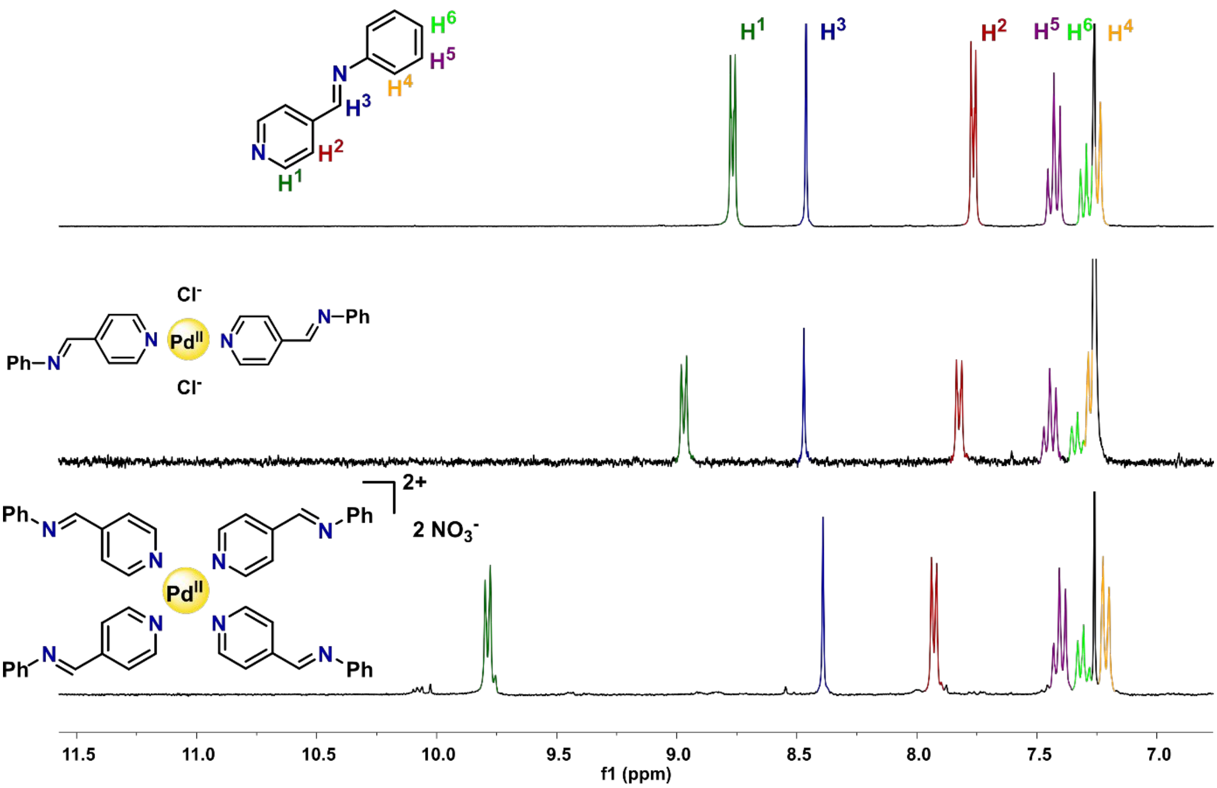

Figure S22. ^{1}H NMR spectra of Pd(II) complexes based on N-phenyl-1-(pyridin-4-yl)methanimine (**L10**).

3.11. Pd(II) complexes based on *N*-phenylisonicotinamide (L11)

[Pd(**L11**)$_2$Cl$_2$]: The complex was prepared according to the method I, using PdCl$_2$ (20.0 mg, 0.11 mmol) and **L11** (44.7 mg, 0.22 mmol). Yield: 82%, 53.1 mg.

^{1}H NMR (600 MHz, DMSO-*d*$_6$) δ = 10.69 (s, 1H), 8.99 (d, *J* = 6.8 Hz, 2H), 7.99 (d, *J* = 6.8 Hz, 2H), 7.76 (d, *J* = 7.7 Hz, 2H), 7.39 (t, *J* = 8.5 Hz, 2H), 7.16 (t, *J* = 7.3 Hz, 1H).

ESI-MS calcd. for [Pd(**L11**)$_2$Cl+DMSO]$^+$ [M-Cl+DMSO]$^+$: *m/z* = 617.0442, observed: *m/z* = 617.0437.

[Pd(**L11**)$_4$](NO$_3$)$_2$: The complex was prepared according to the following procedure. To a Schlenk flask, the solution of Pd(C$_6$H$_5$CN)$_2$Cl$_2$ (37.3 mg, 0.10 mmol) in MeCN (5 mL) and the ligand **L11** (198.2 mg, 1.00 mmol) were placed. The resulting mixture was stirred at room temperature for 0.5 h. After, AgNO$_3$ (34.0 mg, 0.20 mmol) in 0.5 mL H$_2$O was added. The Schlenk flask was sealed and the resulting suspension was stirred for additional 16 h at 65°C excluding light. The reaction mixture was filtered to remove AgCl, then the filtrate was evaporated under reduced pressure. The residue was washed with DCM (10 mL) and diethyl ether (3 x 10 mL), and dried under vacuum. Yield: 64%, 63.7 mg.

^{1}H NMR (300 MHz, DMSO-d_6) δ = 10.62 (s, 1H), 9.44 (d, J = 6.7 Hz, 2H), 8.16 (d, J = 6.8 Hz, 2H), 7.68 (d, J = 7.3 Hz, 2H), 7.37 (t, J = 7.9 Hz, 2H), 7.15 (t, J = 7.0 Hz, 1H).

ESI-MS calcd. for [Pd(**L11**)$_4$]$^{2+}$ [M-2NO$_3$]$^{2+}$: m/z = 449.1107, observed: m/z = 449.1123.

Table S11. ^{1}H NMR chemical shifts (δ, ppm) and shifts differences (Δδ, ppm) in DMSO-d_6 for ligand **L11** and Pd(II) complexes based on this ligand.

	**L11**	[Pd(**L11**)$_2$Cl$_2$]		[Pd(**L11**)$_4$](NO$_3$)$_2$	
	δ	δ	Δδ	δ	Δδ
**H^1**	8.79	8.99	0.20	9.44	0.65
**H^2**	7.86	7.99	0.13	8.16	0.30
**H^3**	7.77	7.76	-0.01	7.68	-0.09
**H^4**	7.38	7.39	0.01	7.37	-0.01
**H^5**	7.14	7.16	0.02	7.15	0.01
**H^6**	10.50	10.69	0.19	10.62	0.12

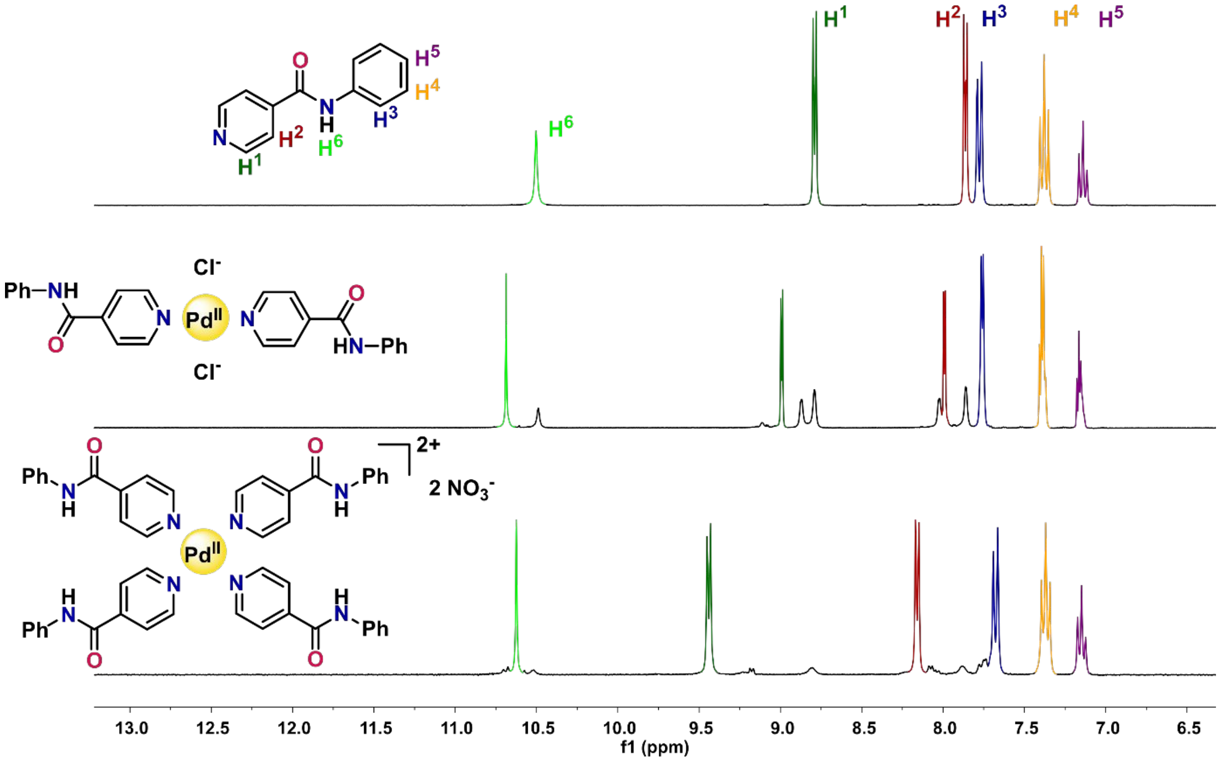

Figure S23. ^{1}H NMR spectra of Pd(II) complexes based on N-phenylisonicotinamide (**L11**).

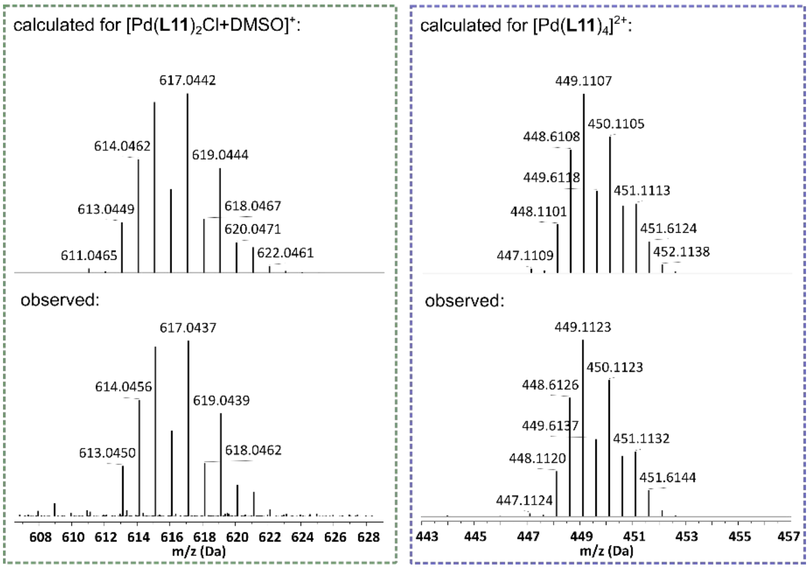

Figure S24. ESI-MS analysis of Pd(II) complexes based on *N*-phenylisonicotinamide (**L11**), showing the observed data (bottom) and the theoretical isotope model (top).

3.12. Pd(II) complexes based on 4,4-dimethyl-1-(pyridin-4-yl)pentane-1,3-dione (L12)

[Pd(**L12**)$_2$Cl$_2$]: The complex was prepared according to the method I, using $PdCl_2$ (19.9 mg, 0.11 mmol) and **L12** (46.1 mg, 0.22 mmol). Yield: 70%, 46.2 mg.

^{1}H NMR (300 MHz, $CDCl_3$) δ = 15.88 (s, 1H), 8.95 (d, *J* = 6.9 Hz, 2H), 7.73 (d, *J* = 6.9 Hz, 2H), 6.33 (s, 1H), 1.26 (s, 9H).

ESI-MS calcd. for [Pd(**L12**)$_2$Cl+MeCN]$^+$ [M-Cl+MeCN]$^+$: *m/z* = 594.1189, observed: *m/z* = 594.1170.

[Pd(**L12**)$_4$](NO_3)$_2$: The complex was prepared according to the method III, using $PdCl_2$ (23.3 mg, 0.13 mmol) and **L12** (269.7 mg, 1.30 mmol). Yield: 83%, 114.7 mg.

^{1}H NMR (300 MHz, $CDCl_3$) δ = 15.67 (s, 1H), 9.75 (d, *J* = 6.8 Hz, 2H), 7.83 (d, *J* = 6.8 Hz, 2H), 6.25 (s, 1H), 1.20 (s, 9H).

ESI-MS calcd. for [Pd(**L12**)$_4$]$^{2+}$ [M-2NO$_3$]$^{2+}$: *m/z* = 463.1726, observed: *m/z* = 463.1743.

Table S12. ^{1}H NMR chemical shifts (δ, ppm) and shifts differences (Δδ, ppm) in $CDCl_3$ for ligand **L12** and Pd(II) complexes based on this ligand.

	**L12**	[Pd(**L12**)$_2$Cl$_2$]		[Pd(**L12**)$_4$](NO_3)$_2$	
	δ	δ	Δδ	δ	Δδ
**H^1**	8.75	8.95	0.20	9.75	1.00
**H^2**	7.69	7.73	0.04	7.83	0.14
**H^3**	6.34	6.33	-0.01	6.25	-0.09
**H^4**	1.26	1.26	0.00	1.20	-0.06
**H^5**	16.09	15.88	-0.21	15.67	-0.42

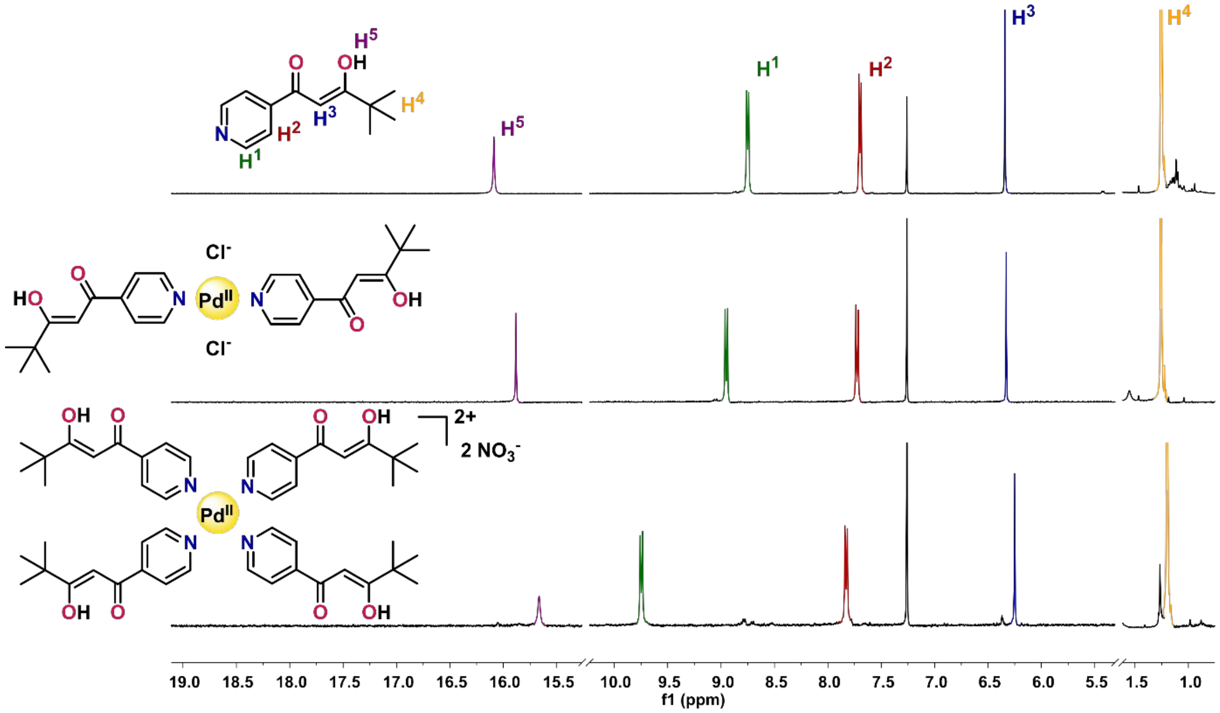

Figure S25. ^{1}H NMR spectra of Pd(II) complexes based on 4,4-dimethyl-1-(pyridin-4-yl)pentane-1,3-dione (**L12**).

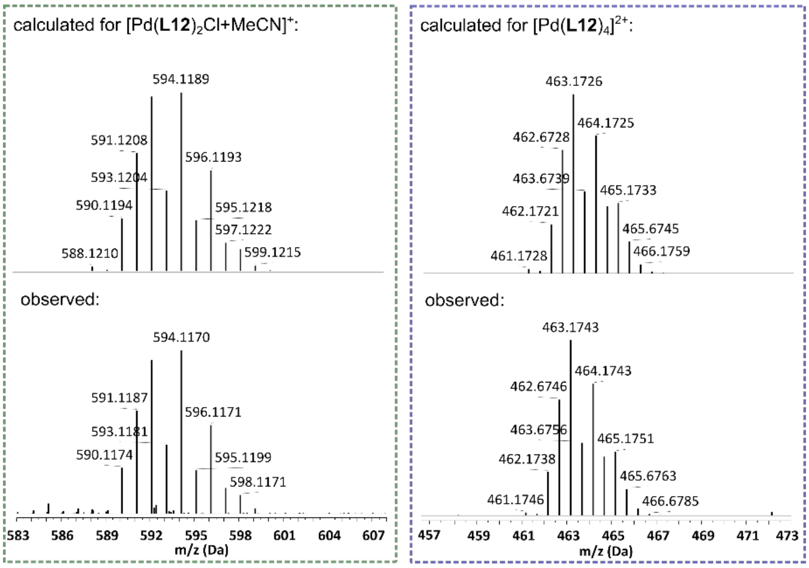

Figure S26. ESI-MS analysis of Pd(II) complexes based on 4,4-dimethyl-1-(pyridin-4-yl)pentane-1,3-dione (**L12**), showing the observed data (bottom) and the theoretical isotope model (top).

4. ^1H NMR analysis of Pd(II) complexes based on pyridine ligands

Table S13. ^1H NMR chemical shifts (δ, ppm) and shifts differences (Δδ, ppm) for ligands **L1** – **L12** and Pd(II) complexes based on these ligands.

		L	[Pd**L**$_2$Cl$_2$]		*trans*-[Pd**L**$_2$(NO$_3$)$_2$]		*cis*-[Pd**L**$_2$(NO$_3$)$_2$]		[Pd**L**$_4$](NO$_3$)$_2$	
		δ	δ	Δδ	δ	Δδ	δ	Δδ	δ	Δδ
**L1**	**H^1**	8.62	8.84	0.22	8.61	-0.01	8.72	0.10	9.63	1.01
	**H^2**	7.29	7.35	0.06	7.48	0.19	7.41	0.12	7.44	0.15
	**H^3**	7.68	7.79	0.11	7.92	0.24	7.84	0.16	7.76	0.08
**L2**	**H^1**	8.45	8.63	0.18	8.40	-0.05	8.51	0.06	9.32	0.87
	**H^2**	7.09	7.13	0.04	7.25	0.16	7.19	0.10	7.18	0.09
	**H^3**	2.34	2.40	0.06	2.46	0.12	2.43	0.09	2.30	-0.04
**L3**	**H^1**	8.41	8.59	0.18	8.33	-0.08	8.45	0.04	9.21	0.80
	**H^2**	6.79	6.81	0.02	6.91	0.12	6.85	0.06	6.87	0.08
	**H^3**	3.83	3.88	0.05	3.91	0.08	3.89	0.06	3.79	-0.04
**L4**	**H^1**	8.79	9.01	0.22	8.78	-0.01	8.89	0.10	9.80	1.01
	**H^2**	7.84	7.90	0.06	8.03	0.19	7.96	0.12	7.98	0.14
	**H^3**	3.96	3.99	0.03	4.01	0.05	4.00	0.04	3.91	-0.05
**L5**	**H^1**	8.79	9.05	0.26	8.82	0.03	8.94	0.15	9.87	1.08
	**H^2**	7.70	7.78	0.08	7.91	0.21	7.85	0.15	7.88	0.18
	**H^3**	2.61	2.65	0.04	2.68	0.07	2.66	0.05	2.55	-0.06
**L6**	**H^1**	8.21	8.25	0.04	-	-	-	-	8.71	0.50
	**H^2**	6.47	6.40	-0.07	-	-	-	-	6.43	-0.04
	**H^3**	2.99	3.02	0.03	-	-	-	-	2.93	-0.06
**L7**	**H^1**	8.49	8.75	0.26	8.50	0.01	8.61	0.12	9.52	1.03
	**H^2**	7.30	7.37	0.07	7.50	0.20	7.43	0.13	7.47	0.17
**L8**	**H^1**	8.79	9.08	0.29	8.83	0.04	8.94	0.15	-	-
	**H^2**	7.52	7.62	0.10	7.78	0.26	7.71	0.19	-	-
**L9**	**H^1**	8.82	9.09	0.27	8.86	0.04	-	-	-	-
	**H^2**	7.52	7.62	0.10	7.77	0.25	-	-	-	-
**L10**	**H^1**	8.77	8.97	0.20	-	-	-	-	9.79	1.02
	**H^2**	7.76	7.82	0.06	-	-	-	-	7.93	0.17
	**H^3**	8.46	8.47	0.01	-	-	-	-	8.39	-0.07
	**H^4**	7.24	7.28	0.04	-	-	-	-	7.21	-0.03
	**H^5**	7.43	7.45	0.02	-	-	-	-	7.41	-0.02
	**H^6**	7.29	7.33	0.04	-	-	-	-	7.30	0.01
**L11**	**H^1**	8.79	8.99	0.20	-	-	-	-	9.44	0.65
	**H^2**	7.86	7.99	0.13	-	-	-	-	8.16	0.30
	**H^3**	7.77	7.76	-0.01	-	-	-	-	7.68	-0.09
	**H^4**	7.38	7.39	0.01	-	-	-	-	7.37	-0.01
	**H^5**	7.14	7.16	0.02	-	-	-	-	7.15	0.01
	**H^6**	10.50	10.69	0.19	-	-	-	-	10.62	0.12
**L12**	**H^1**	8.75	8.95	0.20	-	-	-	-	9.75	1.00
	**H^2**	7.69	7.73	0.04	-	-	-	-	7.83	0.14
	**H^3**	6.34	6.33	-0.01	-	-	-	-	6.25	-0.09
	**H^4**	1.26	1.26	0.00	-	-	-	-	1.20	-0.06
	**H^5**	16.09	15.88	-0.21	-	-	-	-	15.67	-0.42

4.1. The relationship between chemical shifts in the ^{1}H NMR spectra and basicity of free ligands

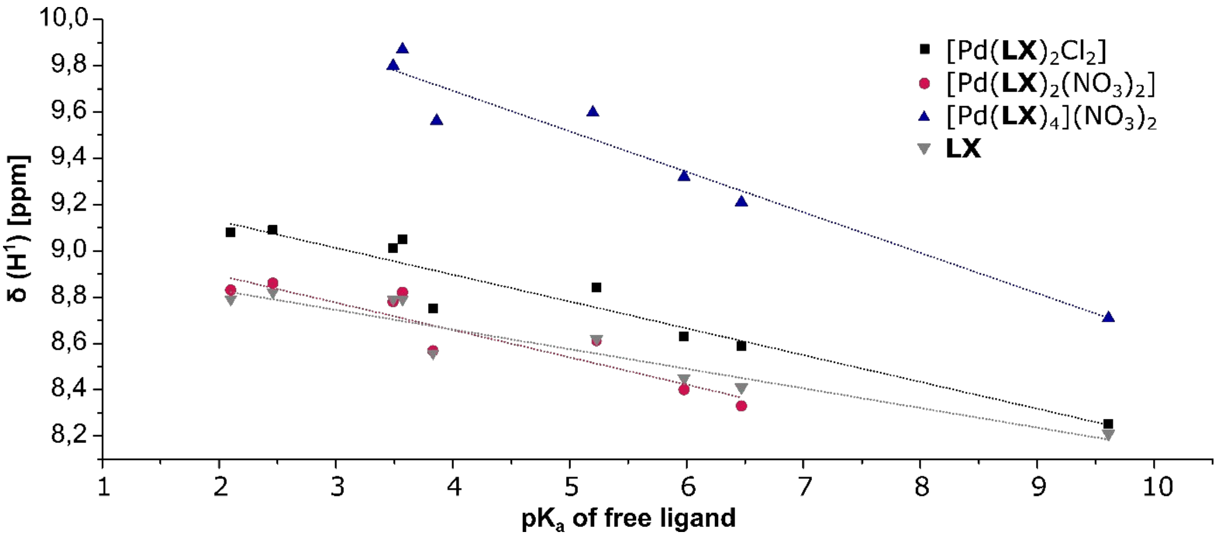

Figure S27. The relationship between chemical shifts (δ, ppm) of the signal H[1] in the ^{1}H NMR spectra ($CDCl_3$, 25°C) and the pK_a values of free ligands for complexes of the general formula: [Pd**L**$_2$Cl$_2$] (black line, slope = -0.1158, R^2 = 0.9196); [Pd**L**$_2$(NO$_3$)$_2$] (red line, slope = -0.1180, R^2 = 0.7991); [Pd**L**$_4$](NO$_3$)$_2$ (blue line, slope = -0.1751, R^2 = 0.9183) and free ligands **L** (grey line, slope = -0.0848, R^2 = 0.8412). Ligands of known pK_a values in the literature are included in the graph.

4.2. The relationship between chemical shift changes in the ^{1}H NMR spectra and basicity of free ligands

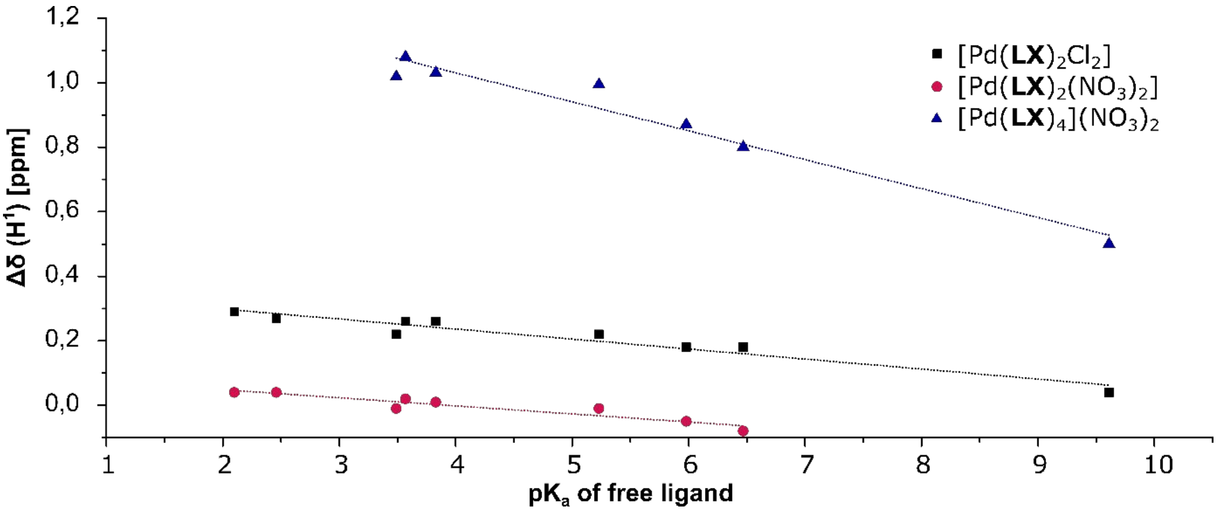

Figure S28. The relationship between chemical shift changes (Δδ, ppm) of the signal H[1] in the ^{1}H NMR spectra ($CDCl_3$, 25°C), where $\Delta\delta$ (H[1]) = $\delta_{complex} - \delta_{ligand}$ and the pK_a values of free ligands for complexes of the general formula: [Pd**L**$_2$Cl$_2$] (black line, slope = -0.0310, R^2 = 0.9320); [Pd**L**$_2$(NO$_3$)$_2$] (red line, slope = -0,0250, R^2 = 0.8856) and [Pd**L**$_4$](NO$_3$)$_2$ (blue line, slope = -0.0896, R^2 = 0.9430). Ligands of known pK_a values in the literature are included in the graph.

5. ^{1}H NMR titration of $[Pd(L2)_4](NO_3)_2$ with $Et_3N \cdot HCl$

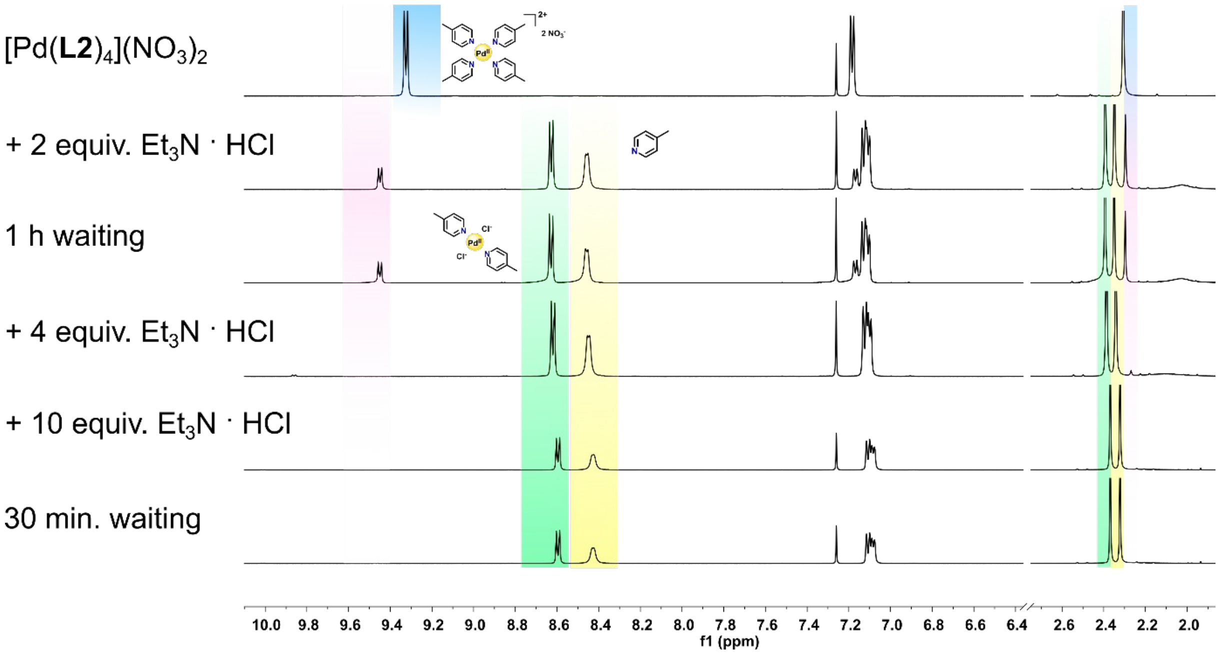

Figure S29. ^{1}H NMR (400 MHz, $CDCl_3$) titration spectra of $[Pd(\mathbf{L2})_4](NO_3)_2$ with $Et_3N \cdot HCl$.

6. ^{1}H NMR titration of $[Pd(L2)_2Cl_2]$ with L2

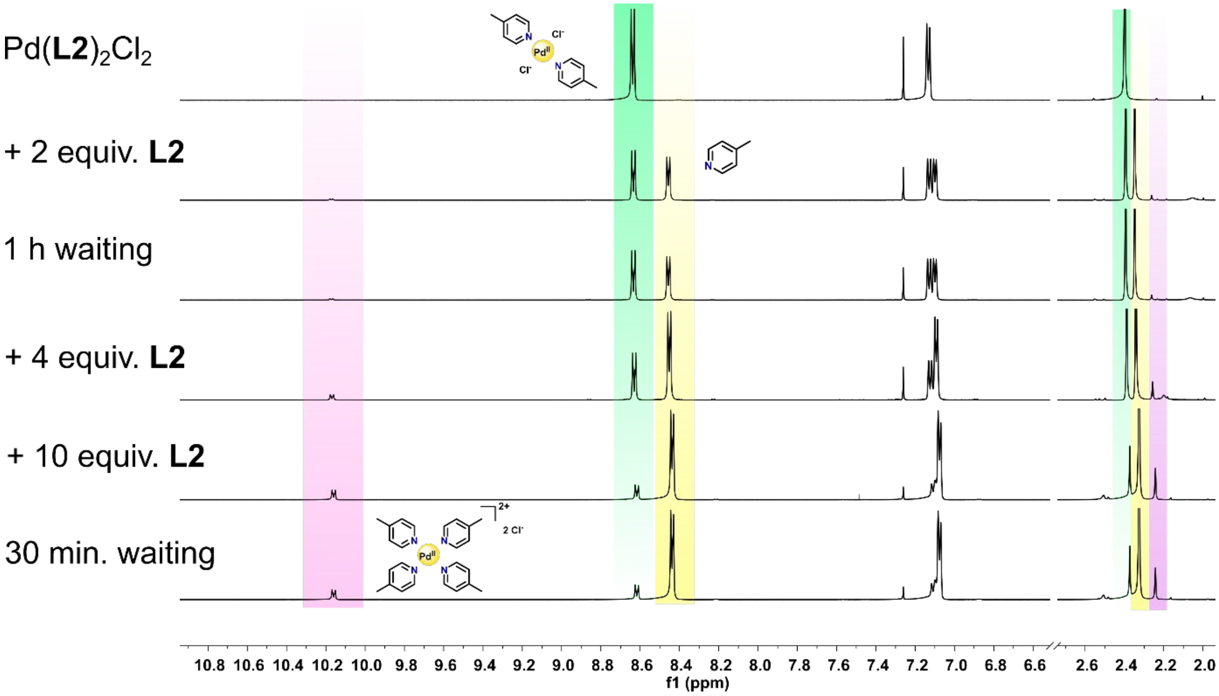

Figure S30. ^{1}H NMR (400 MHz, $CDCl_3$) titration spectra of $[Pd(\mathbf{L2})_2Cl_2]$ with **L2**.

7. Acid – base titrations of the Pd(II) complexes based on the ligand L2

7.1. ^{1}H NMR titration of [Pd(L2)$_2$Cl$_2$] with Et_3N

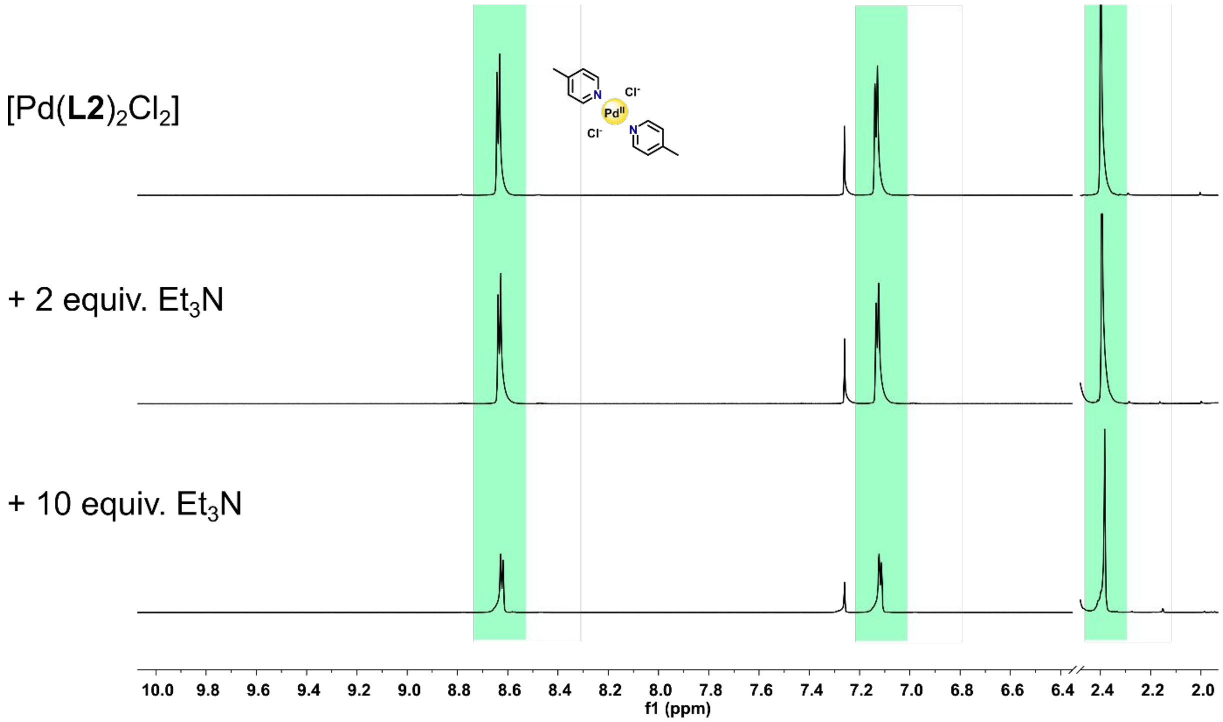

Figure S31. ^{1}H NMR (600 MHz, $CDCl_3$) titration spectra of [Pd(**L2**)$_2$Cl$_2$] upon addition of Et_3N.

7.2. ^{1}H NMR titration of [Pd(L2)$_2$Cl$_2$] with MSA

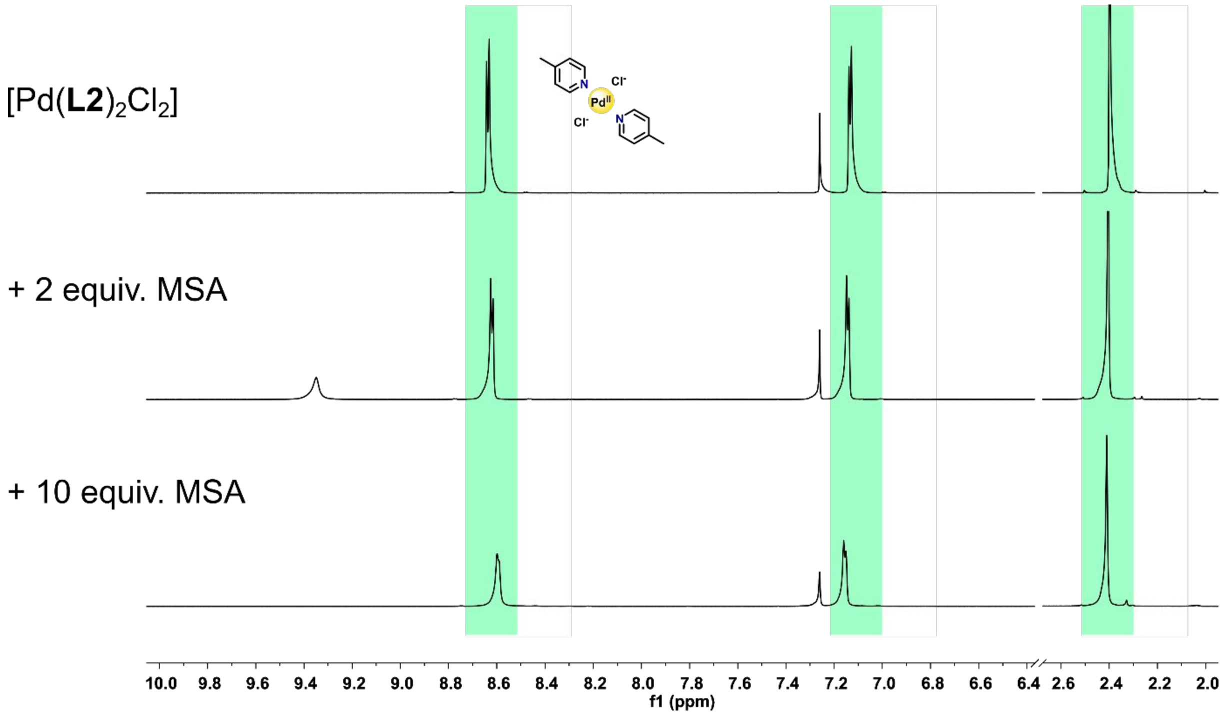

Figure S32. ^{1}H NMR (600 MHz, $CDCl_3$) titration spectra of [Pd(**L2**)$_2$Cl$_2$] upon addition of MSA.

7.3. ^{1}H NMR titration of [Pd(L2)$_2$(NO$_3$)$_2$] with Et$_3$N

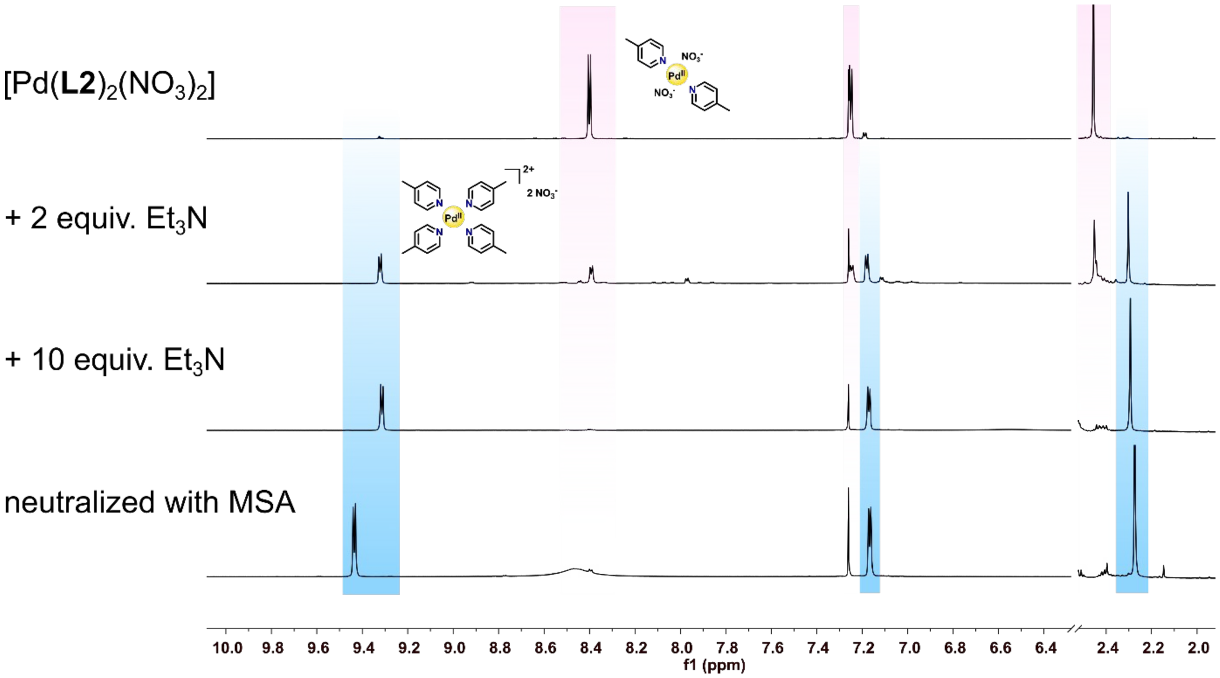

Figure S33. ^{1}H NMR (600 MHz, CDCl$_3$) titration spectra of [Pd(**L2**)$_2$(NO$_3$)$_2$] upon addition of Et$_3$N.

7.4. ^{1}H NMR titration of [Pd(L2)$_2$(NO$_3$)$_2$] with MSA

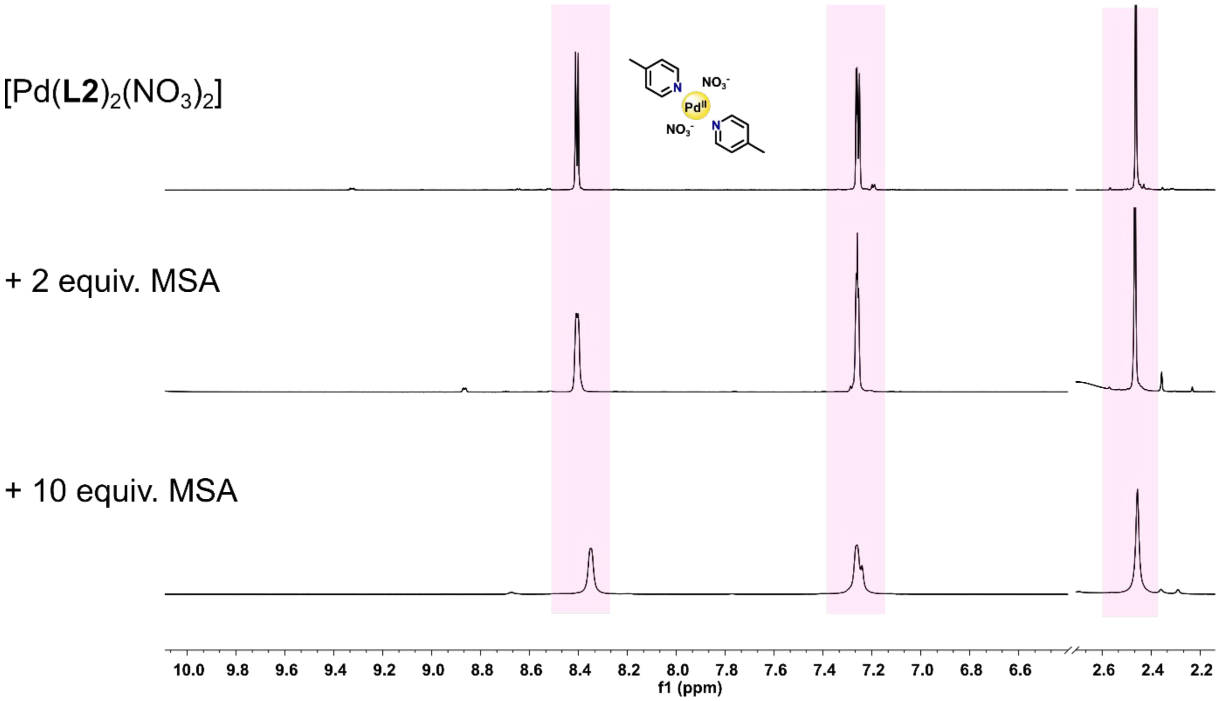

Figure S34. ^{1}H NMR (600 MHz, CDCl$_3$) titration spectra of [Pd(**L2**)$_2$(NO$_3$)$_2$] upon addition of MSA.

7.5. ^{1}H NMR titration of $[Pd(L2)_4](NO_3)_2$ with Et_3N

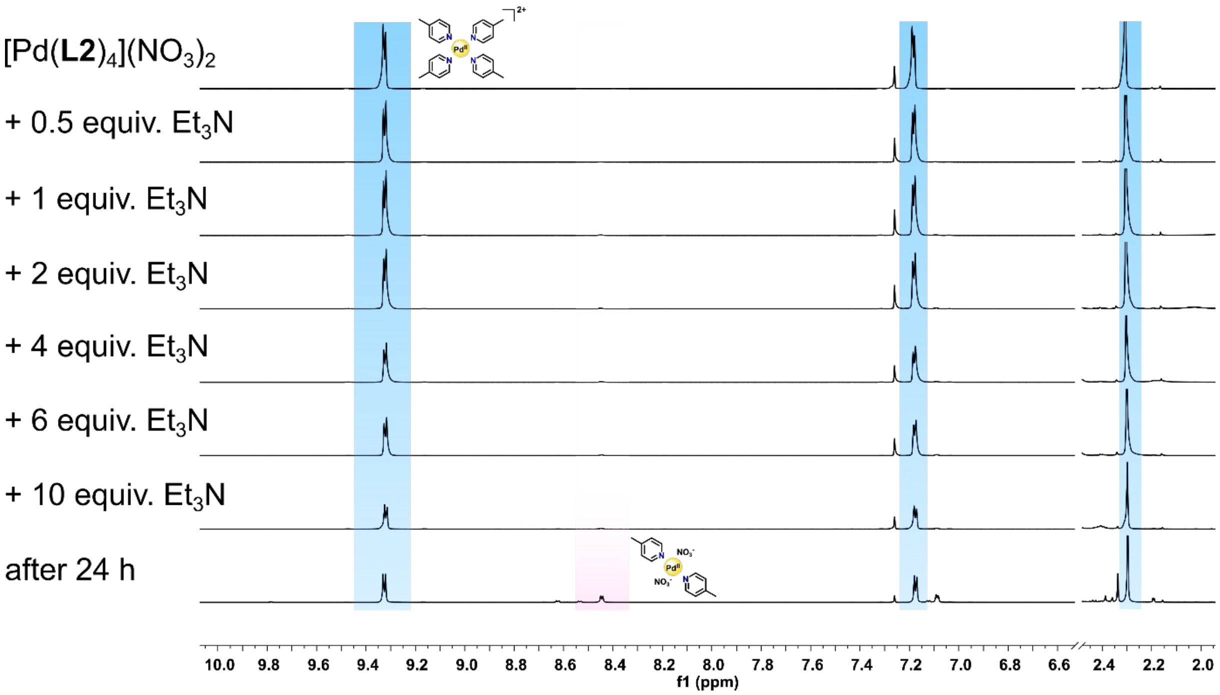

Figure S35. ^{1}H NMR (600 MHz, $CDCl_3$) titration spectra of $[Pd(\mathbf{L2})_4](NO_3)_2$ upon addition of different equivalents of Et_3N.

7.6. ^{1}H NMR titration of $[Pd(L2)_4](NO_3)_2$ with MSA

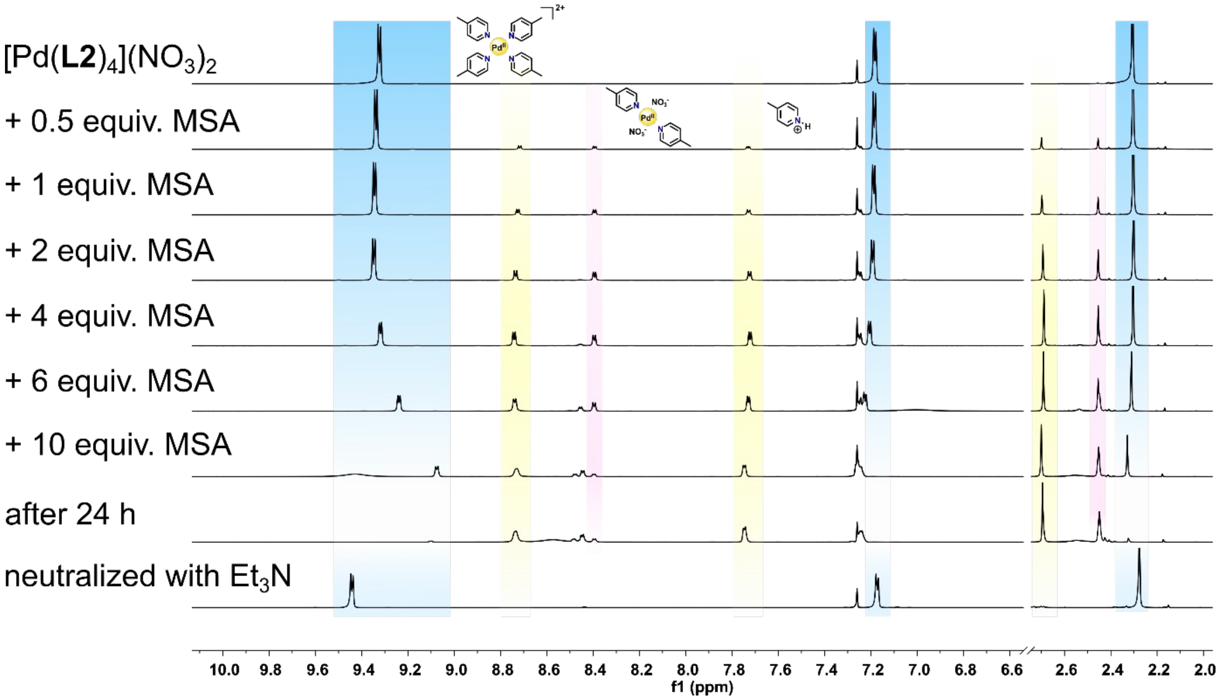

Figure S36. ^{1}H NMR (600 MHz, $CDCl_3$) titration spectra of $[Pd(\mathbf{L2})_4](NO_3)_2$ upon addition of different equivalents of MSA.

8. Stability investigation of Pd(II) complexes at high temperature in DMSO

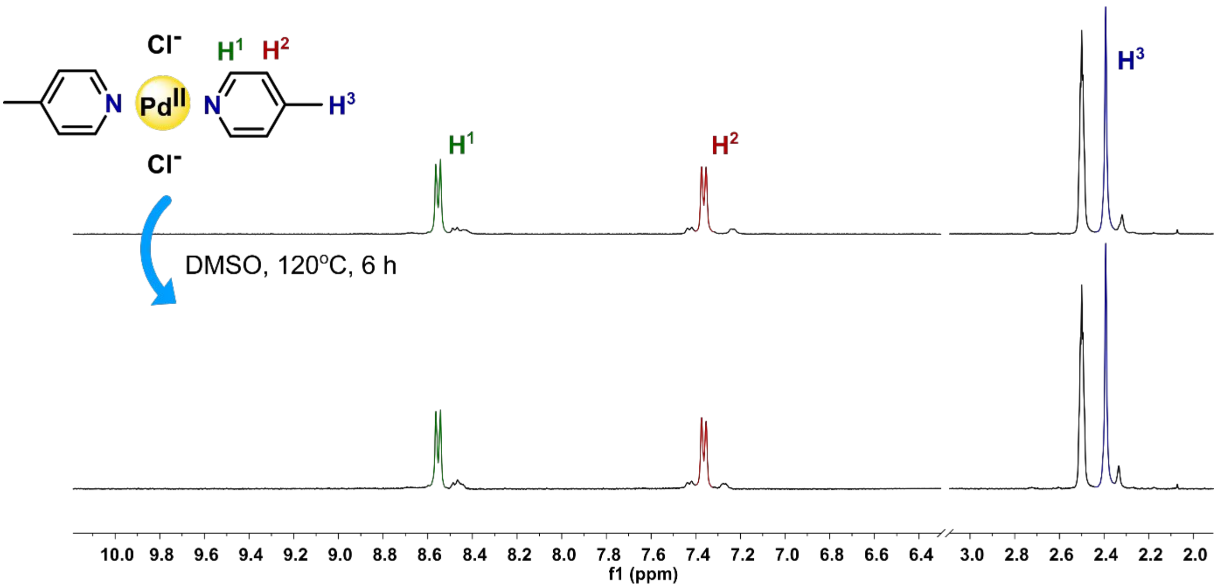

Figure S37. The ^{1}H NMR spectra (300 MHz, DMSO-d_6) showing the stability of of [Pd(**L2**)$_2$Cl$_2$] during heating in 120°C for 6 h.

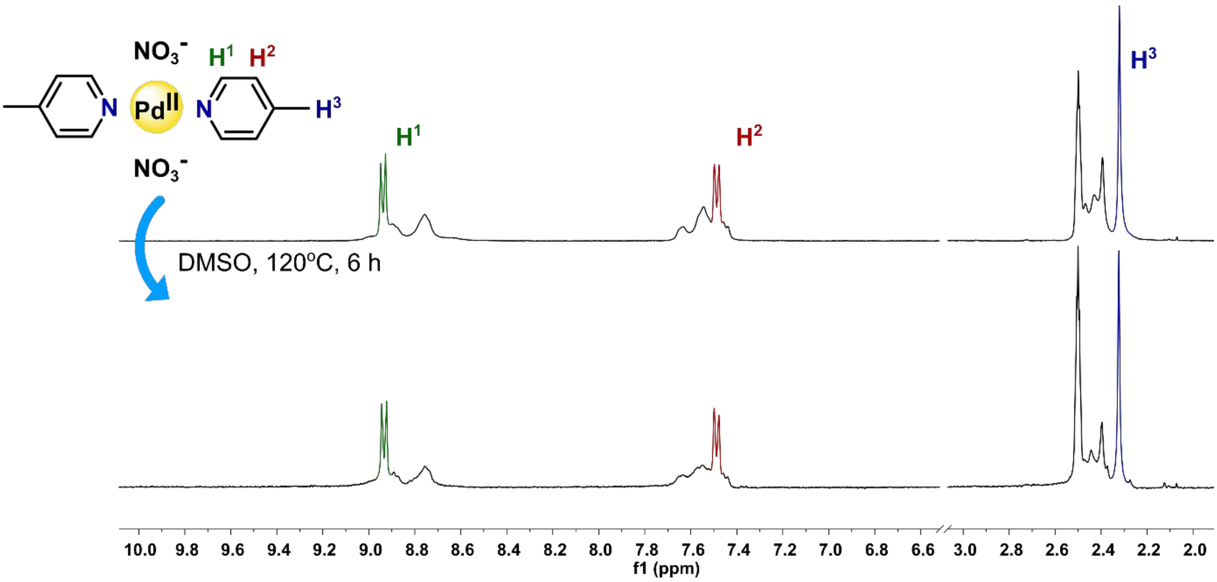

Figure S38. The ^{1}H NMR spectra (300 MHz, DMSO-d_6) showing the stability of of [Pd(**L2**)$_2$(NO$_3$)$_2$] during heating in 120°C for 6 h.

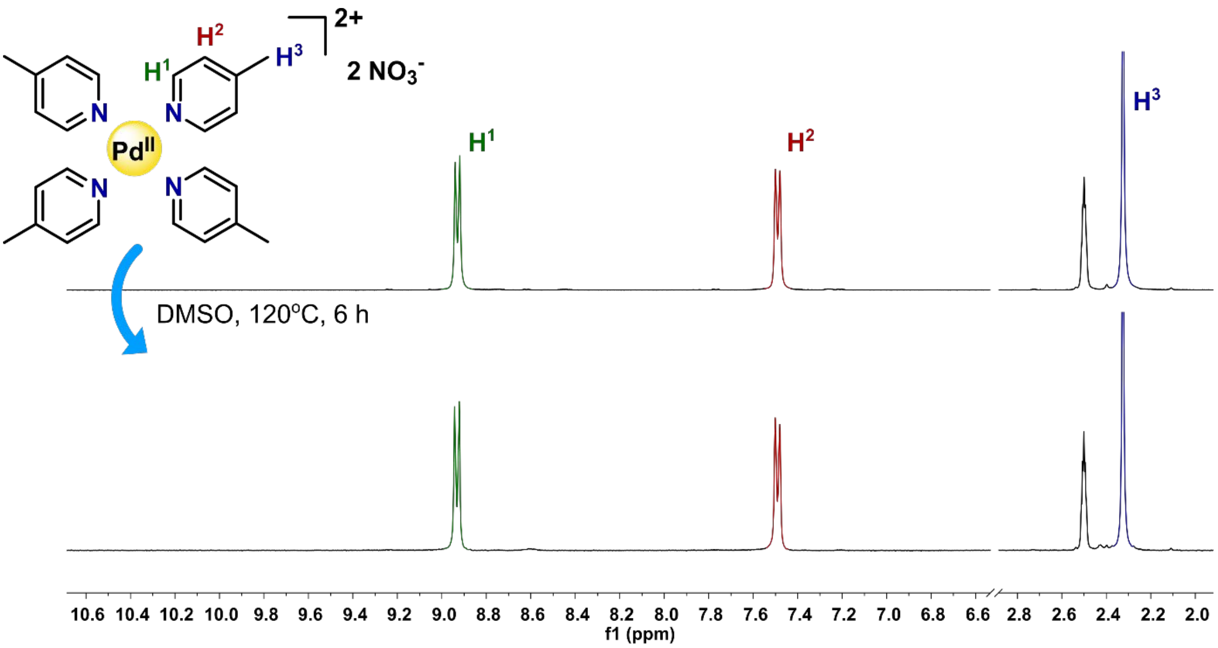

Figure S39. The ^{1}H NMR spectra (300 MHz, DMSO-d_6) showing the stability of of [Pd(**L2**)$_4$](NO_3)$_2$ during heating in 120°C for 6 h.

9. X-ray crystal structure analysis

9.1. Additional details for crystal structure solution and refinement

The crystal of [Pd(**L3**)$_2$Cl$_2$] was recognized as a non-merohedral twin. Twin law [1 0 0 0.74 -1 0 0.17 0 -1], which correspond to a 2-fold rotation axis around [100] direct lattice direction, was determined using ROTAX. The "Make HKLF5" function in WinGX was used to convert the reflection data to the HKLF5 format.[6] The refinement process took into account reflections belonging from the larger domain that are not overlapping with reflections from the smaller domain. The twin fraction was refined at 0.480(6). The chosen crystal of [Pd(**L4**)$_2$Cl$_2$] used for X-ray measurement was also identified as a non-merohedral twin. ROTAX suggested a possible twin matrix [1 0 0 0 -1 0 -1 0 -1], corresponding to 180° rotation about [-201] reciprocal lattice direction. The refinement process was carried out in the same way as in [Pd(**L3**)$_2$Cl$_2$] and the BASF parameter was refined at 0.0695(9).

In [Pd(**L2**)$_4$](NO_3)$_2$ the O2, O2A, O3, and O3A oxygen atoms in nitrate ion are disordered over 2 positions with fixed occupancies at 0.5. Atoms belonging to the –COOMe group near the N2-pyridyl ring in the [Pd(**L4**)$_4$](NO_3)$_2$ crystal structure are disordered over two positions and refined at fixed occupancies of 0.5. In [Pd(**L7**)$_4$](NO_3)$_2$, the N4-pyridyl ring is disordered over two positions with occupancies constrained at 0.5. Two chloroform molecules are disordered. In one solvent molecule (C14 and C14A) all atoms are disordered, and in the other molecule (with the C15 carbon atom) chlorine atoms show disorder. The occupancies of the disordered atoms were refined with occupancies constrained at 0.5. A solvent molecule in the asymmetric unit of [Pd(**L6**)$_4$](NO_3)$_2$ could not be modeled satisfactorily and it was therefore removed from the electron density map using the solvent mask within Olex2.[7]

9.2. Description of the X-ray structure of Pd(II) complexes

Table S14. Crystal data and structure refinement for the complexes based on **L2** and **L3**.

	[Pd(**L2**)$_2$(NO3)$_2$]	[Pd(**L2**)$_4$](NO3)$_2$	[Pd(**L3**)$_2$(NO3)$_2$]	[Pd(**L3**)$_2$Cl$_2$]
CCDC deposit no.	2175523	2175530	2175524	2175521
Empirical formula	$C_{12}H_{14}N_4O_6Pd$	$C_{24}H_{28}N_6O_6Pd$	$C_{12}H_{14}N_4O_8Pd$	$C_{12}H_{14}Cl_2N_2O_2Pd$
Formula weight	416.67	602.92	448.67	395.55
Temperature/K	100.02(10)	293(2)	100.02(10)	131.8(2)
Crystal system	monoclinic	monoclinic	orthorhombic	triclinic
Space group	$P2_1/n$	$C2/c$	$P2_12_12_1$	P-1
a/Å	7.9260(4)	18.5050(2)	8.3401(2)	3.9848(2)
b/Å	18.0008(9)	10.3994(1)	12.6397(4)	8.2533(7)
c/Å	10.4840(5)	15.8150(2)	15.3354(5)	11.0993(7)
α/°	90	90	90	76.721(6)
β/°	91.228(4)	112.4830(10)	90	88.299(5)
γ/°	90	90	90	79.746(6)
V/Å^3	1495.46(12)	2812.13(6)	1616.60(8)	349.57(4)
Z, Z’	4, 1	4, 0.5	4, 1	1, 0.5
ρ_{calc}/gcm^{-3}	1.851	1.424	1.843	1.879
μ/mm^{-1}	1.279	5.723	1.199	14.224
F(000)	832	1232	896	196
Crystal size/mm^3	0.67 × 0.46 × 0.40	0.24 × 0.20 × 0.13	0.55 × 0.28 × 0.20	0.87 × 0.05 × 0.05
Radiation/Å	Mo Kα (λ = 0.71073)	Cu Kα (λ = 1.54184)	Mo Kα (λ = 0.71073)	Cu Kα (λ = 1.54184)
2θ range/°	6.378 to 56.376	9.956 to 152.71	6.214 to 57.392	8.186 to 152.932
Index ranges	-10 ≤ h ≤ 9<br>-14 ≤ k ≤ 23<br>-13 ≤ l ≤ 13	-23 ≤ h ≤ 22<br>-13 ≤ k ≤ 13<br>-19 ≤ l ≤ 17	-10 ≤ h ≤ 6<br>-10 ≤ k ≤ 16<br>-20 ≤ l ≤ 10	-4 ≤ h ≤ 4<br>-10 ≤ k ≤ 10<br>-13 ≤ l ≤ 13
Reflections collected	6095	15962	4615	2240
Independent reflections	3140 [R_{int} = 0.0183, R_{sigma} = 0.0299]	2935 [R_{int} = 0.0179, R_{sigma} = 0.0104]	3253 [R_{int} = 0.0236, R_{sigma} = 0.0517]	1402 [R_{sigma} = 0.0337]
Reflections with I ≥ 2σ (I)	2866	2740	3067	1386
Data/restraints/parameters	3140/0/210	2935/0/189	3253/0/228	1402/0/118
Final R indexes [I ≥ 2σ (I)]	R_1 = 0.0330<br>wR_2 = 0.0795	R_1 = 0.0291<br>wR_2 = 0.0849	R_1 = 0.0354<br>wR_2 = 0.0807	R_1 = 0.0436<br>wR_2 = 0.1350
Final R indexes (all data)	R_1 = 0.0376<br>wR_2 = 0.0816	R_1 = 0.0304<br>wR_2 = 0.0865	R_1 = 0.0387<br>wR_2 = 0.0829	R_1 = 0.0439<br>wR_2 = 0.1354
Goodness-of-fit on F^2	1.152	1.101	1.040	1.234
Largest diff. peak/hole/eÅ^{-3}	1.09/-0.72	0.66/-0.46	2.27/-0.73	1.38/-1.67

Table S15. Crystal data and structure refinement for the complexes based on **L4** and **L5**.

	[Pd(**L4**)$_2$(NO3)$_2$]	[Pd(**L4**)$_2$Cl$_2$]	[Pd(**L4**)$_4$](NO3)$_2$	[Pd(**L5**)$_2$(NO3)$_2$]
CCDC deposit no.	2175531	2175522	2175532	2175528
Empirical formula	$C_{28}H_{28}N_8O_{20}Pd_2 \cdot CHCl_3$	$C_{14}H_{14}N_2O_4Cl_2Pd$	$C_{28}H_{28}N_6O_{14}Pd \cdot H_2O$	$C_{14}H_{14}N_4O_8Pd$
Formula weight	1128.75	451.57	796.98	472.69
Temperature/K	100.02(10)	100.01(10)	293(2)	293(2)
Crystal system	triclinic	monoclinic	triclinic	monoclinic
Space group	*P*-1	*P*2$_1$/*c*	*P*-1	*P*2$_1$/*c*
a/Å	11.6543(4)	3.8243(2)	10.0018(1)	11.1872(4)
b/Å	11.6963(4)	9.6992(4)	10.2197(2)	7.4905(2)
c/Å	17.0748(6)	21.1957(8)	18.8404(4)	10.8438(3)
α/°	91.138(3)	90	103.607(2)	90
β/°	98.120(3)	94.855(4)	90.370(1)	93.037(3)
γ/°	119.739(4)	90	116.196(2)	90
V/Å^3	1989.72(14)	783.39(5)	1665.99(6)	907.42(5)
Z, Z'	2, 1	2, 0.5	2, 1	2, 0.5
ρ_{calc}/gcm^{-3}	1.884	1.914	1.589	1.730
μ/mm^{-1}	1.197	12.895	0.637	8.726
F(000)	1124	448	812	472
Crystal size/mm^3	0.43 × 0.43 × 0.36	0.08 × 0.04 × 0.03	0.20 × 0.10 × 0.01	0.85 × 0.31 × 0.11
Radiation/Å	Mo Kα (λ = 0.71073)	Cu Kα (λ = 1.54184)	Mo Kα (λ = 0.71073)	Cu Kα (λ = 1.54184)
2θ range/°	5.772 to 58.354	8.374 to 150.814	4.558 to 55.468	7.914 to 152.288
Index ranges	-15 ≤ h ≤ 14<br>-16 ≤ k ≤ 15<br>-21 ≤ l ≤ 22	-4 ≤ h ≤ 4<br>-12 ≤ k ≤ 12<br>-26 ≤ l ≤ 26	-12 ≤ h ≤ 12<br>-13 ≤ k ≤ 13<br>-24 ≤ l ≤ 24	-13 ≤ h ≤ 13<br>-8 ≤ k ≤ 9<br>-11 ≤ l ≤ 13
Reflections collected	26178	4741	100939	3615
Independent reflections	9330 [R_{int} = 0.0260, R_{sigma} = 0.0342]	1532 [R_{sigma} = 0.0260]	7223 [R_{int} = 0.0590, R_{sigma} = 0.0355]	1846 [R_{int} = 0.0284, R_{sigma} = 0.0275]
Reflections with I ≥ 2σ (I)	7930	1452	6111	1606
Data/restraints/parameters	9330/0/566	1532/0/108	7223/0/486	1846/0/125
Final R indexes [I ≥ 2σ (I)]	R_1 = 0.0277<br>wR_2 = 0.0596	R_1 = 0.0367<br>wR_2 = 0.1044	R_1 = 0.0501<br>wR_2 = 0.1181	R_1 = 0.0563<br>wR_2 = 0.1835
Final R indexes (all data)	R_1 = 0.0371<br>wR_2 = 0.0632	R_1 = 0.0382<br>wR_2 = 0.1057	R_1 = 0.0670<br>wR_2 = 0.1241	R_1 = 0.0596<br>wR_2 = 0.1918
Goodness-of-fit on F^2	1.024	1.140	1.204	1.162

Largest diff. peak/hole/eÅ^{-3}	0.80/-0.60	0.85/-1.22	0.82/-0.56	2.65/-1.33

Table S16. Crystal data and structure refinement for the complexes based on **L6**.

	[Pd(**L6**)$_2$Cl$_2$]	[Pd(**L6**)$_4$](NO3)$_2$
CCDC deposit no.	2175520	2175527
Empirical formula	$C_{14}H_{20}Cl_2N_4Pd\cdot 2(CHCl_3)$	$C_{28}H_{40}N_{10}O_6Pd$
Formula weight	660.37	719.10
Temperature/K	134.4(10)	100.02(10)
Crystal system	triclinic	triclinic
Space group	*P*-1	*P*-1
a/Å	7.1778(5)	10.7739(8)
b/Å	8.8620(8)	10.8544(10)
c/Å	10.8410(7)	11.2702(13)
α/°	66.812(7)	64.868(11)
β/°	82.991(6)	63.701(9)
γ/°	88.432(7)	67.351(8)
V/Å^3	628.99(9)	1037.7(2)
Z, Z’	1, 0.5	1, 0.5
ρ_{calc}/gcm^{-3}	1.743	1.151
μ/mm^{-1}	13.874	0.491
F(000)	328	372
Crystal size/mm^3	0.36 × 0.25 × 0.07	0.65 × 0.55 × 0.33
Radiation/Å	Cu Kα (λ = 1.54184)	Mo Kα (λ = 0.71073)
2θ range/°	8.94 to 153.152	6.796 to 56.696
Index ranges	-9 ≤ h ≤ 8<br>-11 ≤ k ≤ 10<br>-12 ≤ l ≤ 13	-13 ≤ h ≤ 13<br>-8 ≤ k ≤ 14<br>-14 ≤ l ≤ 14
Reflections collected	4492	7682
Independent reflections	2545 [R_{int} = 0.0277, R_{sigma} = 0.0311]	4374 [R_{int} = 0.0356, R_{sigma} = 0.0649]
Reflections with I ≥ 2σ (I)	2468	3916
Data/restraints/parameters	2545/0/136	4374/0/209
Final R indexes [I ≥ 2σ (I)]	R_1 = 0.0334<br>wR_2 = 0.0898	R_1 = 0.0567<br>wR_2 = 0.1439
Final R indexes (all data)	R_1 = 0.0342<br>wR_2 = 0.0907	R_1 = 0.0637<br>wR_2 = 0.1500
Goodness-of-fit on F^2	1.059	1.057
Largest diff. peak/hole/eÅ^{-3}	0.95/-1.40	1.56/-0.97

Table S17. Crystal data and structure refinement for the complexes based on **L7**.

	[Pd(**L7**)$_2$Cl$_2$]	[Pd(**L7**)$_2$(NO3)$_2$]	[Pd(**L7**)$_4$](NO3)$_2$
CCDC deposit no.	2175525	2175526	2175529
Empirical formula	$C_{10}H_8Cl_4N_2Pd$	$C_{10}H_8N_4O_6Cl_2Pd$	$C_{20}H_{16}Cl_4N_6O_6Pd\cdot 2(CHCl_3)$
Formula weight	404.38	457.50	923.32
Temperature/K	100.01(10)	293(2)	100.01(10)
Crystal system	triclinic	triclinic	triclinic
Space group	*P*-1	*P*-1	*P*-1
a/Å	5.3591(3)	7.8530(3)	10.4344(6)

b/Å	6.9072(5)	10.0686(4)	10.8794(5)
c/Å	9.3915(8)	10.5749(3)	16.5985(7)
α/°	75.523(7)	64.407(3)	98.527(3)
β/°	84.632(6)	88.470(3)	98.958(4)
γ/°	83.510(6)	80.253(3)	106.572(4)
V/Å^3	333.67(4)	742.21(5)	1746.63(15)
Z, Z'	1, 0.5	2, 1	2, 1[a]
ρ_{calc}/gcm^{-3}	2.012	2.047	1.756
μ/mm^{-1}	2.167	1.646	1.342
F(000)	196	448	912
Crystal size/mm^3	0.23 × 0.10 × 0.08	0.34 × 0.26 × 0.16	0.22 × 0.15 × 0.13
Radiation/Å	Mo Kα (λ = 0.71073)	Mo Kα (λ = 0.71073)	Mo Kα (λ = 0.71073)
2θ range/°	6.118 to 74.902	6.614 to 71.53	5.432 to 57.054
Index ranges	-9 ≤ h ≤ 9<br>-8 ≤ k ≤ 11<br>-16 ≤ l ≤ 15	-12 ≤ h ≤ 12<br>-16 ≤ k ≤ 16<br>-17 ≤ l ≤ 16	-13 ≤ h ≤ 8<br>-14 ≤ k ≤ 14<br>-21 ≤ l ≤ 20
Reflections collected	5745	12121	13222
Independent reflections	3328 [R_{int} = 0.0202, R_{sigma} = 0.0377]	6497 [R_{int} = 0.0223, R_{sigma} = 0.0398]	7429 [R_{int} = 0.0250, R_{sigma} = 0.0477]
Reflections with I ≥ 2σ (I)	3050	5053	6001
Data/restraints/parameters	3328/0/96	6497/0/241	7429/38/517
Final R indexes [I ≥ 2σ (I)]	R_1 = 0.0253<br>wR_2 = 0.0526	R_1 = 0.0368<br>wR_2 = 0.0717	R_1 = 0.0354<br>wR_2 = 0.0638
Final R indexes (all data)	R_1 = 0.0295<br>wR_2 = 0.0546	R_1 = 0.0577<br>wR_2 = 0.0823	R_1 = 0.0515<br>wR_2 = 0.0698
Goodness-of-fit on F^2	1.057	1.047	1.022
Largest diff. peak/hole/eÅ^{-3}	0.66/-1.42	0.75/-1.18	0.67/-0.86

[a] Two halves of complex molecules in the asymmetric unit.

9.3. ORTEP representations of Pd(II) complexes

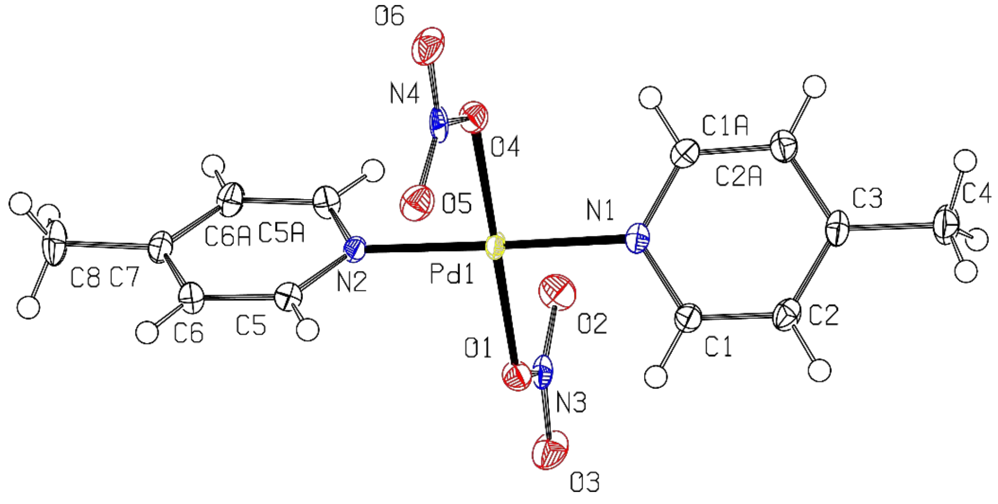

Figure S40. A view of the [Pd(**L2**)$_2$(NO_3)$_2$] asymmetric unit, showing the atom-labelling scheme. Displacement ellipsoids are drawn at the 50% probability level.

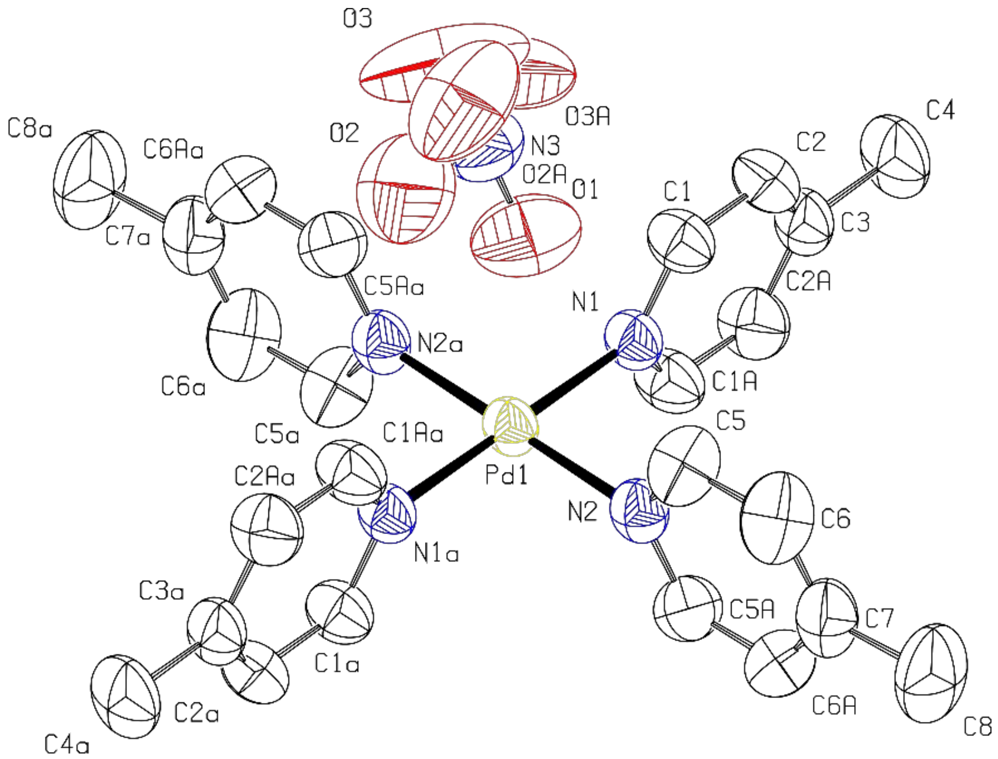

Figure S41. A view of the [Pd(**L2**)$_4$](NO_3)$_2$ structure, showing the atom-labelling scheme. Displacement ellipsoids are drawn at the 30% probability level. H atoms are omitted for clarity. In nitrate counterion the O2, O2A, O3, and O3A oxygen atoms are disordered over 2 positions with fixed occupancies at 0.5. Symmetry code: *(a)* 1−x, 1−y, 1−z.

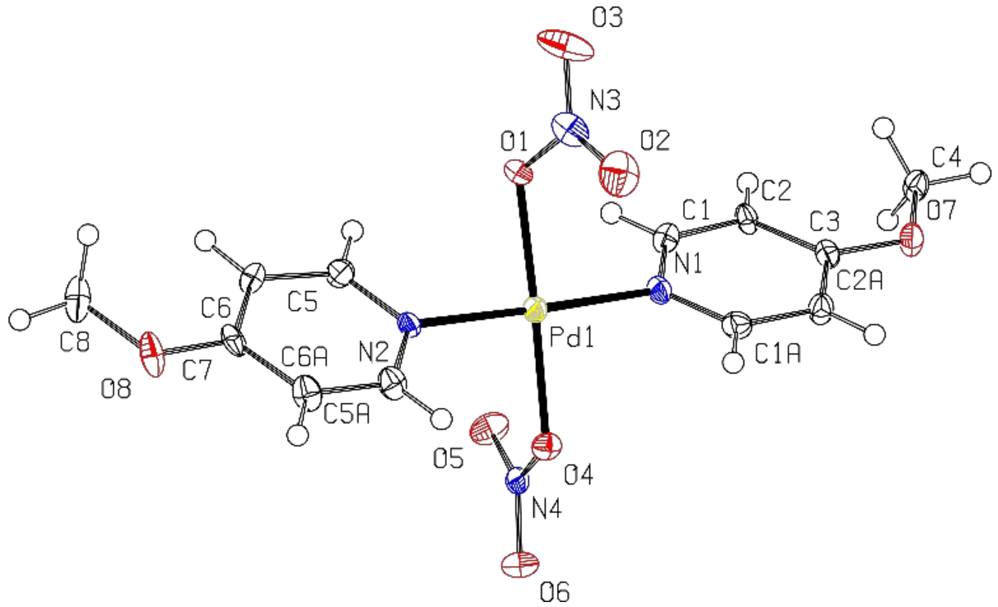

Figure S42. A view of the [Pd(**L3**)$_2$(NO_3)$_2$] asymmetric unit, showing the atom-labelling scheme. Displacement ellipsoids are drawn at the 50% probability level.

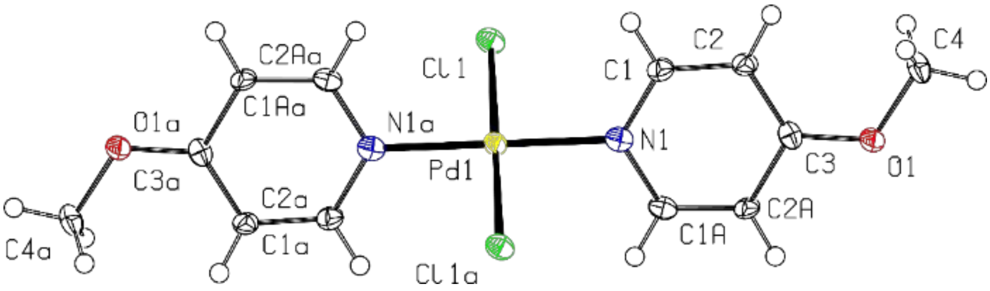

Figure S43. A view of the structure of [Pd(**L3**)$_2$Cl$_2$], showing the atom-labelling scheme. Displacement ellipsoids are drawn at the 50% probability level. Symmetry code: *(a)* 1−x, −y, 1−z.

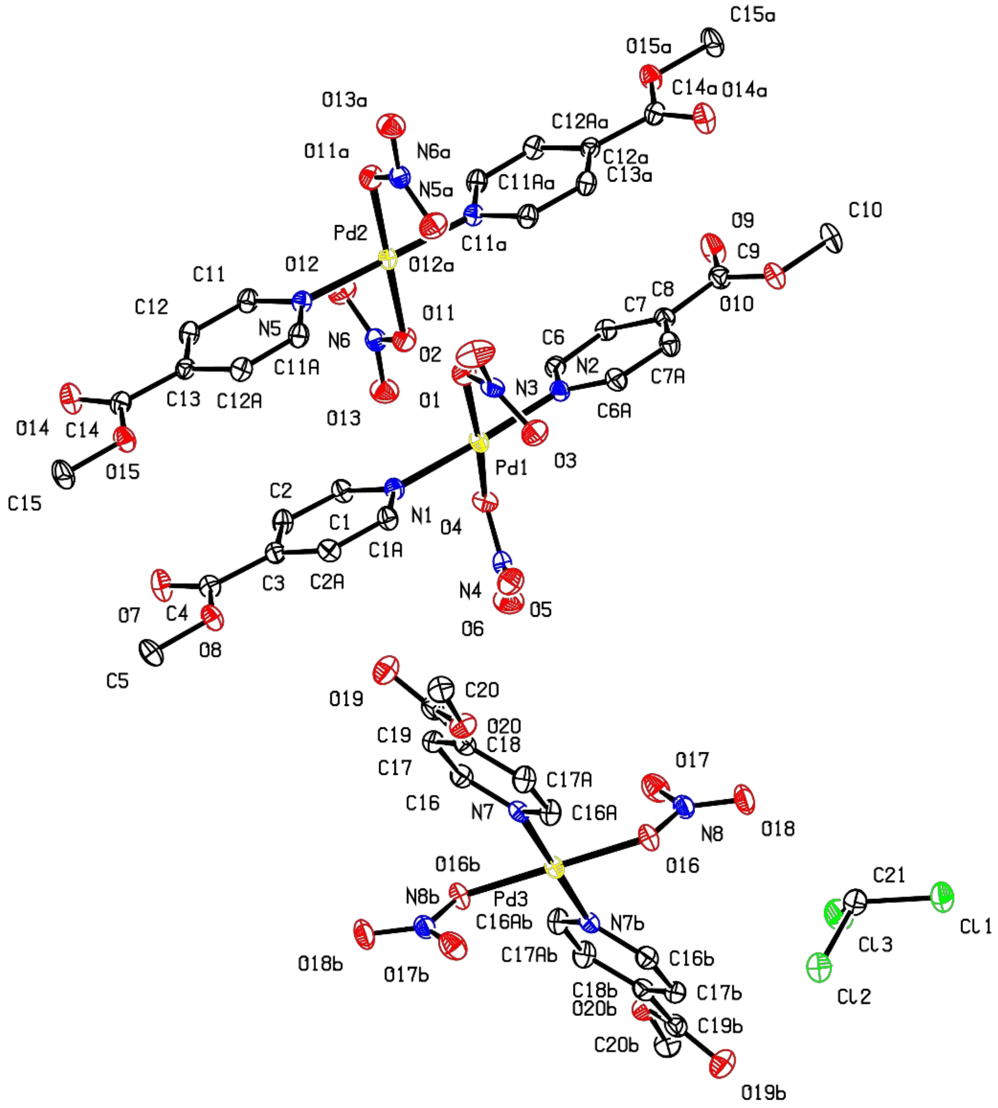

Figure S44. A view of the fragment of [Pd(**L4**)$_2$(NO$_3$)$_2$] crystal structure, showing the atom-labelling scheme. Displacement ellipsoids are drawn at the 50% probability level. H atoms are omitted for clarity. Symmetry codes: *(a)* 2−x, 1−y, 1−z; *(b)* − x, 1−y, −z.

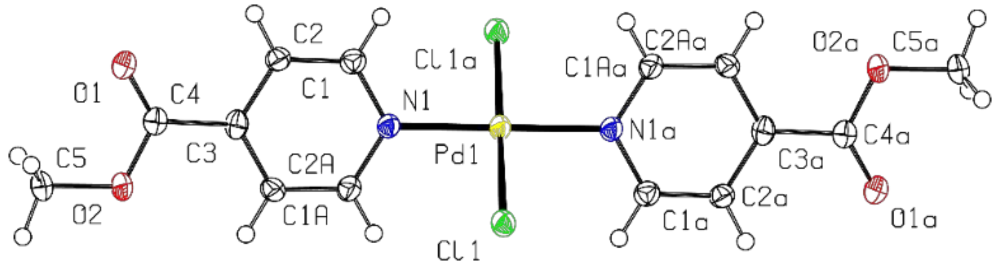

Figure S45. A view of the [Pd(**L4**)$_2$Cl$_2$] complex molecule with the atom-labelling scheme. Displacement ellipsoids are drawn at the 50% probability level. Symmetry code: *(a)* −x, 1−y, −z.

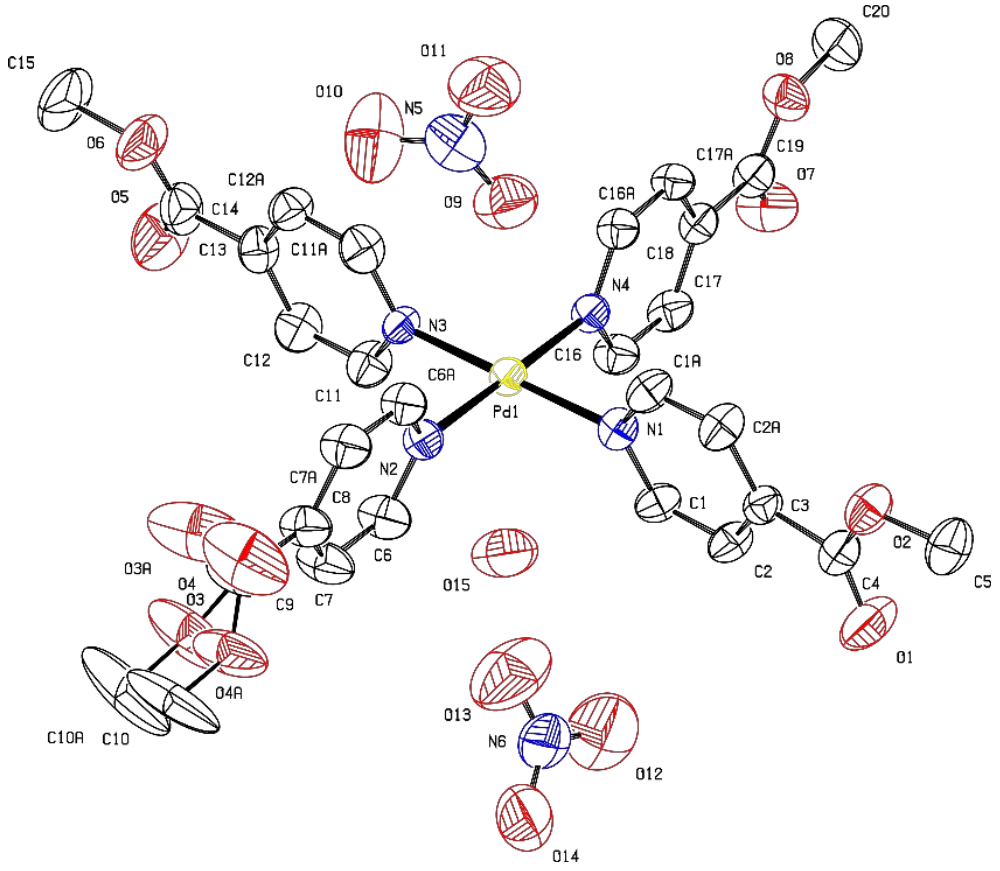

Figure S46. A view of the [Pd(**L4**)$_4$](NO$_3$)$_2$ asymmetric unit, showing the atom-labelling scheme. Displacement ellipsoids are drawn at the 50% probability level. H atoms are omitted for clarity. The -COOMe group near the N2-pyridyl ring are disordered over two positions and refined at fixed occupancies of 0.5.

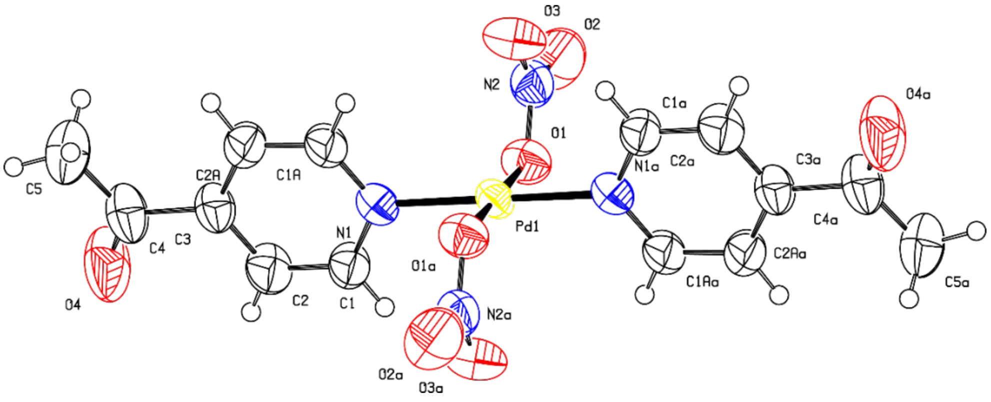

Figure S47. A view of the structure of [Pd(**L5**)$_2$(NO$_3$)$_2$], showing the atom-labelling scheme. Displacement ellipsoids are drawn at the 50% probability level. Symmetry code: *(a)* 1−x, 2−y, 1−z.

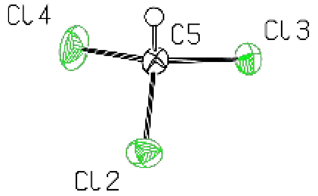

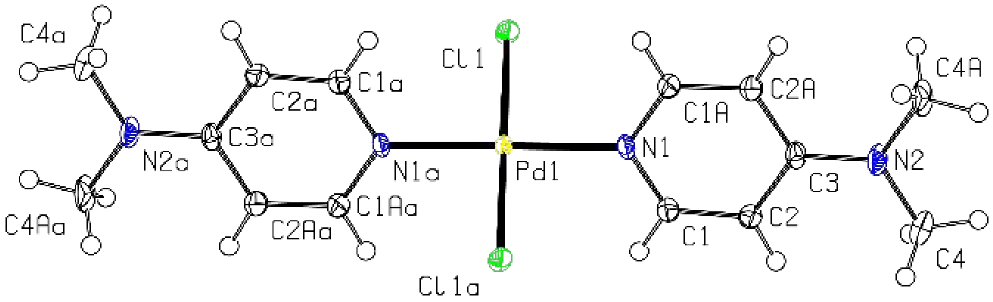

Figure S48. A view of the [Pd(**L6**)$_2$Cl$_2$] structure, showing the atom-labelling scheme. Displacement ellipsoids are drawn at the 50% probability level. Symmetry code: *(a)* 2−x, 2−y, −z.

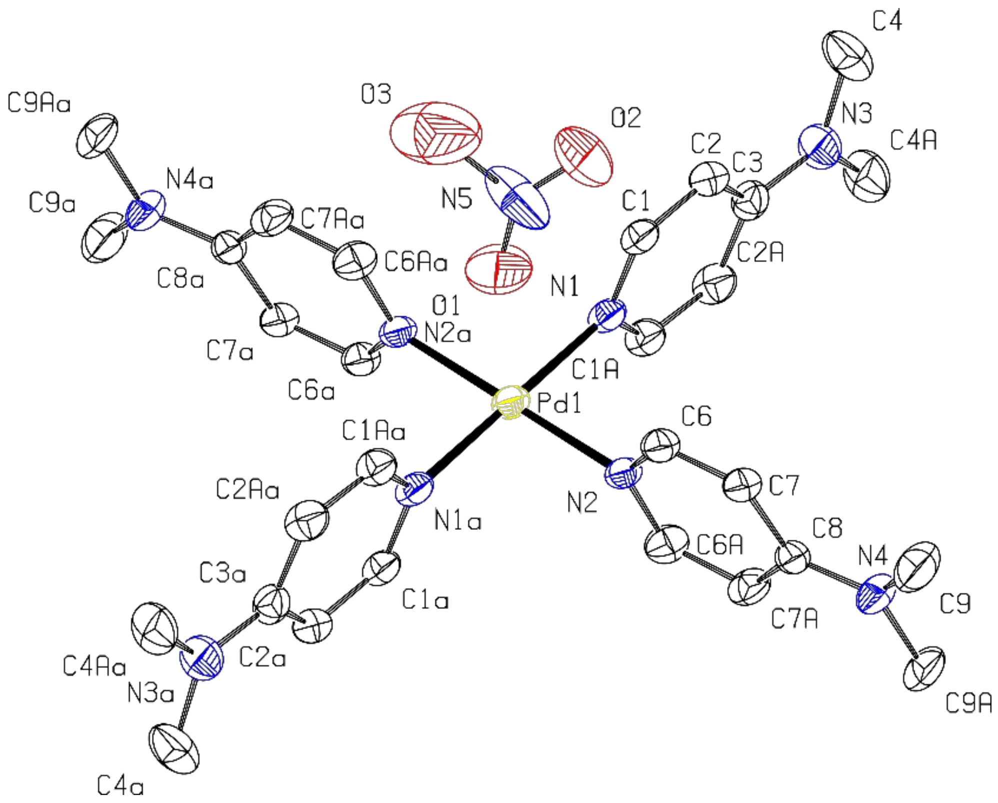

Figure S49. A view of the structure of $[Pd(\mathbf{L6})_4](NO_3)_2$ with the atom-labelling scheme. Displacement ellipsoids are drawn at the 50% probability level. H atoms are omitted for clarity. Symmetry code: *(a)* 1−x, 1−y, 1−z. Solvent molecules were not modelled.

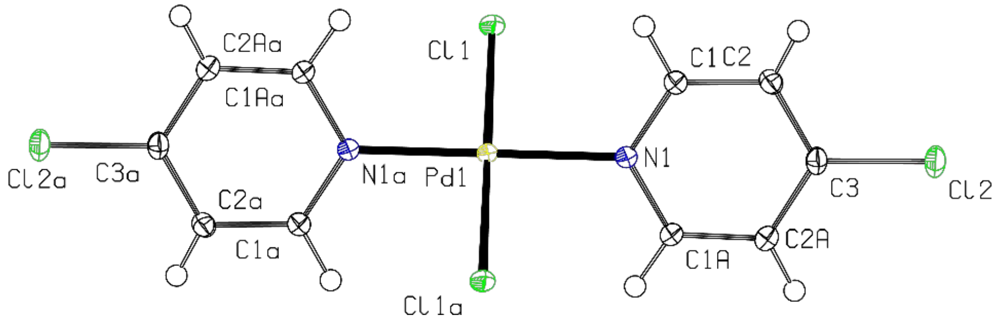

Figure S50. A view of the $[Pd(\mathbf{L7})_2Cl_2]$ complex molecule, showing the atom-labelling scheme. Displacement ellipsoids are drawn at the 50% probability level. Symmetry code: *(a)* −x, 1−y, 1−z.

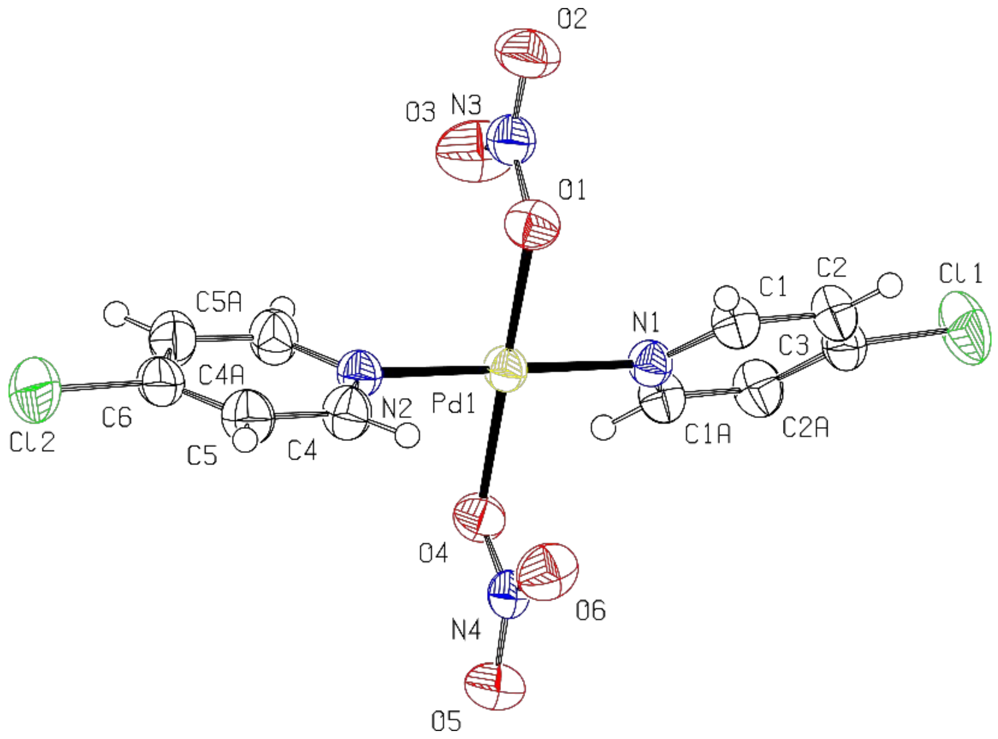

Figure S51. A view of the asymmetric unit of $[Pd(\mathbf{L7})_2(NO_3)_2]$ with the atom-labelling scheme. Displacement ellipsoids are drawn at the 50% probability level.

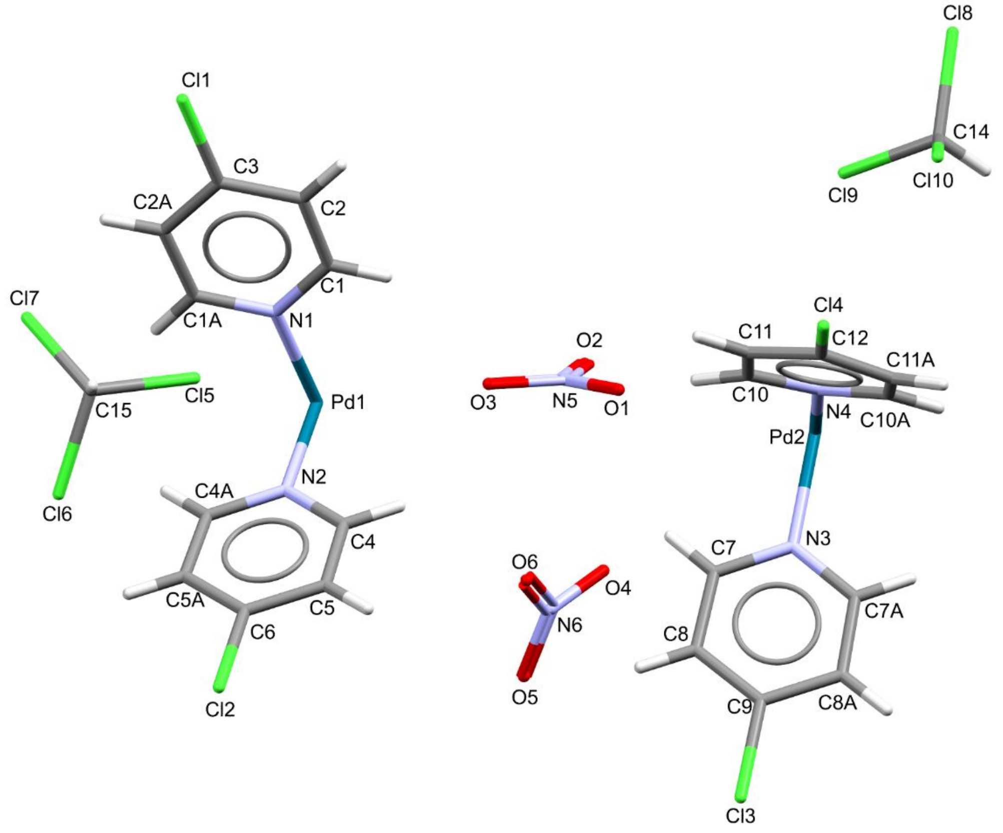

Figure S52. The asymmetric unit of the $[Pd(\mathbf{L7})_4](NO_3)_2{\cdot}2CHCl_3$ with the atom-labelling scheme. Displacement ellipsoids are drawn at the 50% probability level. The disordered fragments are omitted for clarity.

9.4. Weak interactions in the crystal structures

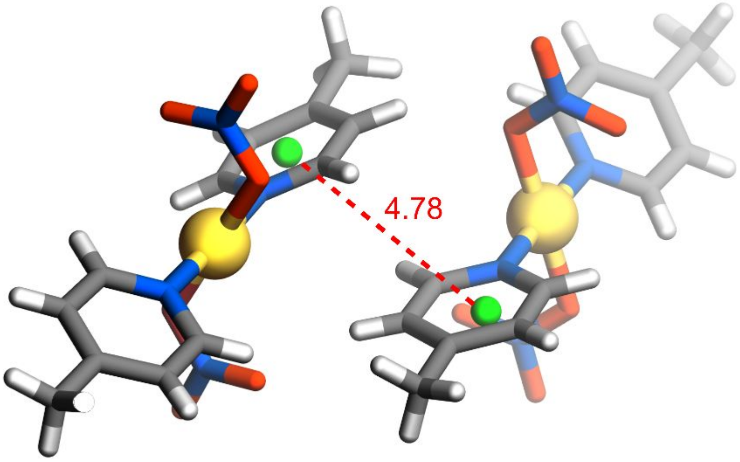

Figure S53. The weak interactions within the crystal structure of *trans*-[Pd(**L2**)$_2$(NO$_3$)$_2$].

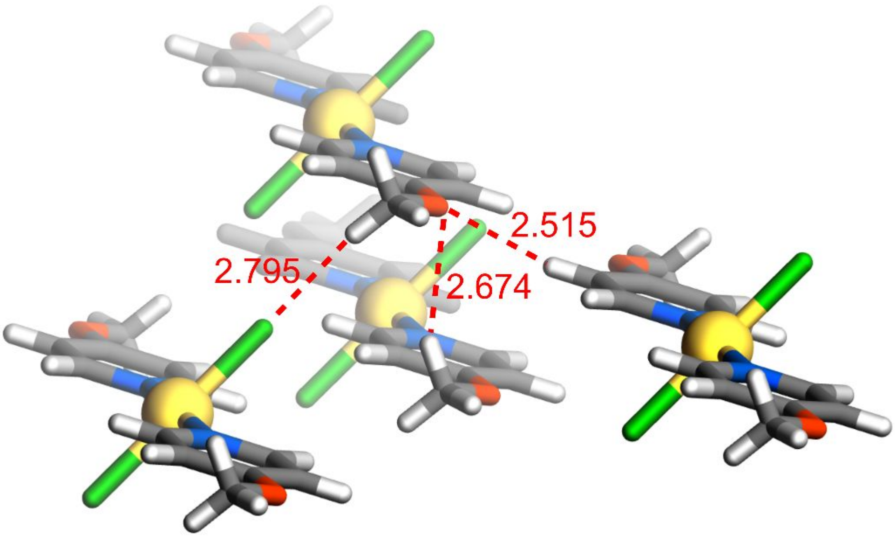

Figure S54. The weak interactions within the crystal structure of *trans*-[Pd(**L3**)$_2$Cl$_2$].

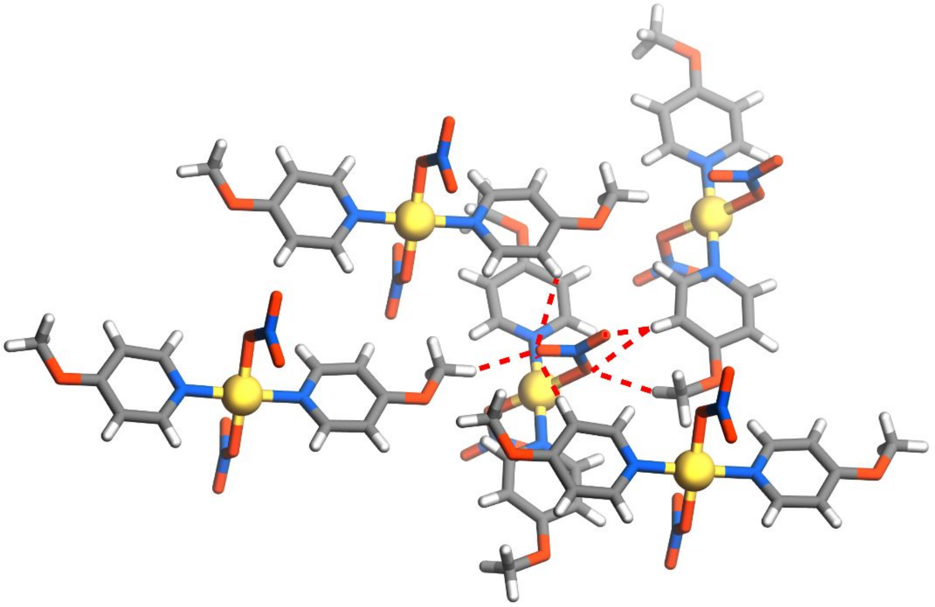

Figure S55. The weak interactions within the crystal structure of *trans*-[Pd(**L3**)$_2$(NO$_3$)$_2$].

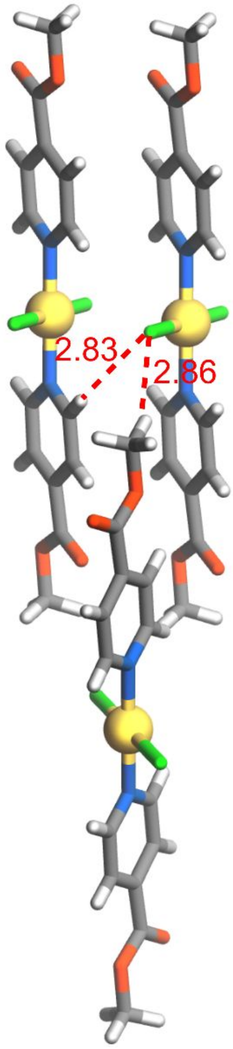

Figure S56. The weak interactions within the crystal structure of [Pd(**L4**)$_2$Cl$_2$].

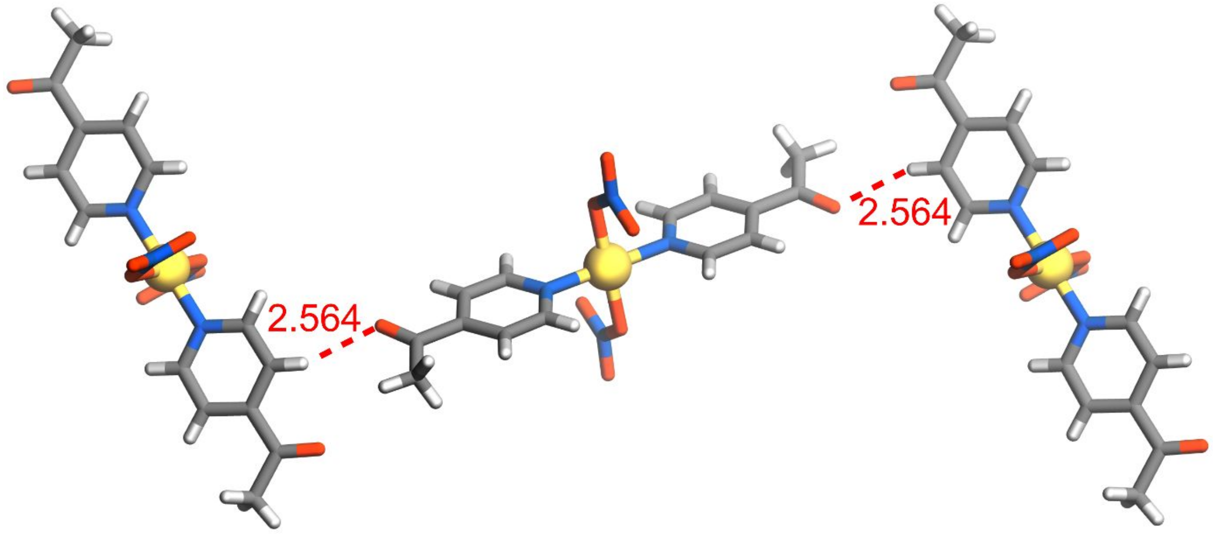

Figure S57. The weak interactions within the crystal structure of [Pd(**L5**)$_2$(NO$_3$)$_2$].

10. Comparison of selected parameters for crystals of Pd(II) complexes

Table S18. Selected geometric parameters for Pd(II) complexes based on the ligands **L1**-**L7**.

Pd(II) complex	Space group	Temp [K]	Pd(II)-N bond length [Å]	Pd(II)-Cl / Pd(II)-O or Pd(II)···O length[a] [Å]	square-planar coordination with valence angles [°]	deviation from the average mean coordination plane[b]	the inclination of pyridine ring relative to the coordination plane (dihedral angle) [°]	coplanarity of opposite pyridine rings (dihedral angle) [°]
[Pd(**L1**)$_2$(NO3)$_2$][8]	*P*-1	100	Pd1-N2 2.0209(11)	Pd1-O11 2.0124(7)	N2-Pd1-O11 90.80(3) N2-Pd1-O11^{i} 89.20(3) (i) –x, -y, -z	0	37.01(3)	coplanar
			Pd2-N4 2.0184(6)	Pd2-O31 2.0265(6)	N4-Pd2-O31 90.89(2) N4-Pd2-O31^{ii} 89.11(2) (ii) –x, -y,1- z	0	76.18(3)	coplanar
[Pd(**L1**)$_2$Cl$_2$][9]	*C*2/*c*	90	Pd1-N1 2.0295(12) Pd1-N2 2.0154(12)	Pd1-Cl1 2.3039(3)	N1-Pd1-Cl1 90.822(7) N2-Pd1-Cl1 89.178(7) N1-Pd1-Cl1^{i} 90.821(7) N1-Pd1-Cl1^{i} 89.179(7) (i) –x+1, y, -z+½	0	46.22(4) – N1-pyridyl 66.74(4) – N2-pyridyl	20.51(5)
[Pd(**L2**)$_2$(NO3)$_2$]	*P*2$_1$/*n*	100	Pd1-N1 2.021(3) Pd1-N2 2.021(3)	Pd1-O1 2.025(2) Pd1-O4 2.014(2)	N1-Pd1-O1 90.57(10) N1-Pd1-O4 89.58(10) N2-Pd1-O1 89.61(9) N2-Pd1-O4 90.27(9)	0.028	140.42(9) – N1-pyridyl 83.51(9) – N2-pyridyl	44.41(11)
[Pd(**L2**)$_2$Cl$_2$][10]	*P*2$_1$/*n*	100	Pd1-N1 2.0139(11)	Pd1-Cl1 2.3054(3)	N1-Pd1-Cl1 89.48(3) N1-Pd1-Cl1^{i} 90.52(3) (i) –x+1, -y, -z	0	125.33(4)	coplanar
[Pd(**L2**)$_4$](NO3)$_2$	*C*2/*c*	RT	Pd1-N1 2.028(2)	Pd1···O1 3.149(3)	N1-Pd1-N2 89.53(7)	0	85.78(9) – N1-pyridyl	coplanar

			Pd1-N2 2.025(2)		N1-Pd1-N2^{i} 90.46(7) (i) –x+1, -y+1, -z+1		92.43(9) – N2-pyridyl	
[Pd(**L3**)$_2$(NO3)$_2$]	*P*$2_12_12_1$	100	Pd1-N1 2.031(5) Pd1-N2 2.018(5)	Pd1-O1 2.020(4) Pd1-O4 2.013(4)	N1-Pd1-O1 89.20(19) N1-Pd1-O4 91.61(19) N2-Pd1-O1 89.11(19) N2-Pd1-O4 89.99(14)	0.020	56.55(17) – N1-pyridyl 46.61(18) – N2-pyridyl	10.3(2)
[Pd(**L4**)$_2$(NO3)$_2$]	*P*-1	100	Pd1-N1 2.014(2) Pd1-N2 2.017(2)	Pd1-O1 2.016(2) Pd1-O4 2.003(2)	N1-Pd1-O1 88.98(7) N1-Pd1-O4 90.28(7) N2-Pd1-O1 92.11(7) N2-Pd1-O4 88.05(7)	0.051	119.49(7) – N1-pyridyl 121.51(6) – N2-pyridyl	3.07(7)
			Pd2-N5 2.011(2)	Pd2-O11 2.011(2)	N5-Pd2-O11 88.75(7) N5-Pd2-O11^{i} 91.25(7) (i) –x+2, -y+1, -z+1	0	79.28(7)	coplanar
			Pd3-N7 2.019(2)	Pd3-O16 2.016(2)	N7-Pd3-O16 89.72(7) N7-Pd3-O16^{ii} 90.28(7) (ii) –x, -y+1, -z	0	42.11(8)	coplanar
[Pd(**L4**)$_2$Cl$_2$]	P2_1/c	100	Pd1-N1 2.018(3)	Pd1-Cl1 2.3085(10)	N1-Pd1-Cl1 89.32(10) N1-Pd1-Cl1^{1} 90.68(10) (i) –x, 1-y, -z	0	52.47(11)	coplanar
[Pd(**L4**)$_2$(NO3)$_2$]	*P*-1	100	Pd1-N1 2.014(2) Pd1-N2 2.017(2)	Pd1-O1 2.016(2) Pd1-O4 2.003(2)	N1-Pd1-O1 88.98(7) N1-Pd1-O4 90.28(7) N2-Pd1-O1 92.11(7) N2-Pd1-O4 88.05(7)	0.051	119.49(7) – N1-pyridyl 121.51(6) – N2-pyridyl	3.07(7)

			Pd2-N5 2.011(2)	Pd2-O11 2.011(2)	N5-Pd2-O11 88.75(7) N5-Pd2-O11^{i} 91.25(7) (i) –x+2, -y+1, -z+1	0	79.28(7)	coplanar
			Pd3-N7 2.019(2)	Pd3-O16 2.016(2)	N7-Pd3-O16 89.72(7) N7-Pd3-O16^{ii} 90.28(7) (ii) –x, -y+1, -z	0	42.11(8)	coplanar
[Pd(**L5**)$_2$(NO3)$_2$]	$P2_1/c$	RT	Pd1-N1 2.018(3)	Pd1-O1 2.007(3)	N1-Pd1-O1 89.51(13) N1-Pd1-O1^{i} 90.49(13) (i) –x, 1-y, -z	0	103.41(16)	coplanar
[Pd(**L6**)$_2$Cl$_2$]	*P*-1	134	Pd1-N1 2.029(3)	Pd1-Cl1 2.3007(7)	N1-Pd1-Cl1 90.29(7) N1-Pd1-Cl1^{i} 89.71(7) (i) –x+2, -y+2, -z	0	116.65(8)	coplanar
[Pd(**L6**)$_4$](NO3)$_2$	*P*-1	100	Pd1-N1 2.030(3) Pd1-N2 2.032(3)	Pd1···O1 3.196 (4)	N1-Pd1-N2 87.58(12) N1-Pd1-N1^{i} 92.42(12) (i) 1–x, 1–y, 1–z	0	90.09(13) – N1-pyridyl 88.27(13) – N2-pyridyl	coplanar
[Pd(**L7**)$_2$Cl$_2$]^{c}	*P*-1	RT	Pd1-N1 2.0167(12)	Pd1-Cl1 2.2985(4)	N1-Pd1-Cl1 90.13(3) N1-Pd1-Cl1^{i} 89.87(3) (i) –x, 1–y, 1–z	0	124.54(4)	coplanar
[Pd(**L7**)$_2$Cl$_2$]^{c} [11]	*P*-1	150	Pd1-N1 2.020(4)	Pd1-Cl1 2.3118(11)	N1-Pd1-Cl1 90.66(11) N1-Pd1-Cl1^{i} 89.34(11) (i) –x, –y, –z	0	124.68(11)	coplanar
[Pd(**L7**)$_2$(NO3)$_2$]	*P*-1	RT	Pd1-N1 2.0094(17) Pd1-N2 2.0116(17)	Pd1-O1 2.0092(16) Pd1-O4 2.0226(16)	N1-Pd1-O1 90.17(7) N1-Pd1-O4 90.64(7) N2-Pd1-O1 89.84(7) N2-Pd1-O4 89.38(7)	0.028	41.56(7) – N1-pyridyl 88.86(7) – N2-pyridyl	47.47(10)

[Pd(**L7**)$_4$](NO3)$_2$	*P*-1	100	Pd1-N1 2.024(2)<br>Pd1-N2 2.018(2)	Pd1···O1 3.210(2)	N1-Pd1-N2<br>91.16(8)<br>N1-Pd1-N2^{i}<br>88.84(8)<br>(i) 2–x, 1–y, 1–z	0	101.84(9) – N1-pyridyl<br>100.88(9) – N2-pyridyl	coplanar
			Pd2-N3 2.024(3)<br>Pd2-N4 2.023(2)	Pd2···O1 2.790(5)	N3-Pd2-N4<br>89.51(10))<br>N3-Pd2-N4i^{i}<br>90.49(10)<br>(ii) 2–x, 1-y, 2-z	0	87.68(10) – N3-pyridyl<br>88.3(2) – N4-pyridyl	coplanar

[a] Pd(II)–Cl or Pd(II)–O bond lengths in disubstituted complexes. Pd(II)···O non-bonded distance in tetra-substituted complexes. [b] Mean coordination plane PdN_2Cl_2 or PdN_2O_2 disubstituted complexes or PdN_4 in tetra-substituted complexes. [c] Structures that are packing polymorphs.

Table S19. C-H···O interactions in the structure of disubstituted complexes with NO_3^- counterions with the participation of protons closest to the pyridyl-*N* atoms of ligand molecules.

Pd(II) complex	D-H···A hydrogen bond	Symmetry code for acceptor atom	H···A [Å]	D-H···A [°]
[Pd(**L2**)$_2$(NO3)$_2$]	C1-H1···O5	x-½, 3/2-y, z-½	2.641(2)	124(1)
	C5-H5···O3	x+½, 3/2-y, z-½	2.626(3)	127.3(2)
	C5A-H5A···O6	1-x, 1-y, 1-z	2.724(3)	127.6(2)
[Pd(**L3**)$_2$(NO3)$_2$]	C1-H1···O7	2-x, ½+y, ½-z	2.644(4)	158.5(4)
	C1A-H1A···O3	x-½, ½-y, 1-z	2.356(5)	170.6(4)
	C5-H5···O6	1-x, y+½, ½-z	2.857(4)	131.3(4)
	C5A-H5A···O2	x-½, ½-y, 1-z	2.508(5)	159.0(4)
[Pd(**L4**)$_2$(NO3)$_2$]	C1-H1···O13		2.6518(17)	163.42(14)
	C1A-H1A···O3		2.7793(18)	133.80(14)
	C6-H6···O13		2.7202(18)	160.35(13)
	C6A-H6A···O3		2.5588(17)	135.46(14)
	C11-H11···O12		2.5275(17)	124.19(14)
	C11A-H11A···O12	2-x, 1-y, 1-z	2.9279(18)	126.34(15)
	C16-H16···O2	-1+x, y, z	2.4495(18)	134.30(15)
	C16-H16···O3	-1+x, y, z	2.5678(16)	139.32(15)
	C16A-H16A···O5	1-x, 1-y, -z	2.4296(17)	121.07(15)
[Pd(**L5**)$_2$(NO3)$_2$]	C1-H1···O2	x, 3/2-y, z+½	2.516(4)	162.3(3)
	C1A-H1A···O3	-x, y-½, -z-½	2.377(8)	163.1(3)
[Pd(**L7**)$_2$(NO3)$_2$]	C1-H1···O2	1–x, 1–y, 1–z	2.86(3)	123.3(19)
	C1A-H1A···O4	-x, -y, 1-z	2.84(2)	126.2(18)
	C4A-H4A···O3	1–x, –y, 1–z	2.71(3)	143(2)

Table S20. C-H···Cl interactions in disubstituted complexes with Cl^- counterions with the participation of protons closest to the pyridyl-*N* atoms of ligand molecules.

Pd(II) complex	D-H···A hydrogen bond	Symmetry code for acceptor atom	H···A [Å]	D-H···A [°]
[Pd(**L3**)$_2$Cl$_2$]	*no significant non-covalent interactions involving ortho-protons*			
[Pd(**L4**)$_2$Cl$_2$]	C1-H1···Cl1	1-x, 1-y, -z	2.8313(11)	133.9(3)
	C1A-H1A···O1	-x, -½+y, ½-z	2.600(3)	124.9(3)
[Pd(**L6**)$_2$Cl$_2$]	*no significant non-covalent interactions involving ortho-protons*			
[Pd(**L7**)$_2$Cl$_2$]	C1-H1···Cl1	x+1, y, z	2.869(19)	126.0(14)
	C1A-H1A···Cl1	x, y+1, z	2.775(19)	134.1(15)

Table S21. C-H···O interactions in tetra-substituted complexes with NO_3^- counterions with the participation of protons closest to the pyridyl-*N* atoms of ligand molecules.

Pd(II) omplex	D-H···A hydrogen bond	Symmetry code for acceptor atom	H···A [Å]	D-H···A [°]
[Pd(**L2**)$_4$](NO3)$_2$	C1-H1···O1		2.554(3)	148.5(2)
	C1A-H1A···O1	1-x, 1-y, 1-z	2.526(4)	148.33(16)
	C5-H5···O1		2.348(3)	150.02(18)
	C5A-H5A···O1	1-x, 1-y, 1-z	2.754(3)	145.68(17)
[Pd(**L4**)$_4$](NO3)$_2$	C1-H1···O15		2.683(6)	150.7(3)
	C1A-H1A···O9		2.674(5)	150.2(3)
	C6-H6···O15		2.734(4)	151.69(3)
	C6-H6···O5	1-x, 1-y, 2-z	2.566(4)	126.3(3)
	C6A-H6A···O9		2.538(4)	149.3(3)
	C11-H11···O15		2.580(5)	152.5(3)

	C11A-H11A···O9		2.620(5)	152.2(3)
	C11A-H11A···O10		2.361(5)	156.2(3)
	C16-H16···O15		2.523(5)	153.4(3)
	C16A-H16A···O9		2.753(4)	151.2(3)
	C16A-H16A···O2	1-x, 1-y, 1-z	2.544(3)	134.6(3)
$[Pd(\mathbf{L6})_4](NO3)_2$	C1-H1···O1		2.698(6)	148.3(3)
	C6-H6···O1		2.567(4)	149.3(3)
	C1A-H1A···O1	1–x, 1–y, 1–z	2.449(5)	150.2(3)
	C6A-H6A···O1	1–x, 1–y, 1–z	2.577(5)	148.6(3)
$[Pd(\mathbf{L7})_4](NO3)_2$	C1-H1···O3		2.412(2)	145.5(2)
	C1A-H1A···O2	2-x, 1-y, 1-z	2.667(2)	168.3(2)
	C4-H4···O6		2.382(2)	131.7(2)
	C4A-H4A···O2		2.282(2)	168.2(2)
	C7-H7···O1		2.544(2)	148.6(2)
	C7A-H7A···O1	2-x, 1-y, 2-z	2.651(2)	147.5(2)
	C10[a]-H10[a]···O1		2.318(2)	150.7(4)
	C10B[a]-H10B[a]···O1		2.315(2)	142.6(4)
	C10A-H10A[a]···O2		2.219(2)	169.2(2)
	C10A-H10C[a]···O2		2.300(2)	149.6(2)

[a] The atoms with partial occupancy (50%).

11. Investigation of catalytic activity in the Suzuki-Miyaura cross-coupling

11.1. Reaction development for the Suzuki-Miyaura cross-coupling

Table S22. Reaction development for the Suzuki-Miyaura cross-coupling between phenylboronic acid and 4'-bromoacetophenone.[a]

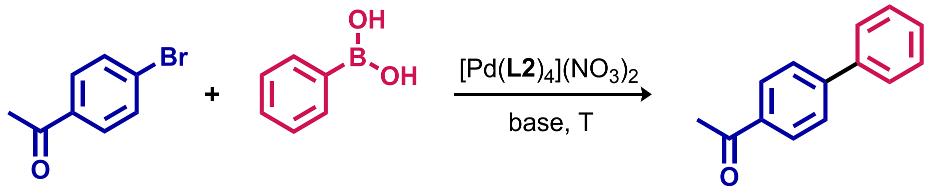

	solvent	base	T [°C]	mol% Pd	GC yield [%][b] 0.5 h	2 h	4 h
1	chloroform	K_3PO_4	80	0.1	59	70	84
2	1,4-dioxane	K_3PO_4	80	0.1	26	29	43
**3**	**toluene**	$\mathbf{K_3PO_4}$	**80**	**0.1**	**86**	**98**	**98**
4	DMF	K_3PO_4	120	0.1	9	13	18
5	toluene	K_3PO_4 (s)[c]	80	0.1	14	38	52
6	toluene	K_2CO_3	80	0.1	82	89	97
7	toluene	NaOH	80	0.1	14	16	18
8	toluene	Et_3N	80	0.1	15	17	20
9	toluene	K_3PO_4	80	0.01	28	42	63
10	toluene	K_3PO_4	80	1	99	100	100

[a] Reaction conditions: 4'-bromoacetophenone (0.2 mmol, 1 equiv.), phenylboronic acid (0.24 mmol, 1.2 equiv.), base (0.4 mmol, 2 equiv.) and the complex $[Pd(\mathbf{L2})_4](NO_3)_2$ were stirred in appropriate solvent (2 mL) at indicated temperature under air atmosphere. [b] Determined by GC measurement of 4'-bromoacetophenone decay. [c] As 2 M aqueous solution.

11.2. Catalytic activity of Pd(II) complexes based on pyridine ligands in the Suzuki-Miyaura reaction

Table S23. Catalytic activity of Pd(II) complexes based on pyridine ligands in the Suzuki-Miyaura cross-coupling between 4'-bromoacetophenone and phenylboronic acid.[a]

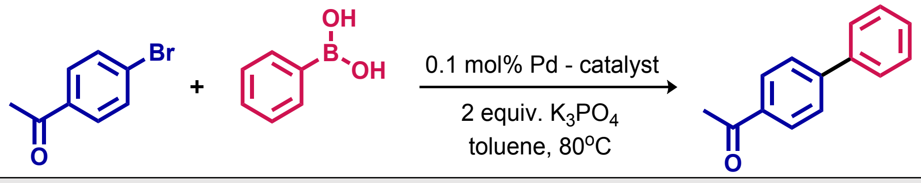

GC yields [%][b]	[Pd**L**$_2$Cl$_2$]	[Pd**L**$_2$(NO$_3$)$_2$]	[Pd**L**$_4$](NO$_3$)$_2$
**L1**	97	93	95
**L2**	93	92	98
**L3**	93	91	91
**L4**	78	72	64
**L5**	86	87	88
**L6**	93	-	90
**L7**	82	74	75
**L8**	88	66	-
**L9**	87	70	-
**L10**	98	-	90
**L11**	86	-	79
**L12**	83	-	92

[a] Reaction conditions: 4'-bromoacetophenone (0.2 mmol, 1 equiv.), phenylboronic acid (0.24 mmol, 1.2 equiv.), base (0.4 mmol, 2 equiv.) and Pd(II) complex (0.1 mol%) were stirred in toluene (2 mL) at 80°C under air atmosphere for 2 h. [b] Determined by GC measurement of 4'-bromoacetophenone decay as the average of three results.

11.3. General synthetic procedure for the Suzuki-Miyaura cross-coupling

A reaction vessel equipped with a stirring bar was charged with aryl bromide **1a-c** (1.0 mmol, 1.0 equiv.) and arylboronic acid **2a-c** (1.2 mmol, 1.2 equiv.) which were dissolved in toluene (10 mL). After, the catalyst [Pd(**L2**)$_4$](NO$_3$)$_2$ (0.001 mmol, 0.001 equiv.) as a solution in chloroform (0.05 mL) and solid K_3PO_4 (2.0 mmol, 2.0 equiv.) were added. The vial was sealed and the reaction mixture was heated for 2 h at 80°C. The resulting solution was then cooled to room temperature, diluted with dichloromethane (50 mL) and washed with distilled water (40 mL). The collected aqueous phase was extracted with dichloromethane (2 × 50 mL). The organic layers were gathered, dried over Na_2SO_4, filtered and the solvent was removed under reduced pressure. The residue was purified by column chromatography on silica gel to obtain the desired products **3aa-cc**.

11.4. Characterization of the cross-coupling products

11.4.1. 4-acetylbiphenyl (3aa)[12]

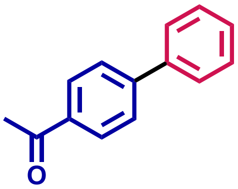

The reaction of 4'-bromoacetophenone **1a** (1 mmol, 199 mg) with phenylboronic acid **2a** (1.2 mmol, 146 mg) according to the general procedure (flash chromatography: hexane/ethyl acetate 20:1) gave 4-acetylbiphenyl **3aa** in the form of white solid. Yield: 93%, 182 mg.

^{1}H NMR (600 MHz, CDCl$_3$) δ = 8.04 (d, *J* = 8.4 Hz, 2H), 7.69 (d, *J* = 8.4 Hz, 2H), 7.63 (d, *J* = 7.2 Hz, 2H), 7.48 (t, *J* = 7.6 Hz, 2H), 7.41 (t, *J* = 7.4 Hz, 1H), 2.64 (s, 3H).

^{13}C NMR (151 MHz, $CDCl_3$) δ = 197.88, 145.92, 140.02, 135.99, 129.09, 129.05, 128.37, 127.41, 127.36, 26.82.

11.4.2. 1-(4'-trifluoromethyl-biphenyl-4-yl)-ethanone (3ab)[13]

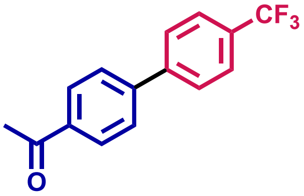

The reaction of 4’-bromoacetophenone **1a** (1 mmol, 199 mg) with 4-(trifluoromethyl)phenylboronic acid **2b** (1.2 mmol, 228 mg) according to the general procedure (flash chromatography: hexane/ethyl acetate 10:1) gave 1-(4'-trifluoromethyl-biphenyl-4-yl)-ethanone **3ab** in the form of white solid. Yield: 84%, 222 mg.

^{1}H NMR (600 MHz, $CDCl_3$) δ = 8.06 (d, J = 8.4 Hz, 2H), 7.73 (s, 4H), 7.70 (d, J = 8.4 Hz, 2H), 2.65 (s, 3H).

^{13}C NMR (151 MHz, $CDCl_3$) δ = 197.70, 144.32, 143.55, 136.74, 130.39 (q, J = 32.5 Hz), 129.19, 127.76, 127.61, 126.96, 126.05 (q, J = 3.8 Hz), 124.26 (q, J = 272.0 Hz), 26.86.

11.4.3. 1-(4'-methyl-biphenyl-4-yl)-ethanone (3ac)[14]

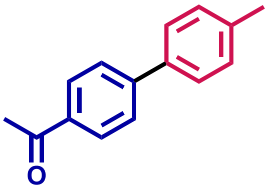

The reaction of 4’-bromoacetophenone **1a** (1 mmol, 199 mg) with 4-tolylboronic acid **2c** (1.2 mmol, 163 mg) according to the general procedure (flash chromatography: hexane/ethyl acetate 25:1) gave 1-(4'-methyl-biphenyl-4-yl)-ethanone **3ac** in the form of white solid. Yield: 90%, 189 mg.

^{1}H NMR (600 MHz, $CDCl_3$) δ = 8.02 (d, J = 8.4 Hz, 2H), 7.67 (d, J = 8.4 Hz, 2H), 7.54 (d, J = 8.0 Hz, 2H), 7.28 (d, J = 8.0 Hz, 2H), 2.63 (s, 3H), 2.41 (s, 3H).

^{13}C NMR (151 MHz, $CDCl_3$) δ = 197.87, 145.85, 138.37, 137.09, 135.74, 129.82, 129.04, 127.23, 127.08, 26.79, 21.31.

11.4.4. Biphenyl (3ba)[12]

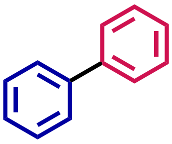

The reaction of bromobenzene **1b** (1 mmol, 105 μL) with phenylboronic acid **2a** (1.2 mmol, 146 mg) according to the general procedure (flash chromatography: hexane) gave biphenyl **3ba** in the form of white solid. Yield: 77%, 119 mg.

^{1}H NMR (600 MHz, $CDCl_3$) δ = 7.60 (d, J = 7.2 Hz, 4H), 7.45 (t, J = 7.6 Hz, 4H), 7.35 (t, J = 7.4 Hz, 2H).

^{13}C NMR (151 MHz, $CDCl_3$) δ = 141.38, 128.89, 127.39, 127.31.

11.4.5. 4-(trifluoromethyl)biphenyl (3bb)[13]

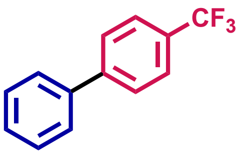

The reaction of bromobenzene **1b** (1 mmol, 105 μL) with 4-(trifluoromethyl)phenylboronic acid **2b** (1.2 mmol, 228 mg) according to the general procedure (flash chromatography: hexane/ethyl acetate 40:1) gave 4-(trifluoromethyl)biphenyl **3bb** in the form of white solid. Yield: 81%, 180 mg.

^{1}H NMR (600 MHz, $CDCl_3$) δ = 7.70 (s, 4H), 7.60 (d, J = 7.5 Hz, 2H), 7.48 (t, J = 7.5 Hz, 2H), 7.41 (t, J = 7.3 Hz, 1H).

^{13}C NMR (151 MHz, $CDCl_3$) δ = 144.87, 139.92, 129.48 (q, J = 32.7 Hz), 129.13, 128.32, 127.57, 127.43, 125.85 (q, J = 3.8 Hz), 124.45 (q, J = 272.0 Hz).

11.4.6. 4-phenyltoluene (3bc)[15]

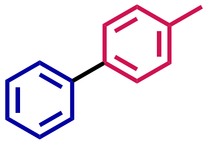

The reaction of bromobenzene **1b** (1 mmol, 105 μL) with 4-tolylboronic acid **2c** (1.2 mmol, 163 mg) according to the general procedure (flash chromatography: hexane) gave 4-phenyltoluene **3bc** in the form of white solid. Yield: 70%, 118 mg.

^{1}H NMR (600 MHz, $CDCl_3$) δ = 7.59 (d, *J* = 7.3 Hz, 2H), 7.50 (d, *J* = 8.0 Hz, 2H), 7.43 (t, *J* = 7.7 Hz, 2H), 7.34 (t, *J* = 7.4 Hz, 1H), 7.26 (d, *J* = 7.7 Hz, 2H), 2.40 (s, 3H).

^{13}C NMR (151 MHz, $CDCl_3$) δ = 141.30, 138.50, 137.16, 129.61, 128.85, 127.14, 127.12, 127.10, 21.25.

11.4.7. 3,5-dimethoxybiphenyl (3ca)[16]

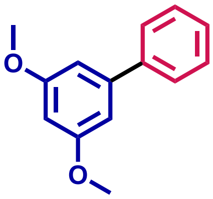

The reaction of 1-bromo-3,5-dimethoxybenzene **1c** (1 mmol, 217 mg) with phenylboronic acid **2a** (1.2 mmol, 146 mg) according to the general procedure (flash chromatography: hexane/ethyl acetate 10:1 to 9:1) gave 3,5-dimethoxybiphenyl **3ca** in the form colorless yellow oil. Yield: 90%, 193 mg.

^{1}H NMR (600 MHz, $CDCl_3$) δ = 7.58 (d, *J* = 7.1 Hz, 2H), 7.43 (t, *J* = 7.6 Hz, 2H), 7.36 (t, *J* = 7.4 Hz, 1H), 6.74 (d, *J* = 2.2 Hz, 2H), 6.48 (t, *J* = 2.2 Hz, 1H), 3.85 (s, 6H).

^{13}C NMR (151 MHz, $CDCl_3$) δ 161.18, 143.64, 141.35, 128.84, 127.69, 127.34, 105.61, 99.43, 55.57.

11.4.8. 3,5-dimethoxy-4'-(trifluoromethyl)-biphenyl (3cb)[17]

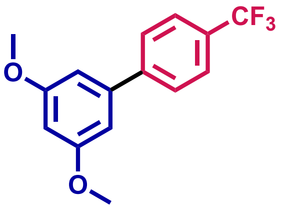

The reaction of 1-bromo-3,5-dimethoxybenzene **1c** (1 mmol, 217 mg) with 4-(trifluoromethyl)phenylboronic acid **2b** (1.2 mmol, 228 mg) according to the general procedure (flash chromatography: hexane/ethyl acetate 25:1 to 20:1) gave 3,5-dimethoxy-4'-(trifluoromethyl)-biphenyl **3cb** in the form of white solid. Yield: 78%, 220 mg.

^{1}H NMR (600 MHz, $CDCl_3$) δ = 7.68 (s, 4H), 6.72 (d, *J* = 2.2 Hz, 2H), 6.51 (t, *J* = 2.2 Hz, 1H), 3.86 (s, 6H).

^{13}C NMR (151 MHz, $CDCl_3$) δ = 161.36, 144.85, 142.10, 129.74 (q, *J* = 32.5 Hz), 127.64, 125.79 (q, *J* = 3.7 Hz), 124.39 (q, *J* = 271.8 Hz), 105.78, 100.09, 55.63.

11.4.9. 3,5-dimethoxy-4'-methyl-biphenyl (3cc)[16]

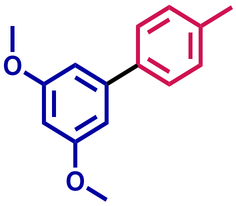

The reaction of 1-bromo-3,5-dimethoxybenzene **1c** (1 mmol, 217 mg) with 4-tolylboronic acid **2c** (1.2 mmol, 163 mg) according to the general procedure (flash chromatography: hexane/ethyl acetate 30:1) gave 3,5-dimethoxy-4'-methyl-biphenyl **3cc** in the form of colorless oil. Yield: 76%, 173 mg.

^{1}H NMR (600 MHz, $CDCl_3$) δ = 7.48 (d, *J* = 8.0 Hz, 2H), 7.24 (d, *J* = 7.9 Hz, 2H), 6.73 (d, *J* = 2.2 Hz, 2H), 6.46 (t, *J* = 2.2 Hz, 1H), 3.85 (s, 6H), 2.40 (s, 3H).

^{13}C NMR (151 MHz, $CDCl_3$) δ = 161.16, 143.56, 138.45, 137.51, 129.55, 127.16, 105.42, 99.17, 55.55, 21.26.

11.5. NMR spectra of the cross-coupling products

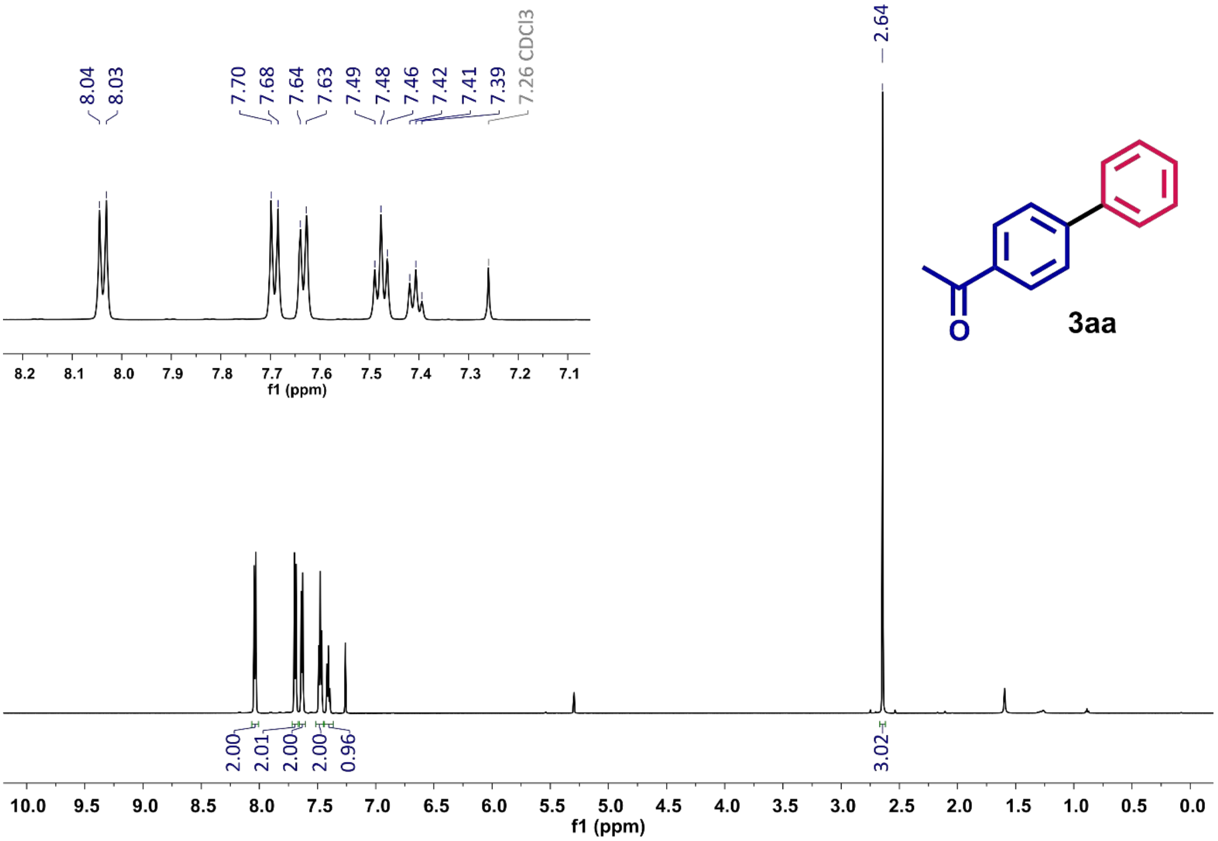

Figure S58. ^{1}H NMR spectrum (600 MHz, $CDCl_3$) of 4-acetylbiphenyl **3aa**.

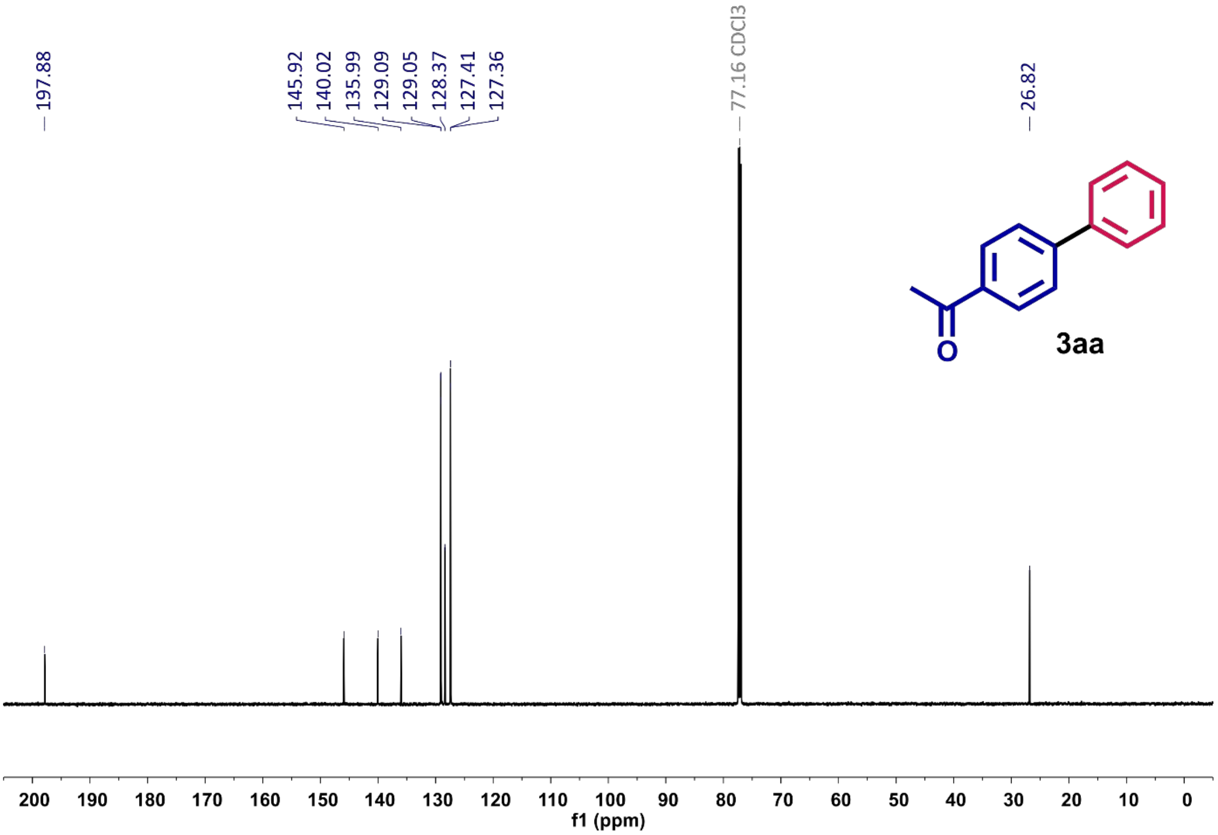

Figure S59. ^{13}C NMR spectrum (151 MHz, $CDCl_3$) of 4-acetylbiphenyl **3aa**.

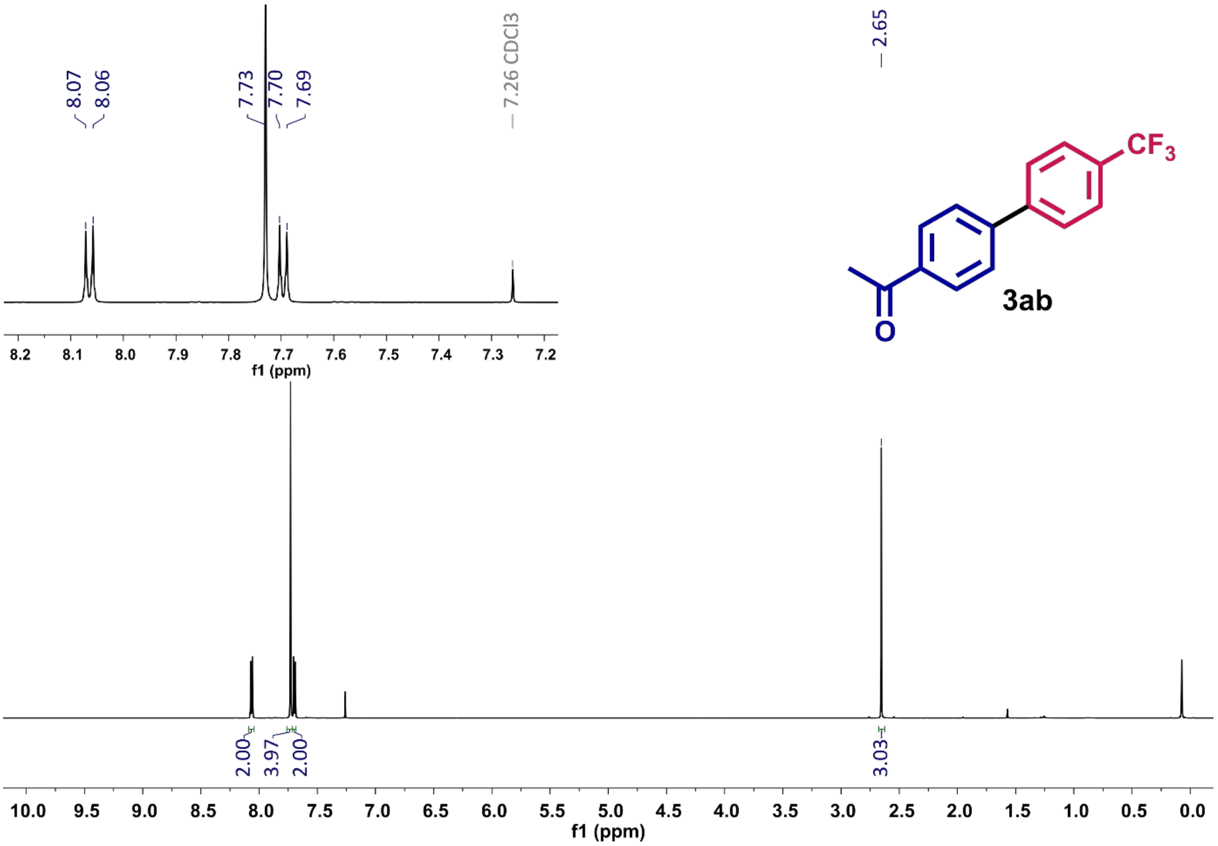

Figure S60. ^{1}H NMR spectrum (600 MHz, $CDCl_3$) of 1-(4'-trifluoromethyl-biphenyl-4-yl)-ethanone **3ab**.

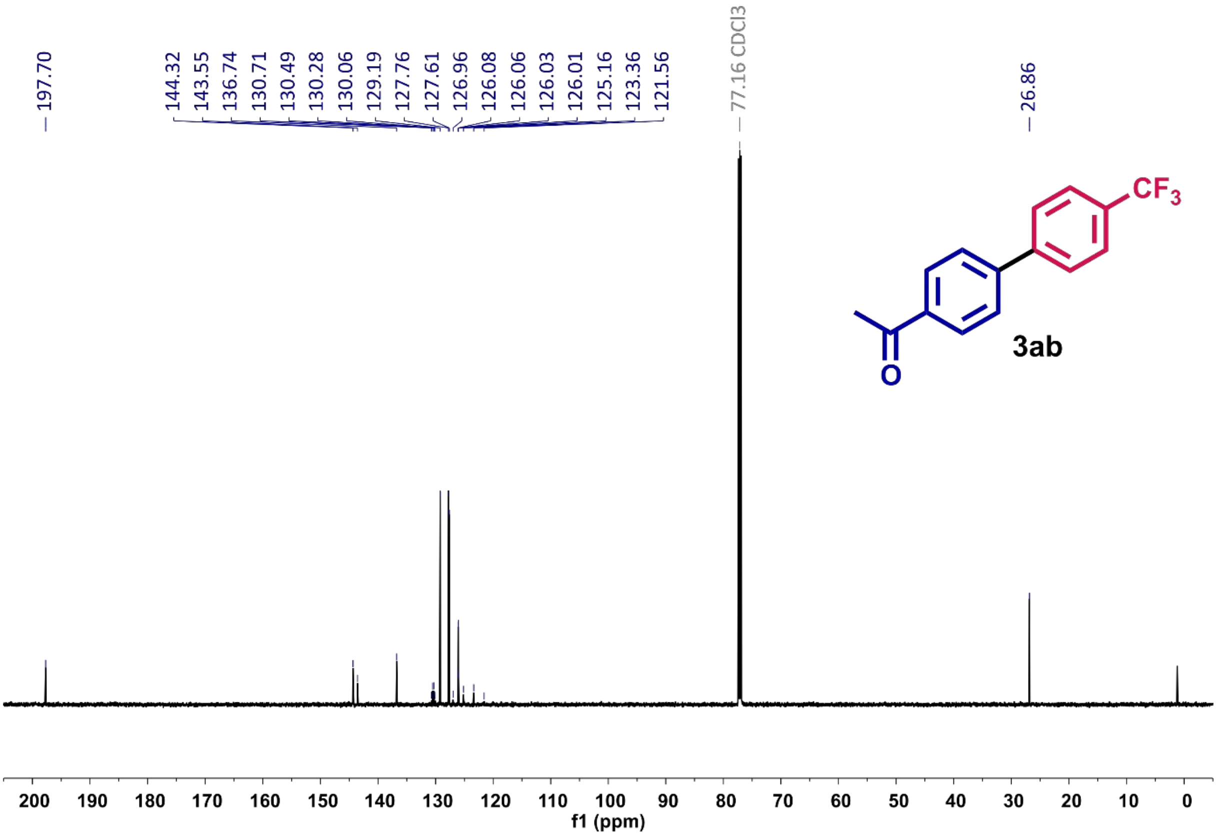

Figure S61. ^{13}C NMR spectrum (151 MHz, $CDCl_3$) of 1-(4'-trifluoromethyl-biphenyl-4-yl)-ethanone **3ab**.

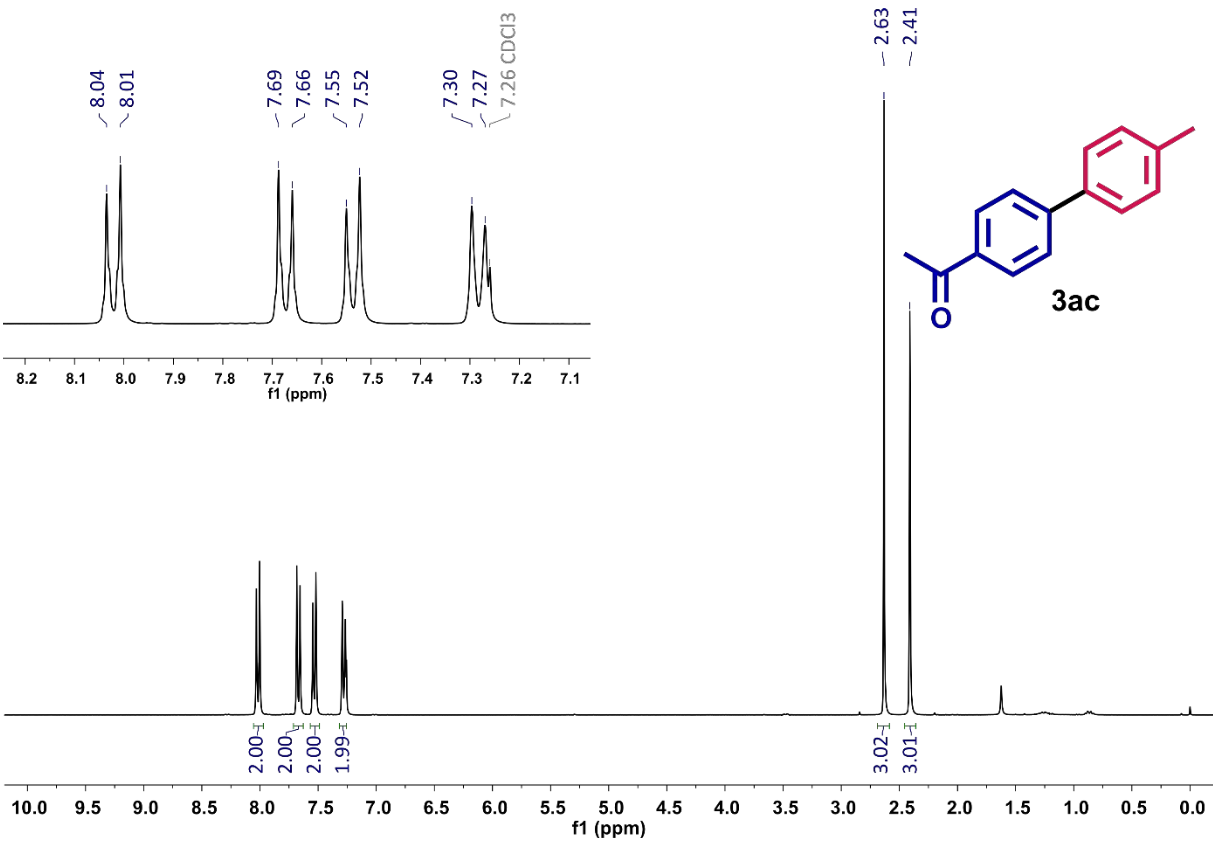

Figure S62. ^{1}H NMR spectrum (600 MHz, $CDCl_3$) of 1-(4'-methyl-biphenyl-4-yl)-ethanone **3ac**.

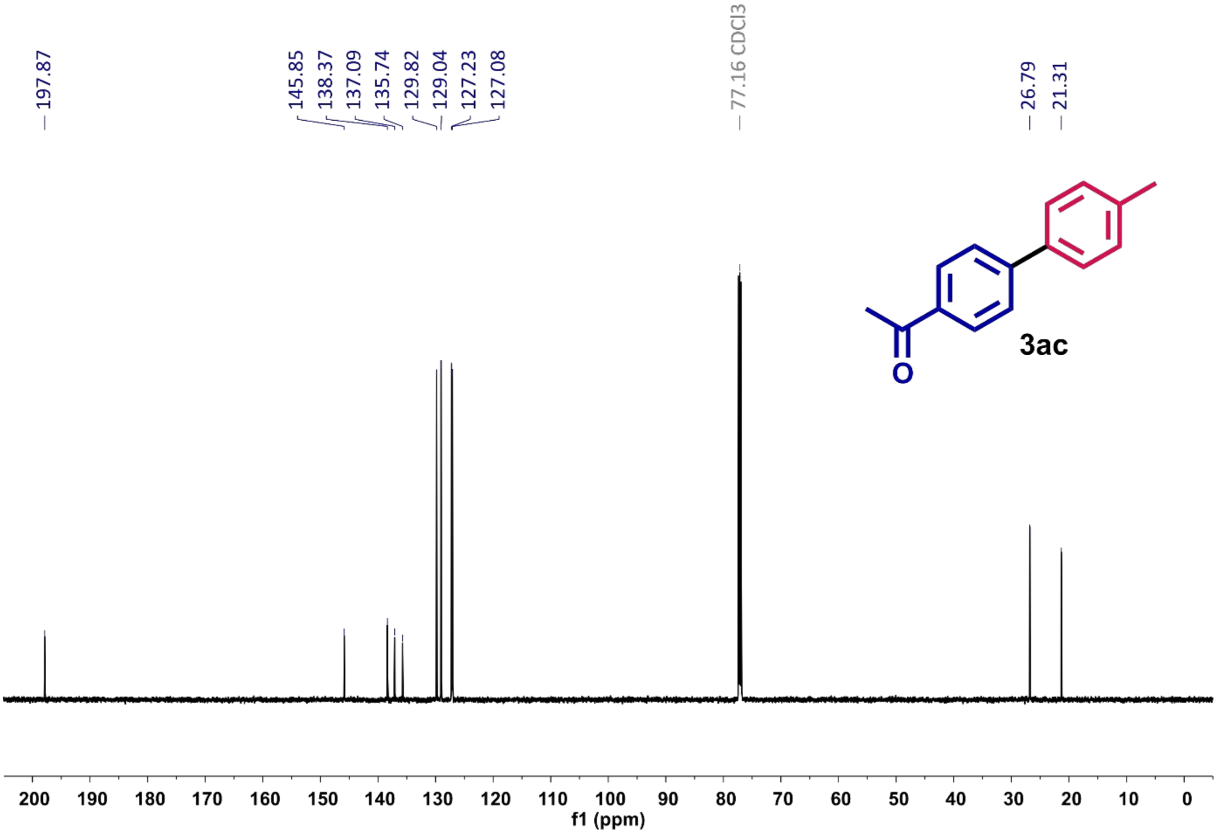

Figure S63. ^{13}C NMR spectrum (151 MHz, $CDCl_3$) of 1-(4'-methyl-biphenyl-4-yl)-ethanone **3ac**.

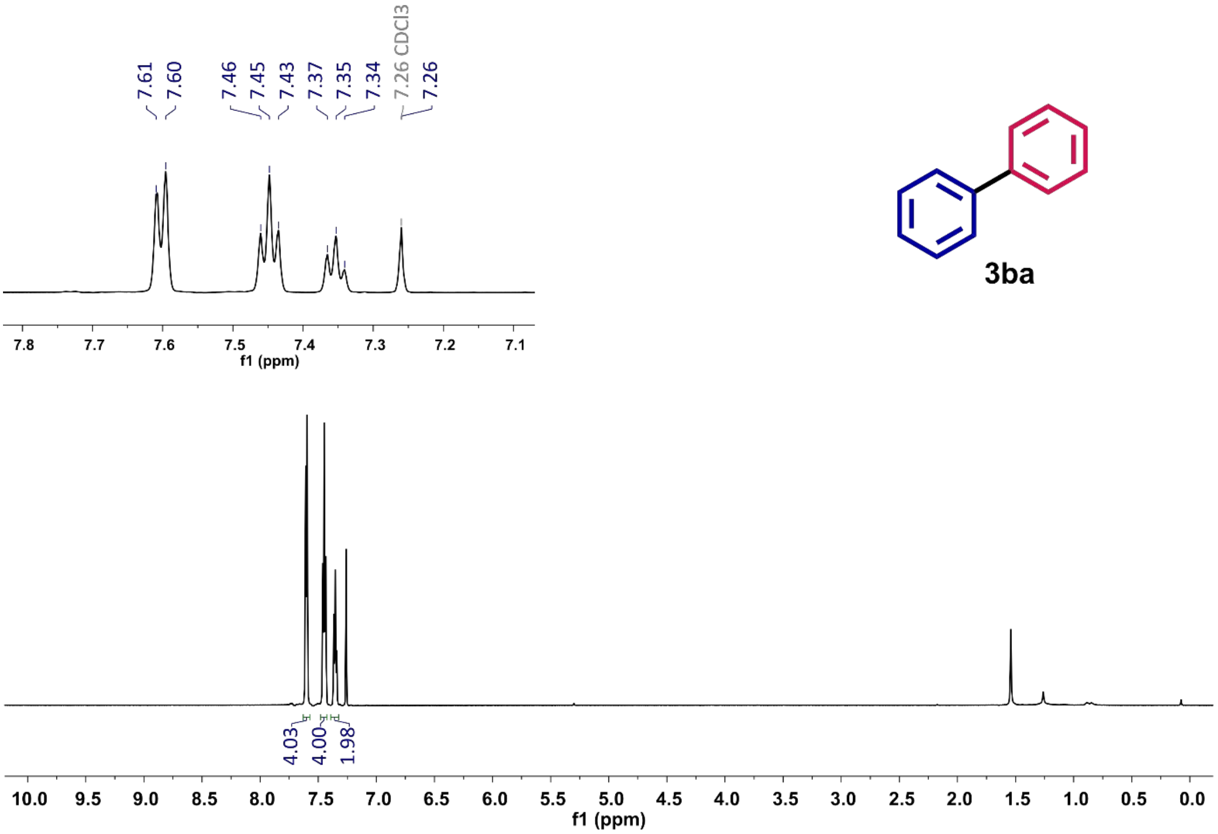

Figure S64. ^{1}H NMR spectrum (600 MHz, $CDCl_3$) of biphenyl **3ba**.

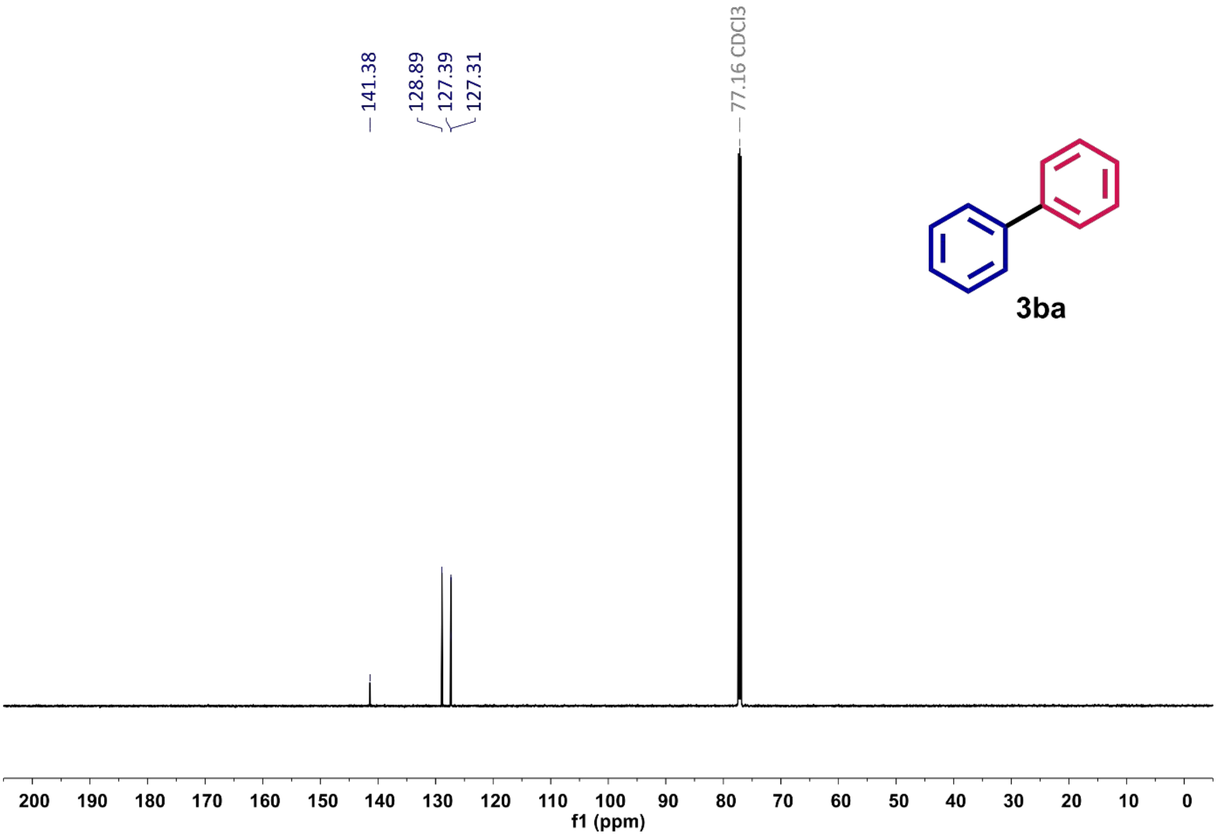

Figure S65. ^{13}C NMR spectrum (151 MHz, $CDCl_3$) of biphenyl **3ba**.

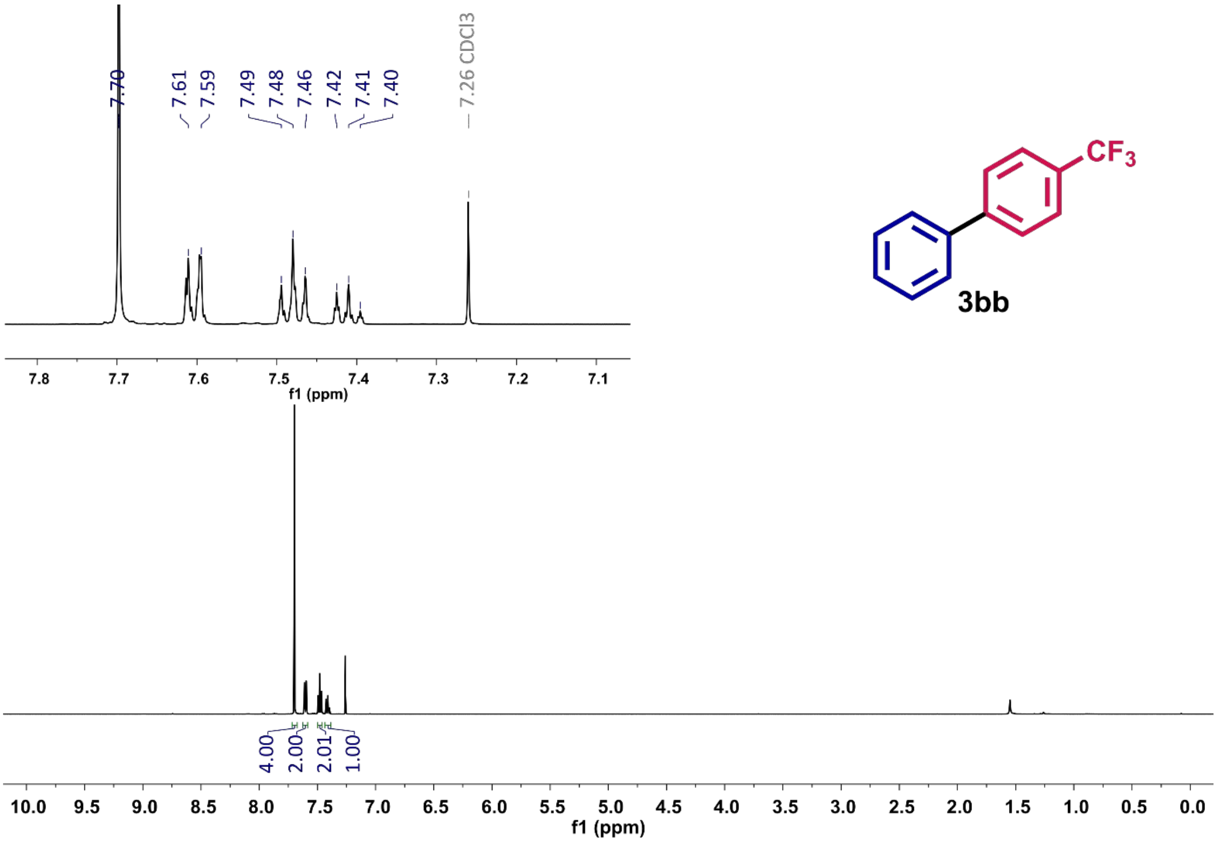

Figure S66. ^{1}H NMR spectrum (600 MHz, $CDCl_3$) of 4-(trifluoromethyl)biphenyl **3bb**.

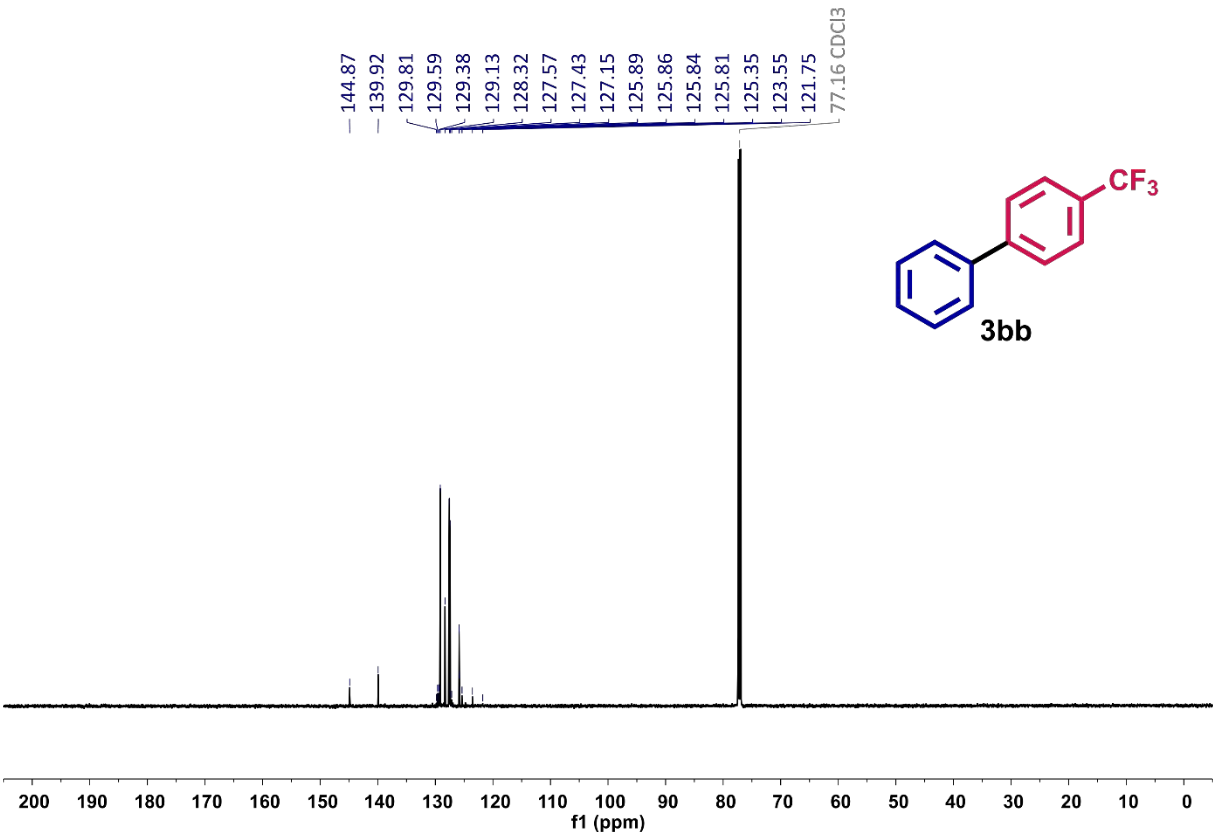

Figure S67. ^{13}C NMR spectrum (151 MHz, $CDCl_3$) of 4-(trifluoromethyl)biphenyl **3bb**.

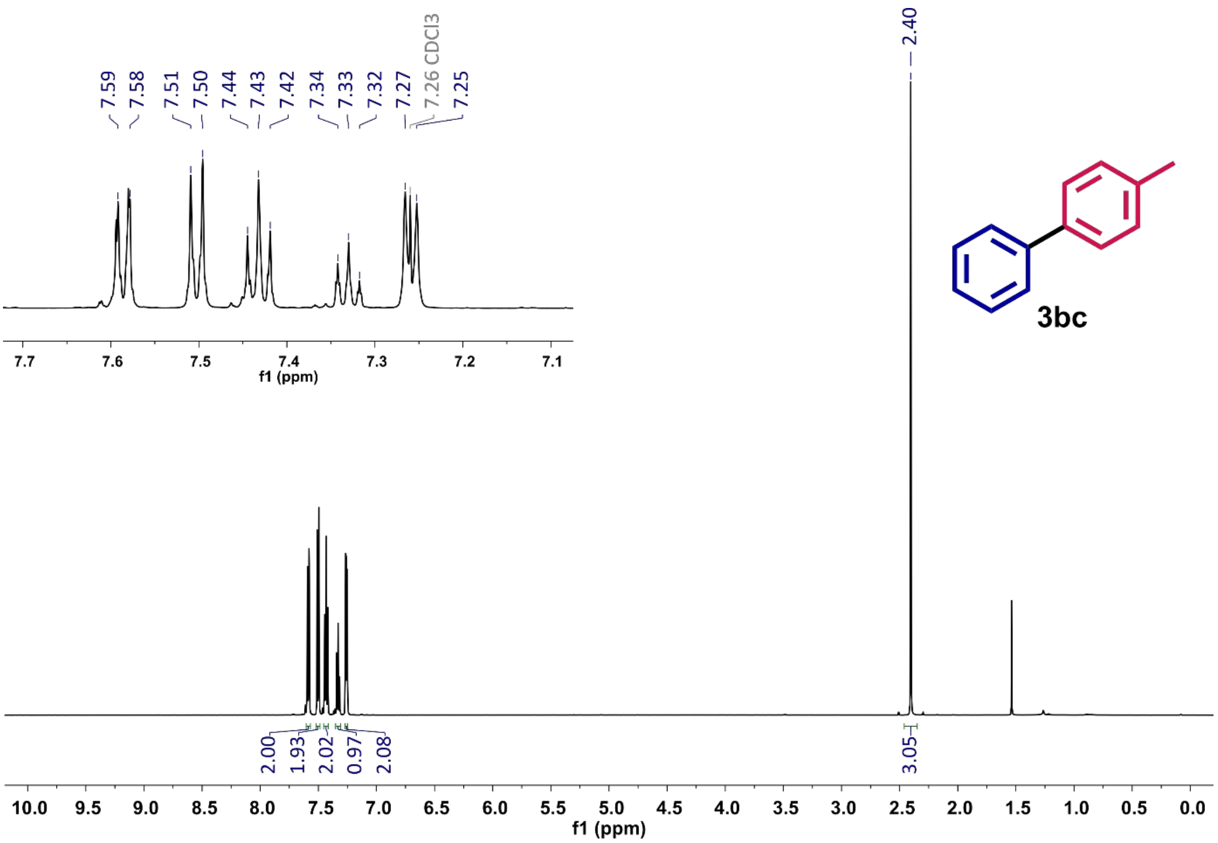

Figure S68. ^{1}H NMR spectrum (600 MHz, $CDCl_3$) of 4-phenyltoluene **3bc**.

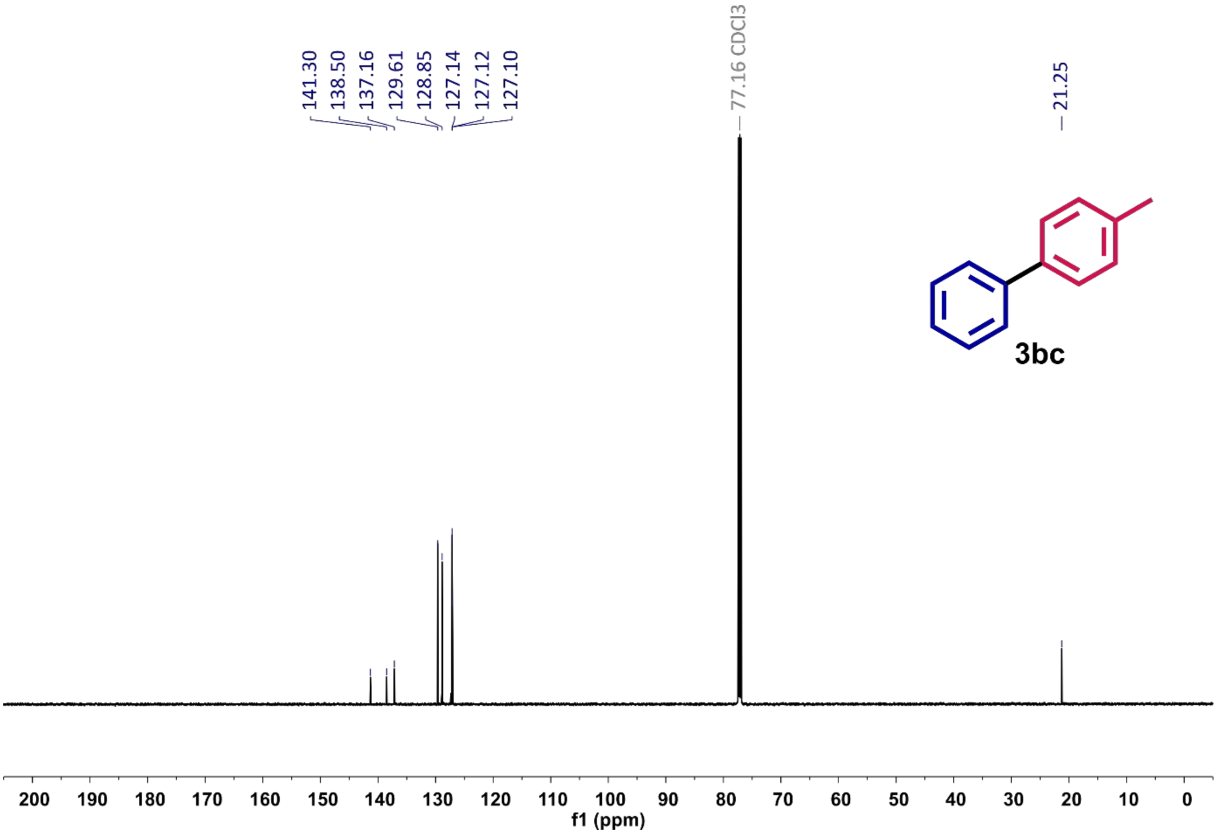

Figure S69. ^{13}C NMR spectrum (151 MHz, $CDCl_3$) of 4-phenyltoluene **3bc**.

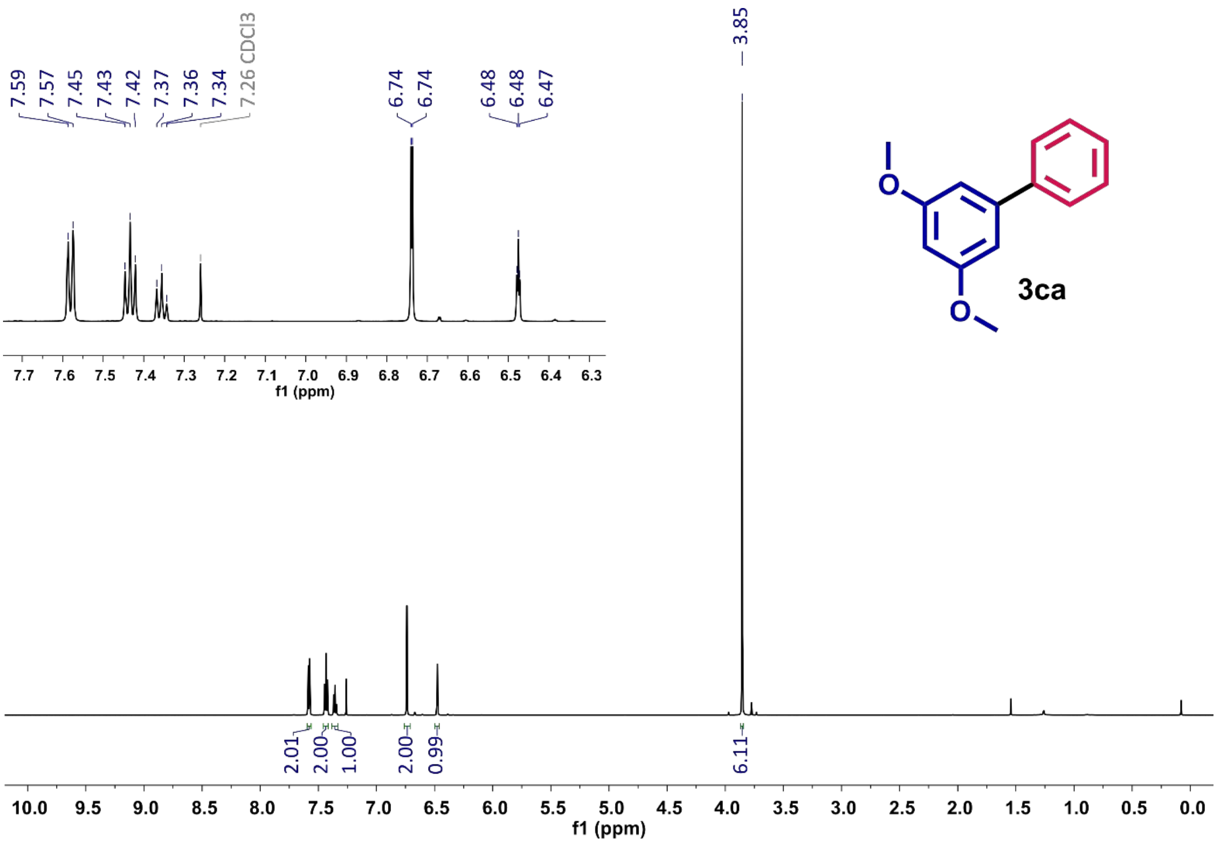

Figure S70. ^{1}H NMR spectrum (600 MHz, $CDCl_3$) of 3,5-dimethoxybiphenyl **3ca**.

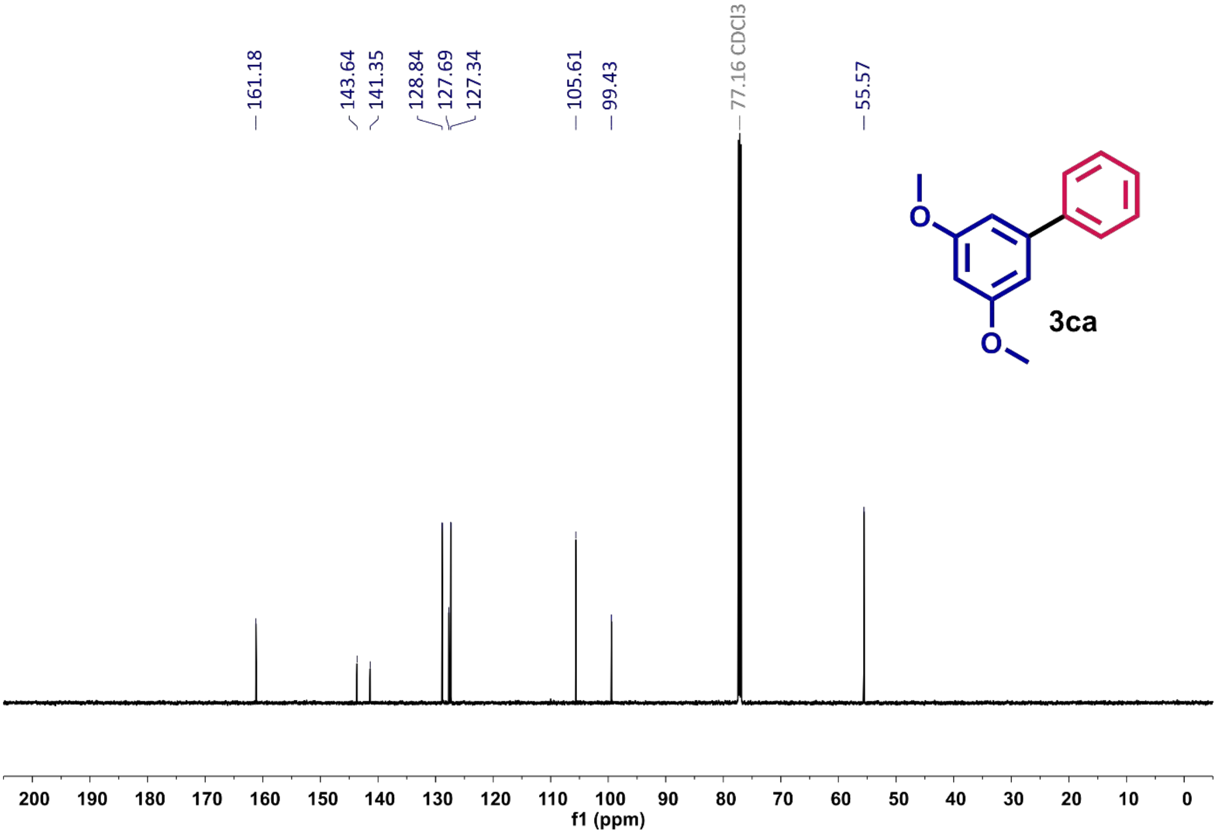

Figure S71. ^{13}C NMR spectrum (151 MHz, $CDCl_3$) of 3,5-dimethoxybiphenyl **3ca**.

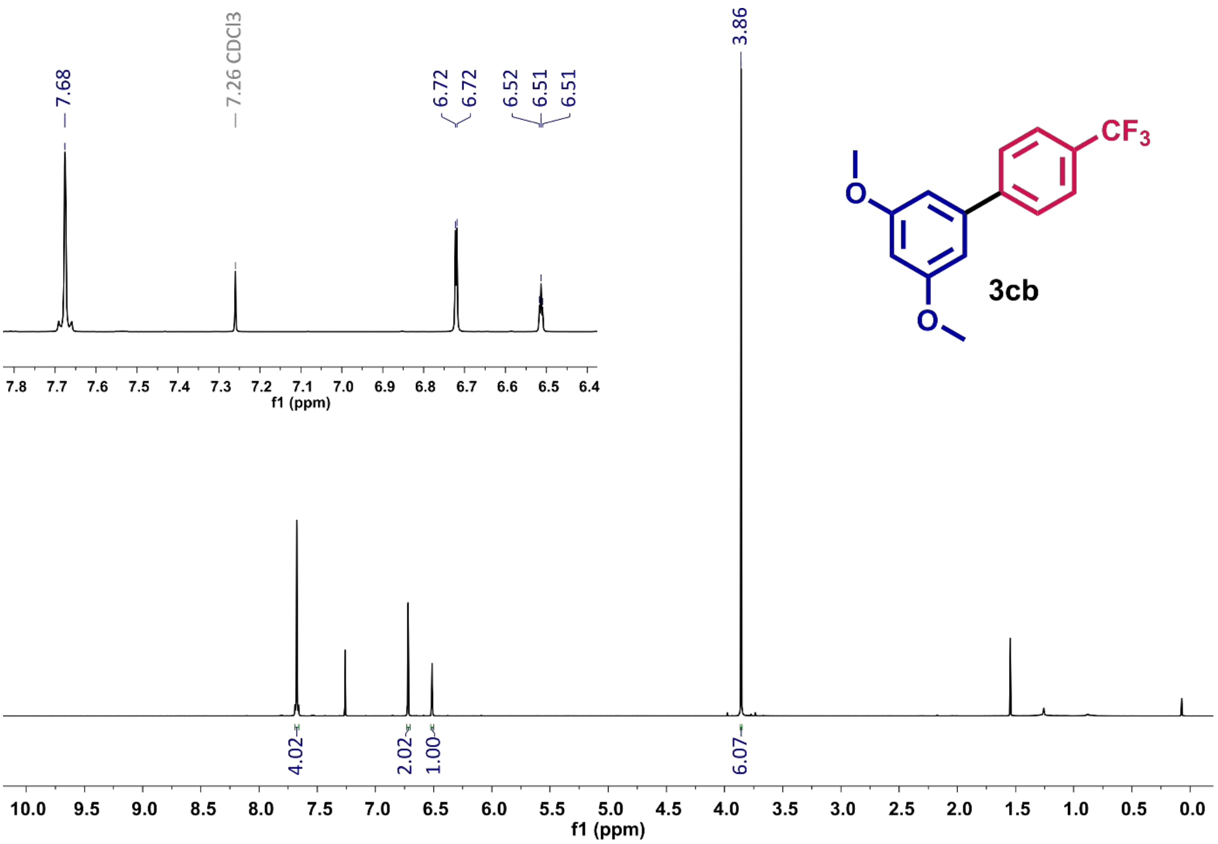

Figure S72. ^{1}H NMR spectrum (600 MHz, $CDCl_3$) of 3,5-dimethoxy-4'-(trifluoromethyl)-biphenyl **3cb**.

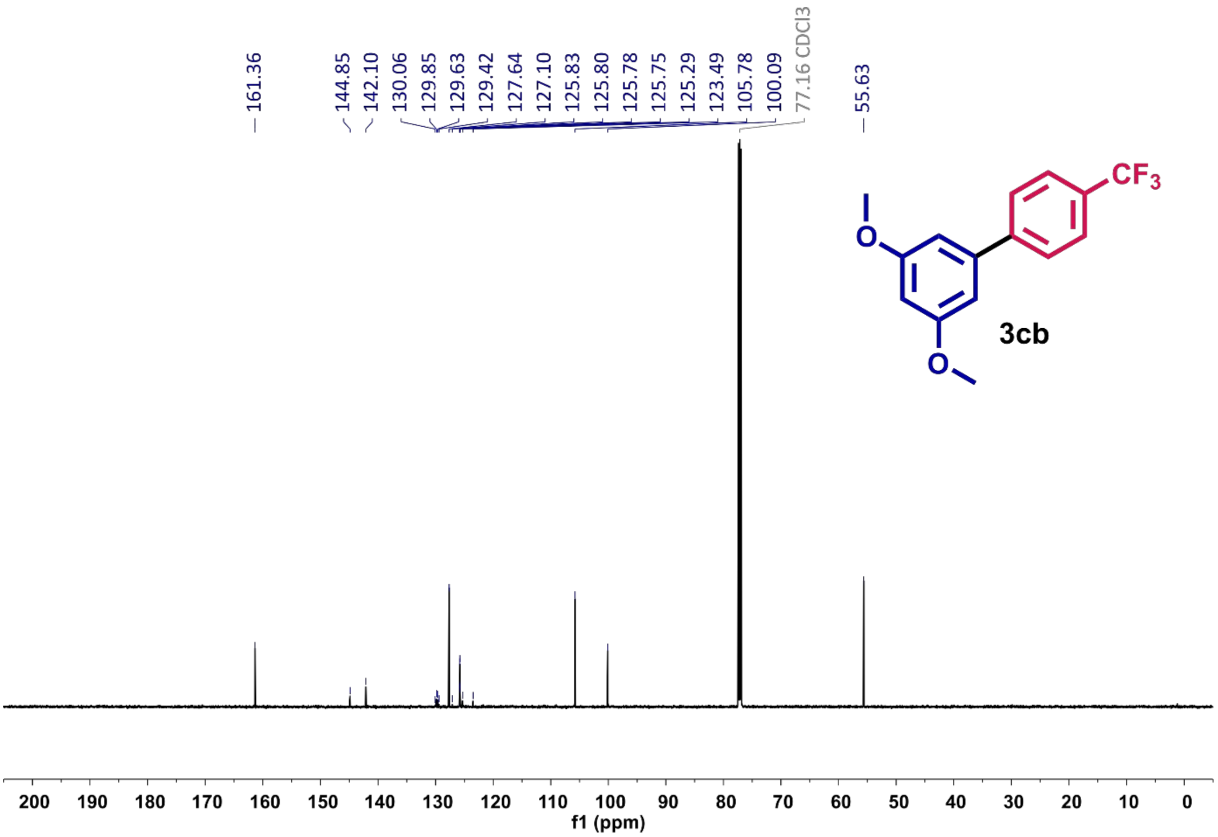

Figure S73. ^{13}C NMR spectrum (151 MHz, $CDCl_3$) of 3,5-dimethoxy-4'-(trifluoromethyl)-biphenyl **3cb**.

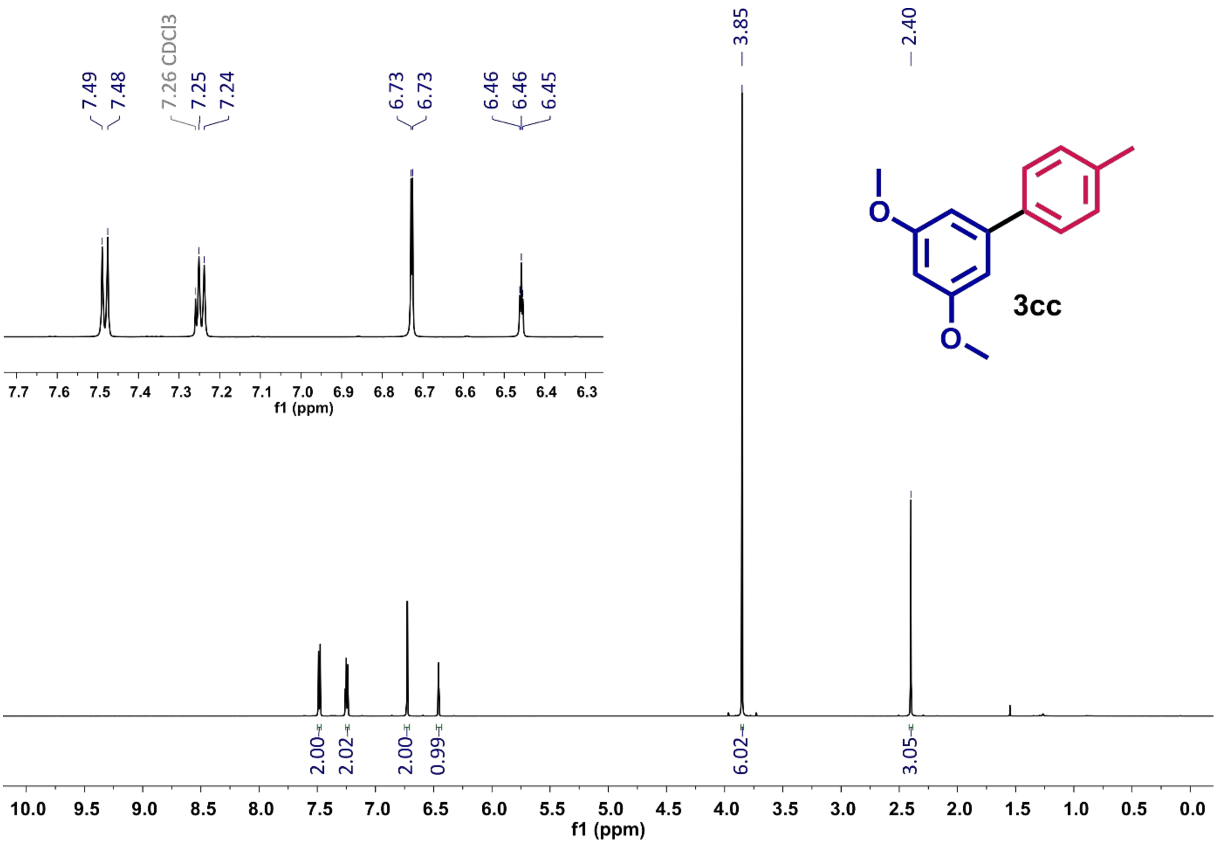

Figure S74. ^{1}H NMR spectrum (600 MHz, $CDCl_3$) of 3,5-dimethoxy-4'-methyl-biphenyl **3cc**.

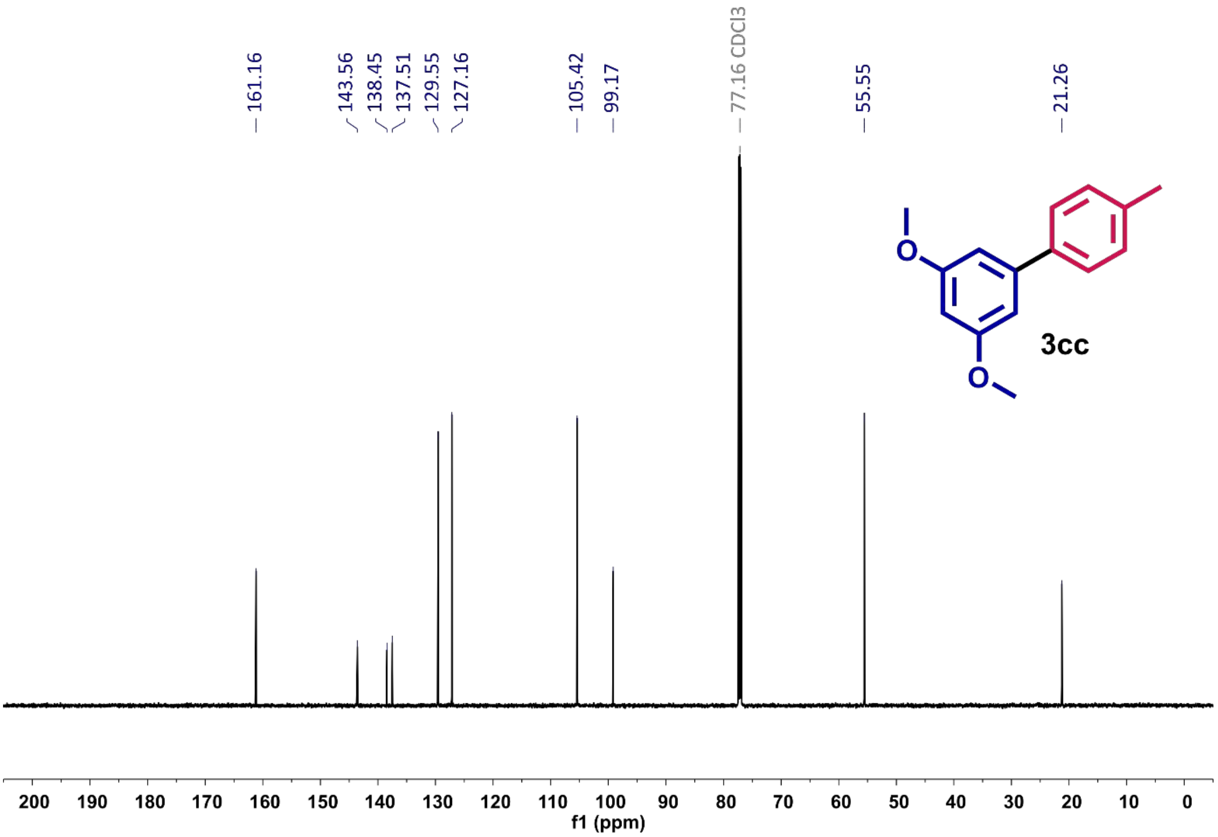

Figure S75. ^{13}C NMR spectrum (151 MHz, $CDCl_3$) of 3,5-dimethoxy-4'-methyl-biphenyl **3cc**.

12. Investigation of catalytic activity in the Suzuki-Miyaura cross-coupling

12.1. Reaction development for the Heck reaction

Table S24. Reaction development for the Heck cross-coupling between styrene and iodobenzene.[a]

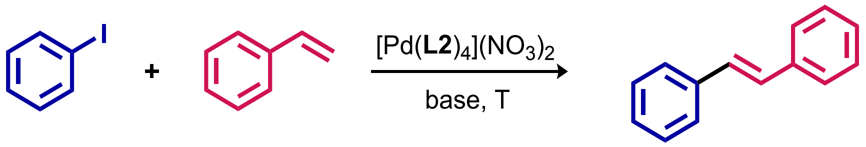

	solvent	base (eq.)	T [°C]	mol% Pd	GC yield [%][b] 1 h	2 h	6 h
1	toluene	K_3PO_4 (2)	80	0.1	0.5	0.7	1
2	toluene	K_3PO_4 (2)	80	1	6	7	10
3	toluene	Et_3N (5)	80	1	20	24	37
4	THF	Et_3N (5)	60	1	7	16	20
5	1,4-dioxane	Et_3N (5)	100	1	40	41	45
6	DMF	Et_3N (5)	120	1	24	49	71
7	DMSO	Et_3N (5)	120	1	86	95	97
**8**	**DMSO**	**Et_3N (5)**	**120**	**0.1**	**82**	**94**	**96**
9	DMSO	Et_3N (5)	120	0.01	25	42	52
10	DMSO	K_3PO_4 (2)	120	0.1	44	45	47
11	DMSO	K_2CO_3 (2)	120	0.1	0	5	8

[a] Reaction conditions: iodobenzene (0.2 mmol, 1 equiv.), styrene (0.24 mmol, 1.2 equiv.), base and the complex [Pd(**L2**)$_4$](NO_3)$_2$ were stirred in appropriate solvent (2 mL) at indicated temperature under air atmosphere. [b] Determined by GC measurement of iodobenzene decay.

12.2. Catalytic activity of Pd(II) complexes based on pyridine ligands in the Heck reaction

Table S25. Catalytic activity of Pd(II) complexes based on pyridine ligands in the Heck cross-coupling between styrene and iodobenzene.[a]

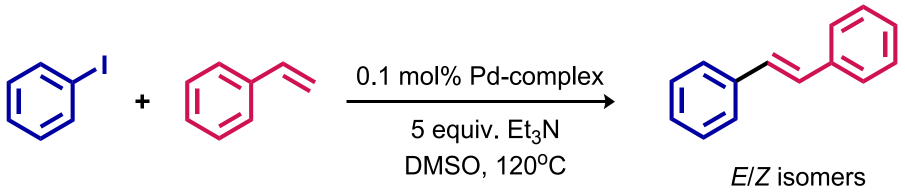

	[Pd**L**$_2$Cl$_2$]		[Pd**L**$_2$(NO_3)$_2$]		[Pd**L**$_4$](NO_3)$_2$	
	GC yield [%][b]	sel. [%][c]	GC yield [%]	sel. [%]	GC yield [%]	sel. [%]
**L1**	85	92	88	91	90	92
**L2**	90	91	91	91	94	91
**L3**	86	91	82	91	76	94
**L4**	89	91	92	92	79	95
**L5**	80	91	92	89	75	93
**L6**	86	91	-	-	83	96
**L7**	90	92	92	91	80	92
**L8**	91	91	93	92	-	-
**L9**	81	92	91	91	-	-
**L10**	93	90	-	-	88	91
**L11**	88	93	-	-	90	89
**L12**	92	91	-	-	77	94

[a] Reaction conditions: iodobenzene (0.2 mmol, 1 equiv.), styrene (0.24 mmol, 1.2 equiv.), Et_3N (1.0 mmol, 5 equiv.) and Pd(II) complex (0.1 mol%) were stirred in DMSO (2 mL) at 120°C under air atmosphere for 2 h. [b] Determined by GC measurement of iodobenzene decay as the average of three results. [c] Determined by GC analysis with the area normalization method.

12.3. General synthetic procedure for the Heck cross-coupling

A reaction vessel equipped with a stirring bar was charged with aryl iodide **4a-c** (1.0 mmol, 1.0 equiv.) and olefin **5a-c** (1.2 mmol, 1.2 equiv.) which were dissolved in DMSO (10 mL). After, the catalyst [Pd(**L2**)$_4$](NO_3)$_2$ (0.001 mmol, 0.001 equiv.) as a solution in DMSO (0.05 mL) and Et_3N (5.0 mmol, 5.0 equiv.) were added. The vial was sealed and the reaction mixture was heated for 6 h at 120°C. The resulting solution was then cooled to room temperature, diluted with ethyl acetate (50 mL) and washed with icy distilled water (40 mL). The collected aqueous phase was extracted with ethyl acetate (2 × 50 mL). The organic layers were gathered, dried over Na_2SO_4, filtered and the solvent was removed under reduced pressure. The residue was purified by column chromatography on silica gel to obtain the desired products **6aa-cc**.

12.4. Characterization of the cross-coupling products

12.4.1. (*E*)-stilbene (6aa)[18]

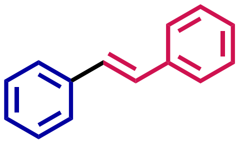

The reaction of iodobenzene **4a** (1 mmol, 112 μL) with styrene **5a** (1.2 mmol, 138 μL) according to the general procedure (flash chromatography: hexane) gave (*E*)-stilbene **6aa** in the form of white solid. Yield: 89%, 160 mg.

^{1}H NMR (600 MHz, $CDCl_3$) δ = 7.54 (d, *J* = 7.3 Hz, 4H), 7.38 (t, *J* = 7.7 Hz, 4H), 7.28 (t, *J* = 7.4 Hz, 2H), 7.13 (s, 2H).

^{13}C NMR (151 MHz, $CDCl_3$) δ = 137.47, 128.83, 128.82, 127.76, 126.65.

12.4.2. Methyl (*E*)-cinnamate (6ab)[18]

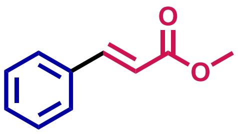

The reaction of iodobenzene **4a** (1 mmol, 112 μL) with methyl acrylate **5b** (1.2 mmol, 109 μL) according to the general procedure (flash chromatography: hexane/ethyl acetate 10:1) gave methyl (*E*)-cinnamate **6ab** in the form of light yellow solid. Yield: 90%, 146 mg.

^{1}H NMR (600 MHz, $CDCl_3$) δ = 7.70 (d, *J* = 16.0 Hz, 1H), 7.53 – 7.52 (m, 2H), 7.39 – 7.38 (m, 2H), 6.45 (d, *J* = 16.0 Hz, 1H), 3.81 (s, 3H).

^{13}C NMR (151 MHz, $CDCl_3$) δ = 167.56, 145.00, 134.52, 130.43, 129.02, 128.20, 117.94, 51.84.

12.4.3. (*E*)-4-bromostilbene (6ba)[19]

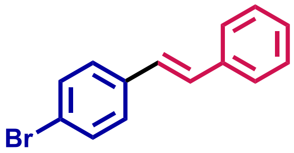

The reaction of 1-bromo-4-iodobenzene **4b** with styrene **5a** (1.2 mmol, 138 μL) according to the general procedure (flash chromatography: hexane) gave (*E*)-4-bromostilbene **6ba** in the form of white solid. Yield: 78%, 202 mg.

^{1}H NMR (600 MHz, $CDCl_3$) δ = 7.51 (d, *J* = 7.2 Hz, 2H), 7.48 (d, *J* = 8.5 Hz, 2H), 7.39 – 7.37 (m, 4H), 7.29 (t, *J* = 6.7 Hz, 1H), 7.10 (d, *J* = 16.3 Hz, 1H), 7.04 (d, *J* = 16.3 Hz, 1H).

^{13}C NMR (151 MHz, $CDCl_3$) δ = 137.19, 136.52, 132.02, 129.67, 128.99, 128.21, 128.15, 127.64, 126.80, 121.55.

12.4.4. Methyl (*E*)-4-bromocinnamate (6bb)[20]

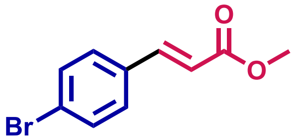

The reaction of 1-bromo-4-iodobenzene **4b** (1 mmol, 283 mg) with methyl acrylate **5b** (1.2 mmol, 109 µL) according to the general procedure (flash chromatography: hexane/ethyl acetate 20:1) gave methyl (*E*)-4-bromocinnamate **6bb** in the form of pale yellow solid. Yield: 91%, 219 mg.

^{1}H NMR (600 MHz, $CDCl_3$) δ = 7.62 (d, *J* = 16.0 Hz, 1H), 7.52 (d, *J* = 8.4 Hz, 2H), 7.38 (d, *J* = 8.5 Hz, 2H), 6.42 (d, *J* = 16.0 Hz, 1H), 3.81 (s, 3H).

^{13}C NMR (151 MHz, $CDCl_3$) δ = 167.29, 143.62, 133.43, 132.29, 129.58, 124.69, 118.64, 51.95.

12.4.5. (*E*)-4,4'-dibromostilbene (6bc)[21]

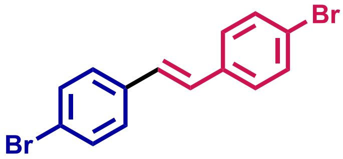

The reaction of 1-bromo-4-iodobenzene **4b** (1 mmol, 283 mg) with 4-bromostyrene **5c** (1.2 mmol, 157 µL) according to the general procedure (flash chromatography: hexane) gave (*E*)-4,4'-dibromostilbene **6bc** in the form of white solid. Yield: 74%, 250 mg.

^{1}H NMR (600 MHz, $CDCl_3$) δ = 7.48 (d, *J* = 8.5 Hz, 4H), 7.36 (d, *J* = 8.5 Hz, 4H), 7.02 (s, 2H).

^{13}C NMR (151 MHz, $CDCl_3$) δ = 136.05, 132.00, 128.27, 128.15, 121.79.

12.4.6. (*E*)-4-acetylstilbene (6ca)[22]

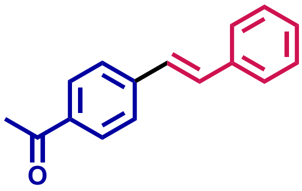

The reaction of 4-iodoacetophenone **4c** (1 mmol, 222 mg) with styrene **5a** (1.2 mmol, 138 µL) according to the general procedure (flash chromatography: hexane/ethyl acetate 15:1 to 10:1) gave (*E*)-4-acetylstilbene **6ca** in the form of white solid. Yield: 55%, 122 mg.

^{1}H NMR (600 MHz, $CDCl_3$) δ = 7.96 (d, *J* = 8.4 Hz, 2H), 7.59 (d, *J* = 8.4 Hz, 2H), 7.54 (d, *J* = 7.4 Hz, 2H), 7.39 (t, *J* = 7.7 Hz, 2H), 7.31 (t, *J* = 7.3 Hz, 1H), 7.23 (d, *J* = 16.3 Hz, 1H), 7.14 (d, *J* = 16.3 Hz, 1H), 2.61 (s, 3H).

^{13}C NMR (151 MHz, $CDCl_3$) δ = 197.59, 142.14, 136.83, 136.09, 131.59, 129.01, 128.93, 128.45, 127.58, 126.95, 126.63, 26.74.

12.4.7. Methyl (*E*)-4-acetylcinnamate (6cb)[23]

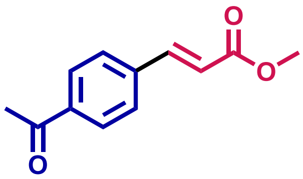

The reaction of 4-iodoacetophenone **4c** (1 mmol, 222 mg) with methyl acrylate **5b** (1.2 mmol, 109 µL) according to the general procedure (flash chromatography: hexane/ethyl acetate 8:2) gave methyl (*E*)-4-acetylcinnamate **6cb** in the form of pale yellow solid. Yield: 88%, 180 mg.

^{1}H NMR (600 MHz, $CDCl_3$) δ = 7.96 (d, *J* = 8.4 Hz, 2H), 7.70 (d, *J* = 16.0 Hz, 1H), 7.60 (d, *J* = 8.4 Hz, 2H), 6.52 (d, *J* = 16.0 Hz, 1H), 3.82 (s, 3H), 2.61 (s, 3H).

^{13}C NMR (151 MHz, $CDCl_3$) δ = 197.40, 167.04, 143.42, 138.82, 138.17, 128.98, 128.27, 120.46, 52.04, 26.82.

12.4.8. (*E*)-4-acetyl-4’-bromostilbene (6cc)

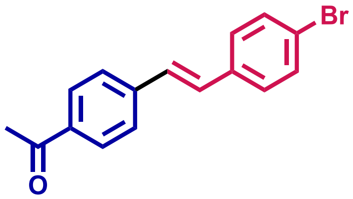

The reaction of 4-iodoacetophenone **4c** (1 mmol, 222 mg) with 4-bromostyrene **5c** (1.2 mmol, 157 µL) according to the general procedure (flash chromatography: hexane/ethyl acetate 9:1 to 8:2) gave (*E*)-4-acetyl-4’-bromostilbene **6cc** in the form of pale yellow solid. Yield: 80%, 241 mg.

^{1}H NMR (600 MHz, $CDCl_3$) δ = 7.95 (d, J = 8.4 Hz, 2H), 7.57 (d, J = 8.4 Hz, 2H), 7.50 (d, J = 8.5 Hz, 2H), 7.40 (d, J = 8.5 Hz, 2H), 7.15 (d, J = 16.4 Hz, 1H), 7.11 (d, J = 16.3 Hz, 1H), 2.61 (s, 3H).

^{13}C NMR (151 MHz, $CDCl_3$) δ = 197.56, 141.72, 136.30, 135.78, 132.07, 130.26, 129.05, 128.38, 128.29, 126.70, 122.27, 26.75.

12.5. NMR spectra of the cross-coupling products

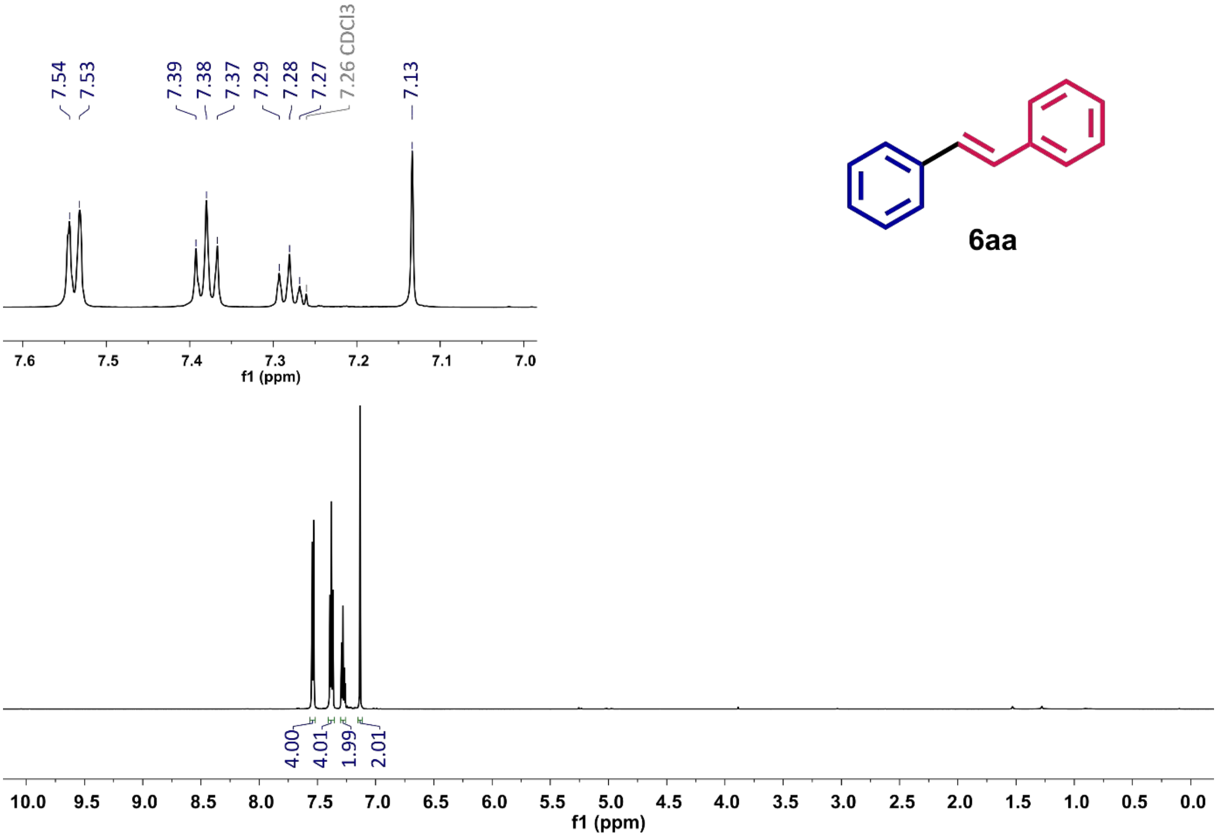

Figure S76. ^{1}H NMR spectrum (600 MHz, $CDCl_3$) of (*E*)-stilbene **6aa**.

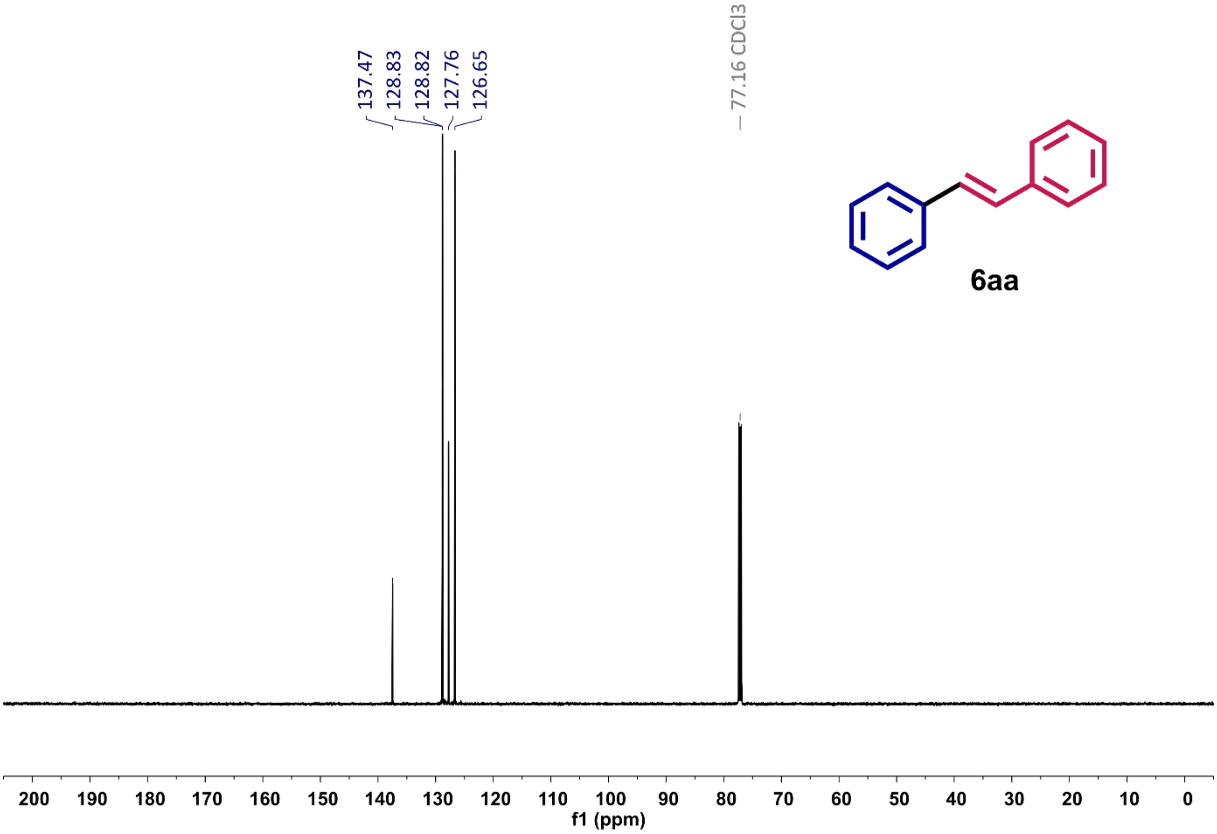

Figure S77. ^{13}C NMR spectrum (151 MHz, $CDCl_3$) of (*E*)-stilbene **6aa**.

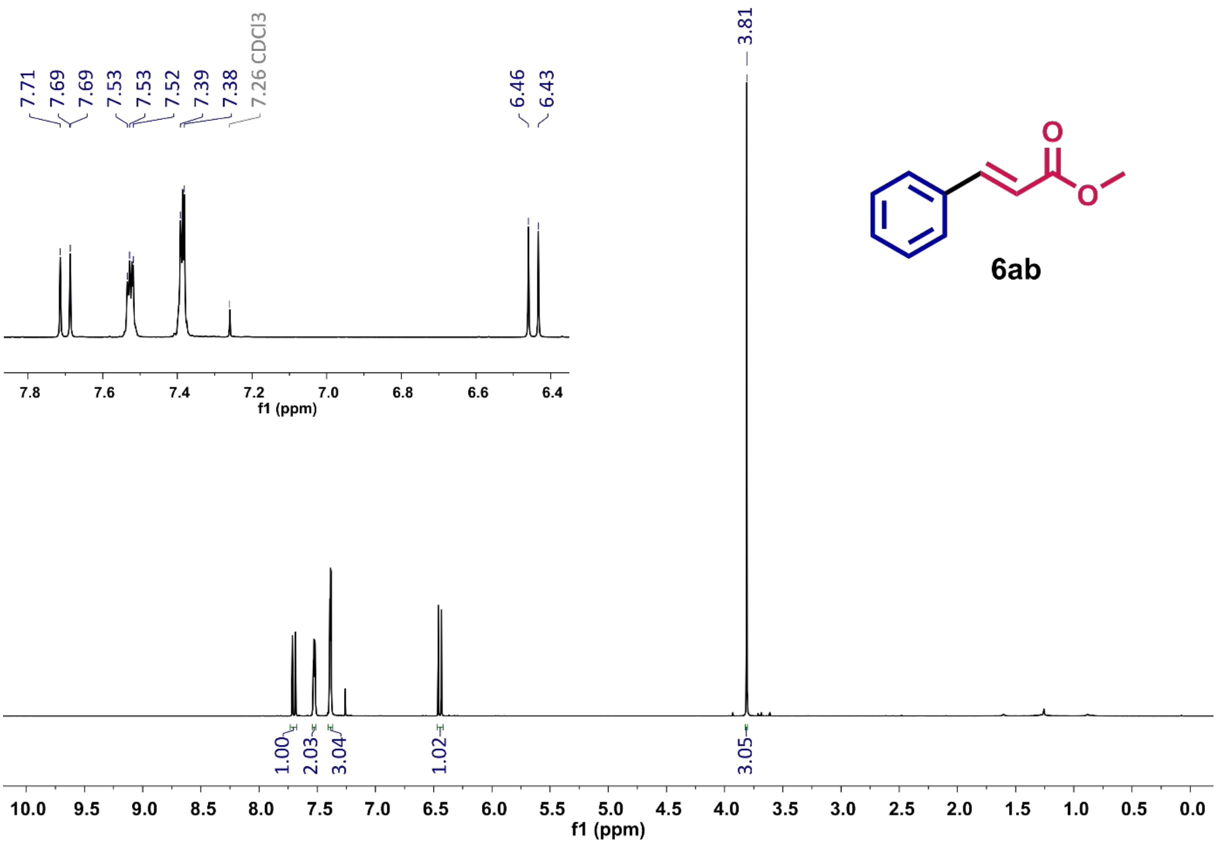

Figure S78. ^{1}H NMR spectrum (600 MHz, $CDCl_3$) of methyl (*E*)-cinnamate **6ab**.

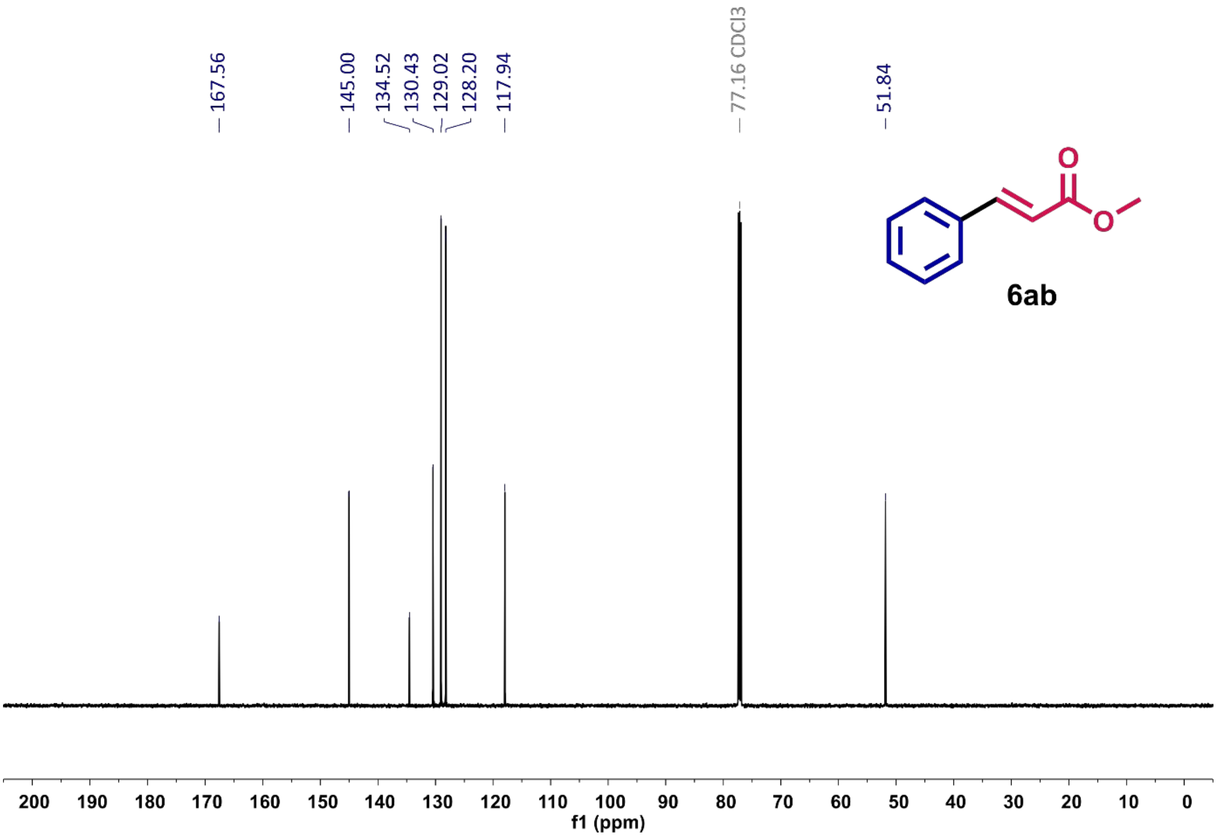

Figure S79. ^{13}C NMR spectrum (151 MHz, $CDCl_3$) of methyl (*E*)-cinnamate **6ab**.

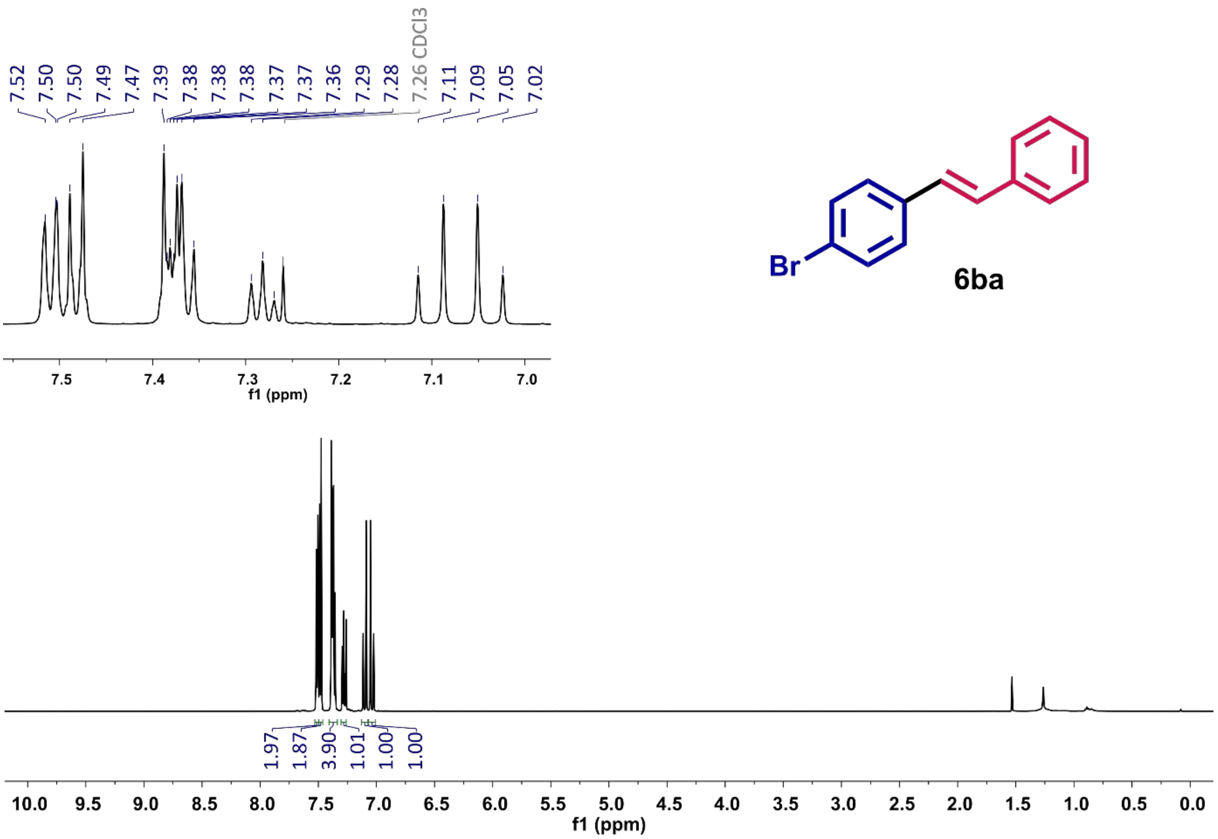

Figure S80. ^{1}H NMR spectrum (600 MHz, $CDCl_3$) of (*E*)-4-bromostilbene **6ba**.

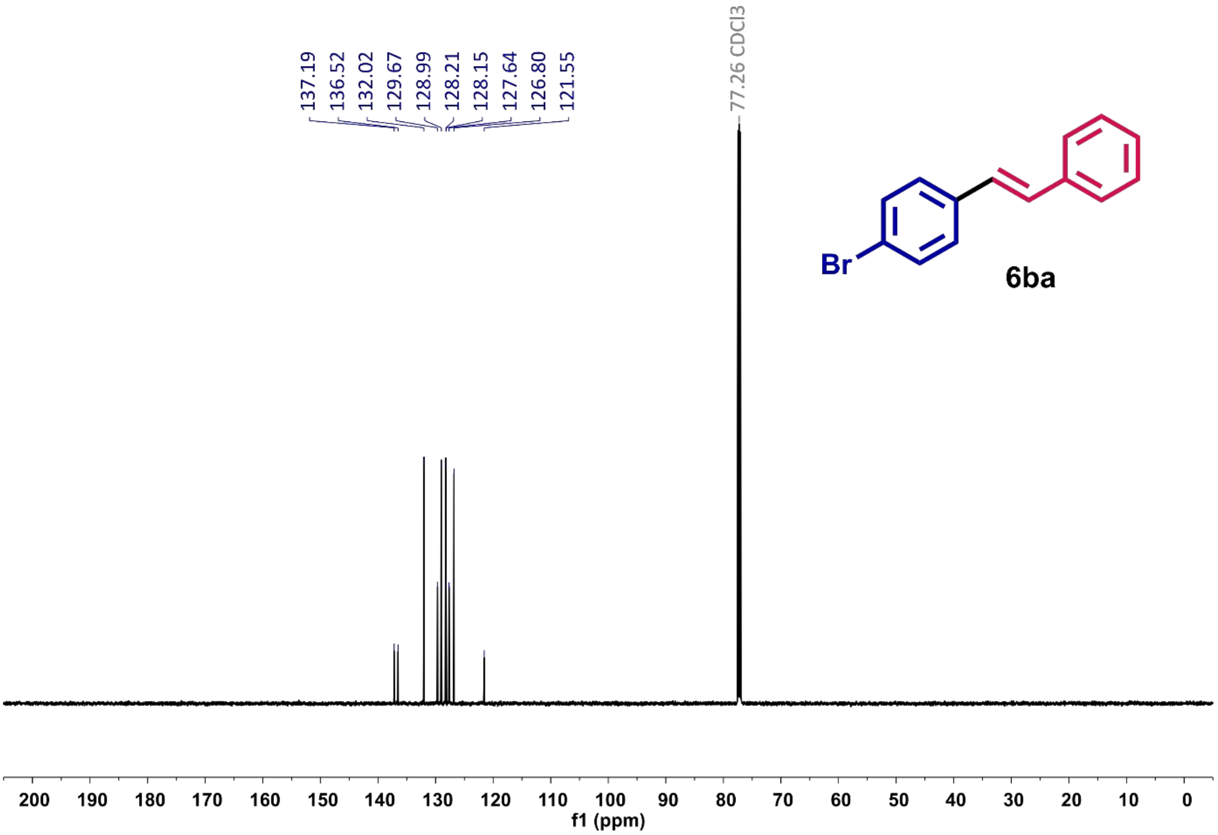

Figure S81. ^{13}C NMR spectrum (151 MHz, $CDCl_3$) of methyl (*E*)-4-bromostilbene **6ba**.

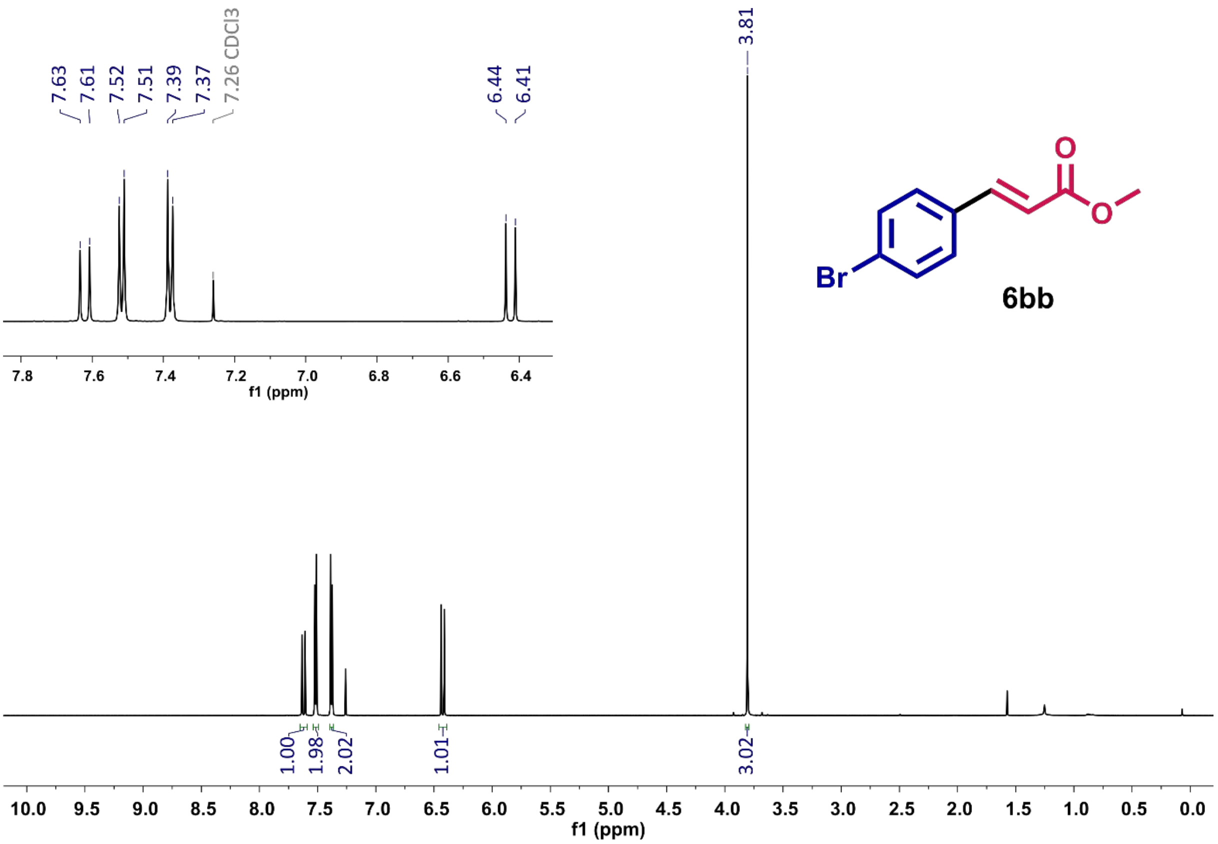

Figure S82. ^{1}H NMR spectrum (600 MHz, $CDCl_3$) of methyl (*E*)-4-bromocinnamate **6bb**.

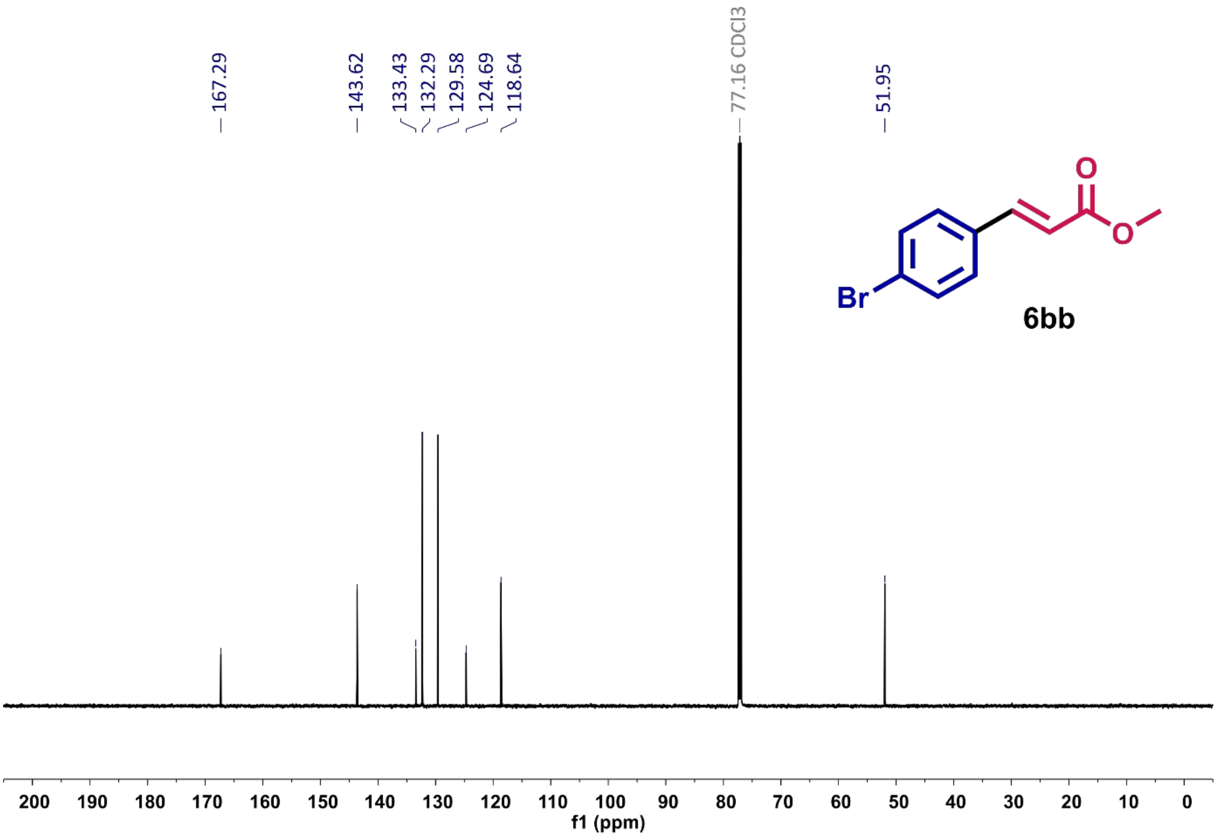

Figure S83. ^{13}C NMR spectrum (151 MHz, $CDCl_3$) of methyl (*E*)-4-bromocinnamate **6bb**.

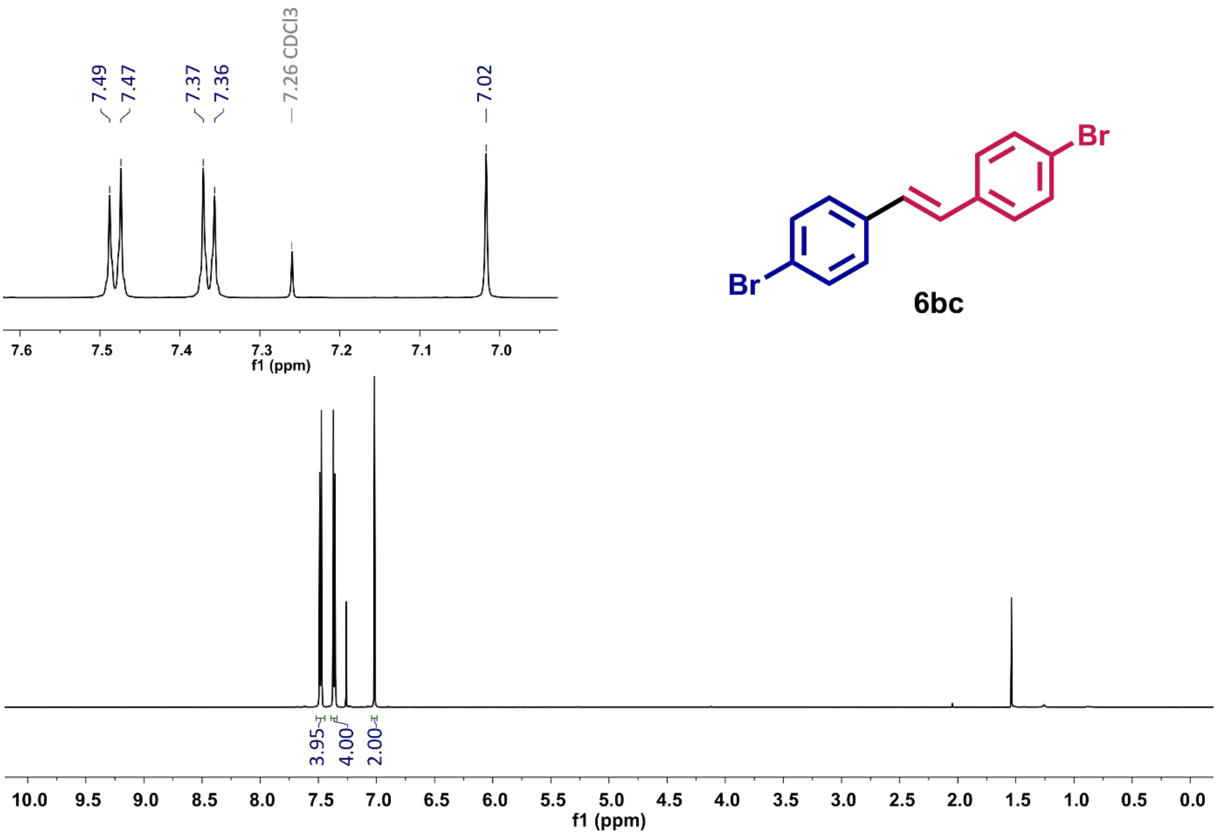

Figure S84. ^{1}H NMR spectrum (600 MHz, $CDCl_3$) of (*E*)-4,4'-dibromostilbene **6bc**.

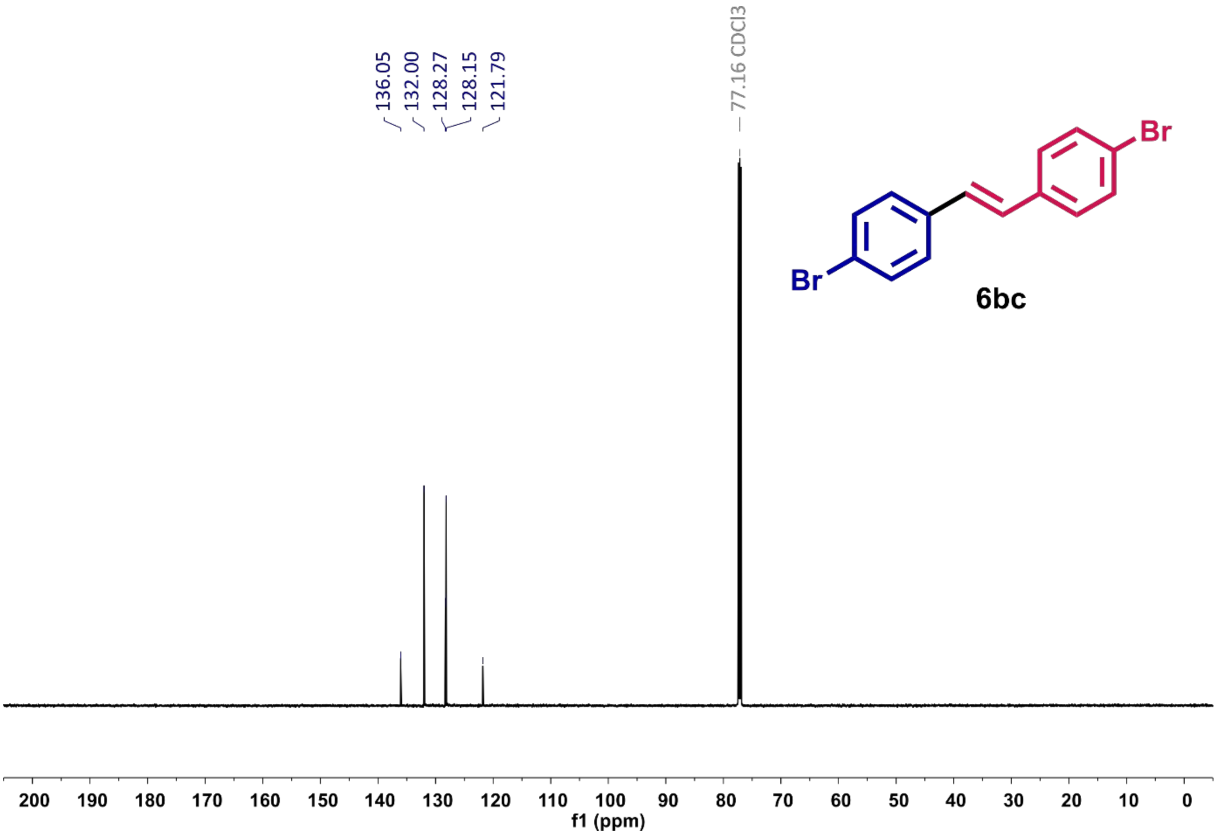

Figure S85. ^{13}C NMR spectrum (151 MHz, $CDCl_3$) of (*E*)-4,4'-dibromostilbene **6bc**.

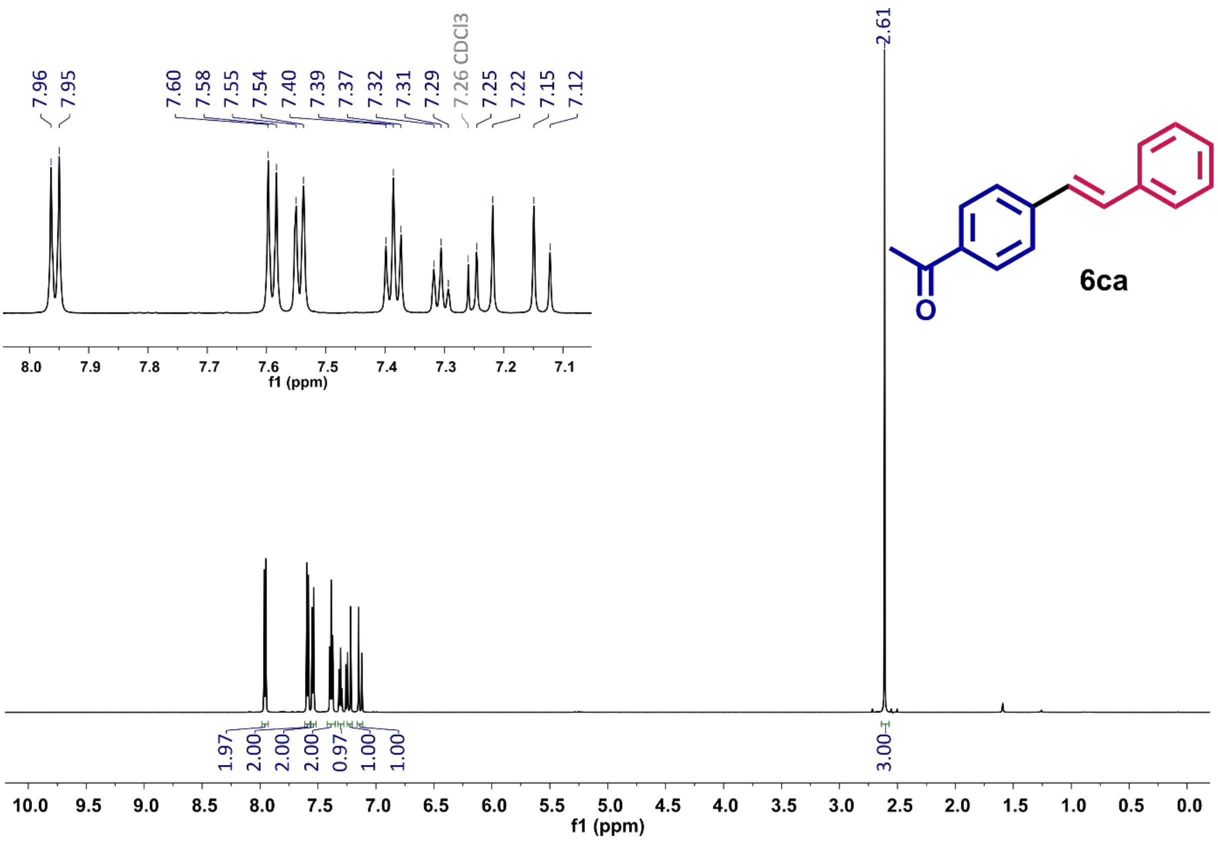

Figure S86. ^{1}H NMR spectrum (600 MHz, $CDCl_3$) of (*E*)-4-acetylstilbene **6ca**.

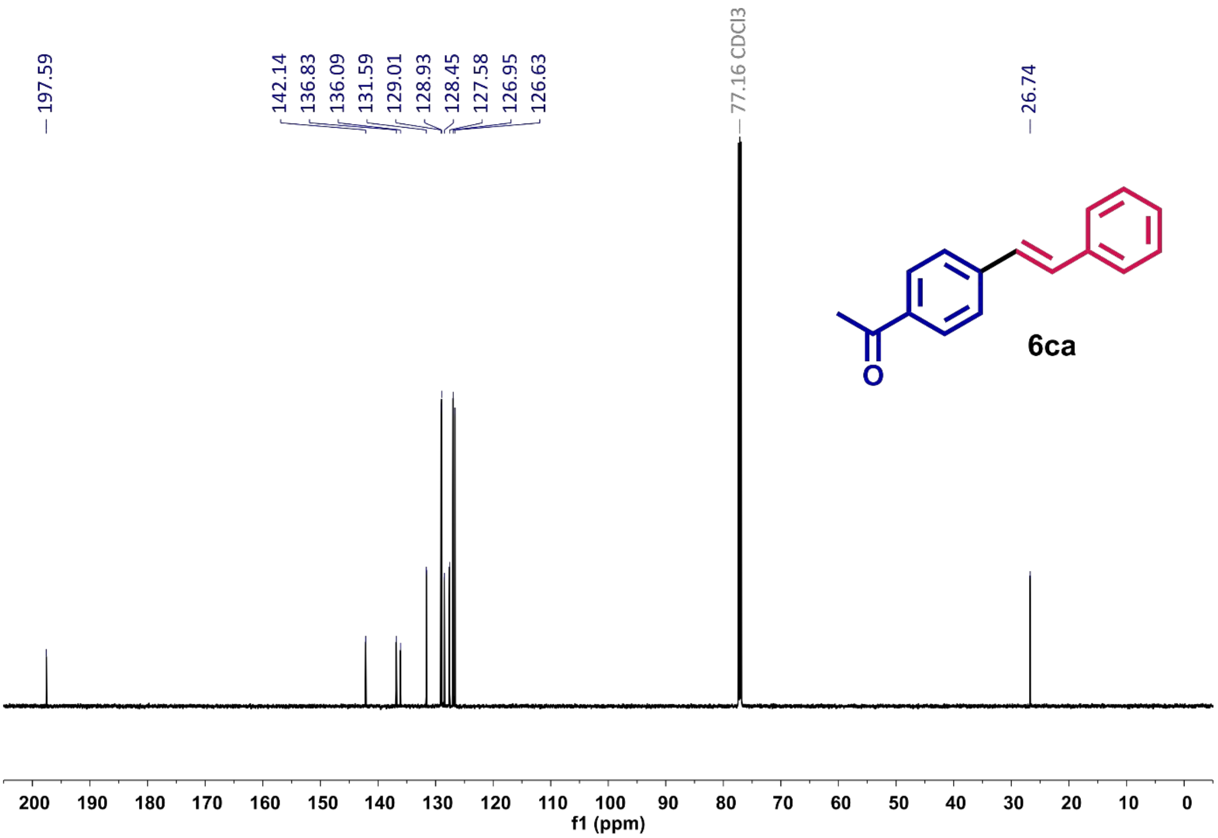

Figure S87. ^{13}C NMR spectrum (151 MHz, $CDCl_3$) of (*E*)-4-acetylstilbene **6ca**.

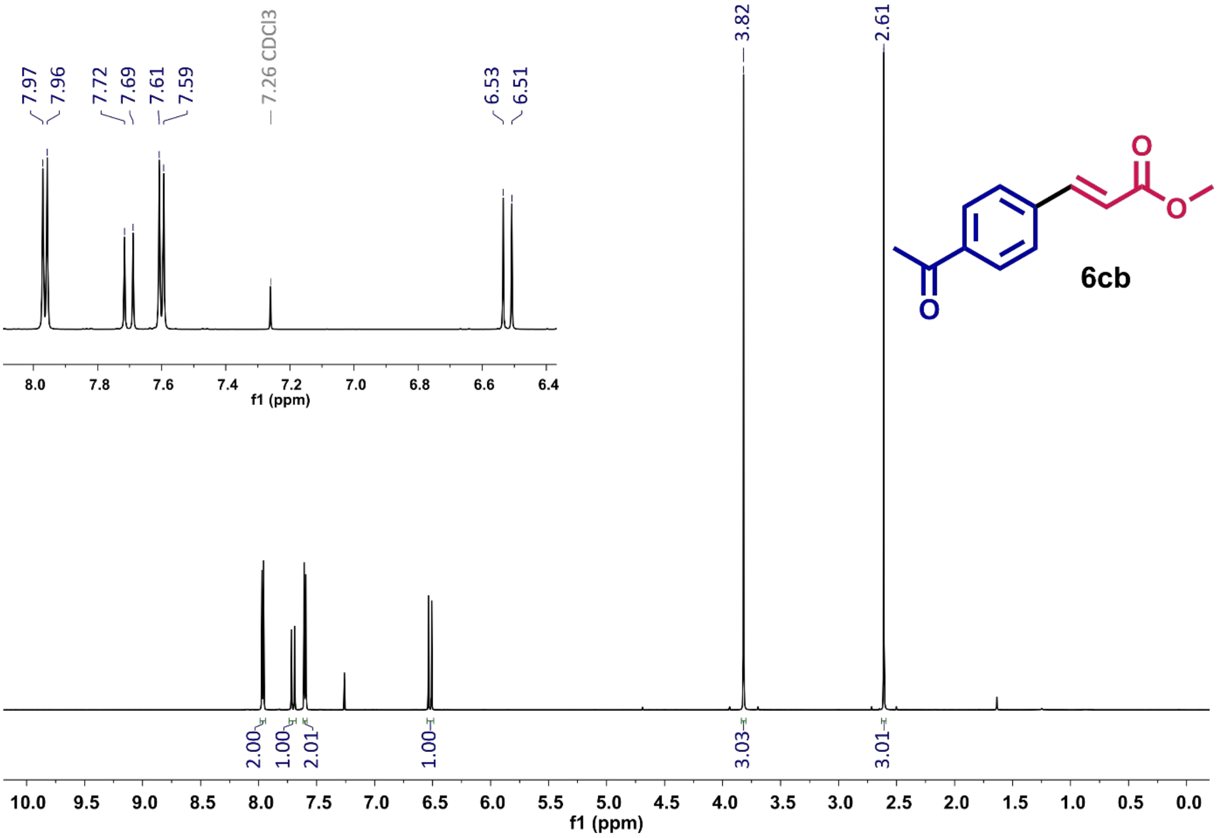

Figure S88. ^{1}H NMR spectrum (600 MHz, $CDCl_3$) of methyl (*E*)-4-acetylcinnamate **6cb**.

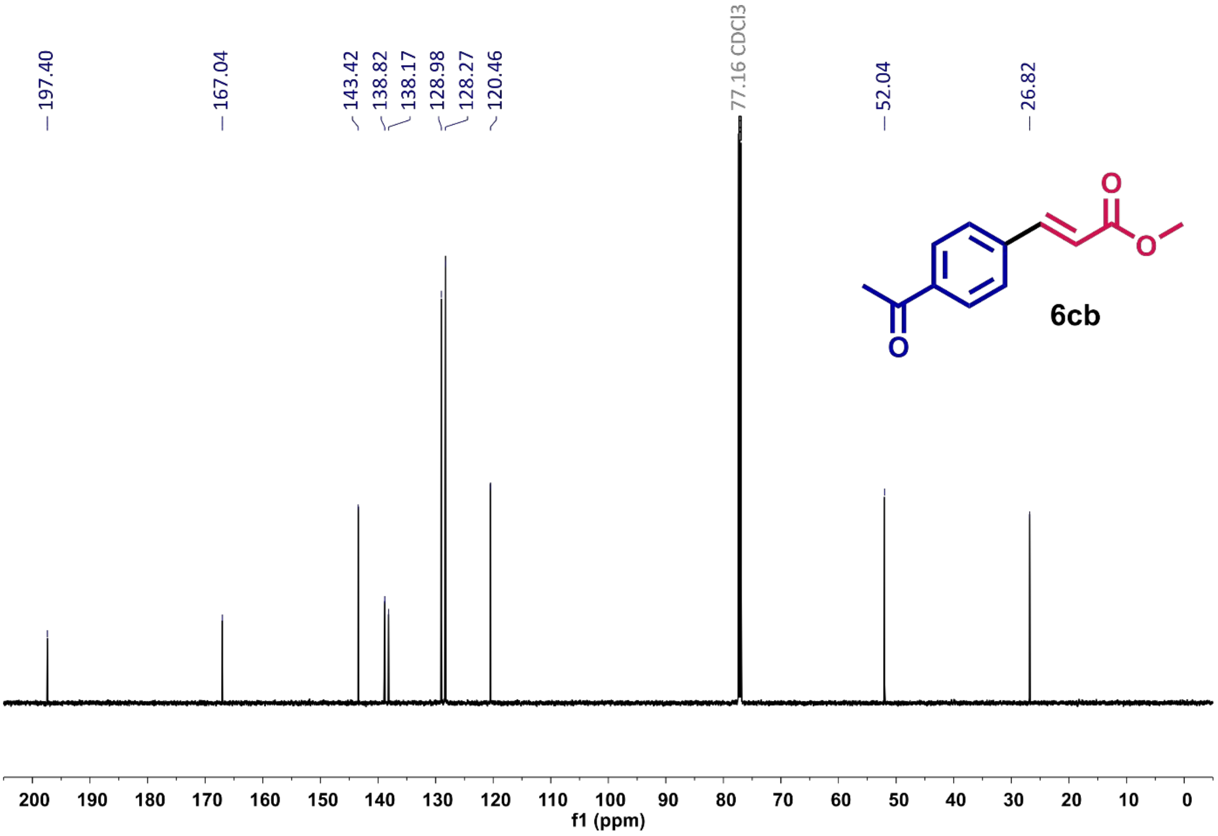

Figure S89. ^{13}C NMR spectrum (151 MHz, $CDCl_3$) of methyl (*E*)-4-acetylcinnamate **6cb**.

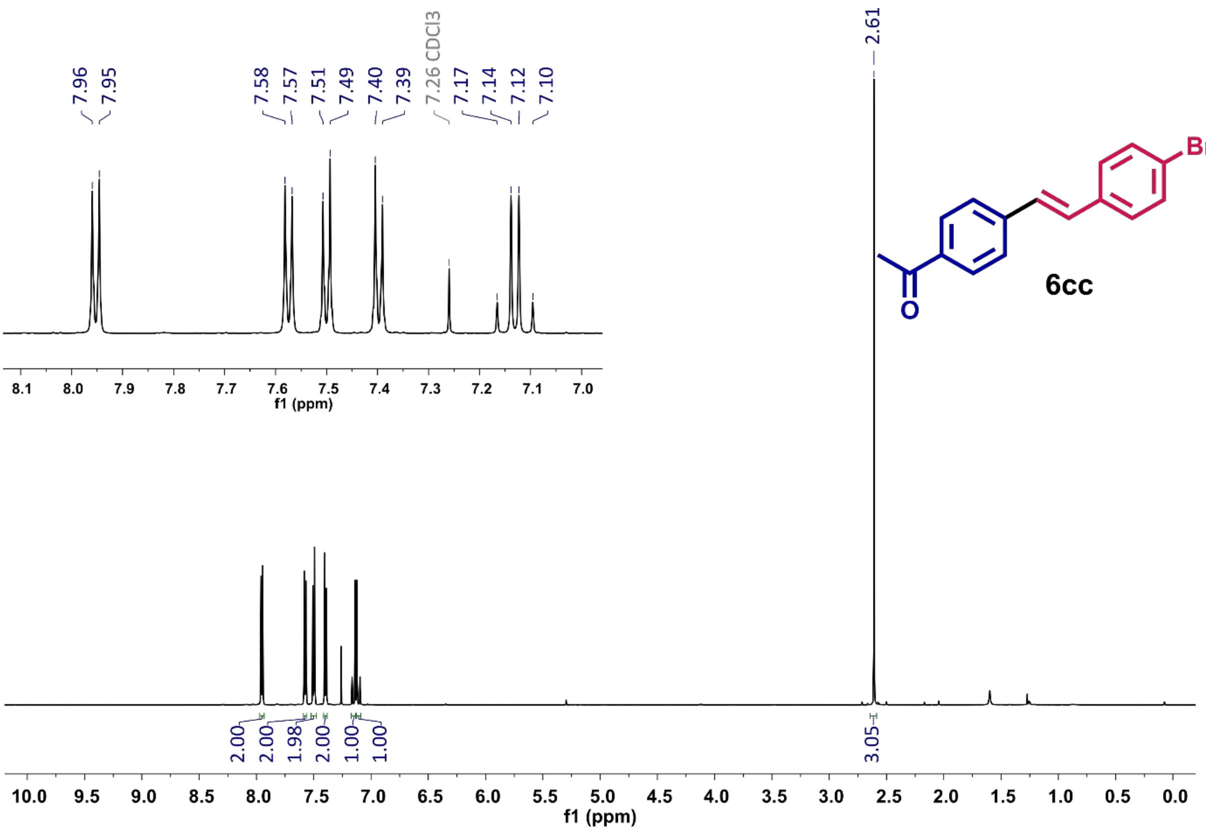

Figure S90. ^{1}H NMR spectrum (600 MHz, $CDCl_3$) of (*E*)-4-acetyl-4'-bromostilbene **6cc**.

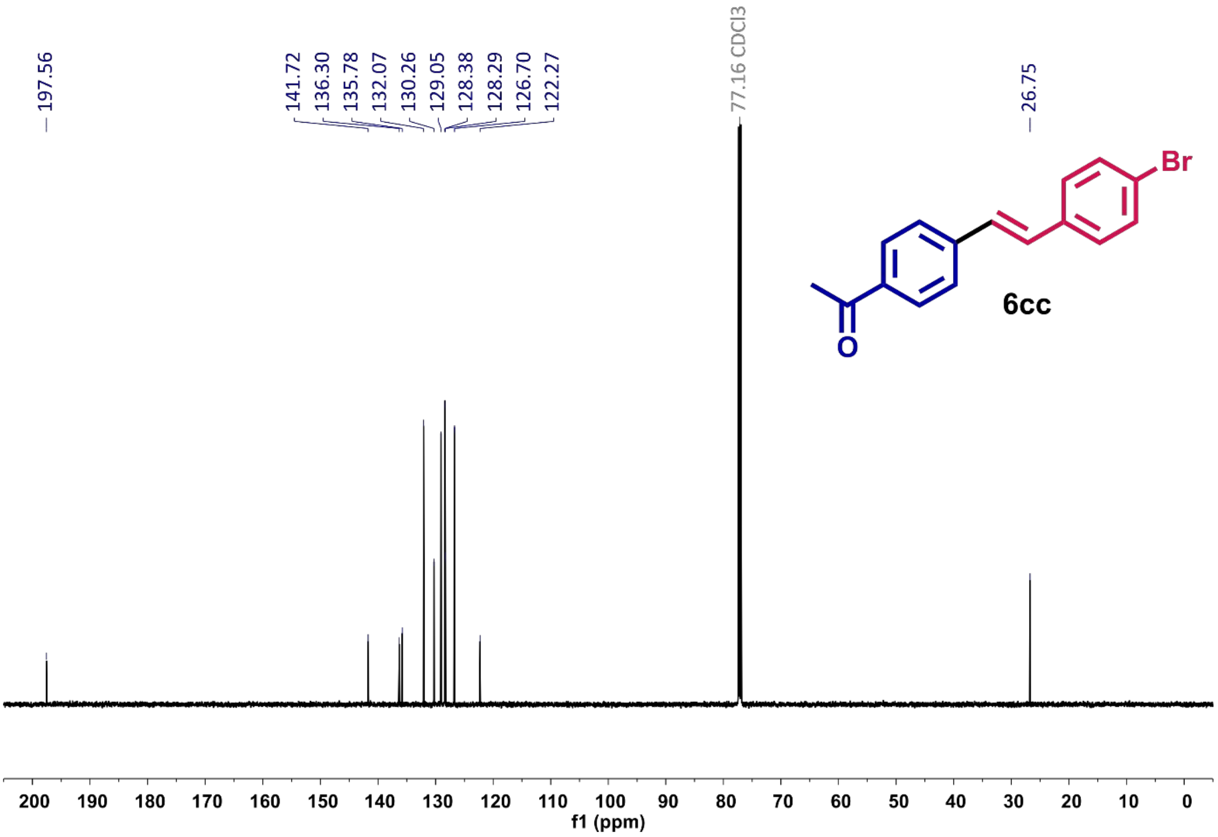

Figure S91. ^{13}C NMR spectrum (151 MHz, $CDCl_3$) of (*E*)-4-acetyl-4'-bromostilbene **6cc**.

13. Influence of the type of pyridine ligands and the nature of Pd(II) complexes on GC yields in catalyzed reactions

13.1. The relationship between GC yield and the character of Pd(II) complex in the Suzuki-Miyaura cross-coupling

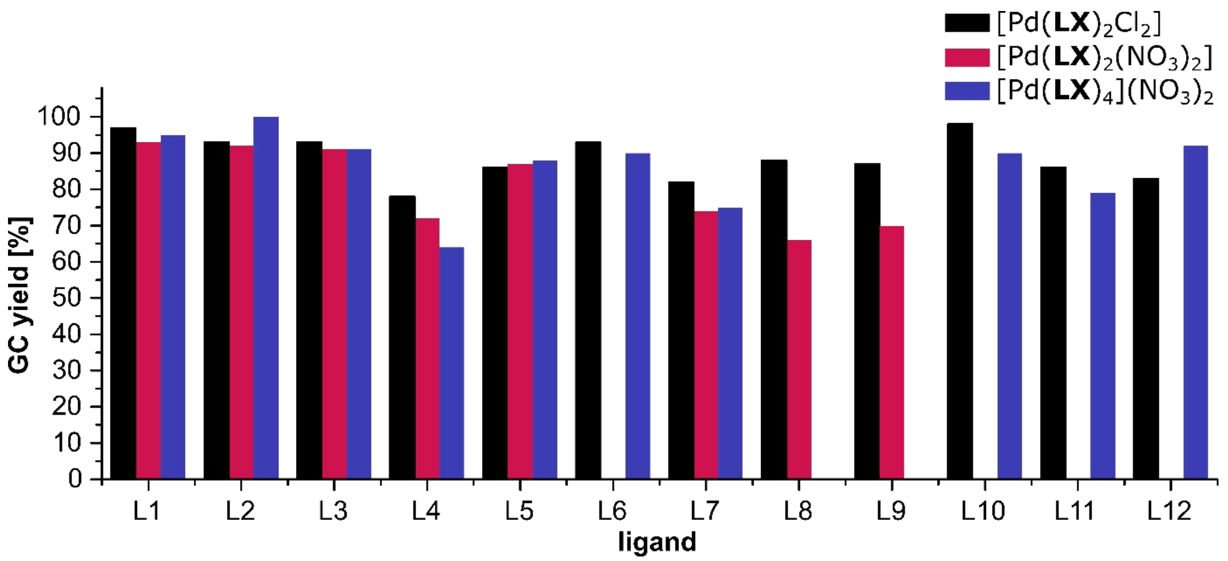

Figure S92. The relationship between GC yield and the character of Pd(II) complex in the catalyzed Suzuki-Miyaura cross-coupling reaction. The data were collected for reactions performed for 2 h.

13.2. The relationship between GC yield and the ligand basicity in the Suzuki-Miyaura cross-coupling

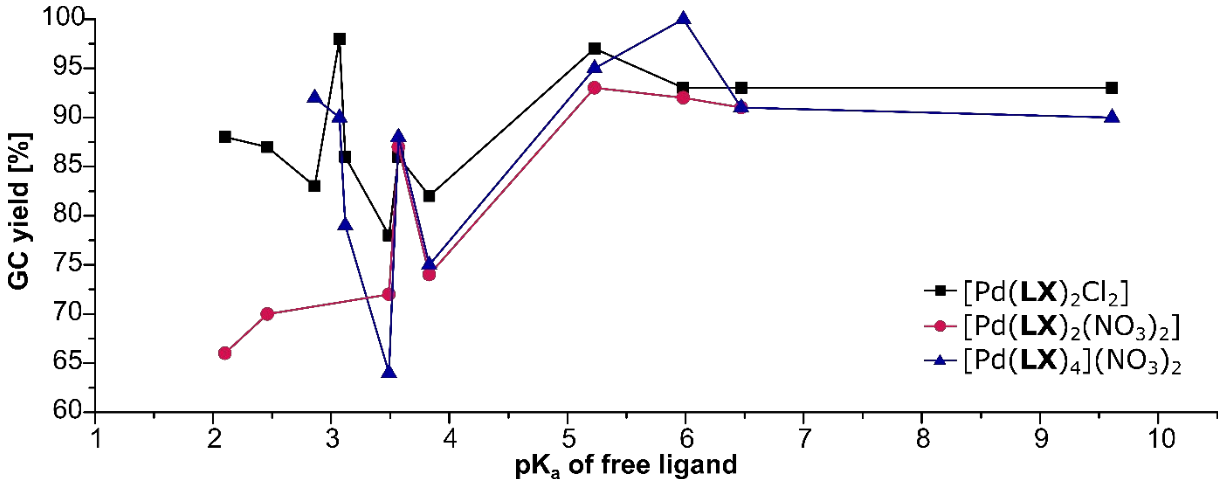

Figure S93. The relationship between GC yield and the pKa values of the pyridine ligands in the catalyzed Suzuki-Miyaura cross-coupling reaction. The data were collected for reactions performed for 2 h. For ligands **L10-L12**, predicted pKa values provided by SciFinder.

13.3. The relationship between GC yield and the character of Pd(II) complex in the Heck cross-coupling

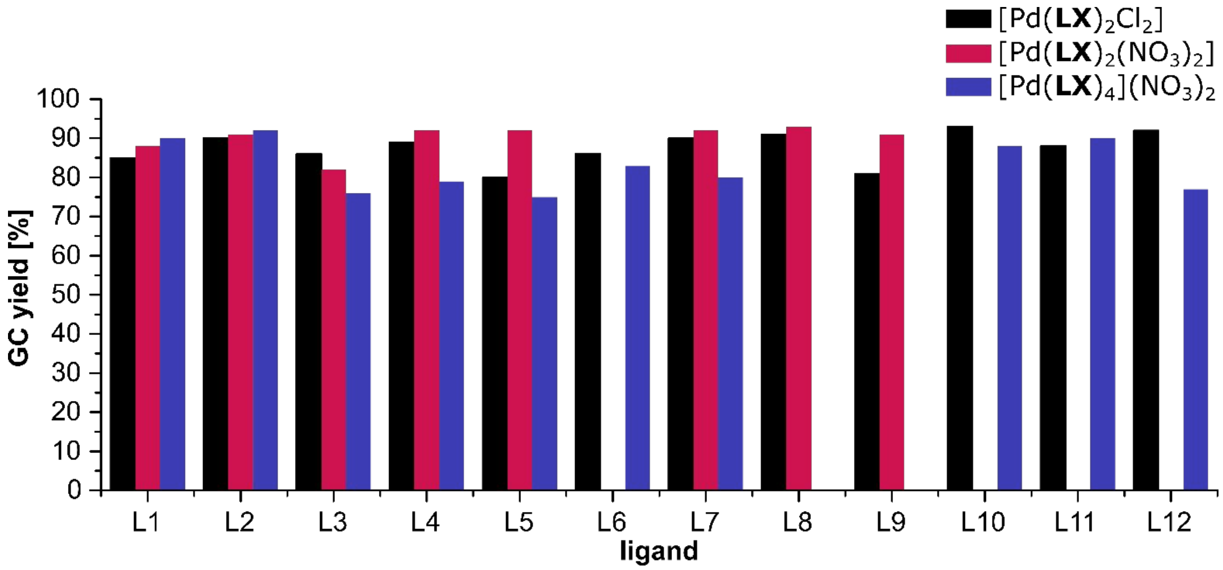

Figure S94. The relationship between GC yield and the character of Pd(II) complex in the catalyzed Heck cross-coupling reaction. The data were collected for reactions performed for 2 h.

13.4. The relationship between GC yield and the ligand basicity in the Heck cross-coupling

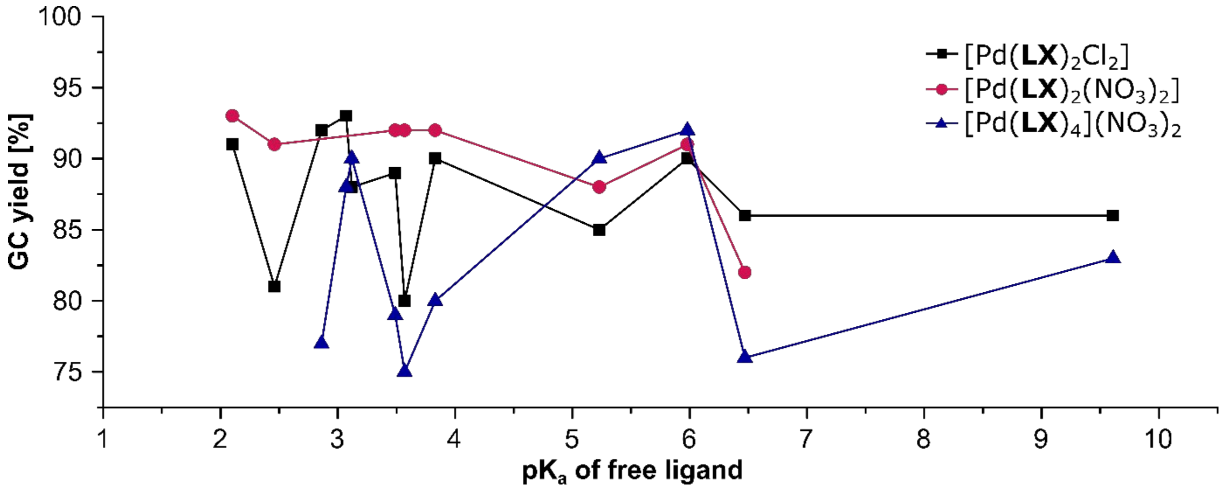

Figure S95. The relationship between GC yield and the pKa values of the pyridine ligands in the catalyzed Heck cross-coupling reaction. The data were collected for reactions performed for 2 h. For ligands **L10-L12**, predicted pKa values provided by SciFinder.

14. References

1. Martins, F. J.; Lima, R. M.; Santos, J. A. d.; Machado, P. d. A.; Coimbra, E. S.; Silva, A. D. d.; Raposo, N. R. B., Biological Properties of Heterocyclic Pyridinylimines and Pyridinylhydrazones. *Lett. Drug. Des. Discov.* **2016,** *13*, 107-114.
2. Sirois, J. J.; Davis, R.; DeBoef, B., Iron-Catalyzed Arylation of Heterocycles via Directed C–H Bond Activation. *Org. Lett.* **2014,** *16* (3), 868-871.

3. Beck, D. E.; Reddy, P. V. N.; Lv, W.; Abdelmalak, M.; Tender, G. S.; Lopez, S.; Agama, K.; Marchand, C.; Pommier, Y.; Cushman, M., Investigation of the Structure–Activity Relationships of Aza-A-Ring Indenoisoquinoline Topoisomerase I Poisons. *J. Med. Chem.* **2016,** *59* (8), 3840-3853.
4. Sato, T.; Yoshida, T.; Al Mamari, H. H.; Ilies, L.; Nakamura, E., Manganese-Catalyzed Directed Methylation of C(sp2)–H Bonds at 25 °C with High Catalytic Turnover. *Org. Lett.* **2017,** *19* (19), 5458-5461.
5. Walczak, A.; Stefankiewicz, A. R., pH-Induced Linkage Isomerism of Pd(II) Complexes: A Pathway to Air- and Water-Stable Suzuki–Miyaura-Reaction Catalysts. *Inorg. Chem.* **2018,** *57* (1), 471-477.
6. Farrugia, L., WinGX and ORTEP for Windows: an update. *J. Appl. Crystallogr.* **2012,** *45* (4), 849-854.
7. Dolomanov, O. V.; Bourhis, L. J.; Gildea, R. J.; Howard, J. A. K.; Puschmann, H., OLEX2: a complete structure solution, refinement and analysis program. *J. Appl. Crystallogr.* **2009,** *42* (2), 339-341.
8. J. Bruns, M. S.; Wickleder, M. S., *Z. Anorg. Allg. Chem.* **2014,** *640*, 2344.
9. Fronczek, F. R., Experimental Crystal Structure Determination CCDC 1402174, 2015.
10. Krogul, A.; Cedrowski, J.; Wiktorska, K.; Ozimiński, W. P.; Skupińska, J.; Litwinienko, G., Crystal structure, electronic properties and cytotoxic activity of palladium chloride complexes with monosubstituted pyridines. *Dalton Trans.* **2012,** *41* (2), 658-666.
11. Zordan, F.; Brammer, L., M−X···X'−C Halogen-Bonded Network Formation in MX2(4-halopyridine)2 Complexes (M = Pd, Pt; X = Cl, I; X' = Cl, Br, I). *Cryst. Growth Des.* **2006,** *6* (6), 1374-1379.
12. Ghonchepour, E.; Islami, M. R.; Tikdari, A. M., Efficient heterogenization of palladium by citric acid on the magnetite nanoparticles surface (Nano-Fe3O4@CA-Pd), and its catalytic application in C-C coupling reactions. *J. Organomet. Chem.* **2019,** *883*, 1-10.
13. Shi, S.; Meng, G.; Szostak, M., Synthesis of Biaryls through Nickel-Catalyzed Suzuki–Miyaura Coupling of Amides by Carbon–Nitrogen Bond Cleavage. *Angew. Chem. Int. Ed.* **2016,** *128* (24), 7073-7077.
14. Takahashi, R.; Kubota, K.; Ito, H., Air- and moisture-stable Xantphos-ligated palladium dialkyl complex as a precatalyst for cross-coupling reactions. *Chem. Commun.* **2020,** *56* (3), 407-410.
15. Ge, J.; Jiang, J.; Yuan, C.; Zhang, C.; Liu, M., Palladium nanoparticles stabilized by phosphine ligand for aqueous phase room temperature Suzuki-Miyaura coupling. *Tetrahedron Lett.* **2017,** *58* (12), 1142-1145.
16. Gao, P.; Szostak, M., Highly Selective and Divergent Acyl and Aryl Cross-Couplings of Amides via Ir-Catalyzed C–H Borylation/N–C(O) Activation. *Org. Lett.* **2020,** *22* (15), 6010-6015.
17. Kang, K.; Huang, L.; Weix, D. J., Sulfonate Versus Sulfonate: Nickel and Palladium Multimetallic Cross-Electrophile Coupling of Aryl Triflates with Aryl Tosylates. *J. Am. Chem. Soc.* **2020,** *142* (24), 10634-10640.
18. Ye, Z.; Chen, F.; Luo, F.; Wang, W.; Lin, B.; Jia, X.; Cheng, J., Palladium-Catalyzed Mizoroki-Heck-Type Reaction of Aryl Trimethoxysilanes. *Synlett* **2009,** *2009* (13), 2198-2200.
19. Jia, X.; Frye, L. I.; Zhu, W.; Gu, S.; Gunnoe, T. B., Synthesis of Stilbenes by Rhodium-Catalyzed Aerobic Alkenylation of Arenes via C–H Activation. *J. Am. Chem. Soc.* **2020,** *142* (23), 10534-10543.
20. Pape, S.; Daukšaitė, L.; Lucks, S.; Gu, X.; Brunner, H., An in Situ Generated Palladium on Aluminum Oxide: Applications in Gram-Scale Matsuda–Heck Reactions. *Org. Lett.* **2016,** *18* (24), 6376-6379.
21. Desai, S. P.; Ye, J.; Zheng, J.; Ferrandon, M. S.; Webber, T. E.; Platero-Prats, A. E.; Duan, J.; Garcia-Holley, P.; Camaioni, D. M.; Chapman, K. W.; Delferro, M.; Farha, O. K.; Fulton, J. L.; Gagliardi, L.; Lercher, J. A.; Penn, R. L.; Stein, A.; Lu, C. C., Well-Defined Rhodium–Gallium Catalytic Sites in a Metal–Organic Framework: Promoter-Controlled Selectivity in

Alkyne Semihydrogenation to E-Alkenes. *J. Am. Chem. Soc.* **2018,** *140* (45), 15309-15318.

22. Zhong, J.-J.; Liu, Q.; Wu, C.-J.; Meng, Q.-Y.; Gao, X.-W.; Li, Z.-J.; Chen, B.; Tung, C.-H.; Wu, L.-Z., Combining visible light catalysis and transfer hydrogenation for in situ efficient and selective semihydrogenation of alkynes under ambient conditions. *Chem. Commun.* **2016,** *52* (9), 1800-1803.
23. Mahmoudi, H.; Valentini, F.; Ferlin, F.; Bivona, L. A.; Anastasiou, I.; Fusaro, L.; Aprile, C.; Marrocchi, A.; Vaccaro, L., A tailored polymeric cationic tag–anionic Pd(ii) complex as a catalyst for the low-leaching Heck–Mizoroki coupling in flow and in biomass-derived GVL. *Green Chem.* **2019,** *21* (2), 355-360.
